# Comprehensive Mapping and Dynamics of Site-Specific Prolyl-Hydroxylation, Lysyl-Hydroxylation and Lysyl O-Glycosylation of Collagens Deposited in ECM During Zebrafish Heart Regeneration

**DOI:** 10.3389/fmolb.2022.892763

**Published:** 2022-06-16

**Authors:** Vivek Sarohi, Shriya Srivastava, Trayambak Basak

**Affiliations:** ^1^ School of Biosciences and Bioengineering (BSBE), Indian Institute of Technology (IIT)- Mandi, Mandi, India; ^2^ BioX Center, IIT-Mandi, Mandi, India

**Keywords:** collagens, post-translational modifications, O-glycosylation, mass-spectrometry, microheterogeneity, extra-cellular matrix, cardiac regeneration

## Abstract

Cardiac fibrosis-mediated heart failure (HF) is one of the major forms of end-stage cardiovascular diseases (CVDs). Cardiac fibrosis is an adaptive response of the myocardium upon any insult/injury. Excessive deposition of collagen molecules in the extracellular matrix (ECM) is the hallmark of fibrosis. This fibrotic response initially protects the myocardium from ventricular rupture. Although in mammals this fibrotic response progresses towards scar-tissue formation leading to HF, some fishes and urodeles have mastered the art of cardiac regeneration following injury-mediated fibrotic response. Zebrafish have a unique capability to regenerate the myocardium after post-amputation injury. Following post-amputation, the ECM of the zebrafish heart undergoes extensive remodeling and deposition of collagen. Being the most abundant protein of ECM, collagen plays important role in the assembly and cell-matrix interactions. However, the mechanism of ECM remodeling is not well understood. Collagen molecules undergo heavy post-translational modifications (PTMs) mainly hydroxylation of proline, lysine, and glycosylation of lysine during biosynthesis. The critical roles of these PTMs are emerging in several diseases, embryonic development, cell behavior regulation, and cell-matrix interactions. The site-specific identification of these collagen PTMs in zebrafish heart ECM is not known. As these highly modified peptides are not amenable to mass spectrometry (MS), the site-specific identification of these collagen PTMs is challenging. Here, we have implemented our in-house proteomics analytical pipeline to analyze two ECM proteomics datasets (PXD011627, PXD010092) of the zebrafish heart during regeneration (post-amputation). We report the first comprehensive site-specific collagen PTM map of zebrafish heart ECM. We have identified a total of 36 collagen chains (19 are reported for the first time here) harboring a total of 95 prolyl-3-hydroxylation, 108 hydroxylysine, 29 galactosyl-hydroxylysine, and 128 glucosylgalactosyl-hydroxylysine sites. Furthermore, we comprehensively map the three chains (COL1A1a, COL1A1b, and COL1A2) of collagen I, the most abundant protein in zebrafish heart ECM. We achieved more than 95% sequence coverage for all the three chains of collagen I. Our analysis also revealed the dynamics of prolyl-3-hydroxylation occupancy oscillations during heart regeneration at these sites. Moreover, quantitative site-specific analysis of lysine-O-glycosylation microheterogeneity during heart regeneration revealed a significant (*p* < 0.05) elevation of site-specific (K^1017^) glucosylgalactosyl-hydroxylysine on the col1a1a chain. Taken together, these site-specific PTM maps and the dynamic changes of site-specific collagen PTMs in ECM during heart regeneration will open up new avenues to decode ECM remodeling and may lay the foundation to tinker the cardiac regeneration process with new approaches.

## 1 Introduction

Cardiac fibrosis leading to heart failure has remained one of the major causes of death worldwide (González et al., 2018). Extracellular matrix (ECM) remodeling in the cardiac tissues is one of the main mechanisms of fibrosis development to an injury/insult in the myocardium (Li et al., 2018). Excessive deposition of collagen molecules in the ECM during cardiac fibrosis has remained the hallmark of fibrosis (Cowling et al., 2019). Initially, this fibrotic response helps in preventing the rupture of the ventricular wall of the heart (Cowling et al., 2019). However, in the mammalian hearts, this persistent fibrotic response progresses towards scar tissue formation, tissue-stiffening and ultimately resulting in heart failure (González et al., 2018). Contrastingly, in nature, some non-mammalian vertebrates such as zebrafish and urodele amphibians (Cutie and Huang, 2021) have mastered a unique exercise that allows their cardiomyocytes to undergo restricted dedifferentiation achieving cardiac regeneration. This exceptional property of adult zebrafish permits heart regeneration in case of a severe tissue insult or experimental amputation (Cutie and Huang, 2021). This unique ability makes Zebrafish an appropriate model system to study complex cellular and molecular processes underlying cardiac regeneration (Bootorabi et al., 2017). Under chronic/acute injury conditions, excess collagens and other proteins get deposited in the myocardium ECM inducing fibrosis (Travers et al., 2016) and eventually causing systolic and diastolic dysfunction. In the case of zebrafish, the fibrosis regression that has been documented as a transient phase occurs through fibroblast inactivation (Sánchez-Iranzo et al., 2018). The crucial understanding of participation and contribution of zebrafish heart ECM remains majorly unexplored. The ECM has recently gained attention to probe a better insight into the mechanistic changes during heart regeneration in zebrafish. In fact, the zebrafish heart ECM has been shown to have the potency to induce mammalian heart regeneration (Chen et al., 2016). Although the role of a few ECM components such as hyaluronic acid has been reported to play a major role in the epithelial-to-mesenchymal transition during regeneration (Missinato et al., 2015), the roles of major components of ECM such as collagens, glycoproteins, and proteoglycans are not well understood. Collagens and other cardiac ECM proteins are not just responsible for maintaining the architecture of the tissue but also play a vital role in mechanical and biochemical interactions that determine different cell behavior and functions (Frangogiannis, 2019).

In the ECM, collagens are the most abundant protein constituent (Di Lullo et al., 2002). Nauroy *et al*. have predicted 58 collagen genes contributing towards the zebrafish matrisome (Nauroy et al., 2018). Collagens are responsible for maintaining the structure and functions of cardiac tissues. These are also crucial in wound healing process (Willyard, 2018). Recently, ablation of COL1A2 in the zebrafish heart has been shown to impair cardiomyocyte regeneration (Sánchez-Iranzo et al., 2018). Other collagen chains such as COL7A11 and COL8A2 present in zebrafish heart ECM have been found upregulated during heart regeneration (Sánchez-Iranzo et al., 2018). Collagens are heavily post-translationally modified triple-helical molecules that form the interstitial fibers of the myocardium. Collagen post-translational modifications (PTMs) play a significant role in the function and stability of the triple helix. The proline residues are the most abundant amino acids found in collagen chains (Myllyharju, 2005). Prolines present in collagen chains are 4-hydroxylated (4-HyP) generally in the “Y” position of the “Gly-Xaa-Yaa” motif, providing the structural mobility for triple helix formation. In addition to this, there are proline residues also present in the “X” position of the “Gly-Xaa-HyP” motif that are commonly 3-hydroxylated (3-HyP) in nature. However, the occurrence of 3-HyP is rarer compared to 4-HyP (Weis et al., 2010). These prolyl hydroxylations are catalyzed by a specific class of prolyl hydroxylases (Hudson and Eyre, 2013; Merl-Pham et al., 2019; Salo and Myllyharju, 2021). Recently, it has been documented that genetic deletion of prolyl-3-hydroxylase 2 (P3H2) in mice evidenced that abolishment of prolyl-3-hydroxylations in basement membrane collagen could be involved in regulating platelet aggregation and development of eye tissues (Pokidysheva et al., 2014; Hudson et al., 2015). Osteogenesis Imperfecta is one of the reported diseases resulting from a lack of 3-hydroxyproline in COL1A1 (P^1164^) (Morello et al., 2006; Cabral et al., 2007).

Apart from proline hydroxylation, lysine hydroxylation, and glycosylation are also critically important for the proper functioning of collagens. In collagen chains, lysine residues occurring within the Gly-Xaa-Lys motif are likely to be hydroxylated by different family members of the lysyl hydrolase; lysyl hydroxylase 1 (LH1), LH2, and LH3. Once hydroxylated, these hydroxylysines (HyK) can further be O-linked glycosylated by the addition of monosaccharides and/or disaccharides by specific glycosyltransferases (Kivirikko et al., 1973; Risteli and Kivirikko, 1974; Schegg et al., 2009; Scietti et al., 2018). Lysine (found in the Gly-Xaa-Lys motif) could be present in four different forms in collagen molecules deposited in the ECM. Unmodified lysine (K), hydroxylysine (HyK), galactosyl-hydroxylysine (G-HyK), and glucosyl-galactosyl-hydroxylysine (GG-HyK) occurring on a single site of the collagen chain. This site-specific variation of lysine residues is termed lysine microheterogeneity. Altered levels of lysine microheterogeneity in collagens lead to dysfunction in the tissues. Musculoskeletal defects (Geister et al., 2019), connective tissue disorder (Salo et al., 2008), and cerebral small vessel disease (Miyatake et al., 2018) are reported to have altered hydroxylysine and lysyl O-glycosylation levels in collagens. These studies indicate the significant role of collagen PTMs in maintaining ECM and tissue homeostasis. ECM homeostasis is remodeled upon injury to the myocardium and thereby ECM remodeling holds the key to the regeneration of amputated zebrafish hearts. Collagens are the most abundant components of ECM and play a significant role in the regeneration of zebrafish hearts. Various functions of collagen are dependent on site-specific PTMs (Sipilä et al., 2007; Jürgensen et al., 2011; Stawikowski et al., 2014). More importantly, these site-specific collagen PTMs could be tissue-specific which demands rigorous exploration in this field.

In the context of zebrafish cardiac tissue regeneration, the dynamics of the site-specific collagen PTM network are still unexplored. Therefore, to comprehensively map the site-specific collagen PTMs constituting the myocardium ECM and to understand the dynamics of collagen PTMs during the regeneration process, we found an appropriate proteomics study performed by Garcia-Puig et al. (2019) on zebrafish heart regeneration. To bridge this gap we downloaded this publicly available data set (PXD011627 and PXD010092) and generated a comprehensive map of site-specific collagen PTMs in adult zebrafish hearts. Furthermore, we quantitated the occupancy levels of collagen PTMs during the regeneration process. Understanding the dynamics of site-specific collagen PTMs in cardiac extracellular matrix during heart regeneration will open up new avenues to decode ECM remodeling and may lay the foundation to tinker the cardiac regeneration process with new approaches.

## 2 Methods

### 2.1 Mass Spectrometry Data Source

In this study, two publicly available datasets were utilized. The first dataset with identifier “PXD011627” and the second dataset with identifier “PXD010092” were both submitted by Garcia-Puig et al. (2019) in ProteomeXchange. In this study, Garcia-Puig *et al*. established the zebrafish myocardium ECM enrichment protocol by decellularizing zebrafish ventricular tissue samples (Garcia-Puig et al., 2019). Moreover, this study assessed the dynamic changes in the ECM proteome of regenerating zebrafish hearts using an amputation model. The samples of regenerating heart ventricles were taken at 7-, 14- and 30-days post-amputation (DPA). Ventricular decellularization was done using SDS and Triton-X. The sham model was used as a control in their study.

In their study, MS data acquisition was done differently for the two datasets. Easy-nLC 1,000 (Thermo) was used for liquid chromatography separation. Nanoflex (Thermo) was used as an ESI source and LC-MS/MS was done on Q-exactive HF mass spectrometer (Thermo) with HCD fragmentation and the data-dependent acquisition opted for dataset “PXD011627”. For dataset “PXD010092” a nano HPLC system (Proxeon) coupled with Maxis Impact (Bruker) Q-TOF mass spectrometer was used and each sample of regenerating and sham model heart ventricle was analyzed using LC-MS in duplicates. Furthermore, healthy human heart sample (ECM) mass-spectrometry data from Barallobre-Barreiro *et al*. (PXD028908) (Barallobre-Barreiro et al., 2021), and mice heart sample (ECM) mass-spectrometry data from Padmanabhan *et al* (PXD002488) (Padmanabhan Iyer et al., 2016) was reanalyzed for the site-specific collagen PTM identification.

### 2.2 Raw Data Description

The publicly available datasets “PXD011627” and “PXD010092” were downloaded from ProteomeXchange. The complete *.d folders for “PXD010092” were shared by Prof. Angel Raya’s research group. Dataset “PXD011627” contains 9 (.raw) files and Dataset “PXD010092” contains 8 (.d) files, corresponding to two files (biological replicate) each for sham, 7-, 14- and 30-days post-amputation samples. Dataset “PXD010092” was not compatible with search engines because (.d) files are not readily accepted by many search engines. To overcome this, (.d) files were converted into. mgf and. mzML format using MSConvert with turbocharger filter. These converted files were further used as inputs by search engines MyriMatch (Tabb et al., 2007) and MSFragger (Kong et al., 2017), respectively. We also downloaded 60 (.raw) files from Barallobre-Barreiro *et al* (PX028908) and 6 (.raw) files from Padmanabhan *et al*. (PXD002488).

### 2.3 Database Search for the Identification of Collagen Chains and PTMs (Hydroxylation of Proline and Lysine, O-Glycosylation of Lysines) Using MyriMatch

Two different search engines were employed to reanalyze these datasets to identify collagen PTMs from the zebrafish myocardium ECM. All the database searches were performed on the high-performance computing (HPC) facility at the Indian Institute of Technology (IIT)- Mandi. First, a general database search was performed on raw MS data (*.raw files) of dataset “PXD011627” with a complete *Danio rerio* uniprot database having 62,593 entries, downloaded on 20 October 2020. For the PXD028908 dataset, a general database search was performed on raw MS data (*.raw files) with a complete *Homo sapiens* uniprot database having 20,396 entries, downloaded on 16 February 2021. For the PXD002488 dataset, a general database search was performed on raw MS data (*.raw files) with a complete *Mus musculus* uniprot database having 17,090 entries, downloaded on 04 December 2021. Precursor ion tolerance was set at 10 ppm and fragment ion m/z tolerance was allowed up to 20 ppm for zebrafish and human data. However, for the mice dataset, a fragment ion tolerance of 50 ppm was used. Carbamidomethylation (+57.0236) on cysteine was used as static modification and methionine oxidation (+15.9949) and hydroxyproline (+15.9949) were used as dynamic modifications with up to 4 maximum dynamic modifications per peptide. A maximum of 2 missed cleavages were allowed for fully tryptic digestion. MyriMatch searches were performed to generate *.pepXML files for respective datasets. These *.pepXML files were further grouped for PSM matches, peptide, and protein group identification using IDPicker with <1% FDR. After a general database search, the identified list of ECM proteins from zebrafish and humans was exported from IDPicker as a *.FASTA database. This *.FASTA database (separately for zebrafish, humans, and mice) was used as a subset database to perform an in-depth collagen PTM search. In the subset database search, up to 4 missed cleavages for fully tryptic digestion were allowed with a similar precursor (10 ppm) and fragment ion tolerance (20 ppm) tolerance. For mice MS data, a fragment ion tolerance of 50 ppm was used. As mentioned previously, (Basak et al., 2016; Merl-Pham et al., 2019), a “Gly-Xaa-Yaa” based motif-specific PTM search strategy was employed using the MyriMatch motif-specific module for the identification of collagen PTMs. Carbamidomethylation on cysteine was used as static modification and methionine oxidation, hydroxyproline (P! +15.9949), hydroxylysine (GXK! +15.994,916), galactosyl-hydroxylysine (GXK! 178.047738) and glucosylgalactosyl-hydroxylysine (GXK! 340.100,562) were used as dynamic modifications with up to 10 maximum dynamic modifications per peptide. IDPicker was used for grouping the *.pepXML output file by controlling FDR at a 1% level for PSMs, peptides, and proteins. 3-hydroxyproline modifications were only considered in case a proline residue was found to be hydroxylated at the “X” position of a “G-Xaa-HyP” motif in the collagen chains. Furthermore, pLabel was used for manual inspection, analysis, and validation of subset database search PSMs for assigning a specific collagen PTM containing peptide.

### 2.4 Database Search for the Identification of Collagen Chains and PTMs (Hydroxylation of Proline and Lysine, O-Glycosylation of Hydroxylysine Sites) Using MSFragger*

mzML files were used for MSFragger mediated database search. In MSFragger based pipeline, the decoy database was generated by a philosopher from the same uniprot database (*Danio rerio*, 62,593 entries, downloaded on 20 October 2020) as in the case of the MyriMatch general search. Precursor tolerance and fragment ion tolerance were kept at 50 and 25 ppm, respectively. PSM, peptide, and protein level FDR were kept at < 1%, with a maximum of three missed cleavages of fully tryptic peptides. Carbamidomethylation (+57.0236) on cysteine was used as static modification and methionine oxidation (+15.9949), hydroxyproline (+15.9949), N-terminal acetylation (+42.010565), hydroxylysine (+15.994,916), galactosyl-hydroxylysine (+178.047738) and glucosylgalactosyl-hydroxylysine (+340.100,562) were used as dynamic modifications with up to 5 maximum dynamic modification per peptide. IDPicker was used for grouping the *.pepXML output file generated from MSFragger searches by controlling FDR at <1% level for PSMs, peptides, and proteins. Similar to MyriMatch-based workflow, pLabel was used for manual inspection and validation for assigning a specific PSM to identify site-specific collagen PTM containing peptides.

### 2.5 Relative Abundances of Collagen Chains in the Heart ECM During Regeneration

Spectral counts (referred to the total number of peptides detected at ∼1% FDR per protein group) from the results of the MyriMatch database search were exported from IDPicker. These spectral counts were normalized with the total spectral counts of each raw MS file ID. The normalized spectral counts corresponding to unique protein groups were further used to quantitate the relative abundance of collagens in wild-type and regenerating zebrafish hearts. Additionally, the same normalized spectral counts data for collagens from MyriMatch database search results were used to generate a heatmap of relative collagen expression during regeneration. Collagen chains identified with ≥3 normalized spectral counts were only considered for the heatmap representation.

### 2.6 Quantitation of Occupancy Level of Collagen PTM Sites Using Skyline

Database search results (*.pepXML files) generated by either MyriMatch and/or MSFragger were parsed through PeptideProphet (TPP pipeline module) for importing the probability scores (0-1) (Pedrioli, 2010). After parsing the *.pepXML files through PeptideProphet, these *.pepXML files were utilized to build a spectral library (.blib) using open-source Skyline (MacLean et al., 2010). A spectral library was used in Skyline for extracting the raw abundance intensity of unmodified and different forms of modified collagen peptides. Occupancy calculation of prolyl hydroxylation sites and microheterogeneity (unmodified, hydroxylated, O-glycosylation of lysines) of lysine sites were performed using MS^1^ based targeted extraction pipeline as described previously (Merl-Pham et al., 2019).

### 2.7 Statistical Analysis

GraphPad prism was used for statistical analysis for calculating the dynamics of different collagen site-specific PTM level quantitation. ANOVA was performed and a *p* value < 0.05 was considered statistically significant.

## 3 Results and Discussion

Myocardium extracellular matrix (ECM) consisting of collagens as a major component, is sought to be one of the key molecules involved in the remodeling process during zebrafish heart regeneration (Sánchez-Iranzo et al., 2018). Collagen and its types are typically triple-helical, large protein molecules secreted in the extracellular space and get assembled to form fibrils in the interstitium of the tissue responsible for structural support, cell-ECM interactions, and cell behavior. (Basak et al., 2016).

### 3.1 Identification of Collagen Chains, and Their Abundances in Wild Type Zebrafish Heart ECM-

**FIGURE 1:**
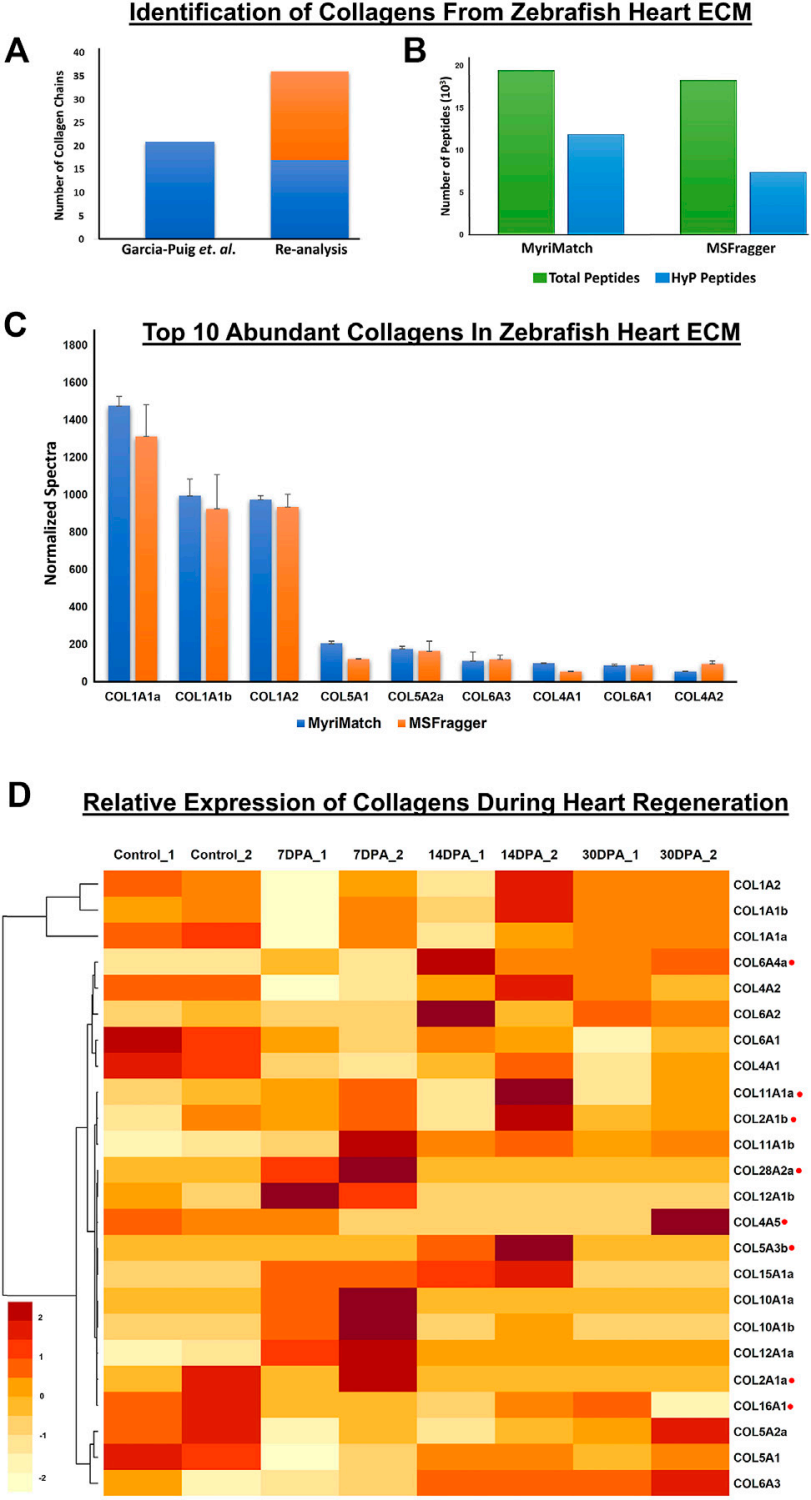
Identification of collagen chains and their relative abundances in zebrafish heart ECM: **(A)** Total number of collagen chains (Cabral et al., 2007) identified previously by Garcia-Puig et al. compared to (Padmanabhan Iyer et al., 2016) number of collagen chains identified in our analysis. **(B)** depicts inclusion of hydroxyproline (HyP) modification in the database search by MyriMatch and MSFragger resulting in identification of almost 61.12 and 40.46% (summed number from all the raw *.pepXML files used for database search) new unique peptides. This strategy yielded more no. of peptide identification resulting in a higher number of collagen chain identification from the same dataset. **(C)** Top 10 abundant collagen chains deposited in the zebrafish heart ECM were identified by two different search engines MyriMatch and MSFragger respectively. **(D)** Relative abundances of different collagen chains during zebrafish heart regeneration are shown by the heatmap. Light yellow represents the lower value (−2) and dark red represents the higher value (+2) in the row. Normalized spectral count values have been used to generate the heatmap (considering ≥ 3 spectral counts per chain). collagen chains marked with red (.) dots are quantitated during regeneration in re-analysis for the first time (DPA = day post amputation).

To identify and quantitate the abundance of collagen chains present in wild-type zebrafish heart ECM, a dual database search engine-based (MyriMatch and MSFragger) comprehensive strategy was implemented. MyriMatch (Tabb et al., 2007) and MSFragger (Kong et al., 2017) were utilized in this study for in-depth identification of collagen chains present in the myocardium ECM of zebrafish from the publicly available datasets (PXD011627, PXD010092) as mentioned earlier. This analytical pipeline identified a total of 36 collagen chains present in the ECM of the zebrafish heart (Figure 1A; Supplementary Table S1). Out of these 36 collagen chains, 17 are commonly identified and 19 collagen chains are newly identified in the ECM of zebrafish heart (Figure 1A, Supplementary Table S1). Previously, Garcia-Puig et al. identified a total of 21 collagen chains from the enriched ECM of zebrafish hearts using this dataset (Garcia-Puig et al., 2019). Since collagens are heavily modified with prolyl hydroxylation, a standard proteomic data analysis pipeline will not be able to identify hydroxylated peptides of collagen chains present in the raw data (Merl-Pham et al., 2019). However, in our analytical pipeline, we included prolyl-hydroxylation as a dynamic modification in the database search yielding a higher number of collagen chain identification. Database searches yielded almost 19,456 and 18,274 total peptide ids from MyriMatch and MSFragger respectively. As expected, almost 61 and 40% of these identified peptides contained prolyl-hydroxylation (Figure 1B, Supplementary Table S2). Thus, it substantiates the use of prolyl-hydroxylation as an important “dynamic modification” in order to explore ECM mass-spectrometry data to increase the identification of collagen chains along with their increased sequence coverage. Normalized spectral counts were used to calculate the abundance of the top 10 collagen chains present in the ECM of wild-type zebrafish hearts (Figure 1C). Both MyriMatch and MSFragger unambiguously yielded COL1A1a, COL1A1b, and COL1A2 chains of collagen 1 to be the highest abundant collagen chains present in the cardiac ECM of zebrafish. These three chains form the triple-helical collagen 1 protomers (Gistelinck et al., 2016), the most abundant protein present in the extracellular matrix of wild-type zebrafish hearts. Apart from Collagen 1, COL5A1, COL5A2a, COL6A3, COL4A1, COL6A1, and COL4A2 chains were found to be in the 10 most abundant collagen chains present in the ECM of WT zebrafish heart. These abundance plots yielded from two different search engines were in agreement highlighting the robustness of the analysis.

### 3.2 Relative Abundances of Collagen Chains in the Heart ECM During Regeneration–

The cardiac regeneration process involves ECM remodeling. Being the most abundant component of ECM (Di Lullo et al., 2002), levels of collagens are also altered during regeneration (Garcia-Puig et al., 2019) (Sánchez-Iranzo et al., 2018). As expected, normalized spectral counts of both the search engines show that Collagen 1 (COL1A1a, COL1A1b, and COL1A2) is most abundant in zebrafish heart ECM during 7, 14, and 30 days post-amputation (Supplementary Figure S1). Here, we specifically focused to showcase the relative changes of different collagen chains present in the ECM during regeneration (Figure 1D, Supplementary Table S3). Out of the total 36 collagen chains identified, relative abundances were calculated for 24 collagen chains summarized in the heatmap presented in Figure 1D (raw data for individual chains are provided in Supplementary Table S3). Interestingly, the level of total collagen 1 (summed abundances of all three chains of the triple-helix) decreased (to 0.68 fold, 32% decrease in the level, not significant) at 7 days post-amputation, was slightly increased at 14 days post-amputation (0.86 fold compared to control, 14% decrease in level compared to sham control model) and it also further increased (0.93 fold compared to sham model (control), 6.2% decrease in level compared to sham model) at 30 days post-amputation. Although these changes were not found to be statistically significant, the expression trend corroborated the previous analysis (Garcia-Puig et al., 2019). A similar expression was found for COL5A1 and COL5A2a during the regeneration process. Levels of these 2 collagen chains were first decreased at 7 days post-amputation and then increased at 14 and 30 days post-amputation (Garcia-Puig et al., 2019). The expression of COL4A2, COL5A1, and COL5A2a in our analysis is also similar to the analysis done by Garcia-Puig et al. (2019) in which they found a decrease in the level of COL4A2, COL5A1, and COL5A2a on 7 days post-amputation indicating a feedback post-transcriptional regulation. Furthermore in this analysis, we quantitated the relative abundances of 8 new collagen (COL6A4, COL11A1, COL2A1b, COL28A2a, COL4A5, COL5A3b, COL2A1a, COL1A1) (Figure 1D, marked with red dots) chains present in the myocardium ECM during heart regeneration. The oscillatory dynamics of abundances of these new collagen chains are depicted in the heatmap (Figure 1D, Supplementary Table S3). These expressions of collagen chains highlight the role of collagen in the extracellular remodeling during regeneration.

### 3.3 Global Characterization and Identification of Novel Site-specific Collagen Post-translational Modifications (PTMs) in Zebrafish Myocardium ECM

**FIGURE 2:**
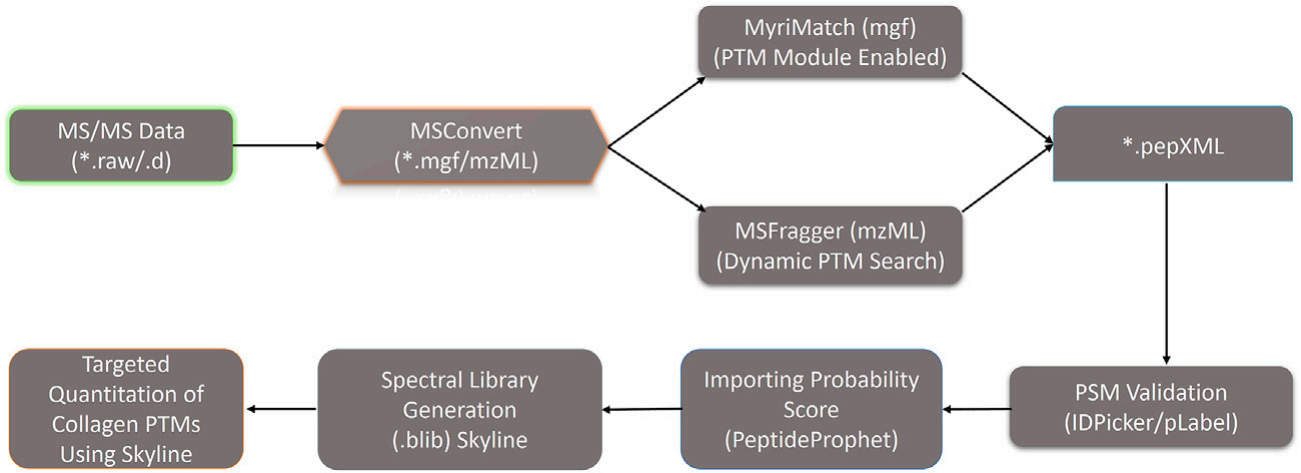
Optimized dual database search engine-based MS analysis pipeline for the global identification and quantitation of site-specific collagen PTMs from zebrafish heart ECM. Thermo. raw or Bruker. d MS/MS files were initially converted to. mgf and. mzML files (by MSConvert) respectively and searched with MyriMatch and MSFragger to identify the collagen present in the zebrafish heart ECM. For MyriMatch, the subset of identified proteins was used as a second database to perform a PTM module enabled search defining specific sequence motifs for the site-specific identification of collagen PTMs in zebrafish heart ECM. For MSFragger, the PTM searches were conducted directly with the entire zebrafish database. From MyriMatch and MSFragger *.pep.XML output files containing each peptide spectrum match (PSM) were further parsed by PeptideProphet to compute the probability score (0,1). The *.pep.XML output file parsed by PeptideProphet was further imported into Skyline along with all the raw MS/MS files in to generate the spectral library (.blib). This spectral library (.blib) in Skyline was used for the targeted MS^1^-based extraction of all the PTM modified and unmodified collagen peptide species for each specific site. The area of MS^1^ area for each peptide for different samples was computed from Skyline.

Collagen PTMs such as O-glycosylation (on hydroxylysine residues) and hydroxylation (on lysine and proline residues) have been shown to be crucial for embryonic development, assembly of ECM fibrils, and cell-matrix interactions (Pokidysheva et al., 2014; Hudson et al., 2015). Collagen molecules get heavily post-translationally modified during the biosynthesis of new chains in the endoplasmic reticulum (Hennet, 2019). Recently, the use of a hydroxyproline-based search strategy using mass-spectrometry data yielded increased coverage for collagen chains (Merl-Pham et al., 2019; Basak et al., 2016; Shao et al., 2020). However, it is challenging to identify the site-specific O-glycosylation sites present in the big collagen chains (Basak et al., 2016). The role of collagen PTMs during tissue regeneration is completely unexplored. Classical amino acid analysis-based approaches could throw insights regarding the composition (including modified amino acids) of these collagen triple helices. However, site-specific identification of these collagen PTMs (hydroxylation, O-glycosylation)–can only be achieved by high-resolution mass spectrometry. So, this study primarily focused on the identification of site-specific collagen PTMs present in the ECM of the zebrafish heart. Furthermore, the dynamics of these PTMs in collagen I (most abundant collagen in heart ECM, a heteromeric trimer constituting three different chains COL1A1a, COL1A1b, and COL1A2) is being showcased during the regeneration of zebrafish heart. A total of 36 collagen chains were identified from the zebrafish heart ECM in this analysis as mentioned previously. Identification of collagen PTMs from a global MS analysis, without biochemical purification, has remained challenging. We had previously developed a MyriMatch-based workflow to identify site-specific collagen PTMs from crude ECM proteome preparations using high-resolution mass spectrometry (Basak et al., 2016). Here, our *inhouse* collagen PTM identification pipeline (Figure 2) with a dual search engine-based strategy maximized the identification of prolyl-hydroxylation, lysyl-hydroxylation, and O-glycosylation sites present in 23 collagen chains from zebrafish heart ECM (Table 1). We detected a total of 95 3-hydroxyproline, 108 hydroxylysine, 29 galactosyl-hydroxylysine, and 128 glucosylgalactosyl-hydroxylysine sites in 23 collagen chains (Table 1) present in the zebrafish heart ECM. This is the first catalog of site-specific collagen PTMs identified in the ECM of the zebrafish heart. Notably, site-specific collagen PTMs were identified in the highest abundant COL1A1a, along with COL11A1a and COL22A1 spanning across three orders of magnitude (log scale) (see Table 1). This highlights the sensitivity and the depth of dynamic range of our analytical pipeline to explore the PTMs of collagens present in the ECM of zebrafish heart. Collagen I seemed to have a higher number of prolyl-3-hydroxylations and lysyl-hydroxylation sites. Collagen 5 is a minor constituent of fibril assembly (Wenstrup et al., 2004). However, this analysis revealed 12 (P^686^, P^746^, P^824^, P^1112^, P^1115^, P^1163^, P^1166^, P^1190^, P^1193^, P^1253^, P^1421^, P^1424^) and nine sites (P^241^, P^277^, P^532^, P^661^, P^868^, P^901^, P^937^, P^967^, P^1105^) of 3-HyP in COL5A1 and COL5A2a, respectively. COL5A2a was then further mapped with 11 sites (K^329^, K^338^, K^500^, K^557^, K^572^, K^761^, K^761^, K^794^, K^638^, K^803^, K^887^) of glucosyl-galactosyl hydroxylysines. Only one galactosyl-hydroxylysine site (K^1344^) was identified in COL5A1. As previously shown in the zebrafish genome (from fin tissue) analysis (Duran et al., 2015), no type III collagen chain is detected in our analysis from the zebrafish cardiac ECM. The absence of type III collagen is a unique feature of zebrafish heart ECM and warrants further scope to investigate the role of collagen III during regeneration. Four different chains of collagen 6 (COL6A1, COL6A2, COL6A3, COL6A4a) were found to harbor O-glycosylation but only one prolyl-3-hydroxylation site (3-HyP^470^ in COL6A2) (Table 1). Although basement membrane collagen IV is not a major constituent of heart ECM; in the case of collagen IV alpha 1 only seven sites (K^463^, K^466^, K^882^, K^909^, K^1149^, K^1179^, K^1182^) of glucosyl-galactosyl-hydroxylation were identified. Surprisingly, 24 glucosyl-galactosyl-hydroxylation sites were identified in COL4A2. A total of 4 (P^201^, P^204^, P^294^, P^297^, p^1338^) and 3 (P^339^, P^555^, P^613^) prolyl-3-hydroxylation sites were identified in COL4A1 and COL4A2, respectively. It appeared that COL4A2 is way more glycosylated than COL4A1 in the zebrafish cardiac ECM compared to mice (Basak et al., 2016). Interestingly, we have also identified the COL4A5 chain with a common hydroxylysine site harboring microheterogeneity of galactosyl-hydroxylysine and glucosyl-galactosyl-hydroxylysine (K^225^). Additionally, many other sites of prolyl-3-hydroxylations, lysyl-hydroxylation and O-glycosylation sites of lysine have been identified in various other collagen types (COL11A1b, COL7A1, COL22A1, etc.; see Table 1) highlighting the advantageous nature of these analyses increasing the depth of site-specific PTM identification coverage in the ECM. Taken together, these results showcased the comprehensive mapping and site-specific identification of many collagens present in the zebrafish heart ECM.

**TABLE 1:** Mass-Spectrometry-based identification of site-specific hydroxylation and glycosylation of different collagens present in the zebrafish cardiac ECM.

Collagen chains	3-Hydroxyproline Sites (95)	Hydroxylysine Sites (108)	Galactosyl-Hydroxylysine Sites (29)	Glucosylgalactosyl-Hydroxylysine Sites (128)
COL1A1a	P^176^, P^188^, P^317^, P^401^, P^443^, P^446^, P^551^, P^623^, P^707^, P^755^, P^800^, P^854^, P^869^, P^878^, P^881^, P^911^, P^980^, P^992^, P^1034^, P^1103^, P^1106^, P^1148^, P^1166^, P^1169^	K^261^, K^270^, K^336^, K^381^, K^414^, K^426^, K^432^, K^504^, K^522^, K^570^, K^596^, K^641^, K^693^, K^726^, K^735^, K^765^, K^810^, K^819^, K^846^, K^918^, K^1017^, K^1080^, K^1191^	K^261^, K^270^, K^432^, K^504^, K^570^, K^693^, K^819^, K^846^, K^1191^	K^261^, K^270^, K^432^, K^504^, K^522^, K^570^, K^693^, K^726^, K^819^, K^846^, K^1017^
COL1A1b	P^176^, P^191^, P^302^, P^320,^ P^404^, P^470^, P^554^, P^758^, P^872^, P^881^, P^914^, P^938^, P^983^, P^1031^, P^1109^, P^1151^, P^1175^	K^215^, K^264^, K^273^, K^339^, K^384^, K^417^, K^429^, K^525^, K^573^, K^618^, K^696^, K^738^, K^746^, K^768^, K^813^, K^849^, K^900^, K^1020^, K^1193^	K^264^, K^273^, K^849^, K^1020^	K^264^, K^273^, K^339^, K^849^
COL1A2	P^56^, P^59^, P^244^, P^286^, P^361^, P^451^, P^469^, P^673^, P^718^, P^787^, P^820^, P^838^, P^925^, P^1066^, P^1081^	K^74^, K^179^, K^188^, K^254^, K^299^, K^344^, K^350^, K^454^, K^488^, K^500^, K^533^, K^578^, K^644^, K^647^, K^683^, K^731^, K^737^, K^836^, K^935^, K^998^, K^1004^	K^74^, K^188^, K^254^, K^344^, K^350^, K^644^	K^74^, K^167^, K^188^, K^254^, K^299^, K^578^, K^644^, K^647^, K^935^
COL4A1	P^201^, P^204^, P^294^, P^297^, p^1338^			K^463^, K^466^, K^882^, K^909^, K^1149^, K^1179^, K^1182^
COL5A1	P^686^, P^746^, P^824^, P^1112^, P^1115^, P^1163^, P^1166^, P^1190^, P^1193^, P^1253^, P^1421^, P^1424^	K^1134^, K^1296^, K^1329^, K^1497^_,_ K^1578^, K^1581^, K^1592^, K^1604^, K^1644^	K^1344^	K^623^, K^626^, K^753^, K^813^, K^945^, K^1104^, K^1209^, K^1245^, K^1290^, K^1338^, K^1383^, K^1455^_,_ K^1482^_,_ K^1578^, K^1581^
COL5A2a	P^241^, P^277^, P^532^, P^661^, P^868^, P^901^, P^937^, P^967^, P^1105^	K^280^, K^329^, K^449^, K^482^, K^494^, K^709^, K^749^, K^803^, K^878^, K^986^, K^1036^, K^1078^, K^1124^, K^1135^		K^329^, K^338^, K^500^, K^557^, K^572^, K^761^, K^761^, K^794^, K^638^, K^803^, K^887^
COL6A1	P^574^, P^577^	K^482^, K^538^		K^398^, K^488^, K^538^, K^560^, K^563^
COL6A3		K^1796^, K^1975^		K^1660^, K^1778^, K^1796^, K^1711^, K^1963^, K^1880^, K^1889^
COL6A2	P^470^	K^387^	K^539^	K^339^, K^387^, K^426^, K^435^, K^438^, K^456^, K^459^, K^539^, K^545^
COL11A1a	P^919^	K^1313^	K^1217^, K^1304^	K^911^, K^1148^, K^1151^
COL4A2	P^339^, P^555^, P^613^	K^593^, K^704^, K^707^, K^1620^, K^1651^	K^689^	K^54^, K^72^, K^318^, K^567^, K^582^, K^593^, K^662^, K^674^, K^689^, K^704^, K^707^, K^725^, K^948^, K^951^, K^969^, K^1045^, K^1048^, K^1076^, K^1235^, K^1247^, K^1283^, K^1292^, K^1331^, K^1346^
COL4A5	P^93^	K^127^, K^214^	K^225^	K^225^
COL6A4a	-	-	K^1501^	K^1490^, K^1787^, K^1796^, K^2148^
COL2A1a	-	K^612^	K^423^	K^423^, K^1178^
COL2A1b				
COL16A1	-	K^1008^, K^1343^, K^1495^, K^1498^	-	K^644^, K^654^, K^1008^, K^1233^
COL11A1b	-	-	-	K^1191^, K^1400^
COL5A2b	P^838^	-	-	K^731^
COL11A2	P^1133^	K^959^, K^963^	-	K^1188^, K^1197^, K^1200^, K^1203^
COL7A1	-	-	-	K^1504^, K^2136^
COL5A3b	P^437^, P^467^	-	-	-
COL22A1	-	K^495^, K^1128^	K^788^, K^1538^	K^498^, K^513^, K^785^
COL17A1a	P^1156^	-	-	-

### 3.4 Characterization of PTMs in Collagen I From the Zebrafish Heart ECM

Collagen I is the highest abundant ECM protein present in the zebrafish hearts (Gistelinck et al., 2016). The composition of the heterotrimer of the collagen I chain forming a protomer (triple helices) is different compared to mammals (Gistelinck et al., 2016). In mammals, two alpha 1 chain and one alpha 2 chain form the protomer. However, in zebrafish, three different genes (COL1A1a, COL1A1b, and COL1A2) code for three different collagen I chains (collagen alpha 1a, collagen 1 alpha 1b (alpha 3), and collagen 1 alpha 2), respectively. High expression of these three genes has been documented in adult zebrafish tissues as well as during embryonic and larval development indicating equimolar stoichiometry in forming the protomers (Gistelinck et al., 2016). However, different glycosylated forms of COL1A1a have been found in zebrafish embryos indicating altered PTM levels during development (Gistelinck et al., 2016). Thus, tissue-specific mapping of collagen I site-specific PTMs are of the highest importance in order to further delineate their function. Here, we have comprehensively mapped the site-specific prolyl-hydroxylation, lysyl-hydroxylation, and lysine O-glycosylation in three different chains (COL1A1a, COL1A1b, and COL1A2) of collagen I in zebrafish heart ECM.

#### 3.4.1 Comprehensive Site-specific Map of PTMs in COL1A1a From the Zebrafish Heart ECM–

**FIGURE 3:**
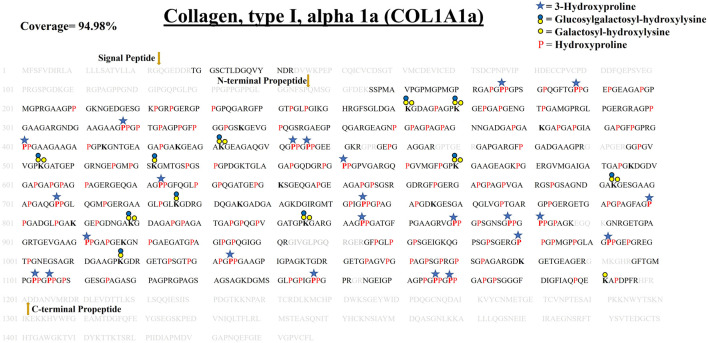
Comprehensive map of proline/lysine hydroxylation sites and lysine O-glycosylation sites in COL1A1a of WT zebrafish heart ECM. Identified peptide sequence in the proteomic analysis is shown in black color, sequence not identified in this analysis are shown in grey color. A total of 94.98% sequence coverage of COL1A1a is detected (considering the matured form of COL1A1The signalgnal peptide is 22 amino acids (1–22) long. Sequence alignment matching with human COL1A1 revealed the propeptide cleavage sites. Dark yellow arrows show N terminal (23–146) and C-Term (1,202–1,447) propeptide cleavage sites. As shown in the top right corner, red bold “**P**” with a blue star represents 3-hydroxyproline on the Xaa position followed by 4-hydroxyproline the on Yaa position in the Gly-Xaa-Yaa motif. 4-hydroxyproline on Yaa position is represented with red color “P”. Hydroxyproline on unusual Xaa position with (Ala, Val, Met, Ile, Ser, Glu, Arg, and Asp) on Yaa position are also identified but cannot label either 3-hydroxyproline or 4-hydroxyproline. Hydroxylysine sites are presented by bold “**K**”. Lysine sites highlighted with a yellow circle represents galactosyl-hydroxylysine sites and yellow plus blue coloued circles represent glucosylgalactosyl-hydroxylysine sites. The presence of glucosylgalactosyl-hydroxylysine, galactosyl-hydroxylysine, and hydroxylysine on the same site shows lysine microheterogeneity. A summary of these site-specific PTMs of COL1A1a is presented in Table 1, and all the PSMs for O-glycosylated lysine and 3-hydroxyproline sites are provided in Supplementary Figure S2.1-S2.21.

**FIGURE 4:**
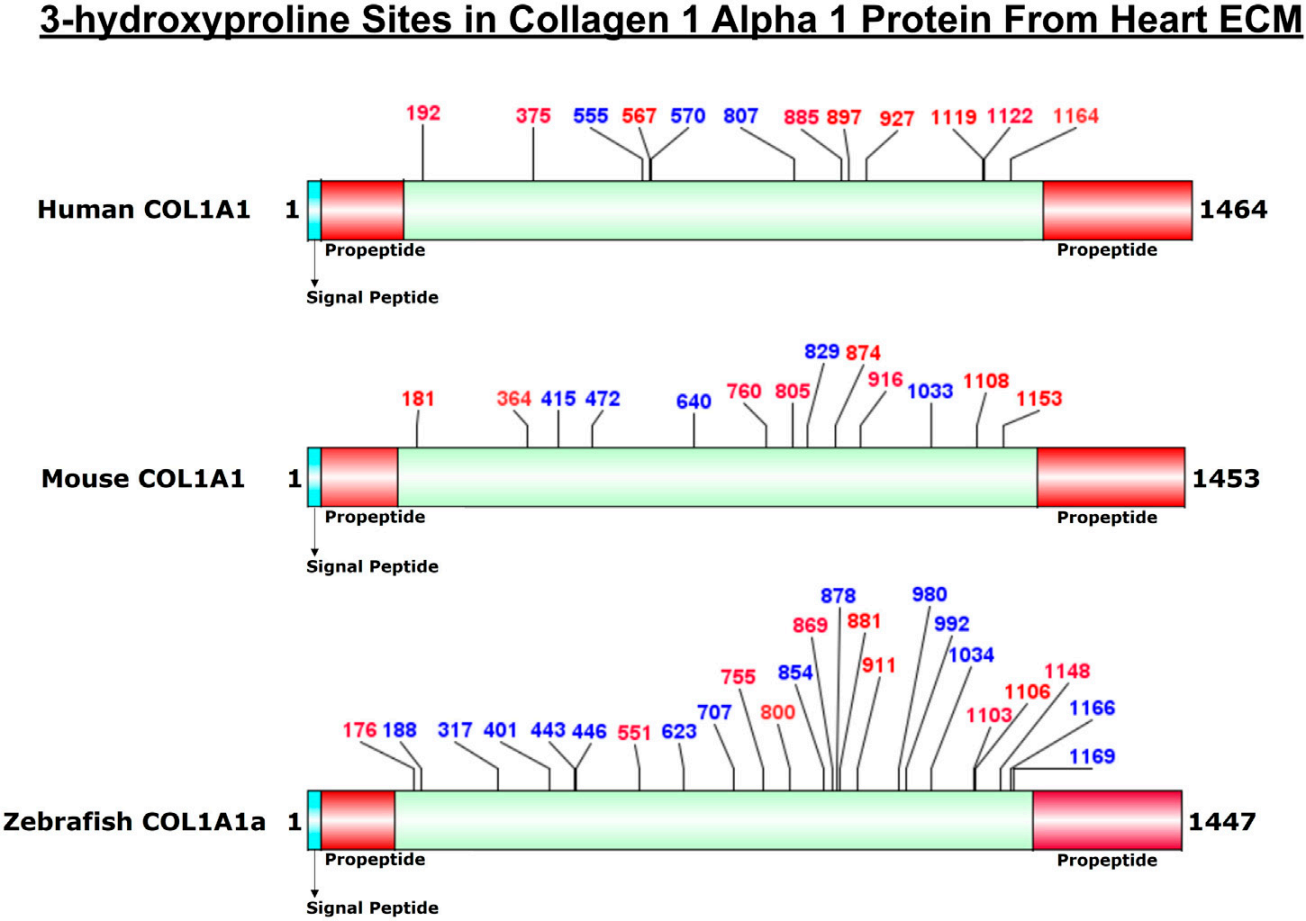
Comparison of 3-HyP sites identified in COL1A1a from zebrafish heart ECM to COL1A1 of human and mice heart ECM. The horizontal box represents the full-length COL1A1 sequence and the vertical black lines indicate the corresponding 3-HyP sites. The information for 3-HyP sites of human and mice heart ECM were re-analyzed in this manuscript from the available raw MS data from Barallobre-Barreiro et al. and Padmanabhan et al. The 3-HyP sites marked with red represent the conserved sites among human, mouse, and zebrafish.

COL1A1a chain was found to be the highest abundant out of all the three chains of collagen 1 triple helix in ECM of zebrafish heart. This chain has an important role in the stability and proper functioning of zebrafish cardiac tissues (Gistelinck et al., 2016). It is a large 1,447 amino acid long collagen chain. For determining the N (23–146, FSP/QMS), and C terminal propeptides cleavage site (1,202–1,447, RA/DDAN), we performed cleavage site sequence alignment matching with human COL1A1, which has also been reported previously (Gistelinck et al., 2016). However, we also detected a peptide sequence from N terminal propeptide hinting toward the dynamics of collagen assembly in the ECM. PTM module enabled dual search engine-based database search yielded 94.98% sequence coverage (considering the processed chain as the full length) (Figure 3; Table 1, Supplementary Figure S2). A total of 92 4-HyP sites on the “Yaa” position of “Gly-Xaa-Yaa” motif were detected. This estimate is suggestive of about 95% 4-HyP occupancy in the Yaa positioned (in Gly-Xaa-Yaa motif) prolines corroborating the previous Edman sequencing analysis in COL1A1 from mammals (Fietzek and Kühn, 1975). Furthermore, we have also detected 41 hydroxyproline sites in the “Xaa” position of the “Gly-Xaa-Yaa” motif. This finding is similar (n = 37) to human COL1A1 (Merl-Pham et al., 2019). In addition, we analyzed the occurrence of prolyl-3-hydroxylation sites providing crucial structural equilibrium in the collagen molecules deposited in ECM and affecting interactions with other ECM proteins (Pokidysheva et al., 2014; Grafe et al., 2014; Montgomery et al., 2018). We assigned these HyP as 3-hydroxyproline (3-HyP) as determined by previous experimental inferences (Fietzek and Kühn, 1975). A total of twenty-four 3-hydroxyproline sites (see Table 1) at the “Xaa” position of the G-Xaa-HyP motif in COL1A1a were identified. Out of these, 10 sites (P^176^, P^551^, P^755^, P^800^, P^869^, P^881^, P^911^, P^1103^, P^1106^_,_ and P^1148^) are evolutionarily conserved in mice (Padmanabhan Iyer et al., 2016) and human heart tissue (Barallobre-Barreiro et al., 2021) (Figure 4; Table 1, Supplementary Figure S2 and Supplementary Figure S3). This highlights probable conserved functions of these sites in collagen molecule providing structural support and maintaining homeostatic ECM assembly. Interestingly, we report the identification of 14 novels (P^188^, P^317^, P^401^, P^443^, P^446^, P^623^, P^707^, P^854^, P^878^, P^980^, P^992^, P^1034^, P^1166^_,_ and P^1169^) 3-HyP sites in the COL1A1a from zebrafish heart ECM. In zebrafish heart ECM, COL1A1a harbors numerous unique 3-HyP sites close to the N-terminal propeptide cleavage sites (3-HyP^188,317^), spanning across the triple-helical region as well. Two clusters of “Gly-Pro-Pro” motif present close to C-terminal propeptide were found to be 3-hydroxylated (3-HyP^1103,1106^ and 3-HyP^1166,1169^). The presence of many new 3-HyP sites compared to human and mouse hearts is itself a unique finding in the zebrafish COL1A1a (Figure 4) protein structure. These unique prolyl-3-hydroxylation sites may contribute to ECM remodeling during heart regeneration and warrants further investigations.

#### 3.4.2 Comprehensive Site-specific Map of PTMs in COL1A1b From the Zebrafish Heart ECM–

**FIGURE 5:**
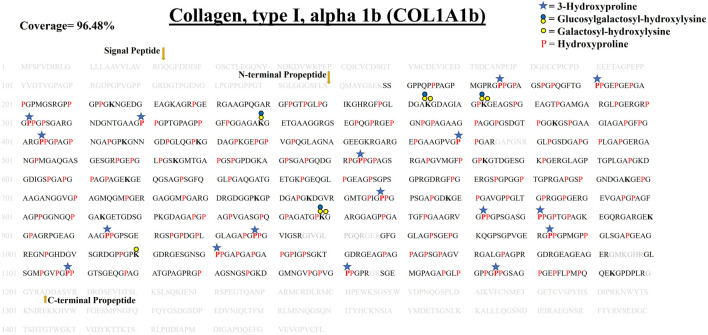
Comprehensive PTM map of COL1A1b of WT zebrafish heart ECM, presenting proline/lysine hydroxylation sites and lysine O-glycosylation sites. The representation of PTM sthe ites is similar to the COL1A1a PTM map as shown in top right corner. Peptides identified in proteomics analysis are shown in black color and unidentified peptides are shown in grey color. A total of 96.48% sequence coverage of COL1A1a is detected (considering the matured form of COL1A1b). The signal peptide is 22 amino acids (1–22) long. Sequence alignment matching and previous analysis by Gistelink et al. with human COL1A1 revealed the propeptide cleavage sites. Dark yellow arrows show N terminal (23–150) and C-Term (1,204–1,447) propeptide cleavage sites. Red bold “**P**” with a blue star represents 3-HyP and red “P” represents 4-HyP. Hydroxylysine is represented with bold “**K**” and yellow and blue circle represents the lysine O-glycosylation. A summary of these site-specific PTMs of COL1A1b is presented in Table 1, and all the PSMs for O-glycosylated lysine and 3-hydroxyproline sites are provided in Supplementary Figures. S2.22–S2.52.

In zebrafish cardiac ECM, the second chain contributing to the collagen 1 triple helix is COL1A1b. It is a 1,449 amino acid long chain. Our analysis revealed COL1A1b to be the next highest abundant collagen chain present in the zebrafish cardiac ECM. We identified and comprehensively mapped site-specific PTMs of COL1A1b. N terminal (150, FLS/QMA) and C terminal propeptide cleavage sites (1,204, YRA/DDA) were determined by sequence alignment, corroborating the previous report (Gistelinck et al., 2016). We detected 96.48% sequence coverage (considering the matured form) for COL1A1b. A total of 99 4-HyP sites on the “Yaa” position of “Gly-Xaa-Yaa” motif were detected out of the possible 103 sites. Similar to COL1A1a, we detected seventeen 3-HyP sites in COL1A1b on the ‘Xaa’ position of the Gly-Xaa-HyP motif out of the possible 27 sites. It seems that COL1A1b is less 3-hydroxylated at proline residues compared to COL1A1a. Furthermore, we have also detected 51 hydroxyproline sites in the “Xaa” position of “Gly-Xaa-Yaa” motif. As described previously, mass-spectrometry-based methods will not be able to resolve whether these prolyl-hydroxylations are occurring at the 3′ or 4′ position. However, recent studies have shown that these are most probably 4-prolyl-hydroxylations (Van Huizen et al., 2019). Surprisingly, we have detected a smaller number of lysine O-glycosylation sites in COL1A1b. A total of 19 hydroxylysines, 4 galactosyl-hydroxylysine, and 4 glucosylgalactosyl-hydroxylysine sites have been identified (Table 1). These comprehensive PTM maps of COL1A1b of zebrafish heart ECM are summarized in Figure 5 (the corresponding PSMs are summarized for each PTM site in Supplementary Figure S2).

#### 3.4.3 Comprehensive Site-specific Map of PTMs in COL1A2 From the Zebrafish Heart ECM–

**FIGURE 6:**
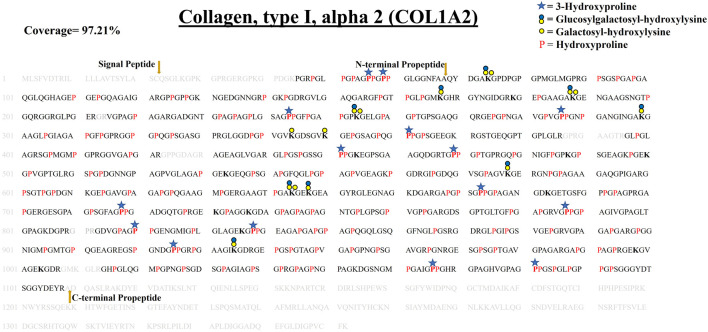
Comprehensive map of COL1A2 of ECM of WT zebrafish heart. It presents proline/lysine hydroxylation sites and lysine glycosylation sites. Representation of PTM sites is similar to COL1A1a and COL1A1b PTM maps. Peptides identified in proteomics analysis are shown in black color and unidentified peptides are shown in grey color. Total 97.21% sequence coverage of COL1A2 is detected (considering the matured form of COL1A1b). The signal peptide is 22 amino acids (1–22) long. N terminal propeptide (23–68) and C terminal propeptide (1,109–1,352) cleavage sites are marked with dark yellow arrows. Red bold “**P**” with blue star represents 3-HyP and red “P” represents 4-HyP. Hydroxylysine is represented with bold “**K**” and yellow and blue circle represents the lysine O-glycosylation. A summary of these site-specific PTMs of COL1A2 is presented in Table 1, and all the PSMs for O-glycosylated lysine and 3-hydroxyproline sites are provided in Supplementary Figure S2.53–S2.83.

COL1A2 is the third contributing collagen chain in the collagen 1 triple helix of zebrafish heart ECM. COL1A2 is a little smaller than the other 2 chains of collagen 1 triple helix. It consists of 1,352 amino acids. We performed a similar propeptide cleavage site sequence alignment match of human COL1A2 with zebrafish COL1A2 (Figure 6; Table 1, Supplementary Figure S2). We detected 97.21% sequence coverage of COL1A2 (considering the processed mature form). A total of 82 4-HyP sites on the ‘Yaa’ position of “Gly-Xaa-Yaa” motif were detected out of the possible 83 sites highlighting almost complete occupancy of 4-HyP possible sites. We identified 15 3-HyP sites in COL1A2 on “Xaa” position of the G-Xaa-HyP motif out of the possible 21 sites. Furthermore, we have also detected 60 hydroxyproline sites in the “Xaa” position of “Gly-Xaa-Yaa” motif. Along with proline modification, lysine modifications are also detected in COL1A2. A total of 21 hydroxylysine sites were detected in COL1A2. Hydroxylysines serve as the substrate for further O-glycosylation. A total of 6 galactosyl-hydroxylysine sites and 9 glucosylgalactosyl-hydroxylysine sites were detected in COL1A2. Figure 6, summarizes the first comprehensive PTM map of COL1A2.

### 3.5 Quantitation of Site-specific Prolyl-3-Hydroxylation Occupancy During Regeneration of Zebrafish Heart

**FIGURE 7:**
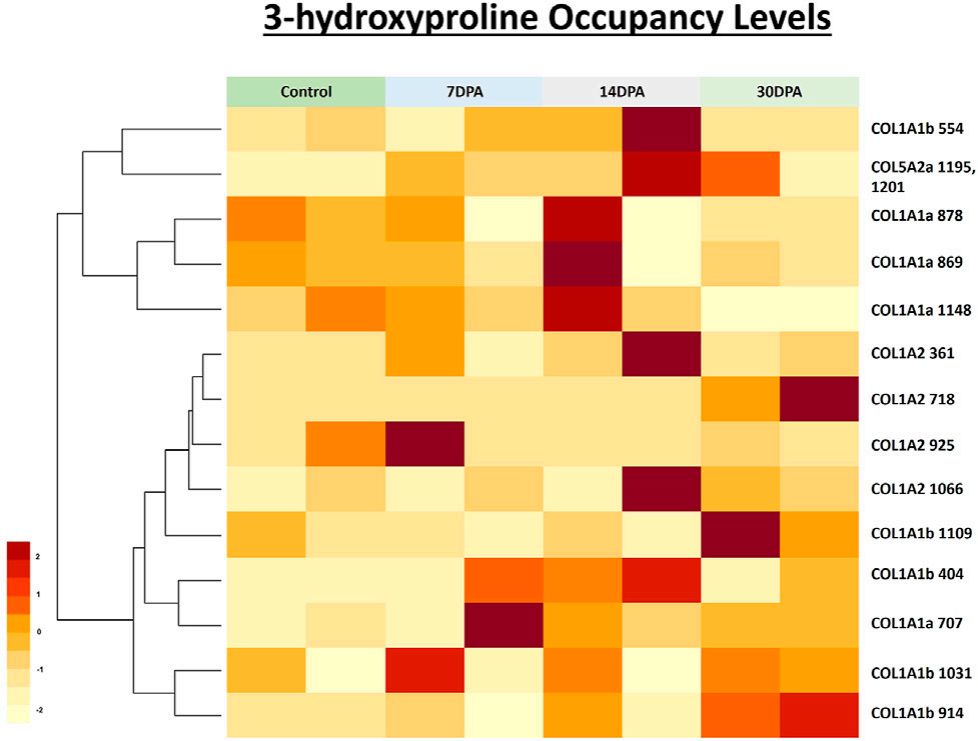
Heatmap depicting the relative occupancy level of 3-hydroxyproline sites in three different chains of collagen 1 deposited in zebrafish heart ECM during regeneration. Further occupancy of one 3-HyP cluster of COL5A2a^1195, 1201^ was also quantitated during zebrafish heart regeneration. Normalized occupancy values of prolyl-3-hydroxylations were computed to generate the heat map. Light yellow represents the low value (lowest occupancy) and dark red shows the higher value (highest occupancy) in the row.

Identification of prolyl-3-hydroxylation sites in collagen chains of heart ECM provided new insights into the tissue-specific variation of collagen molecules providing structural support in ECM. Latest studies have revealed that the absence of prolyl-3-hydroxylation in one site (P^1164^) of COL1A1 is associated with osteogenesis imperfecta. Mutation in the prolyl-hydroxylases can cause the formation of dysfunctional collagen (without a 3-HyP site) as found in osteogenesis imperfecta disease conditions (Cabral et al., 2007). Furthermore, Merl-Pham *et al*. recently showed that the occupancy of 3-HyP^771^ of human COL1A1 was increased during TGF-beta treated fibrotic conditions in primary human lung fibroblast (Merl-Pham et al., 2019). These indicate that changes in the occupancy of the 3-HyP level can have important consequences on the functionality of the collagen molecules present in the ECM. Therefore, we check the occupancy level of 3-HyP in collagens during the regeneration of the zebrafish heart. We utilized our proteomic pipeline and quantitated the occupancy levels of collagen PTMs during zebrafish heart regeneration in the sham model used as control, 7-, 14- and 30-day post-amputation. The critical prolyl-3-hydroxylation site (P^1164^) involved in osteogenesis imperfecta had been previously shown to be fully 3-hydroxylated in mouse skin fibroblast (Morello et al., 2006). Surprisingly, this conserved site present in zebrafish (3-HyP^1148^) control cardiac ECM is only about half (48%) 3-hydroxylated (Table 2; Figure 7). Furthermore, the occupancy of this site decreased to ∼7% in the 30 DPA regenerated zebrafish heart. The occupancy level of two other 3-HyP sites (P^869^, P^878^) of COL1A1a decreased at 30 DPA compared to control and 7 DPA occupancy (Table 2; Figure 7). Only 3-HyP^707^ occupancy level was increased (7.5–20%) at 30 DPA regenerated zebrafish heart compared to sham. This increased occupancy may favor the assembly of ECM by enhancing collagen fibril formation by forming a water bridge with the available free carbonyl group of the subsequent collagen chain. However, a decrease in occupancy of other 3-HyP sites may favor the reduced interaction of deposited collagen molecules in ECM thereby favoring the transition to regeneration from the fibrotic milieu. Although, the exact functional role of these site-specific 3-HyPs needs to be experimentally assessed. A total of five 3-hydroxylation sites (P^404,554,914,1031,1109^) occupancy levels were quantitated in COL1A1b. Out of these, 4 sites (P^404,914,1031,1109^) showed the trend of increased occupancy level at 30 DPA regenerated zebrafish heart. We quantitated the occupancy level of 4 sites in COL1A2. Most of these sites were having <10% prolyl-3-hydroxylation. Interestingly, we identified a cluster of 3-HyP sites (P^1195,1201^) in COL5A2a to be highly 3-hydroxylated (24%) after 7 DPA samples compared to only 1.7% occupancy in control. The occupancy of this 3-HyP cluster showed dynamic changes at 14 DPA (55%) and further reverting to 30% at 30 DPA, in the regenerated zebrafish heart. The site-specific variation in the occupancy level of 3-hydroxyproline could be due to varied gene expression of specific enzymes expressed in the zebrafish heart during post-amputation regeneration. A recent report from Sanchez-Iranzo et al. showed the expression of prolyl-3-hydroxylase-3 (P3H3) and prolyl-3-hydroxylase-4 (P3H4) decreased at 7 days post cryoinjury (log fold change -0.4,-0.2, respectively), in the zebrafish heart. However, in the same RNA seq analysis, the expression level of prolyl-3-hydroxylase-1 (P3H1) and prolyl-3-hydroxylase-2 (P3H2) were found to be increased (log fold change1.41,1.75 respectively) in the zebrafish heart at 7 days post-cryoinjury model. Following the regeneration in the same model till 60 days post-cryoinjury, the expression level of P3H2 and P3H4 increased (log fold change 0.94, 1.73 respectively) and the expression of P3H1 and P3H3 decreased (log fold change −0.56, −1.13) respectively highlighting the dynamic changes in the expression of these isoforms of prolyl-3-hydroxylases. However, the enzyme-substrate specificity for these different isoforms of prolyl-3-hydroxylases is not known yet. Thus, ascertaining a direct correlation between the expression level of one enzyme isoform with the substrate (occupancy level of site-specific modifications) specificity in this dataset is difficult. But, the varied expression of these enzymes during the post-cryoinjury regeneration model in zebrafish heart further supports the specific regulation of site-specific dynamic prolyl-3-hydroxylation changes in collagens deposited in the zebrafish heart ECM during regeneration. The dynamics of prolyl-3-hydroxylation occupancy in collagen molecules deposited in ECM during zebrafish heart regeneration are reported here for the first time.

**TABLE 2:** Quantitative occupancy (%) of 3-hydroxyproline (3-HyP) sites identified in three different chains of collagen I (COL1A1a, COL1A1b, and COL1A2) from zebrafish cardiac ECM. Results are expressed as mean ± S.D. Significant statistical differences were estimated by ANOVA (*p < 0.05, ^ns^p > 0.05).

3-HyP site	3-Hydroxyproline average occupancy %			
	Sham average	7 DPA Average	14 DPA Average	30 DPA Average
COL1A1a P^707^	7.55 ± 4.3	31.49 ± 38.1	21.21 ± 7.8	20.50 ± 1.5 ^ns^
COL1A1a P^869^	35.37 ± 6.9	22.82 ± 13.3	48.10 ± 63.8	20.12 ± 2.9 ^ns^
COL1A1a P^878^	28.87 ± 7.2	17.57 ± 18.4	27.63 ± 35.8	12.76 ± 1.8 ^ns^
COL1A1a P^1148^	48.73 ± 14.7	44.03 ± 17.0	68.96 ± 41.1	7.23 ± 4.6^ns^
COL5A2a P^1195, 1201^	1.77 ± 1.3	23.97 ± 4.5	55.24 ± 47.4	30.04 ± 41.1 ^ns^
COL1A2 P^718^	0.19 ± 0.1	0.20 ± 0.3	0.10 ± 0.1	10.54 ± 7.4 ^ns^
COL1A2 P^925^	5.14 ± 5.7	10.00 ± 13.2	0.53 ± 0.7	1.86 ± 1.8 ^ns^
COL1A2 P^1066^	3.92 ± 3.4	3.87 ± 3.1	12.95 ± 15.7	6.91 ± 2.0 ^ns^
COL1A2 P^361^	0.96 ± 0.3	1.52 ± 1.6	4.66 ± 4.5	1.18 ± 0.4 ^ns^
COL1A1b P^404^	7.88 ± 1.9	18.28 ± 12.8	30.08 ± 6.7	11.95 ± 7.1 ^ns^
COL1A1b P^554^	47.73 ± 4.0	46.52 ± 14.8	76.14 ± 29.7	42.91 ± 1.3 ^ns^
COL1A1b P^914^	17.09 ± 1.4	16.74 ± 10.6	21.16 ± 14.0	47.35 ± 6.2 ^ns^
COL1A1b P^1031^	12.44 ± 16.1	32.42 ± 34.0	18.31 ± 24.2	33.45 ± 3.6 ^ns^
COL1A1b P^1109^	10.36 ± 7.6	3.93 ± 1.7	4.76 ± 6.0	32.64 ± 18.6 ^ns^

### 3.6 Comprehensive Mapping and Microheterogeneity of Collagen 1 Lysine O-Glycosylation Sites During Regeneration of Zebrafish Heart

In collagens, lysine hydroxylation and glycosylation play important role in collagen assembly and fibril formation (Terajima et al., 2014; Terajima et al., 2016). Musculoskeletal defects and cerebral small vessel disease are caused due to lack of collagen glycosylation (Geister et al., 2019; Miyatake et al., 2018; Kivirikko et al., 1973). A total of 23 HyK, 9 G-HyK, and 11 GG-HyK sites in zebrafish COL1A1a were identified from wild-type (Sham) zebrafish heart ECM (Figure 3). Classical amino acid analysis has revealed that fibrillar collagens are less glycosylated compared to basement membrane collagen IV (Terajima et al., 2014; Perdivara et al., 2013; Yamauchi and Sricholpech, 2012). However, collagen glycosylation in fibrillar collagen is important for crosslinking mediated fibrillar assembly. Microheterogeneity of lysine sites has been determined in collagens chains (Yamauchi et al., 1982). A single lysine site of a collagen chain could be present in the form of unmodified, hydroxylysine (HyK), galactosyl-hydroxylysine (G-HyK), and glucosyl-galactosyl-hydroxylysine (GG-HyK). This microheterogeneity was found to be highly dynamic in the COL1A1a chain during zebrafish heart regeneration. We detected microheterogeneity on a total of eight lysine sites in the COL1A1a chain. Figure 5 and Figure 6 respectively show the site-specific mapping of lysine O-glycosylation sites in COL1A1b and COL1A2 chains from wild-type zebrafish heart ECM. In COL1A1b a total of 24 HyK, 4 G-HyK (K^264^, K^273^, K^849^, K^1020^), and 4 GG-HyK (K^264^, K^273^, K^399^, K^849^) sites were identified. Three of these sites showed microheterogeneity in glycosylation patterns. Similarly, a total of 21 HyK, 6 G-HyK (K^74^, K^188^, K^254^, K^344^, K^350^, K^644^), and 9 GG-HyK (K^74^, K^167^, K^188^, K^254^, K^299^, K^578^, K^644^, K^647^, K^935^) sites were identified in COL1A2 chain for the first time from zebrafish heart ECM. Four of these sites showed microheterogeneity in different glycosylation moieties (galactosyl or glucosyl-galactosyl). Overall, our analysis mapped the different lysine O-glycosylation sites in three different chains of collagen 1 chains. Collagen 1A1a was found to be less deposited during the regeneration of the zebrafish heart by our as well as Garcia-Puig et al. analysis (Garcia-Puig et al., 2019). Furthermore, we wanted to assess the changes in O-glycosylation of collagen I chains during zebrafish heart regeneration.

**FIGURE 8:**
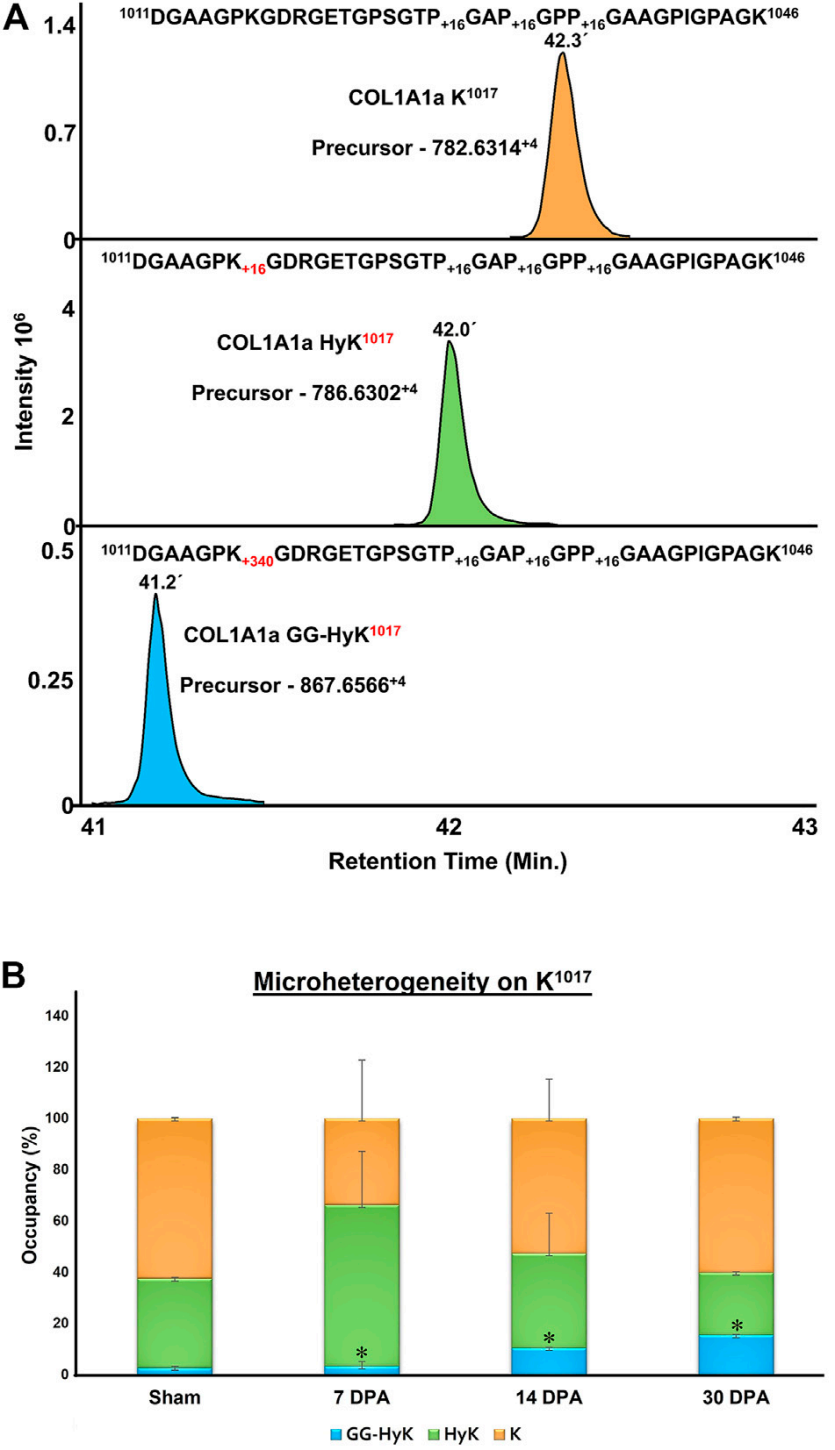
Quantitation of microheterogeneity of K^1017^ site in COL1A1a present in zebrafish heart ECM during regeneration. **(A)** Chromatogram plots represent the elution of unmodified **(K)**, hydroxylysine (HyK), and glucosylgalactosyl-hydroxylysine (GG-HyK) modified K^1017^ site containing peptide ^1011^DGAAGP**K**GDRGETGPSGTPGAPGPPGAAGPIGPAGK^1046^ **(B)** Graphical representation of Skyline-based MS^1^ quantitation of micro-heterogenic distribution of unmodified K^1017^ (yellow), HyK^1017^ (green) and GG-HyK277 (blue) species in COL1A1a from the ECM digest of control, 7 DPA, 14 DPA, and 30 DPA regenerating zebrafish heart. The different colors in the bar represent the occupancy of different forms at the K^1017^ site in COL1A1a with mean ± SEM. An increase in glucosylgalactosyl-hydroxylysine levels during regeneration is significant (ANOVA, *p* < 0.05) (See Table 3)

In order to assess the changes in the microheterogeneity of lysine O-glycosylation sites on collagen I during regeneration of zebrafish heart, the Skyline-based targeted MS^1^ quantitative method was integrated into our pipeline as described previously (Merl-Pham et al., 2019). The relative microheterogeneity analysis of site-specific lysine in three different chains of collagen I was performed. A total of 12 sites of lysine microheterogeneity from three different chains of collagen I was assessed (Table 3). Figure 8 represents an example of a COL1A1a peptide ^1011^DGAAGPKGDRGETGPSGTPGAPGPPGAAGPIGPAGK^1046^ eluting at 42.3 min during the chromatographic separation as non-glycosylated form with three 4-HyP (P^1029,1032,1035^) sites. Hydroxylation of K^1017^ resulted in an early elution of this peptide at ∼42 min in the C18 column-based separation. Further, the glucosyl-galactosyl-hydroxylysine form of K^1017^ was further eluted earlier (at 41.2 min) than the hydroxylation form. We determined the microheterogeneity of this site (K^1017^) across control, 7-day post-amputation (DPA), 14 DPA, and 30 DPA samples across zebrafish heart regeneration. Interestingly, the glucosyl-galactosyl-hydroxylation of K^1017^ increased significantly (*p* < 0.01) to 15.4% in the 30 DPA regenerated heart as compared to control (2.74%), (3.35%) 7 DPA. and 14 DPA (10.5%) samples (Figure 8; Table 3). The increase in the micro heterogenic distribution of O-glycosylation the in K^1017^ site was evident by a decrease in the HyK level during regeneration (Figure 8; Table 3). Using a similar strategy, we found K^846^ (COL1A1a) to be specifically present in a full glycosylated form (either G-HyK or GG-HyK). The GG-HyK^846^ was decreased at 30 DPA regenerated zebrafish heart. We also quantitated the microheterogeneity of three lysine sites (K^264,273,849^) in COL1A1b and 2 sites in COL1A2 (K^254,644^) (see Table 3). The micro-heterogenic distribution of G-HyK^264^ of COL1A1b was found to have an increasing trend at 30 DPA. The changes in the level of hydroxylation and O-glycosylation of lysine sites present in collagen I could be potentially due to the gene expression changes of lysyl-hydroxylases and glycosyltransferases during zebrafish heart regeneration. In a similar model of zebrafish heart regeneration, proteomic analysis performed by Ma *et al*. (2018) revealed a significant elevation of LH2 levels at 2 DPA (Supplementary Figure S4). However, at 14 DPA the LH2 protein level stabilizes to sham levels. Similar oscillatory gene expressional observations of the genes (PLOD1a, PLOD2, and PLOD3) coding for different isoforms of lysyl-hydroxylases has been documented in the zebrafish heart cryoinjury model (Sánchez-Iranzo et al., 2018). Global gene expression analysis revealed increased expression of these three genes in the zebrafish heart at 7 days post-injury. However, the level of gene expression of PLOD1a, PLOD2, and PLOD3 decreases at 60 days post injury in the regenerated heart (Sánchez-Iranzo et al., 2018). These micro-heterogenic site-specific glycosylation profiles point towards the differences present in three different chains of collagen I that may play an important role in the ECM remodeling mediated zebrafish heart regeneration.

**TABLE 3:** Quantitative micro-heterogenic occupancy (%) of O-glycosylated lysine sites identified three different chains of collagen I (COL1A1a, COL1A1b, and COL1A2) from zebrafish cardiac ECM. Results are expressed as mean ± SEM. Statistically significant differences were estimated by ANOVA (*p < 0.05, ^ns^ > 0.05). N/D, not detected; HyK, hydroxylysine; G-HyK, galactosyl-hydroxylysine; GG-HyK, glucosylgalactosylhydroxylysine.

		Microheterogeneity %			
Lysine sites	Modification	Sham	7 DPA	14 DPA	30 DPA
COL1A1a K^261^	K	36.62 ± 9.7	43.75 ± 4.38	46.09 ± 4.89	32.30 ± 5.08 ^ns^
	HyK	38.38 ± 10.38	34.81 ± 12.3	21.13 ± 2.85	45.72 ± 8.65 ^ns^
	G-HyK	16.71 ± 4.9	17.85 ± 12.0	31.26 ± 3.01	20.22 ± 3.01 ^ns^
	GG-HyK	8.28 ± 6.5	3.59 ± 4.3	1.32 ± 0.97	2.35 ± 0.57 ^ns^
COL1A1a K^270^	K	50.46 ± 5.3	32.92 ± 4.4	26.58 ± 1.7	46.48 ± 4.1 ^ns^
	HyK	34.97 ± 0.7	37.13 ± 1.3	46.12 ± 1.1	40.69 ± 4.4 ^ns^
	G-HyK	12.26 ± 5.0	29.03 ± 4.4	24.56 ± 2.7	11.52 ± 0.4 ^ns^
	GG-HyK	2.32 ± 1.1	0.93 ± 1.3	2.74 ± 0.2	1.30 ± 0.7 ^ns^
COL1A1a K^504^	K	35.12 ± 0.2	6.54 ± 0.3	34.96 ± 46.9	51.61 ± 20.2 ^ns^
	HyK	7.20 ± 4.4	3.49 ± 0.3	16.03 ± 22.4	16.85 ± 5.2 ^ns^
	G-HyK	57.68 ± 4.2	89.97 ± 0.0	49.01 ± 69.3	31.54 ± 15.0 ^ns^
	GG-HyK	ND	ND	ND	ND
COL1A1a K^570^	K	59.52 ± 12.5	24.79 ± 15.4	17.67 ± 1.6	42.84 ± 51.5 ^ns^
	HyK	14.06 ± 4.6	6.83 ± 7.6	4.50 ± 0.1	0.21 ± 0.3 ^ns^
	G-HyK	ND	ND	ND	ND
	GG-HyK	26.42 ± 17.1	68.39 ± 7.8	77.83 ± 1.6	56.95 ± 51.8 ^ns^
COL1A1a K^693^	K	16.05 ± 1.8	11.35 ± 1.4	21.14 ± 15.4	17.46 ± 2.3 ^ns^
	HyK	73.86 ± 2.2	83.40 ± 0.1	70.60 ± 23.8	74.25 ± 1.7 ^ns^
	GK	1.34 ± 0.3	0.25 ± 0.4	0.56 ± 0.8	1.47 ± 0.2 ^ns^
	GGK	8.75 ± 0.4	5.00 ± 1.0	7.69 ± 9.2	6.82 ± 3.8 ^ns^
COL1A1a K^846^	K	ND	ND	ND	ND
	HyK	ND	ND	ND	ND
	G-HyK	48.69 ± 29.7	85.04 ± 19.4	51.02 ± 9.0	68.87 ± 5.5 ^ns^
	GG-HyK	51.31 ± 29.7	14.96 ± 19.4	48.98 ± 9.0	31.13 ± 5.5 ^ns^
COL1A1a K^1017^	K	62.44 ± 0.3	33.69 ± 22.8	52.55 ± 21.8	60.09 ± 0.5 ^ns^
	HyK	34.82 ± 0.4	62.96 ± 20.9	36.95 ± 22.1	24.44 ± 0.2 ^ns^
	G-HyK	ND	ND	ND	ND
	GG-HyK	2.74 ± 0.7	3.35 ± 1.9	10.50 ± 0.3	15.47 ± 0.3 *
COL1A1b K^264^	K	50.52 ± 15.5	40.63 ± 50.7	52.94 ± 15.8	20.39 ± 20.2 ^ns^
	HyK	41.23 ± 8.2	53.16 ± 56.0	30.76 ± 34.6	46.65 ± 44.7 ^ns^
	G-HyK	5.19 ± 4.4	4.64 ± 3.6	9.75 ± 10.8	27.51 ± 20.7 ^ns^
	GG-HyK	3.05 ± 2.9	1.57 ± 1.6	6.54 ± 7.9	5.45 ± 3.7 ^ns^
COL1A1b K^273^	K	ND	ND	ND	ND
	HyK	41.65 ± 47.5	50.93 ± 39.7	24.43 ± 25.6	33.90 ± 36.0 ^ns^
	G-HyK	5.58 ± 4.6	33.38 ± 20.8	27.55 ± 13.3	19.99 ± 10.5 ^ns^
	GG-HyK	52.77 ± 42.9	15.69 ± 19.0	48.02 ± 38.9	46.11 ± 25.5 ^ns^
COL1A1b K^849^	K	27.06 ± 1.1	10.35 ± 13.7	11.17 ± 2.2	22.95 ± 3.4 ^ns^
	HyK	67.46 ± 1.1	68.53 ± 16.8	70.77 ± 9.7	62.89 ± 0.3 ^ns^
	G-HyK	3.35 ± 0.4	6.76 ± 9.4	12.97 ± 4.5	9.48 ± 1.5 ^ns^
	GG-HyK	2.13 ± 0.4	14.36 ± 12.4	5.09 ± 2.9	4.68 ± 2.2 ^ns^
COL1A2 K^254^	K	6.48 ± 3.2	20.75 ± 14.6	21.61 ± 14.3	55.69 ± 18.4 ^ns^
	HyK	89.24 ± 1.6	78.24 ± 13.4	69.76 ± 2.2	43.90 ± 18.1 ^ns^
	G-HyK	0.11 ± 0.0	0.10 ± 0.1	0.22 ± 0.3	0.05 ± 0.0 ^ns^
	GG-HyK	4.17 ± 4.8	0.91 ± 1.1	8.40 ± 11.7	0.36 ± 0.3 ^ns^
COL1A2 K^644^	K	ND	ND	ND	ND
	HyK	84.59 ± 1.8	61.15 ± 40.2	95.15 ± 6.5	90.77 ± 11.1 ^ns^
	G-HyK	ND	ND	ND	ND
	GGK	15.41 ± 1.8	38.85 ± 40.2	4.85 ± 6.5	9.23 ± 11.1 ^ns^

## 4 Conclusion

In conclusion, our analysis presents the first comprehensive mapping and dynamics of site-specific collagen PTMs from the crude extracellular matrix proteins in wild-type and regenerating zebrafish hearts. We optimized an in-house dual database search engine-based mass-spectrometry data analysis strategy to efficiently identify long post-translationally modified collagen peptides from crude ECM analysis. We identified the highest number of collagen chains (36) present in the zebrafish heart ECM. Our analysis revealed that the expression of collagens gets altered during regeneration. Global characterization of zebrafish heart ECM revealed the identification of site-specific PTMs (proline hydroxylation, lysine hydroxylation, and lysine glycosylation sites) in 23 different collagen chains. These are the first comprehensive site-specific collagen PTM maps of zebrafish cardiac ECM highlighting some novel sites. Further, we present the first comprehensive PTM maps of three chains (COL1A1a, COL1A1b, and COL1A2) of collagen 1. Our analysis also identified the conserved 3-hydroxyproline sites occurring in the COL1A1 chain in humans, mice, and zebrafish heart ECM. This finding highlights the importance of these conserved 3-HyP sites involved in the basic function of collagens in humans, mice, and zebrafish myocardium. Moreover, we established that site-specific PTM (Prolyl-3-hydroxylations and lysyl O-glycosylation) changes in collagen chains are correlated with the regeneration process. Glycosylation on one site (GG-HyK^1017^) of COL1A1a is found to be significantly increased during the regeneration process. Taken together, our analysis will widen new avenues to dig deeper in understanding the role of collagen PTMs during zebrafish heart regeneration.

## Data Availability

Publicly available datasets were analyzed in this study. The datasets PXD011627 and PXD010092 can be accessed at: https://www.proteomexchange.org/.
