# Supplementary material for: Comprehensive Mapping and Dynamics of Site-Specific Prolyl-Hydroxylation, Lysyl-Hydroxylation and Lysyl O-Glycosylation of Collagens Deposited in ECM During Zebrafish Heart Regeneration
Source: Front Mol Biosci. 2022 Jun 16;9:892763. doi: 10.3389/fmolb.2022.892763 (PMC9245515; doi:10.3389/fmolb.2022.892763)

Supplementary material for Comprehensive mapping and dynamics of site-specific prolyl-hydroxylation, lysyl-hydroxylation and lysyl O-glycosylation of collagens deposited in ECM during zebrafish heart regeneration

Vivek Sarohi[1,2], Shriya Srivastava[1], Trayambak Basak[1,2*]

[1] School of Basic Sciences (SBS), Indian Institute of Technology (IIT)- Mandi

[2] BioX Center, IIT-Mandi, Himachal Pradesh, India-175075

*** Correspondence:**
Trayambak Basak, Ph.D.

SBS, IIT-Mandi,
Himachal Pradesh,
India-175075
Email- trayambak@iitmandi.ac.in
Ph- (+91)1905-267826
Lab page- Proteomics Lab@IIT-Mandi

Table of Contents

S.N.	Title	Page No.
1	Supplementary Table 1	2-3
2	Supplementary Table 2	4
3	Supplementary Table 3	5
4	Supplementary Figure 1	5-6
5	Supplementary Figure 2	6-89
6	Supplementary Figure 3	90-113
7	Supplementary Figure 4	114

Supplementary Tables

Table 1- Comparison of number of collagens identified in published data and re-analysis.

**Anna et. al.**		**Sarohi et. al.**					
**ECM Decellurization Protocol Dataset**	**Regeneration Dataset**	**ECM Decellurization Dataset**	**Regeneration Dataset**	**Control**	**7DPA**	**14DPA**	**30DPA**
col12a1a	col10a1a	COL1A1a	COL1A1a	**YES**	**YES**	**YES**	**YES**
col18a1b	col10a1b	COL6A3	COL1A2	**YES**	**YES**	**YES**	**YES**
col1a1a	col11a1b	COL1A1b	COL1A1b	**YES**	**YES**	**YES**	**YES**
col1a1b	col12a1a	COL1A2	COL5A2a	**YES**	**YES**	**YES**	**YES**
col1a2	col12a1b	COL6A1	COL5A1	**YES**	**YES**	**YES**	**YES**
col4a1	col15a1	COL6A4a	COL6A3	**YES**	**YES**	**YES**	**YES**
col4a2	col1a1a	COL6A2	COL4A2	**YES**	**YES**	**YES**	**YES**
col5a1	col1a1b	COL5A1	COL6A1	**YES**	**YES**	**YES**	**YES**
col5a2a	col1a2	COL4A2	COL4A1	**YES**	**YES**	**YES**	**YES**
col6a1	col21a1	COL5A2a	COL6A2	**YES**	**YES**	**YES**	**YES**
col6a2	col26a1	COL4A1	COL6A4a	**YES**	**YES**	**YES**	**YES**
col6a3	col4a1	COL2A1b	COL2A1a	**YES**	**YES**	**YES**	**YES**
col6a3	col4a2	COL11A1a	COL2A1b	**YES**	**YES**	**YES**	**YES**
col6a4a	col5a1	COL12A1a	COL16A1	**YES**	**YES**	**YES**	**YES**
	col5a2a	COL18A1b	COL11A1a	**YES**	**YES**	**YES**	**YES**
	col6a1	COL5A2b	COL4A5	**YES**	**YES**	**YES**	**YES**

	col6a2		COL11A1b	**YES**	**YES**	**YES**	**YES**
	col6a3		COL12A1a	**YES**	**YES**	**YES**	**YES**
	col6a6		COL11A2	**YES**	**YES**	**YES**	**YES**
			COL7A1	**YES**	**YES**	**YES**	**YES**
			COL5A2b	**YES**	**YES**	**NO**	**NO**
			COL5A3b	**YES**	**NO**	**YES**	**NO**
			COL17A1a	**YES**	**NO**	**NO**	**NO**
			COL22A1	**YES**	**NO**	**YES**	**NO**
			COL10A1b	**NO**	**YES**	**YES**	**NO**
			COL10A1a	**N0**	**YES**	**NO**	**NO**
			COL12A1b	**YES**	**YES**	**YES**	**YES**
			COL15A1a	**NO**	**YES**	**YES**	**YES**
			COL27A1b	**NO**	**YES**	**NO**	**NO**
			COL28a2b	**NO**	**YES**	**YES**	**NO**
			COL5A3a	**NO**	**NO**	**YES**	**NO**
			COL27A1a	**NO**	**NO**	**NO**	**YES**
			COL28A2a	**NO**	**YES**	**NO**	**NO**
			COL15A1b	**NO**	**YES**	**YES**	**YES**
			COL17A1b	**NO**	**YES**	**NO**	**NO**
			COL18A1b	**YES**	**NO**	NO	NO

Table 2a- Contribution of hydroxyproline containing peptides in identification of more number of collagen chains in database search in MyriMatch.

File	Total Peptides	Peptides with HyP
MyriMatch Control_1	3554	2269
MyriMatch Control_2	3122	2145
MyriMatch 7DPA_1	2810	1309
MyriMatch 7DPA_2	2137	1456
MyriMatch 14DPA_1	2102	1073
MyriMatch 14DPA_2	1901	1233
MyriMatch 30DPA_1	1995	1275
MyriMatch 30DPA_2	1835	1132
**Total**	**19456**	**11892**

Table 2b- Contribution of hydroxyproline in identification of collagens in database search in MSFragger.

File	Total Peptides	Peptides with HyP
FragPipe Control_1	2696	1334
FragPipe Control_2	2638	1304
FragPipe 7DPA_1	2590	697
FragPipe 7DPA_2	2233	1004
FragPipe 14DPA_1	1967	701
FragPipe 14DPA_2	1856	719
FragPipe 30DPA_1	2473	938
FragPipe 30DPA_2	1821	697
**Total**	**18274**	**7394**

Table 3. Normalized spectral counts from MyriMatch database search shows the relative abundance of collagens during regeneration.

Genes	Control_1	Control_2	7DPA_1	7DPA_2	14DPA_1	14DPA_2	30DPA_1	30DPA_2
COL1A1a	1441.06	1513.02	694.06	1326.08	851.60	1235.44	1289.54	1298.98
COL1A1b	933.76	1059.52	460.05	1007.66	781.99	1315.14	1006.41	1027.91
COL1A2	990.68	960.99	408.68	816.20	522.81	1226.24	893.61	955.45
COL5A1	214.47	195.71	81.05	118.90	162.91	165.54	137.14	169.08
COL5A2a	168.28	186.26	100.46	131.00	109.60	134.89	145.99	187.87
COL6A3	146.00	78.28	93.61	104.80	191.05	208.46	192.44	249.60
COL4A1	101.46	97.18	43.38	38.29	59.24	82.77	30.97	64.41
COL6A1	93.21	82.33	58.22	50.38	68.13	58.25	30.97	53.68
COL4A2	56.92	56.69	30.82	38.29	47.39	64.38	53.09	45.63
COL6A2	47.84	56.69	55.94	50.38	136.26	58.25	92.90	85.88
COL6A4a	21.45	20.25	37.67	22.17	81.46	49.05	53.09	61.73
COL16A1	12.37	16.20	5.71	6.05	4.44	9.20	11.06	0.00
COL2A1a	0.00	18.90	0.00	20.15	0.00	0.00	0.00	0.00
COL2A1b	0.00	18.90	17.12	26.20	0.00	42.92	8.85	18.79
COL4A5	6.60	5.40	4.57	0.00	0.00	0.00	0.00	13.42
COL11A1b	3.30	8.10	11.42	34.26	20.73	24.52	15.48	18.79
COL11A1a	3.30	6.75	10.27	16.12	0.00	27.59	0.00	8.05
COL12A1a	1.65	2.70	14.84	18.14	8.89	9.20	8.85	8.05
COL12A1b	1.65	0.00	6.85	4.03	0.00	0.00	0.00	0.00
COL10A1b	0.00	0.00	10.27	24.18	0.00	6.13	0.00	0.00
COL15A1a	0.00	0.00	7.99	8.06	10.37	12.26	0.00	0.00
COL10A1a	0.00	0.00	7.99	18.14	0.00	0.00	0.00	0.00
COL5A3b	0.00	0.00	0.00	0.00	5.92	15.33	0.00	0.00
COL28A2a	0.00	0.00	2.28	4.03	0.00	0.00	0.00	0.00

Supplementary Figures- Supplementary Figure 1 . Figure shows the relative abundance of 10 most abundant collagens in zebrafish heart ECM during regeneration quantitated by using normalized spectral counts of database search of MSFragger and MyriMatch. (A) Abundance of 10 abundant collagens in zebrafish heart ECM at 7 day post amputation. (B) Abundance of top 10 collagens in zebrafish heart ECM at 14 day post amputation. (C) Abundance of 10 abundant collagen in zebrafish heart ECM at 30 day post amputation. Collagen 1 triple helix was found to be most abundant in zebrafish heart ECM at 7, 14 and 30 day post amputation.

A

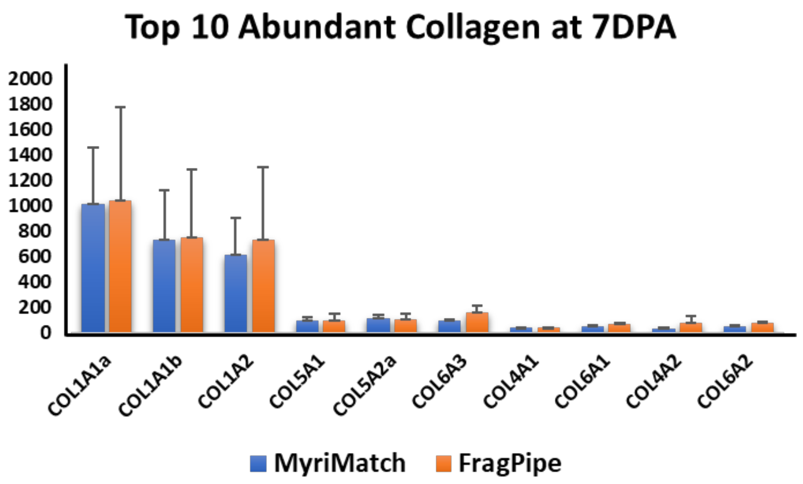
Top 10 Abundant Collagen at 7DPA
2000
1800
1600
1400
1200
1000
800
600
400
200
0
COL1A1a
COL1A1b
COL1A2
COL5A1
COL5A2a
COL6A3
COL4A1
COL6A1
COL4A2
COL6A2
MyriMatch
FragPipe

B

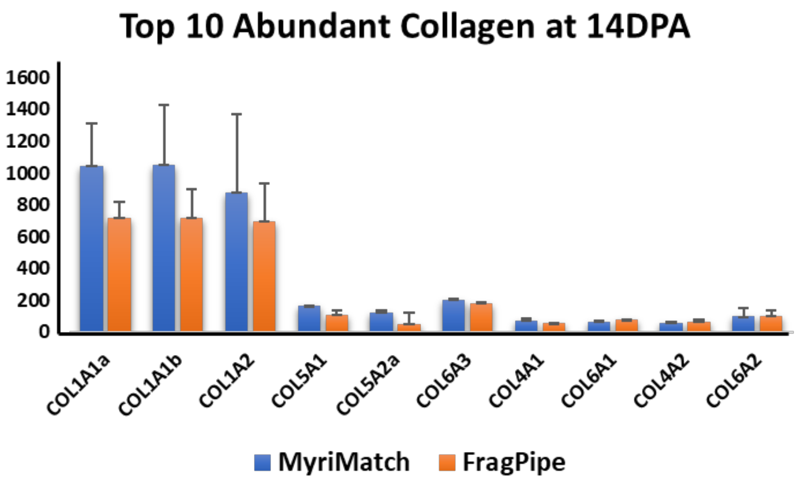
Top 10 Abundant Collagen at 14DPA
1600
1400
1200
1000
800
600
400
200
0
COL1A1a
COL1A1b
COL1A2
COL5A1
COL5A2a
COL6A3
COL4A1
COL6A1
COL4A2
COL6A2
MyriMatch
FragPipe

C

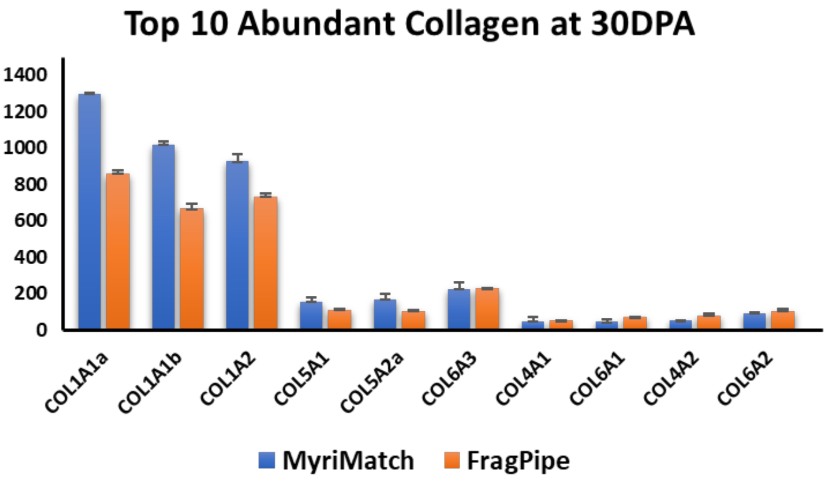
Top 10 Abundant Collagen at 30DPA
1400
1200
1000
800
600
400
200
0
COL1A1a
COL1A1b
COL1A2
COL5A1
COL5A2a
COL6A3
COL4A1
COL6A1
COL4A2
COL6A2
MyriMatch
FragPipe

Supplementary Figure 2: Peptide spectral matches (PSM) for 3-hydroprolines, hydroxylysines, galactosyl-hydroxylysines and glucosylgalactosyl-hydroxylysines detected in 3 chains of collagen 1 (COL1A1a, COL1A1b and COL1A2) in zebrafish heart ECM. PSMs with <20 ppm fragment mass tolerance were included in the analysis. PSMs were annotated using pLABEL.

S2.1

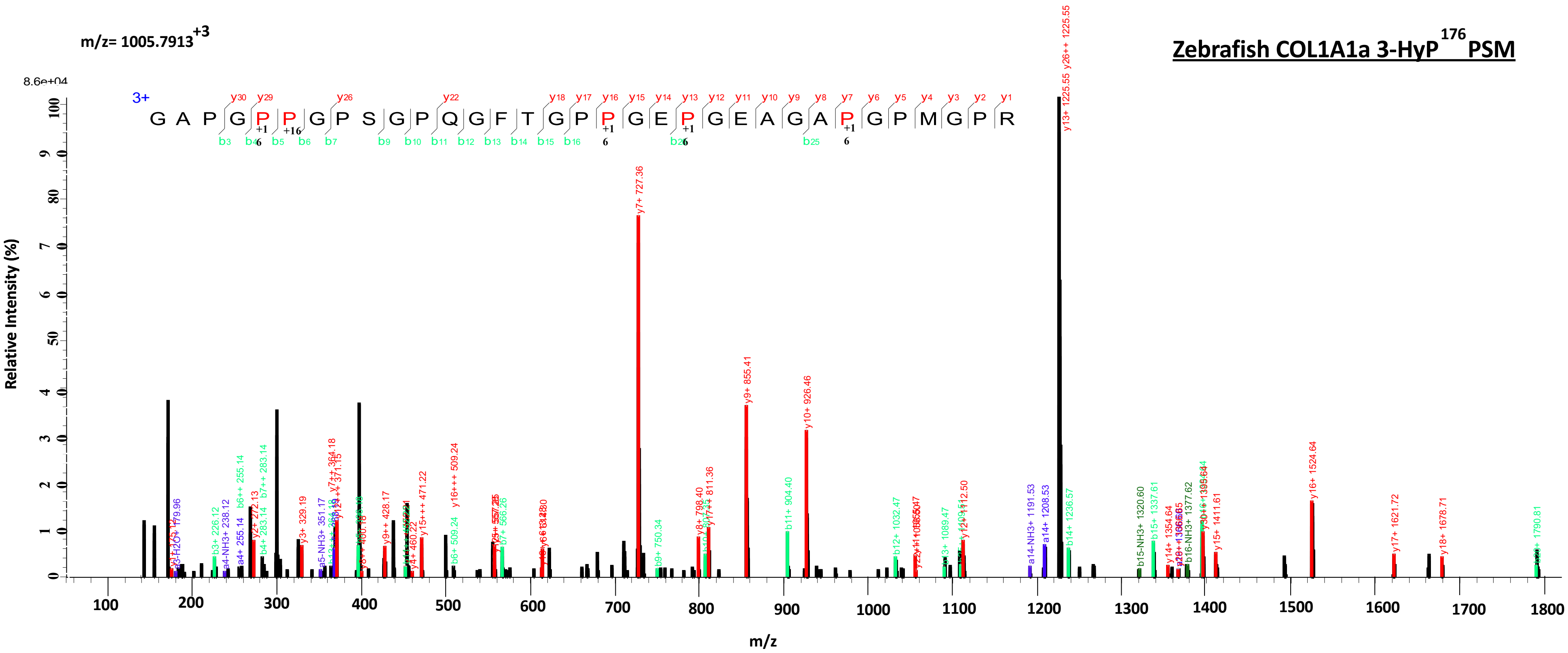

m/z= 1005.7913 +3
Zebrafish COL1A1a 3-HyP176 PSM
3+
G A P G P P G P S G P Q G F T G P P G E P G E A G A P G P M G P R
Relative Intensity (%)
m/z
y13+ 1225.55 y26++ 1225.55
y7+ 727.36
y9+ 855.41
y10+ 926.46
y16+ 1524.64

S2.2

m/z= 612.3027 +2

Zebrafish COL1A1a 3-HyP[401]

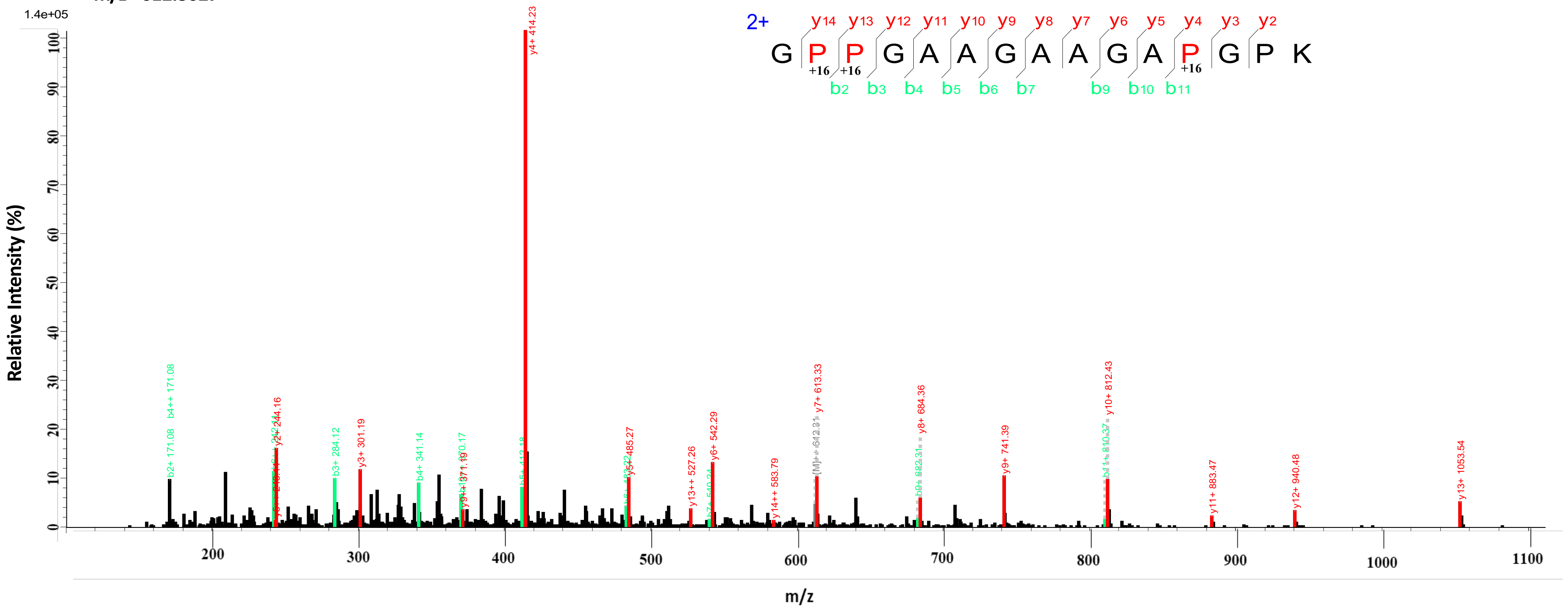

S2.3

m/z= 683.6754^{+3}

Zebrafish COL1A1a 3-HyP^{551} PSM

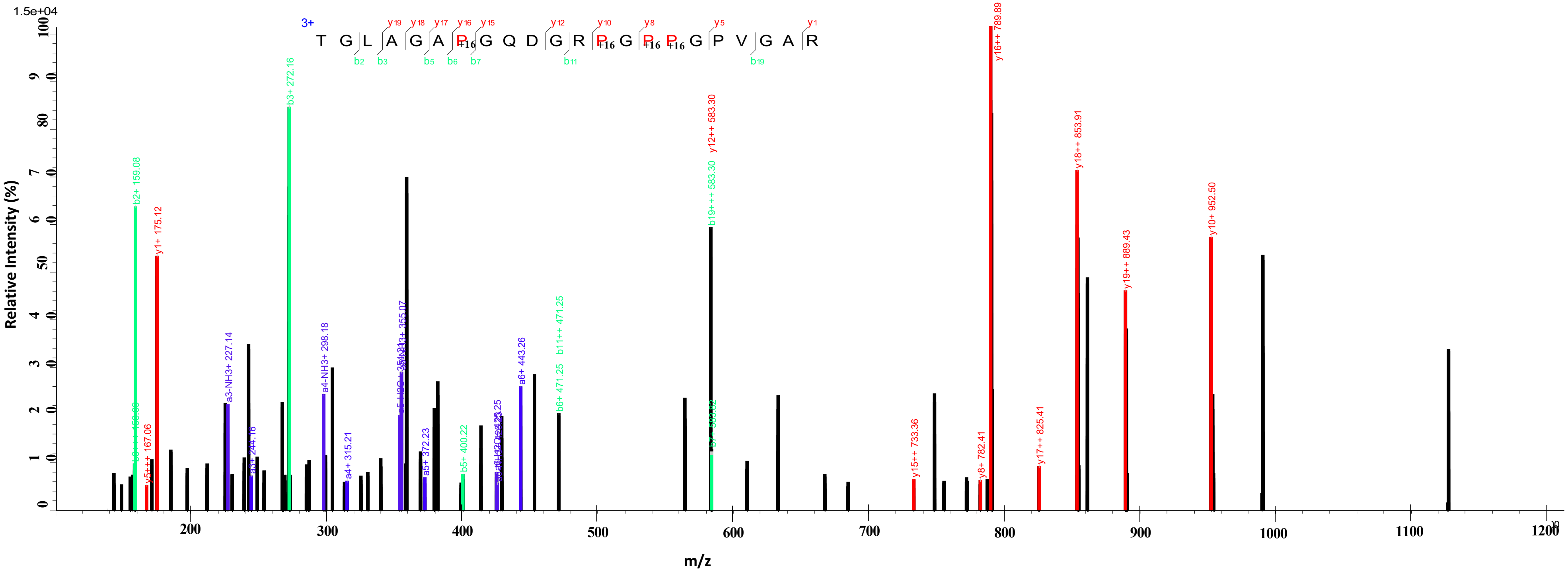

S2.4 m/z= 891.6615 +4

Zebrafish COL1A1a 3-HyP800

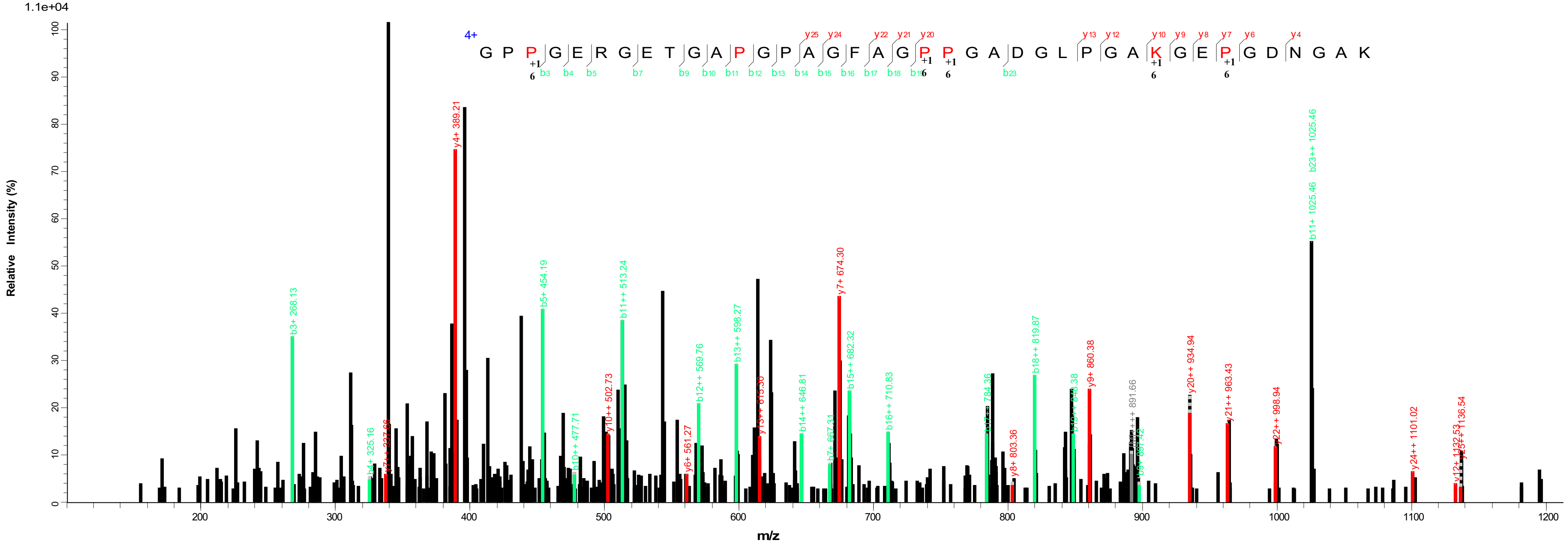

S2.5 m/z= 930.9360^{+2}

Zebrafish COL1A1a 3-HyP$^{869, 878}$

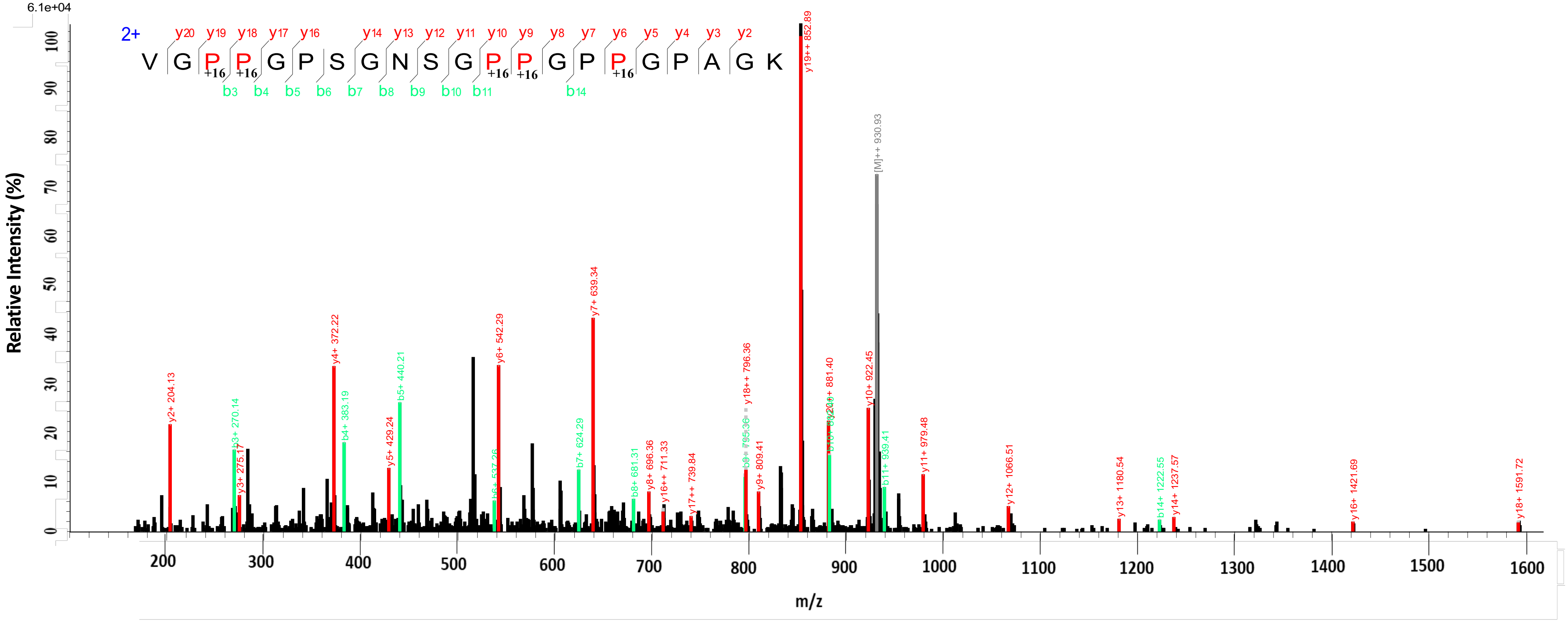

S2.6 m/z= 930.9403^{+2}

Zebrafish COL1A1a 3-HyP[869, 881]

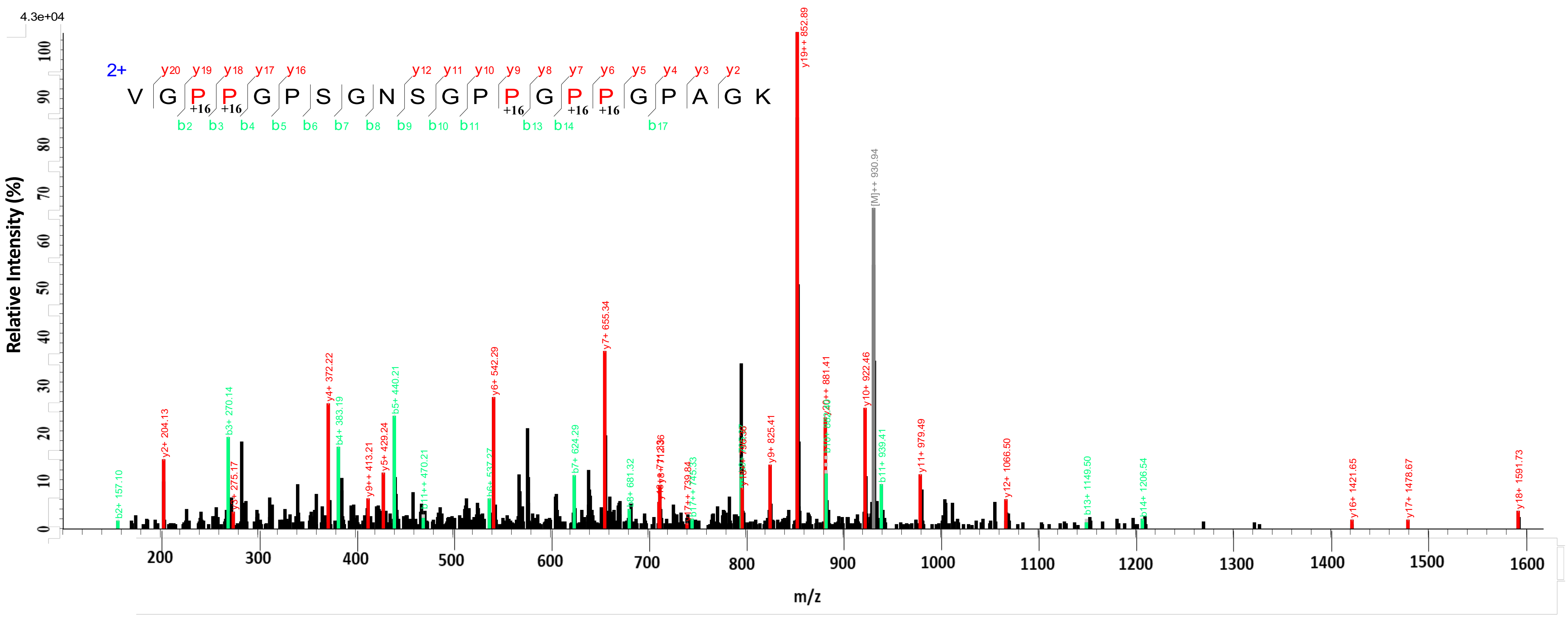

S2.7
m/z= 985.7098 +4

Zebrafish COL1A1a 3-HyP992

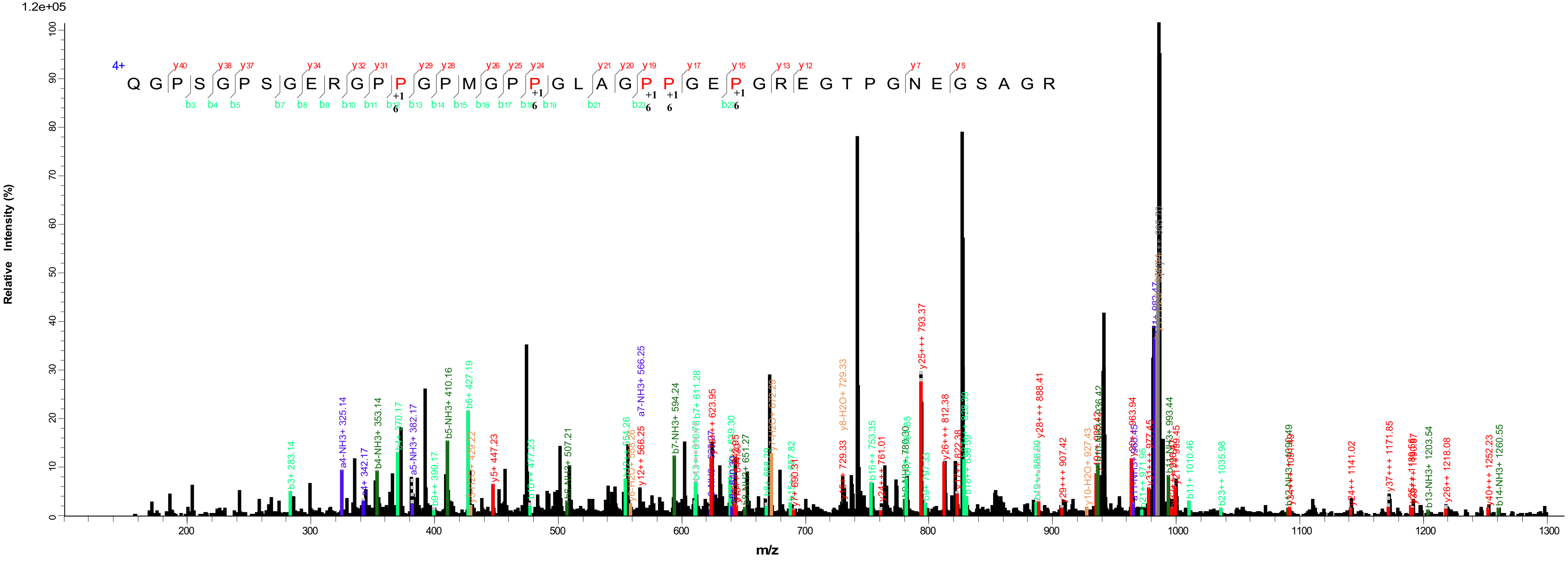

S2.8

m/z= 889.0699 +3

Zebrafish COL1A1a 3-HyP1103PSM

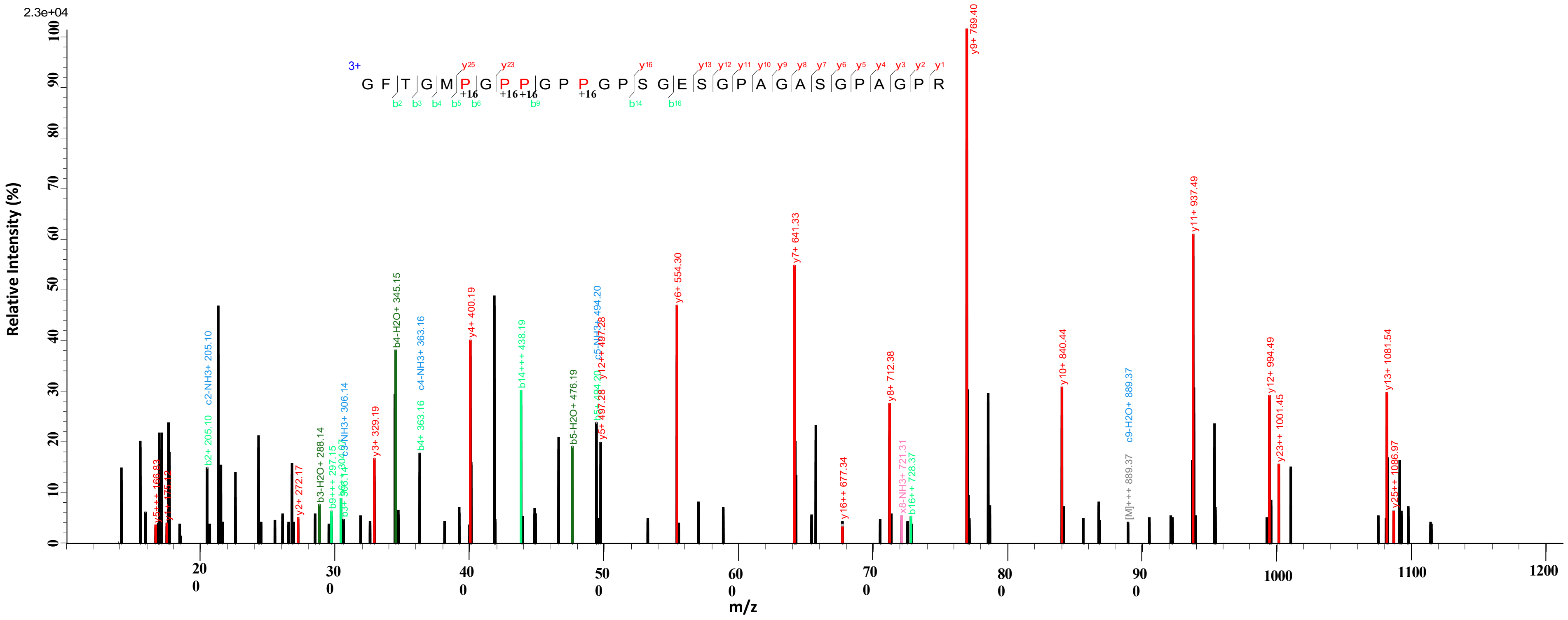

S2.9

m/z= 894.4028 +3

Zebrafish COL1A1a 3-HyP[1106] PSM

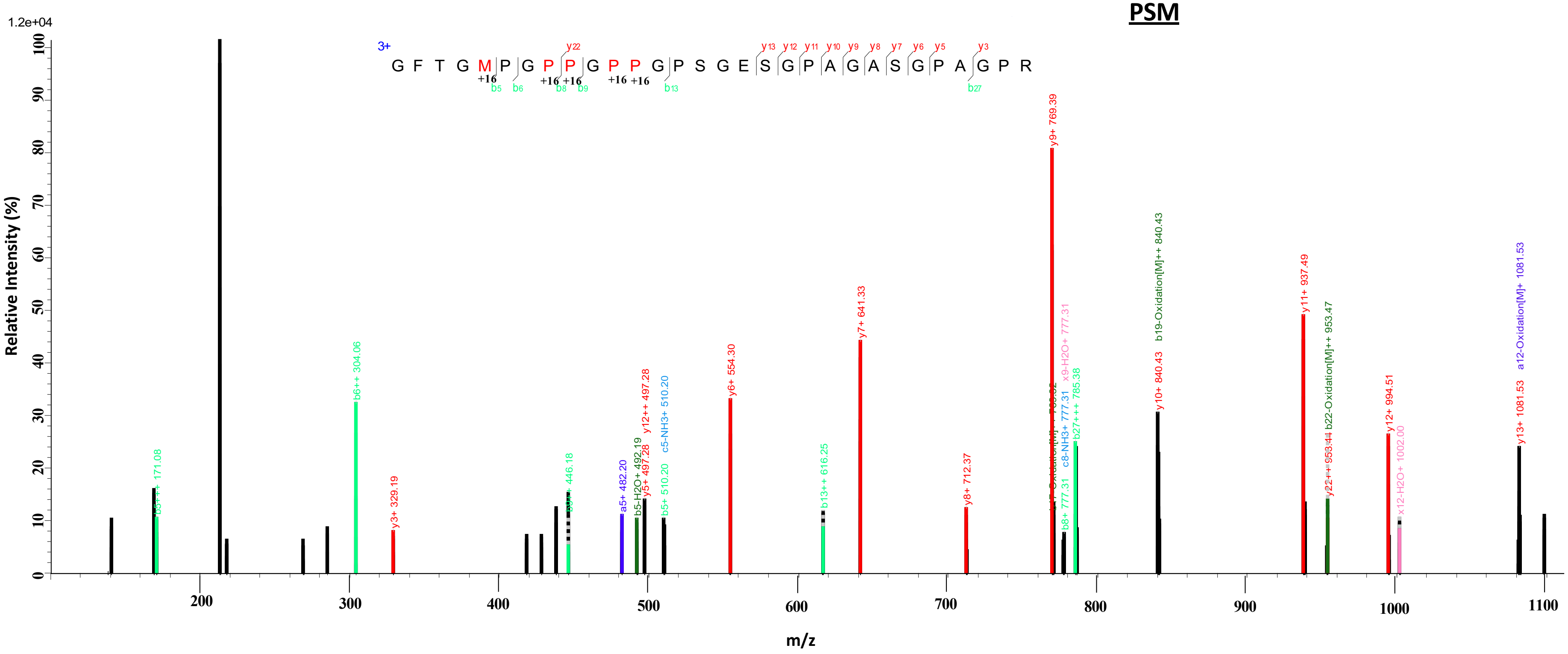

S2.10

m/z=776.8748^{+2}

Zebrafish COL1A1a 3-HyP^{1148} PSM

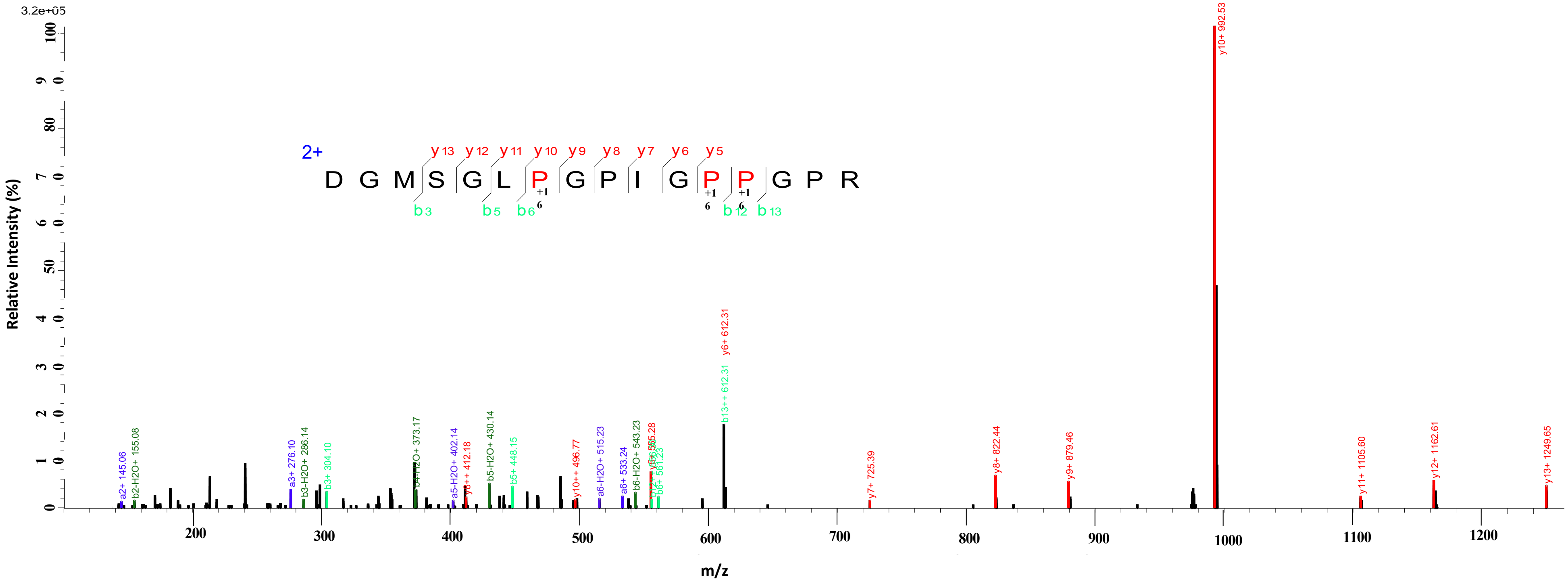

S2.11 m/z= 1173.2279^{+3}

Zebrafish COL1A1a 3-HyP^{1166}

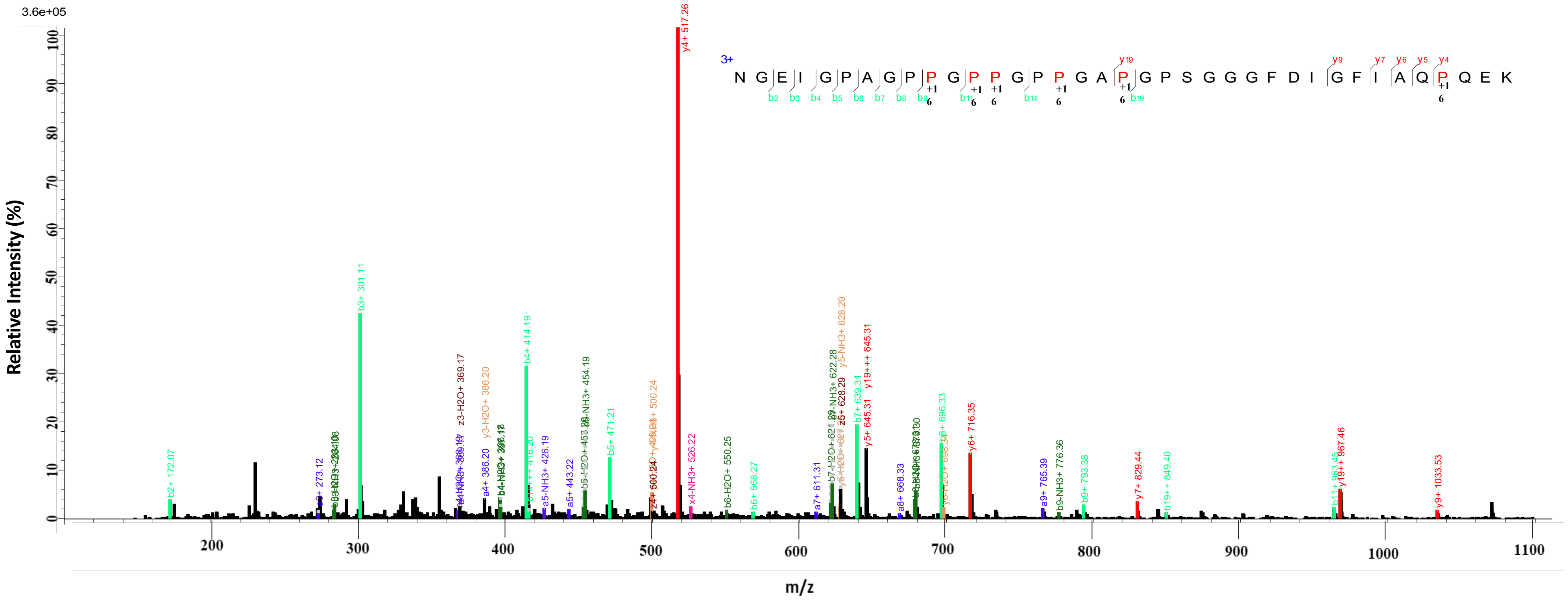

S2.12 m/z= 865.8969^{+4}

Zebrafish COL1A1a GK^{261}

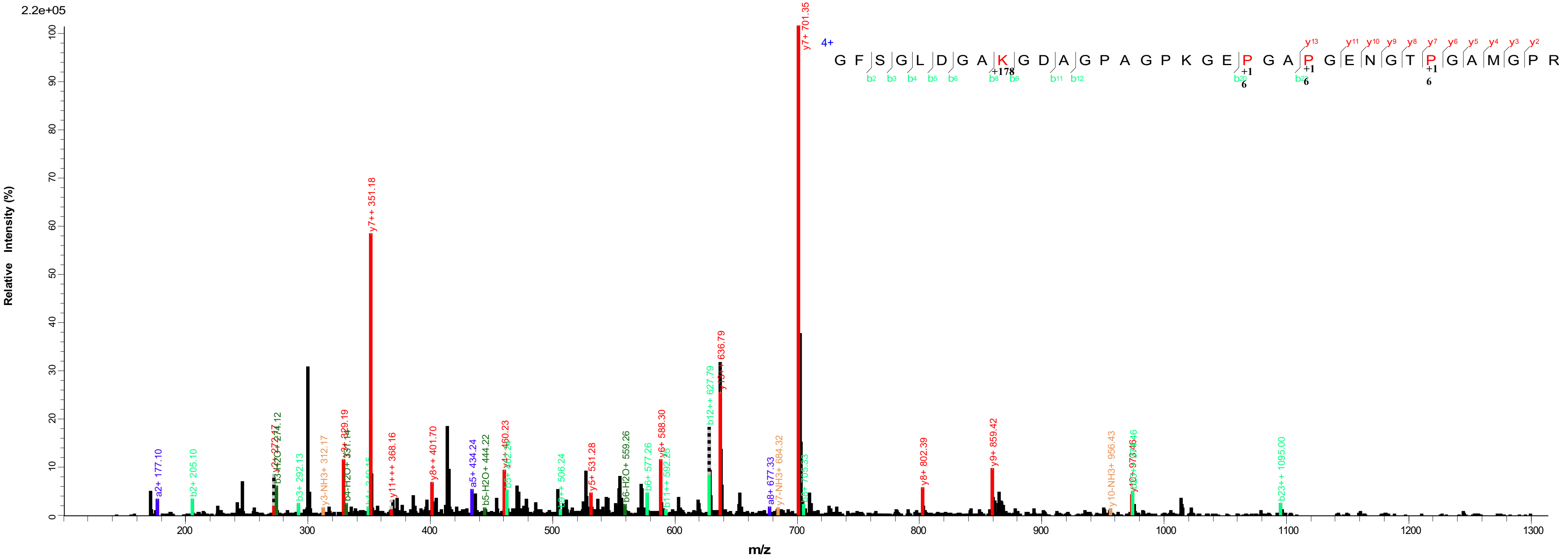

S2.13

m/z= 653.3007 +3

Zebrafish COL1A1a GG-HyK[261] and HyK[270] PSM

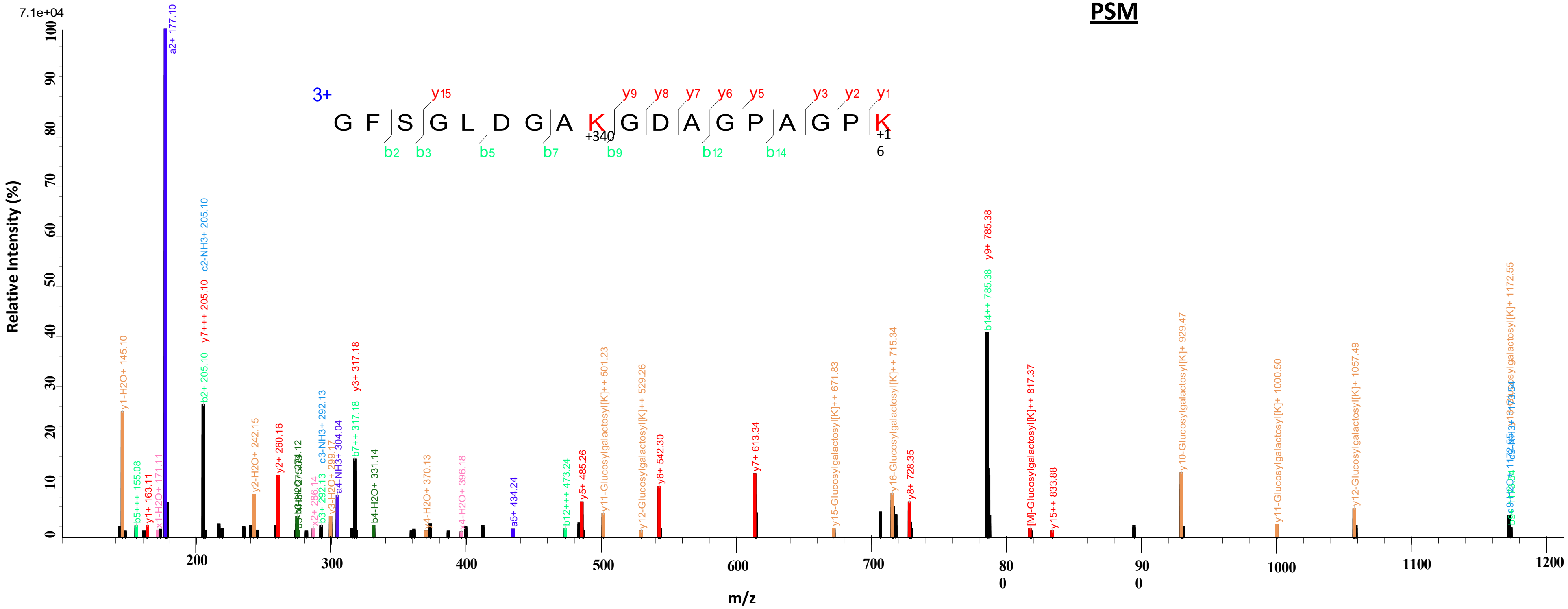

S2.14

m/z= 850.7228 +3

Zebrafish COL1A1a HyK336 PSM

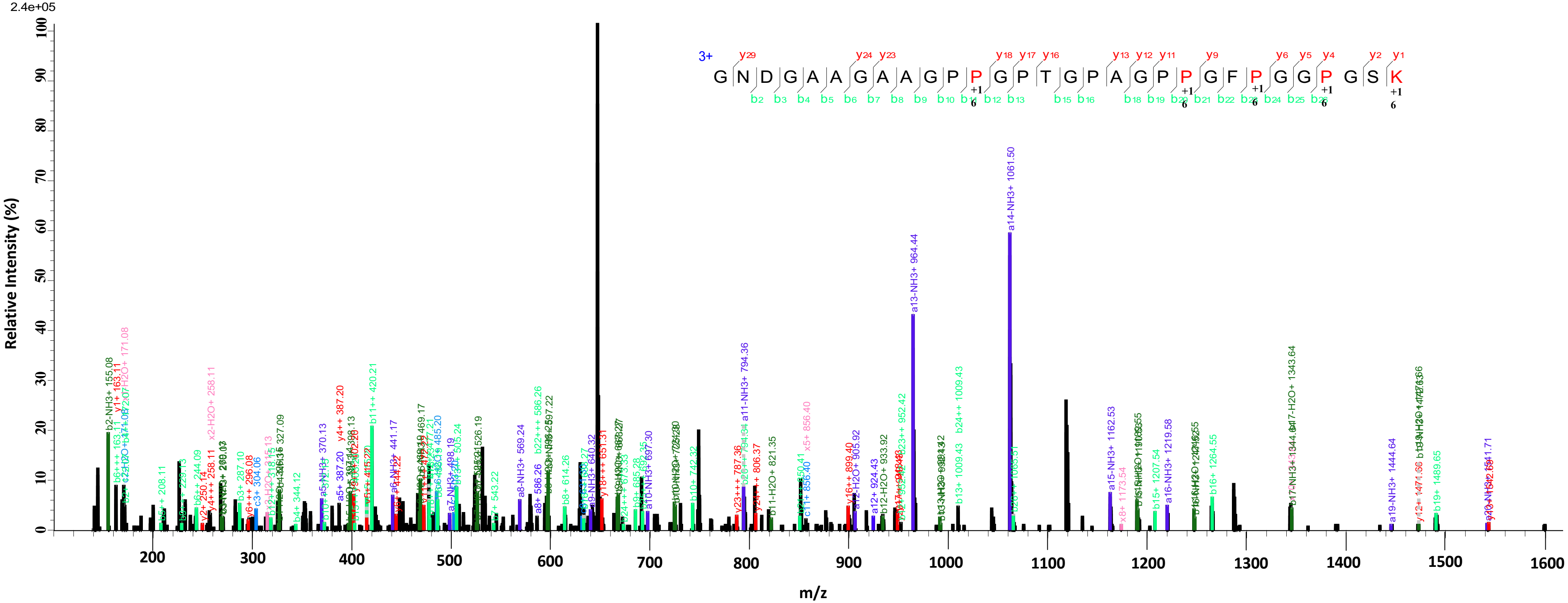

S2.15 m/z= 781.3598^{+5}

Zebrafish COL1A1a GG-HyK^{432} HyK^{426}

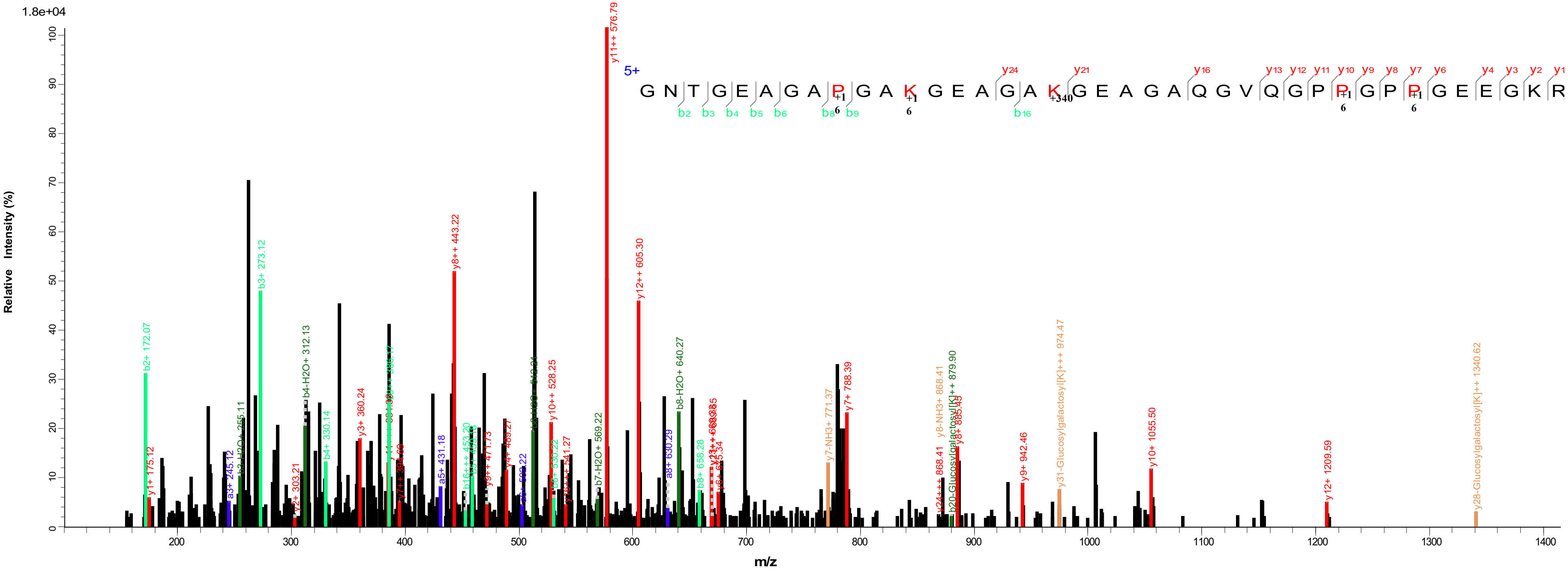

S2.16 m/z= 568.2762^{+3}

Zebrafish COL1A1a GG-HyK^{504}

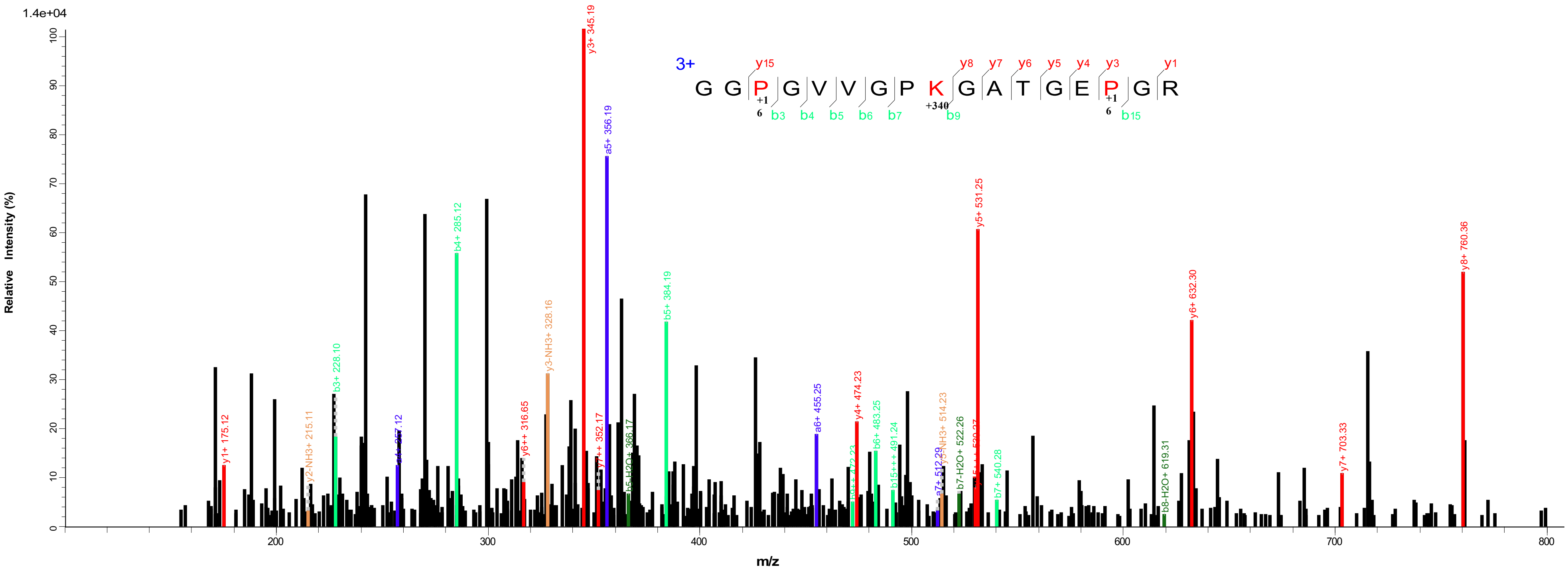

S2.17 m/z= 729.0046 +3

Zebrafish COL1A1a GG-HyK^{570}PSM

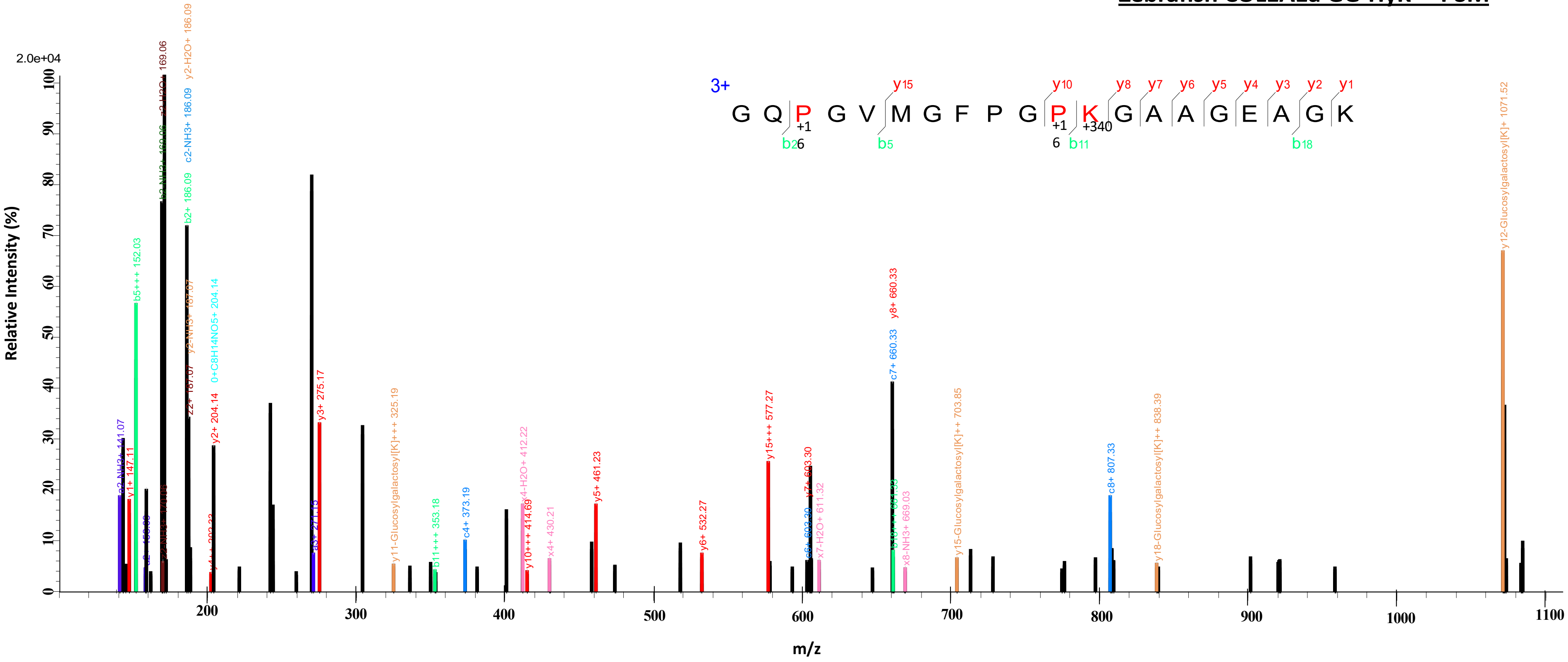

S2.18

m/z= 1189.8509 +3

Zebrafish COL1A1a GG-HyK693

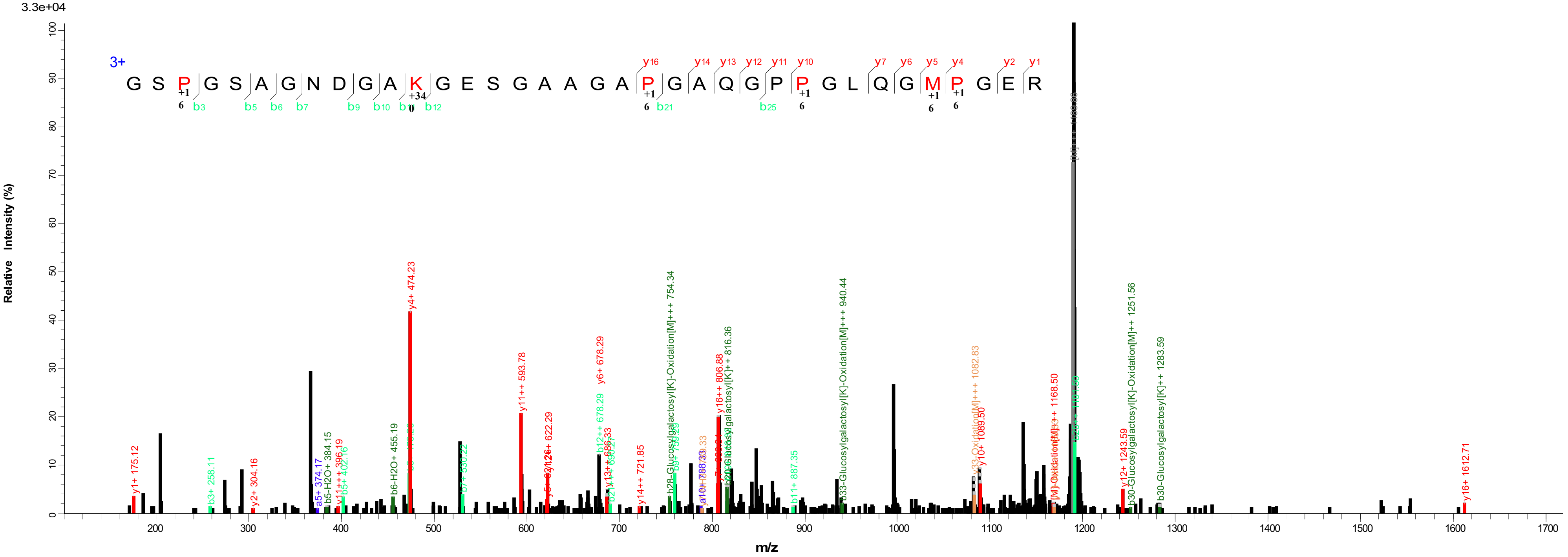

S2.19

m/z= 903.1029 +3

Zebrafish COL1A1a G-HyK 846 PSM

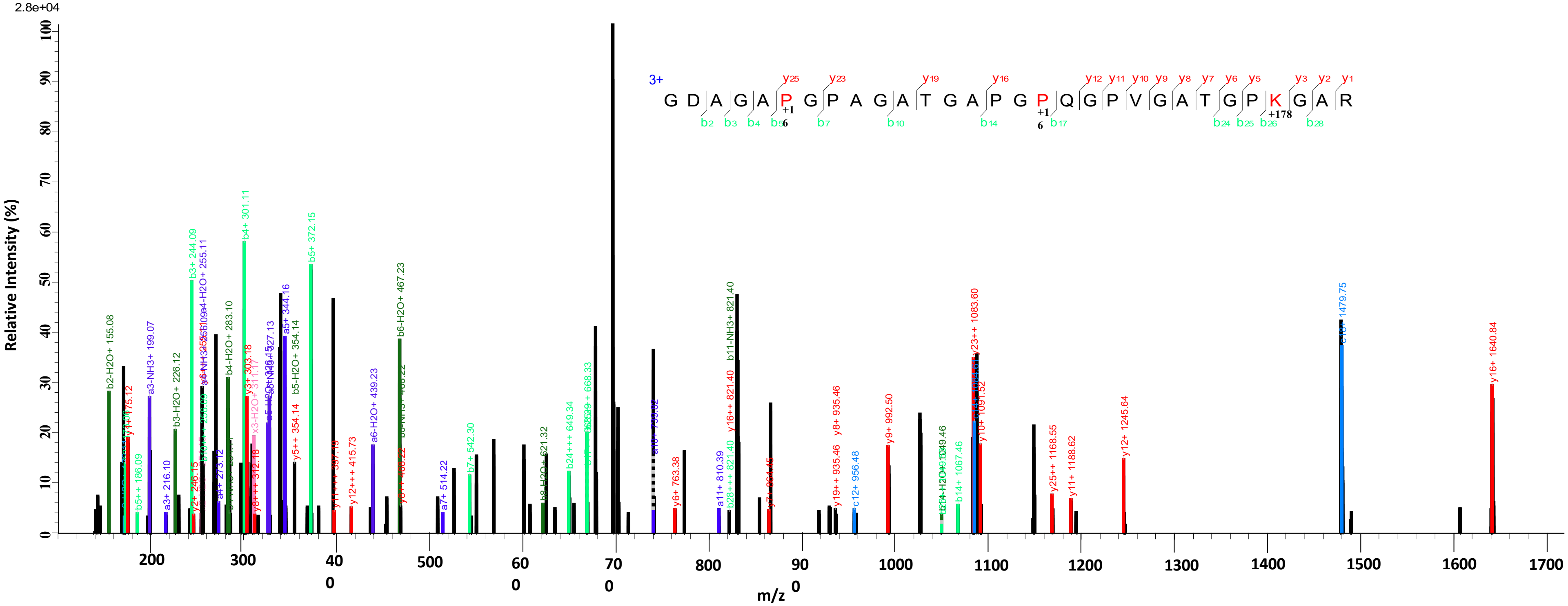

S2.20 m/z= 957.1208+3

Zebrafish COL1A1a GG-HyK846 PSM

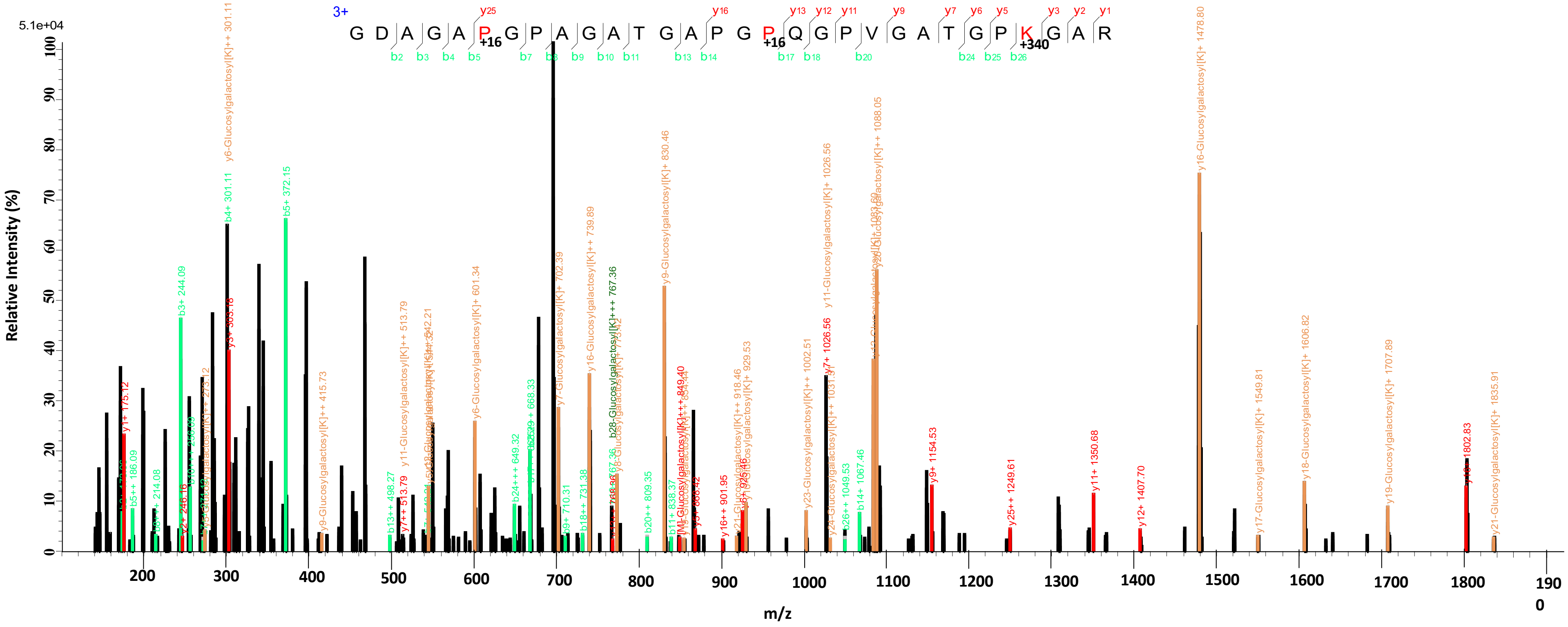

S2.21 m/z= 867.6599^{+4}

Zebrafish COL1A1a GG-HyK[1017]

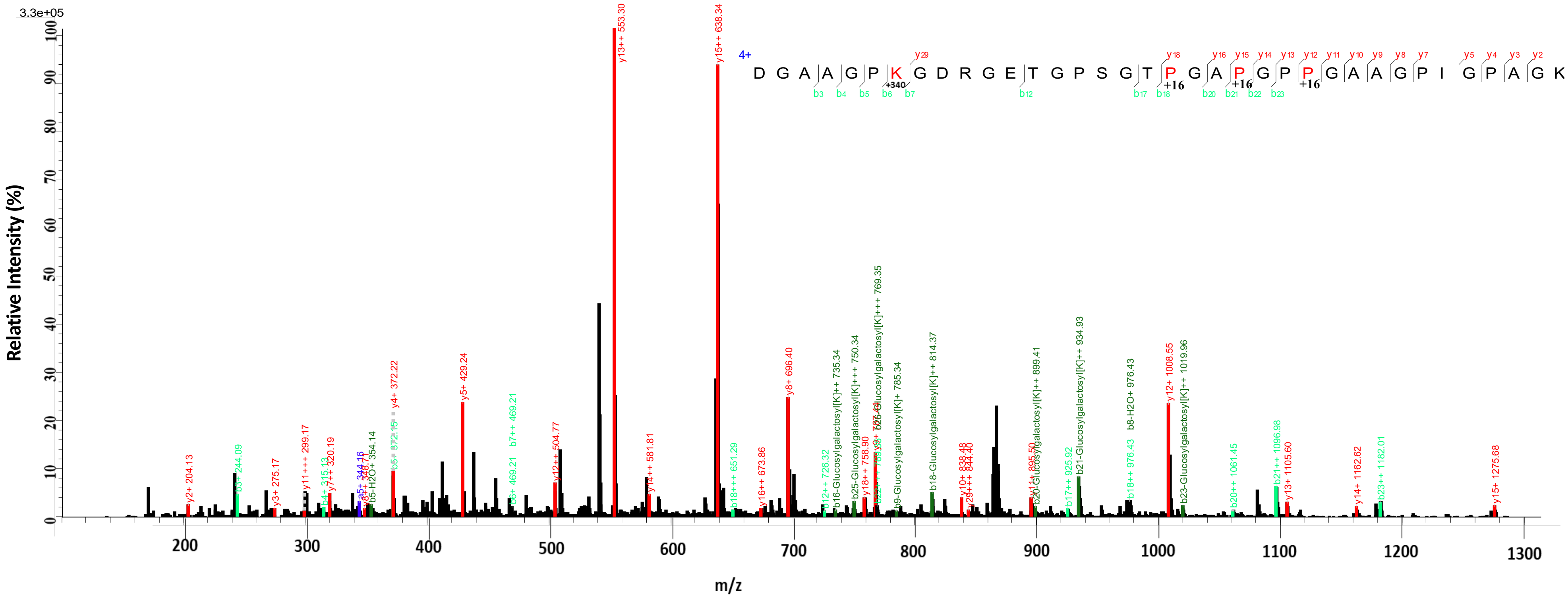

S2.22

m/z= 1011.1247 $^{+3}$

Zebrafish COL1A1b 3-HyP ^{176}

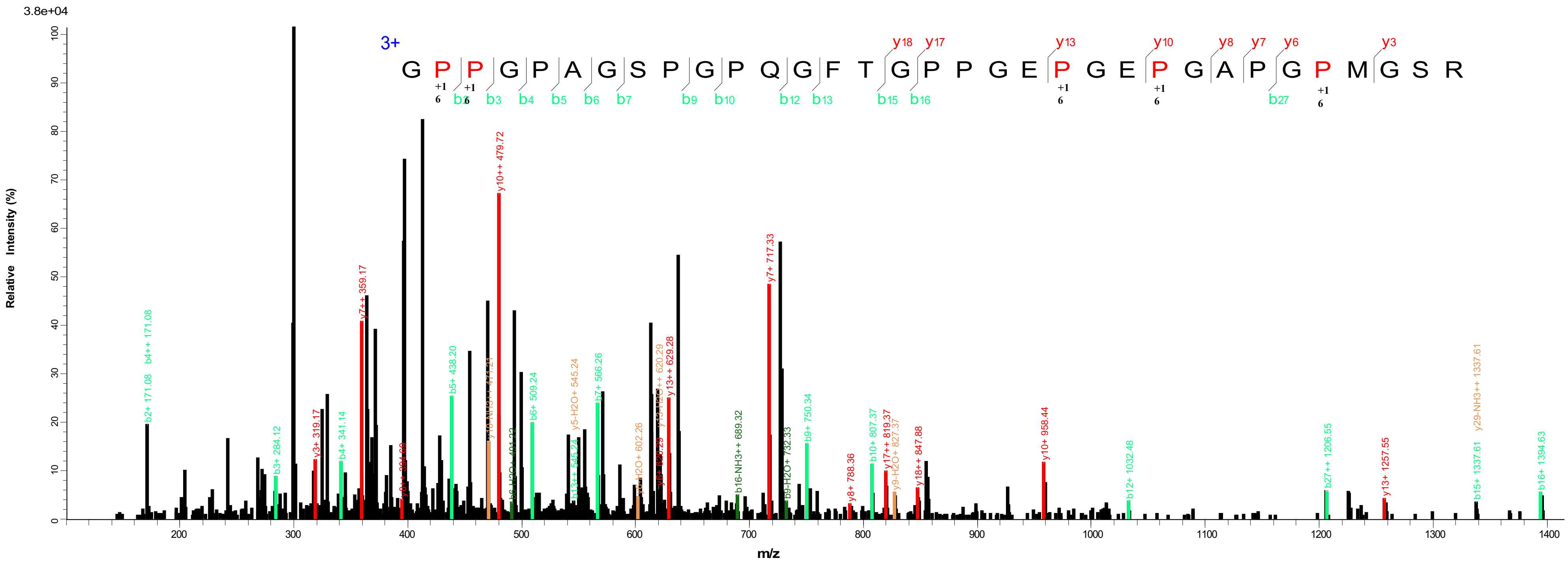

S2.23

m/z= 1021.7845^{+3}

Zebrafish COL1A1b 3-HyP191

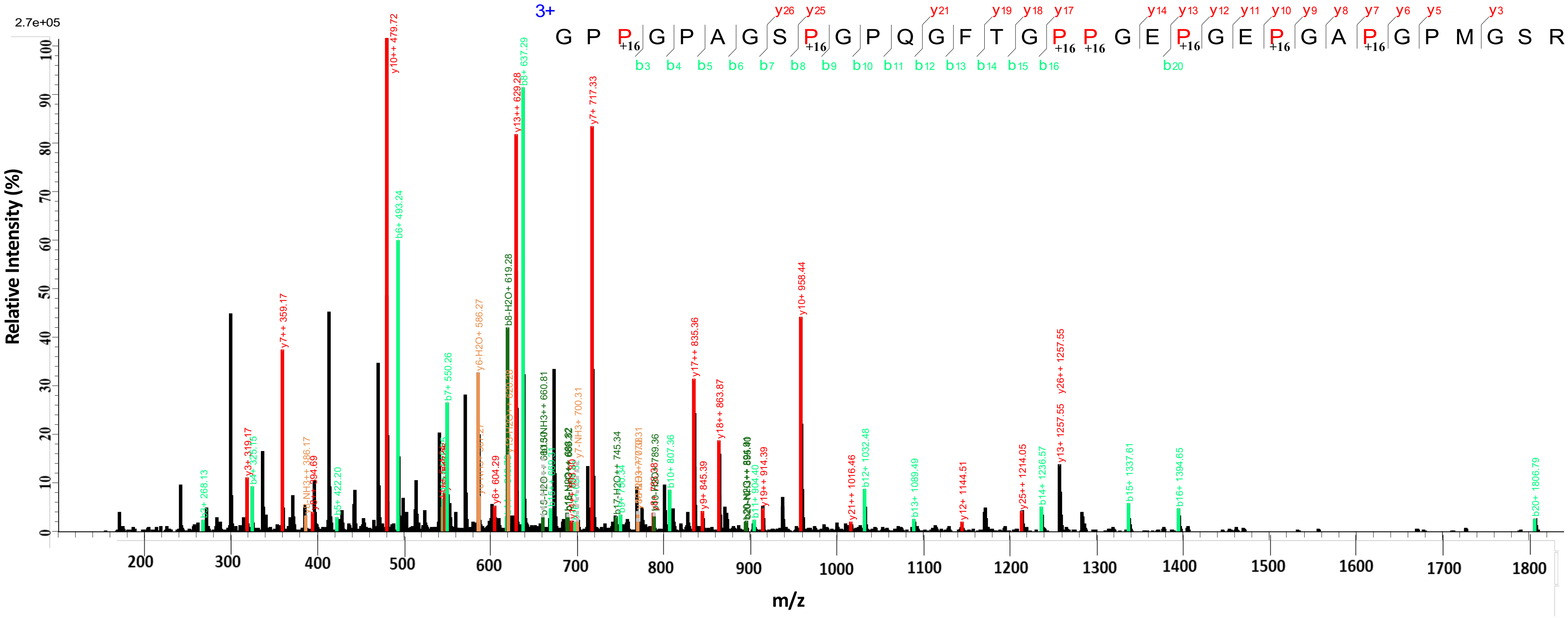

S2.24 m/z= 379.8611^{+3}

Zebrafish COL1A1b 3-HyP^{302}

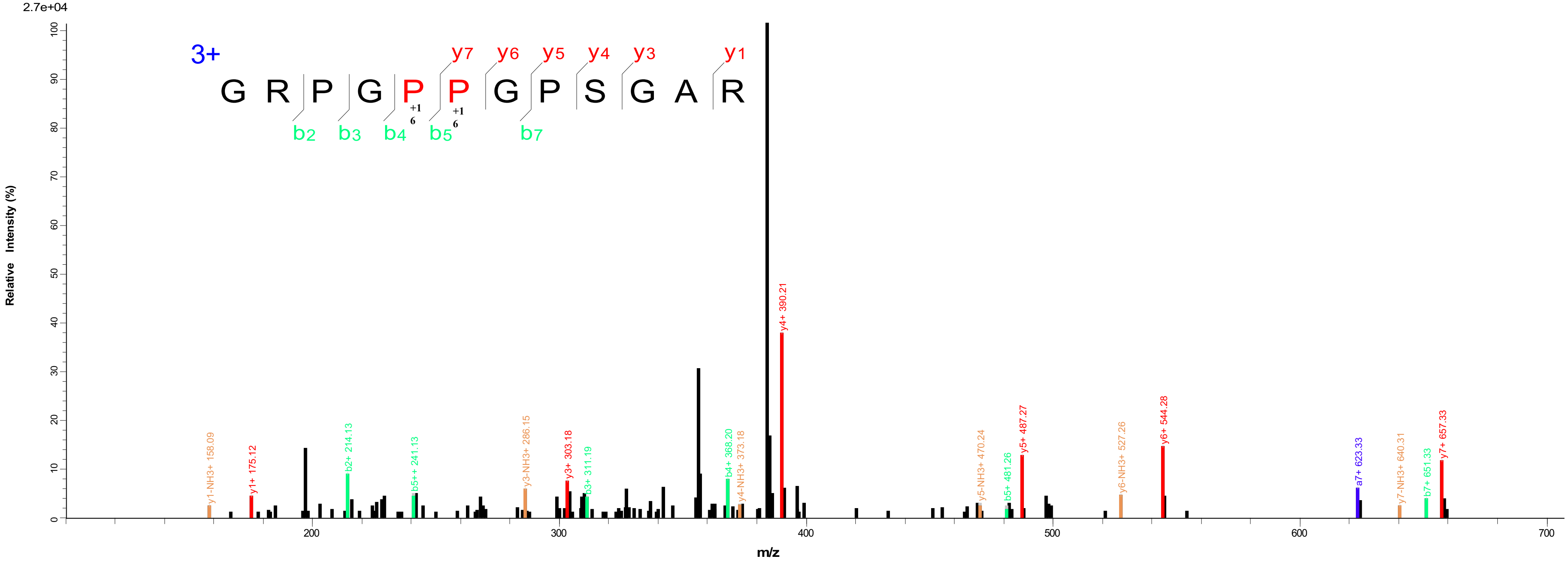

S2.25

m/z= 659.8209^{+2}

Zebrafish COL1A1b 3-HyP[404]

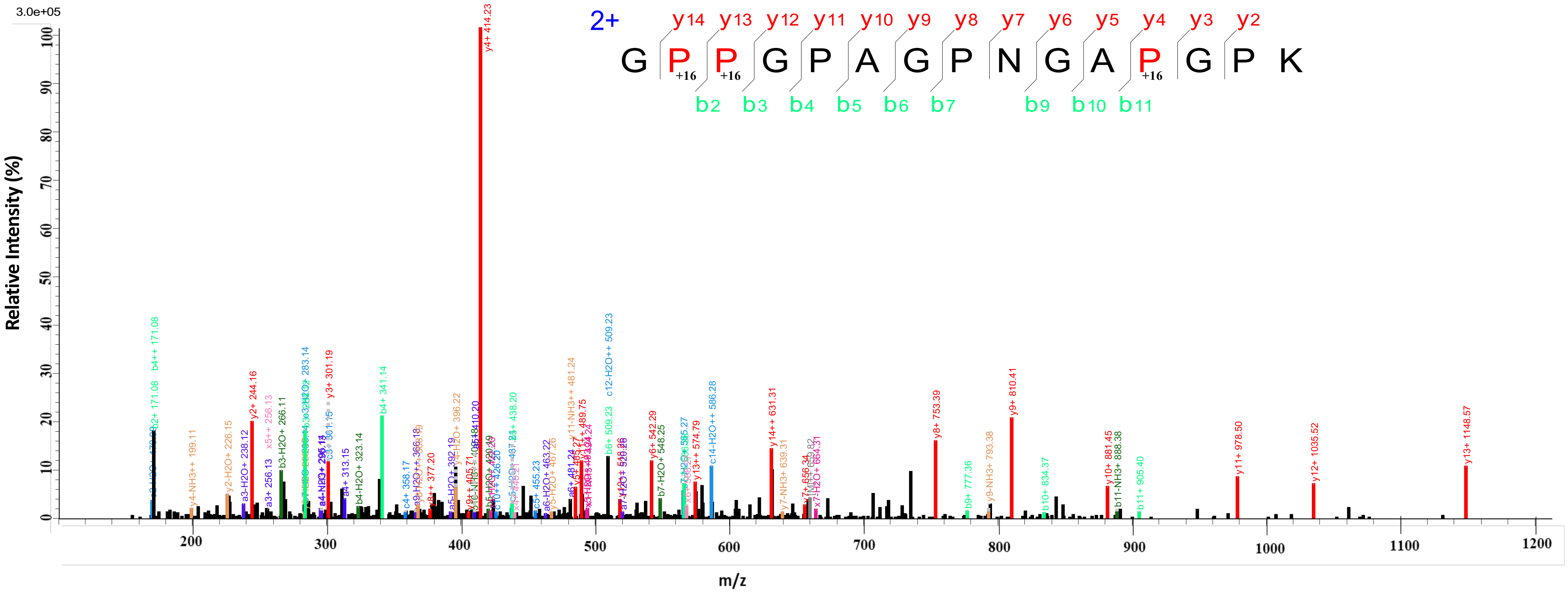

S2.26

m/z= 669.3250[+2]

Zebrafish COL1A1b 3-HyP[470]

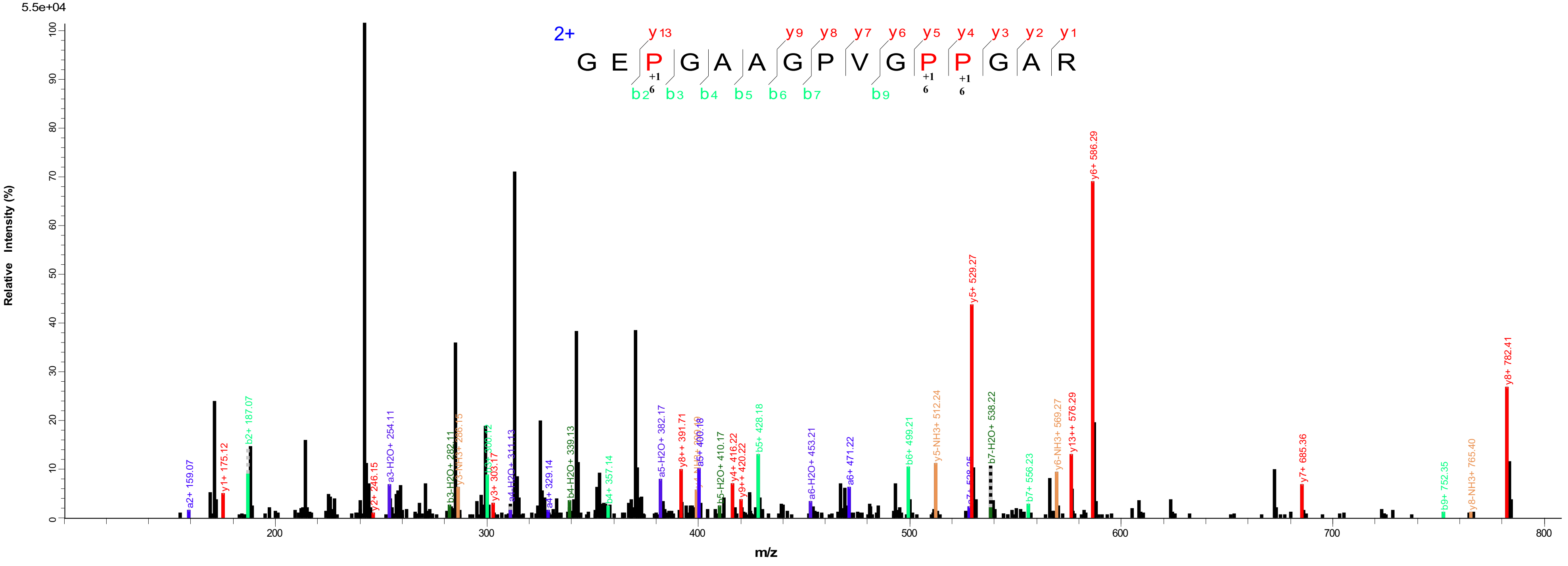

S2.27
m/z= 669.6452 +3

Zebrafish COL1A1b 3-HyP554

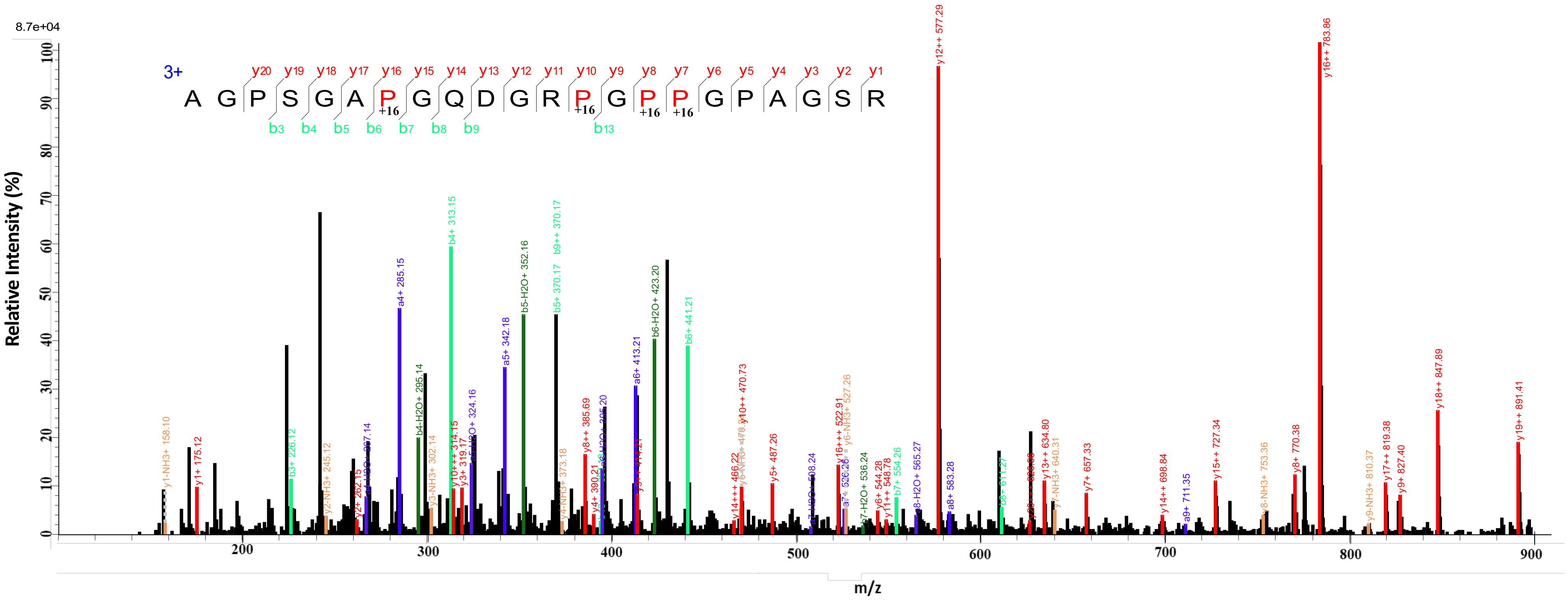

S2.28

m/z= 903.4370 $^{+2}$

Zebrafish COL1A1b 3-HyP 872, 881

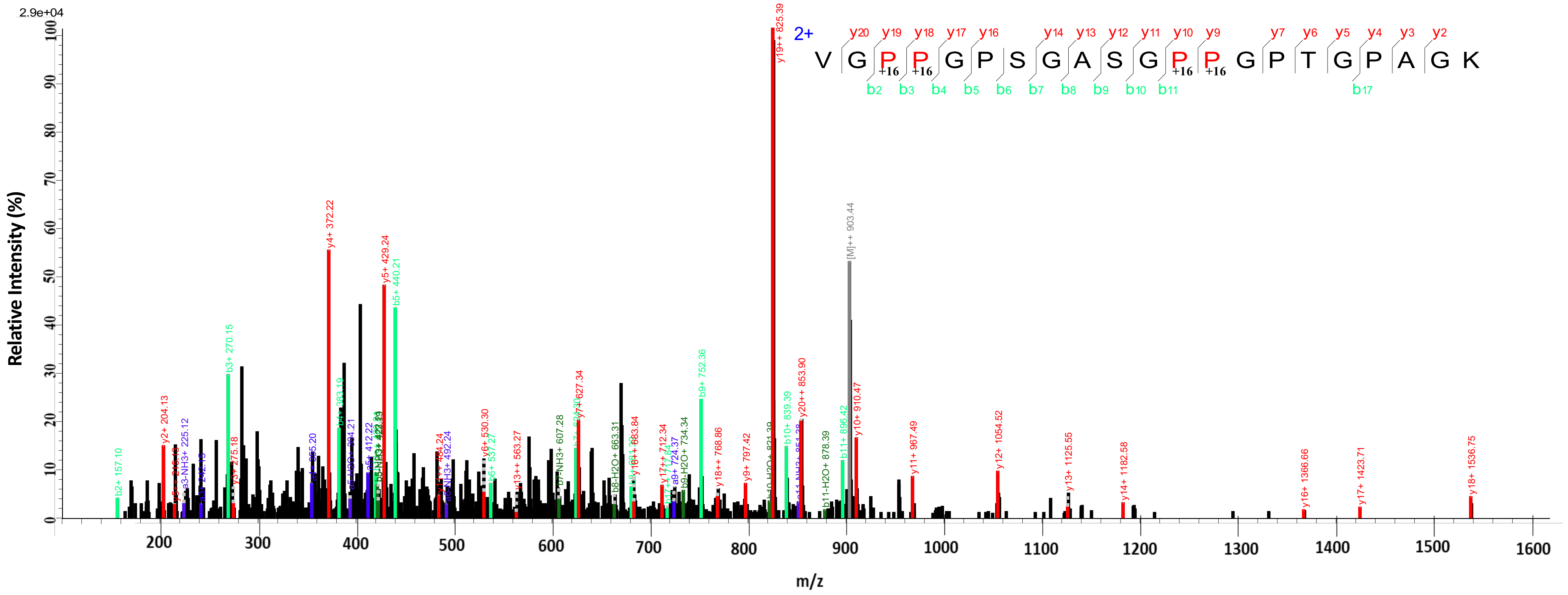

S2.29

Zebrafish COL1A1b 3-HyP[983] PSM

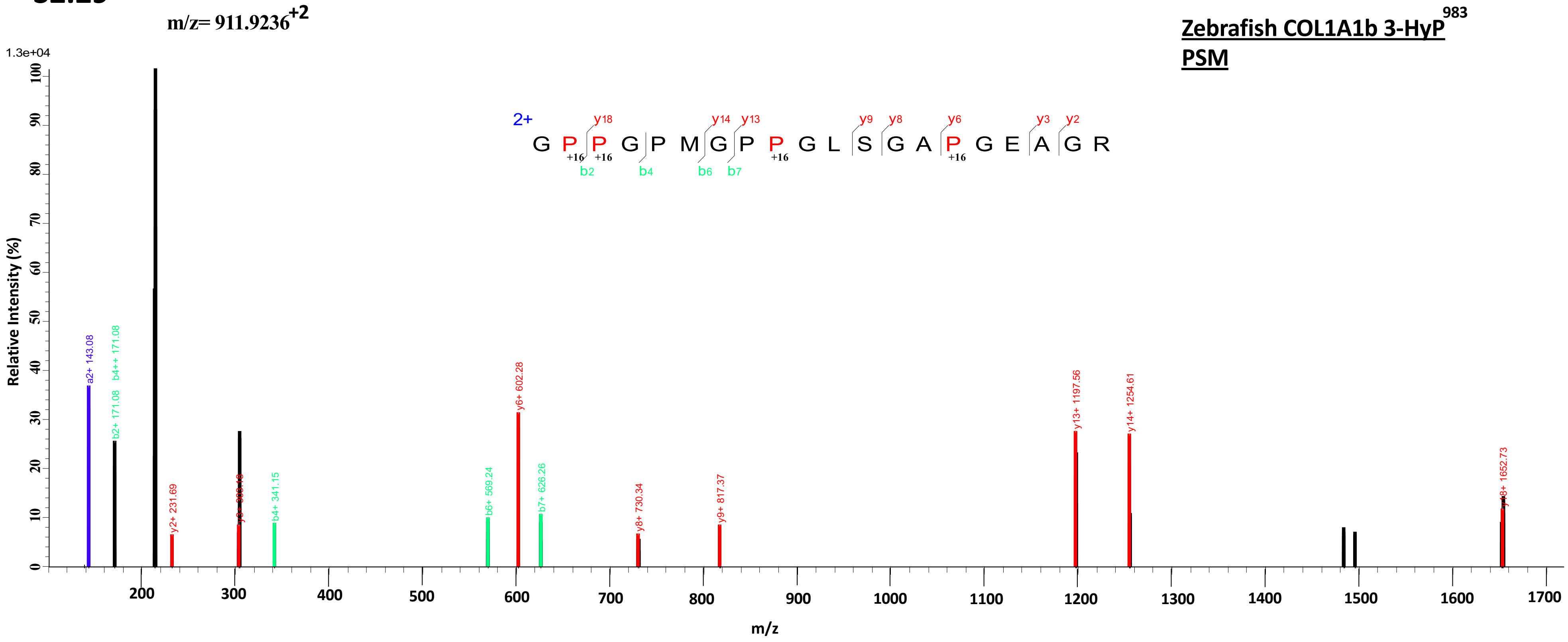

S2.30

m/z= 608.9786^{+3}

Zebrafish COL1A1b 3-HyP^{989}

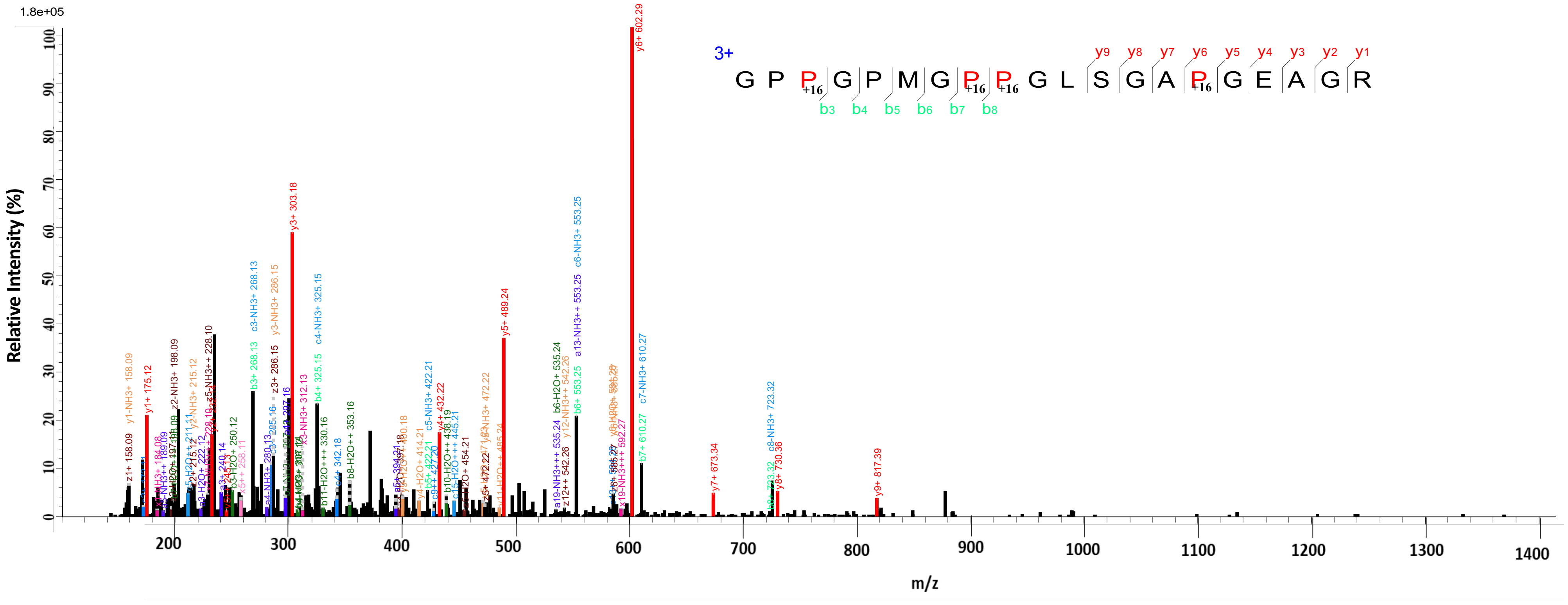

S2.31

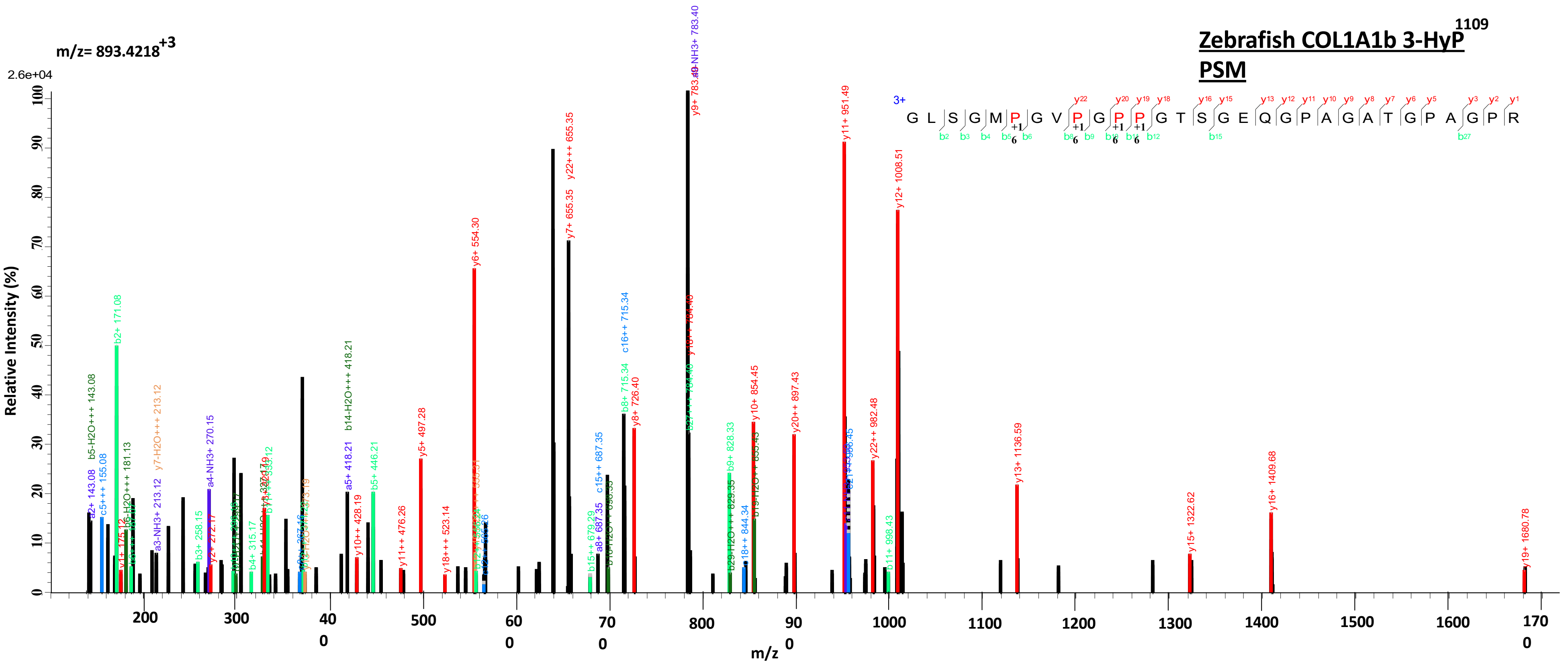

m/z= 893.4218+3
Zebrafish COL1A1b 3-HyP 1109 PSM
Relative Intensity (%)
m/z
2.6e+04
G L S G M P G V P G P P G T S G E Q G P A G A T G P A G P R

S2.32

Zebrafish COL1A1b 3-HyP[1151] PSM

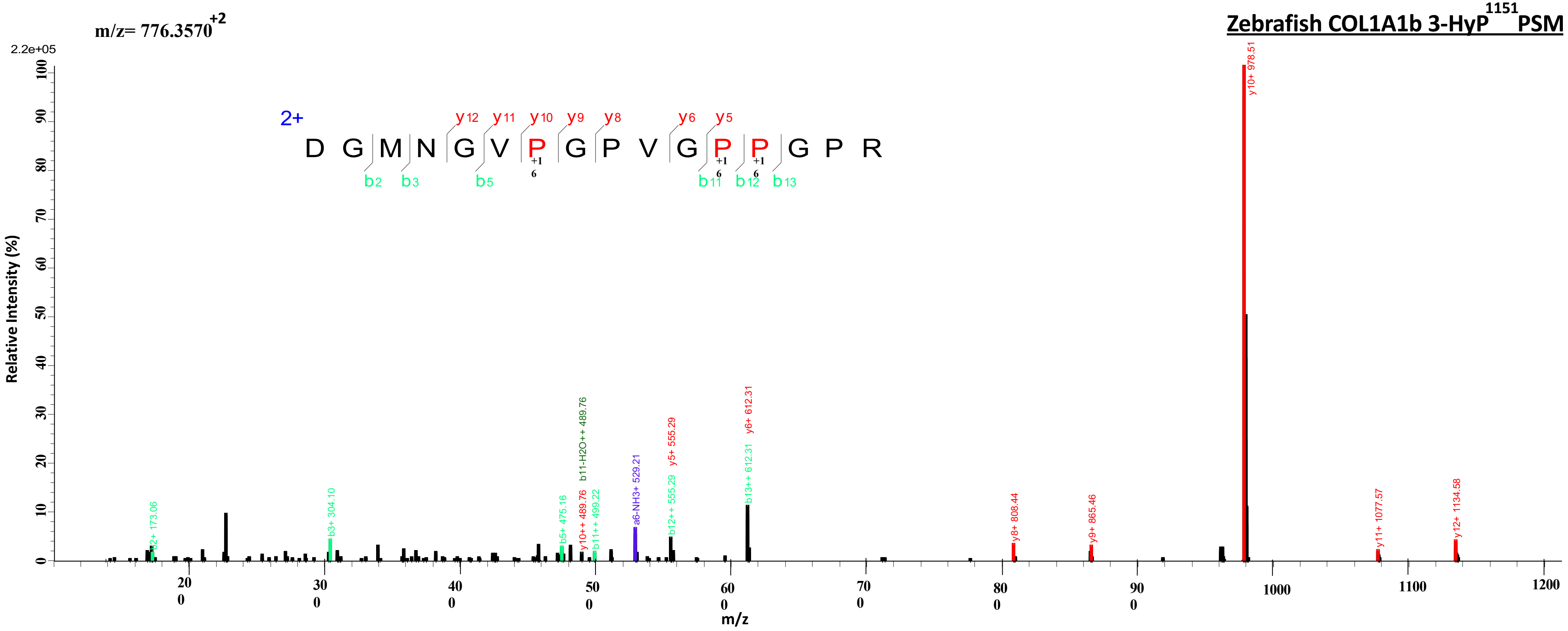

S2.33

m/z= 1027.9785^{+4}

Zebrafish COL1A1b 3-HyP[1175]

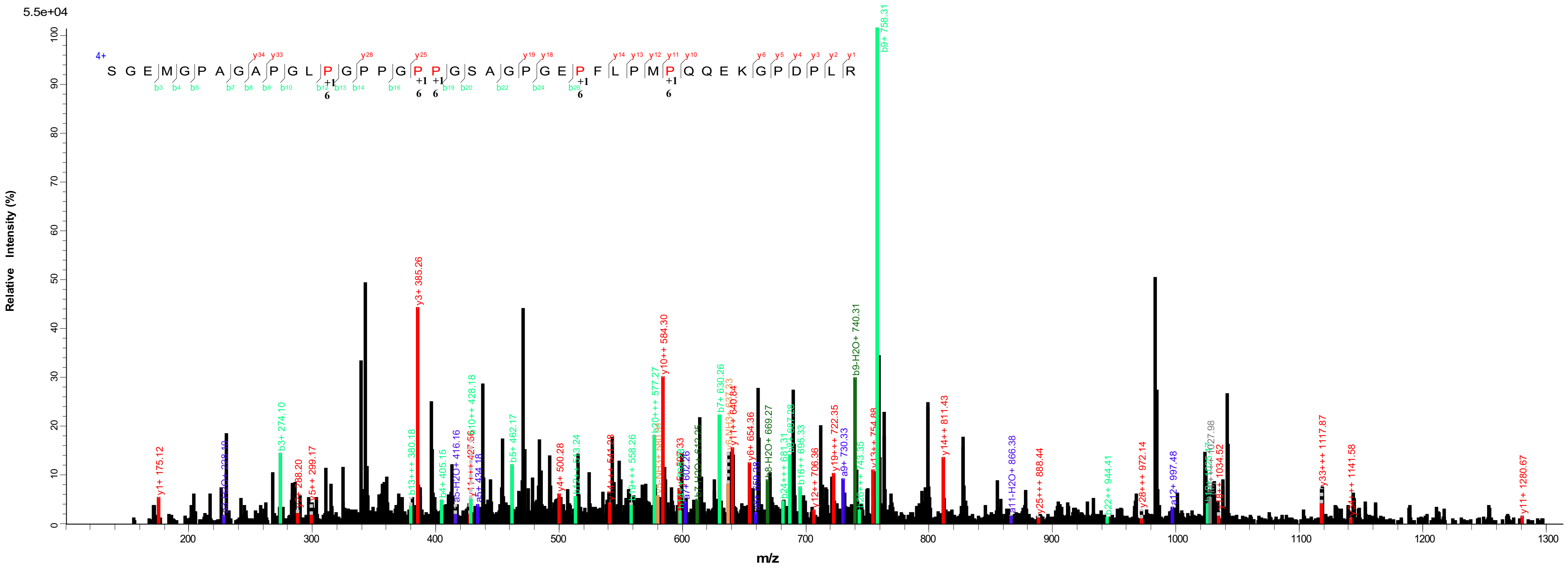

S2.34

m/z= 1152.1869+3

Zebrafish COL1A1b HyK158

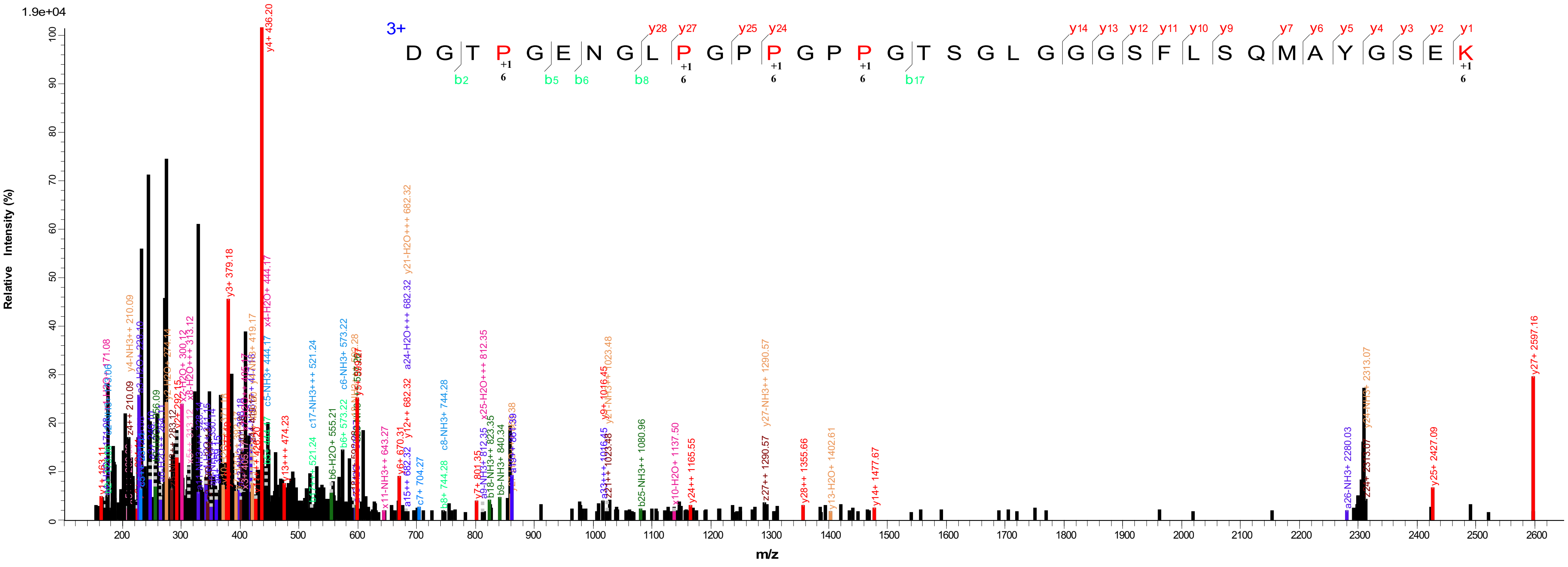

S2.35

m/z= 939.9584^{+2}

Zebrafish COL1A1b GG-HyK[252]

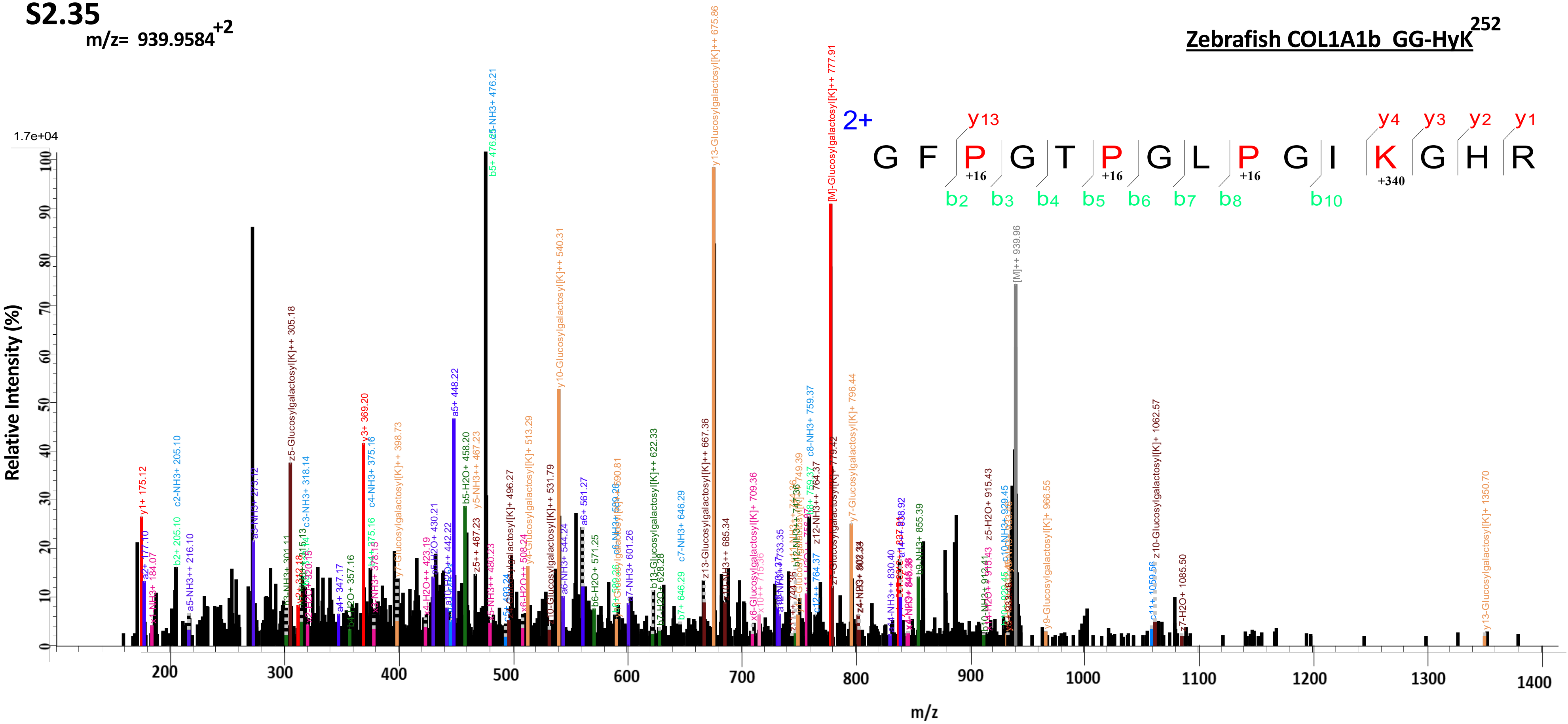

S2.36

m/z= 667.3167 +3

Zebrafish COL1A1b GG-HyK264, HyK273 PSM

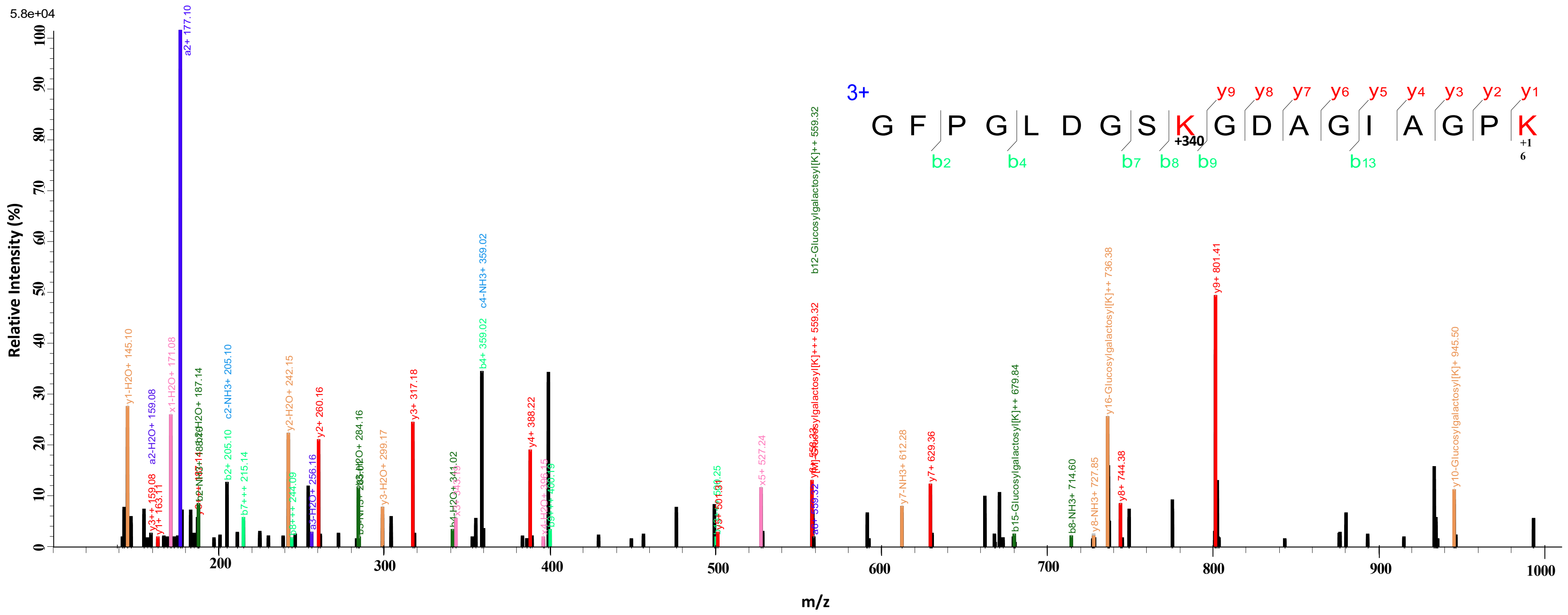

S2.37

m/z= 904.4053 +3

Zebrafish COL1A1b GG-HyK 273

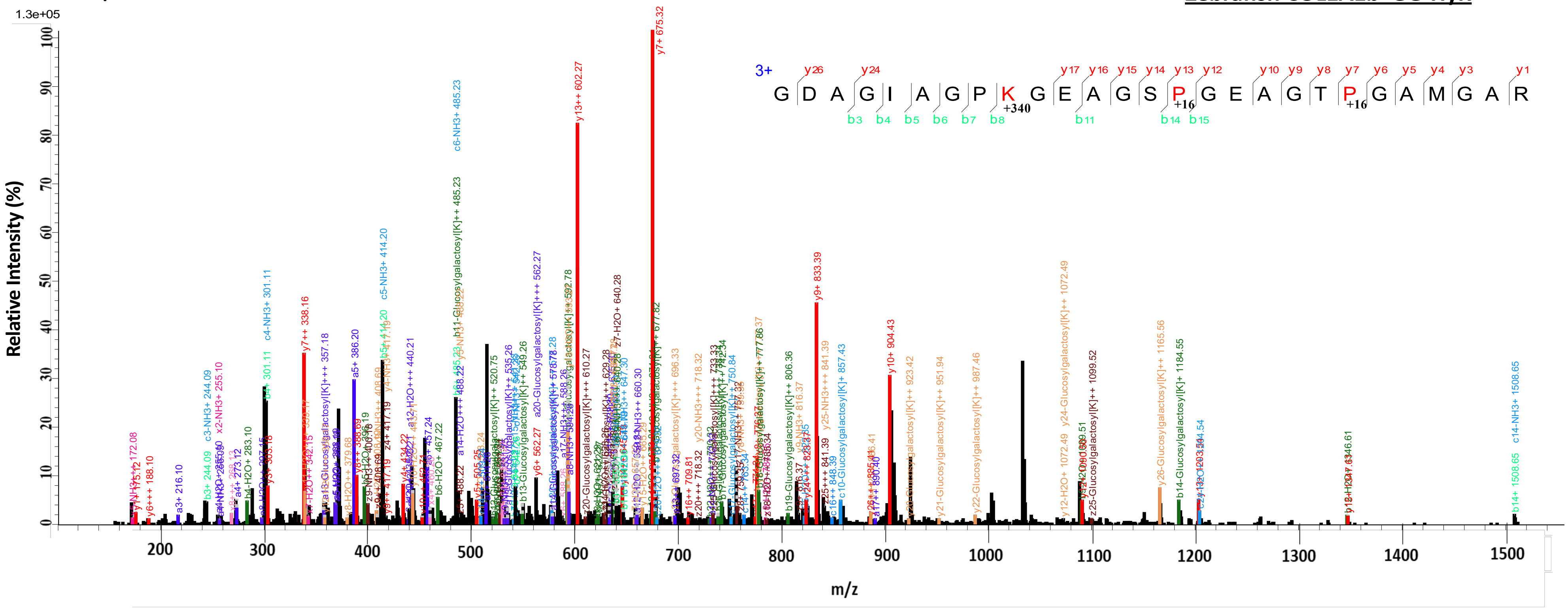

S2.38

m/z= 850.3848 +3

Zebrafish COL1A1b G-HyK[273]

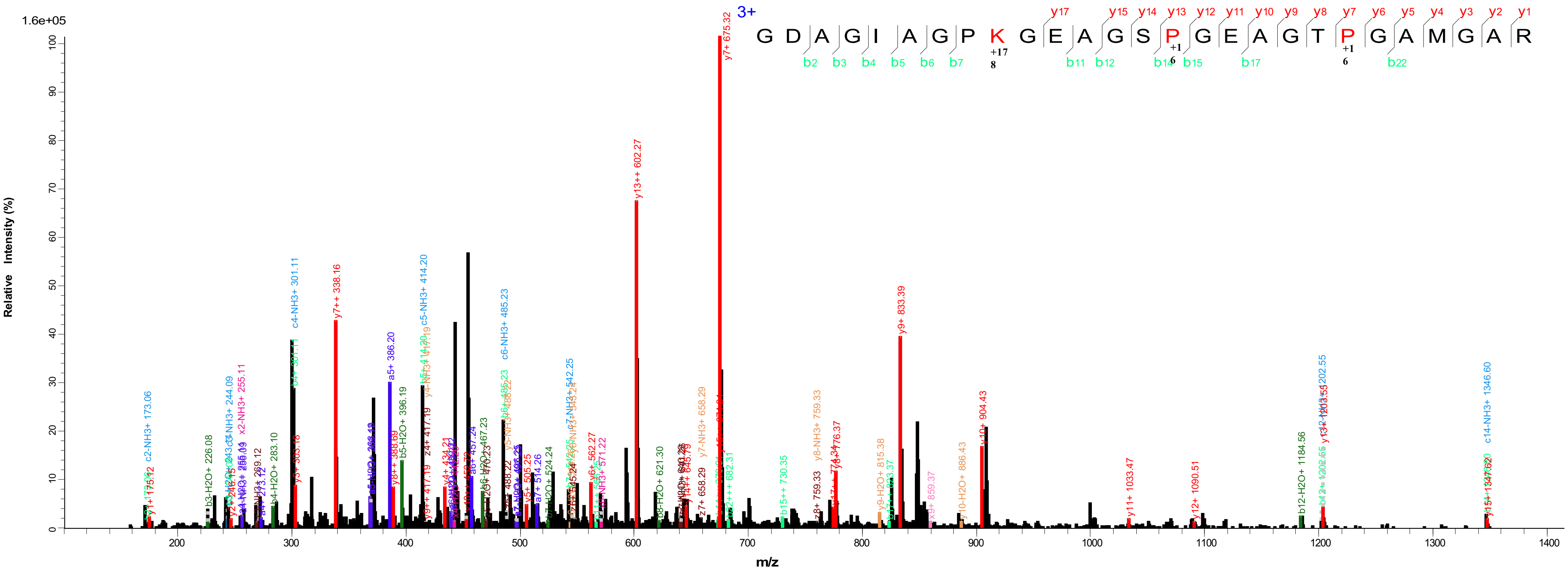

S2.39

m/z= 855.7258^{+3}

Zebrafish COL1A1b HyK^{339} PSM

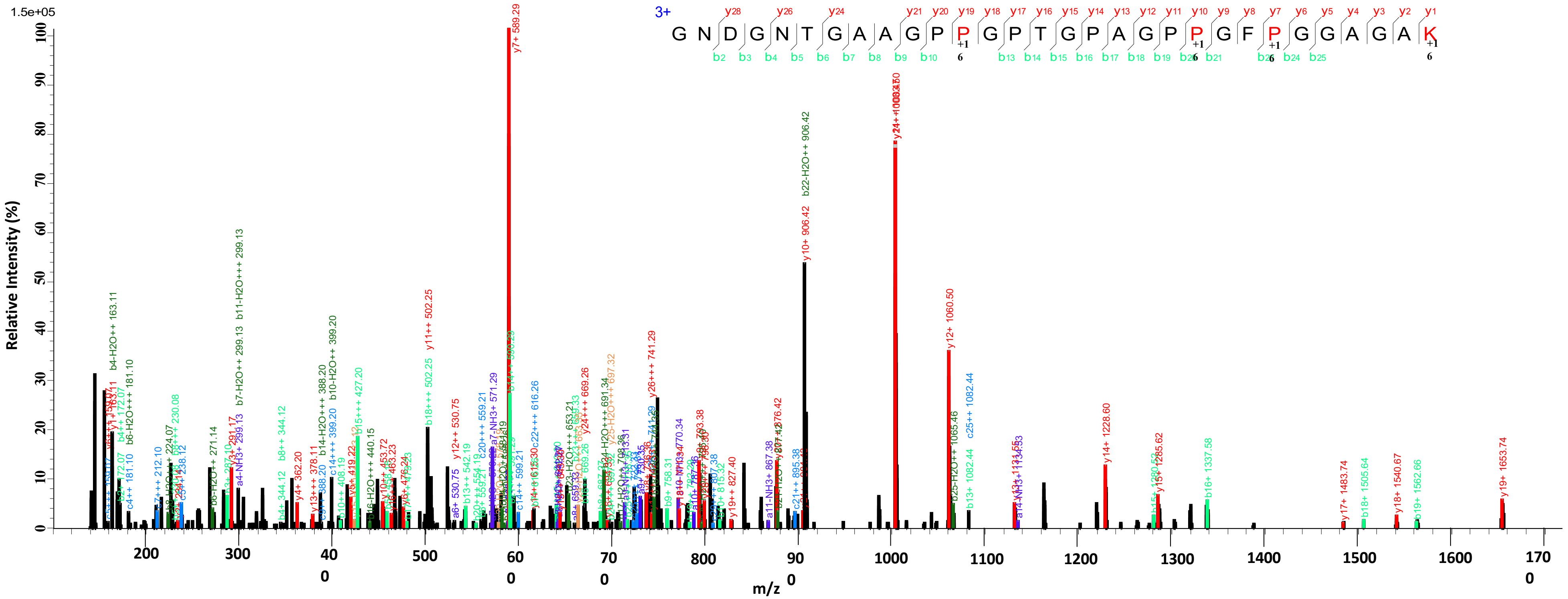

S2.40 m/z= 1215.8705^{+3}

Zebrafish COL1A1b GG-HyK^{339}

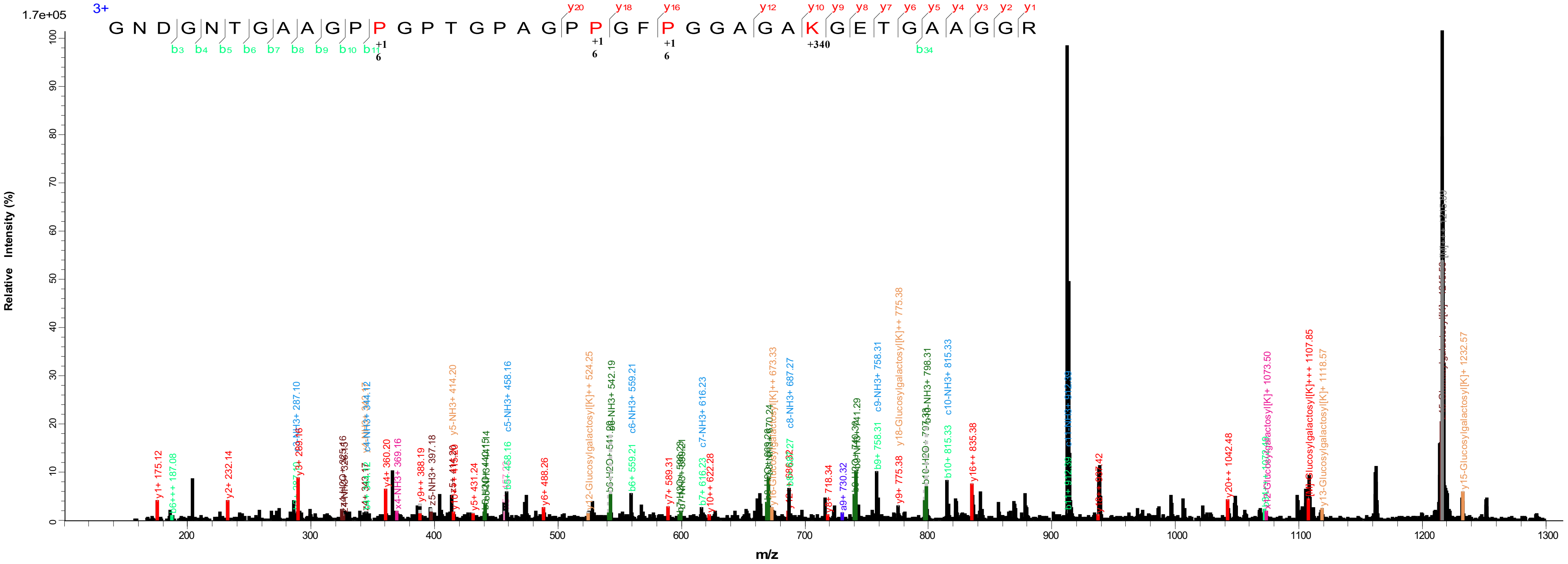

S2.41 m/z= 1133.9917^{+2}

Zebrafish COL1A1b HyK^{384}

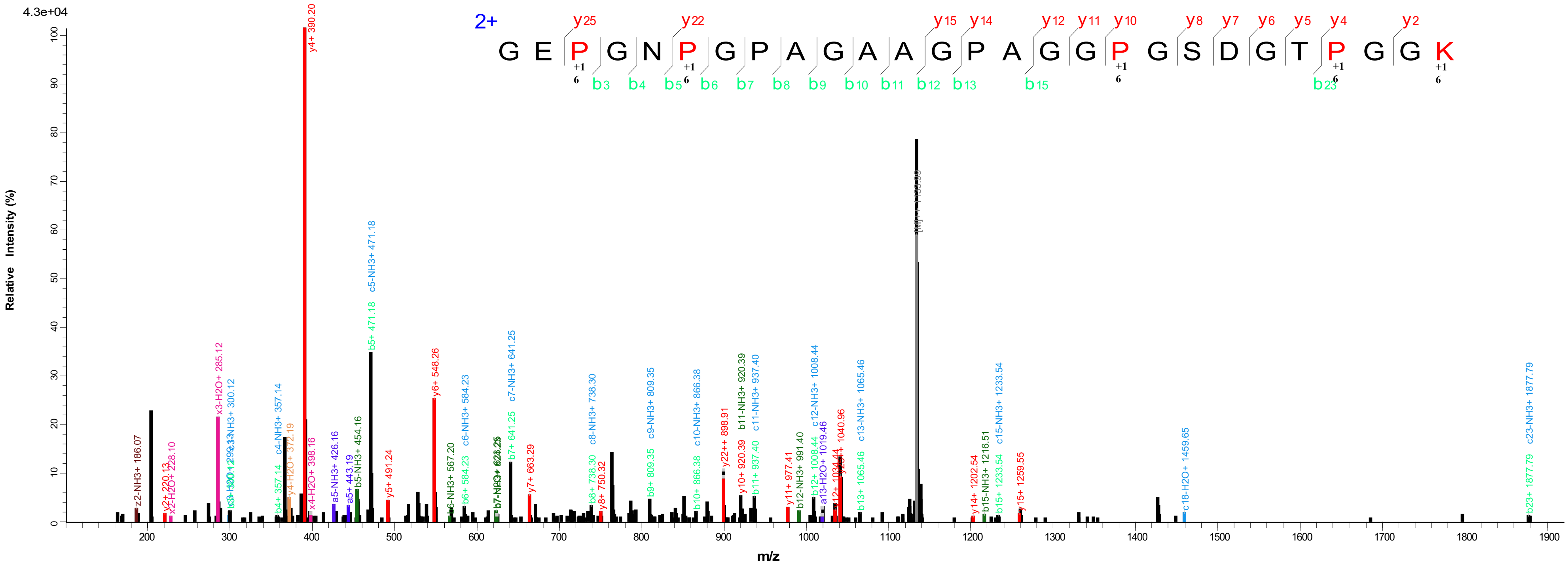

S2.42

m/z= 925.9442^{+4}

Zebrafish COL1A1b HyK^{429}

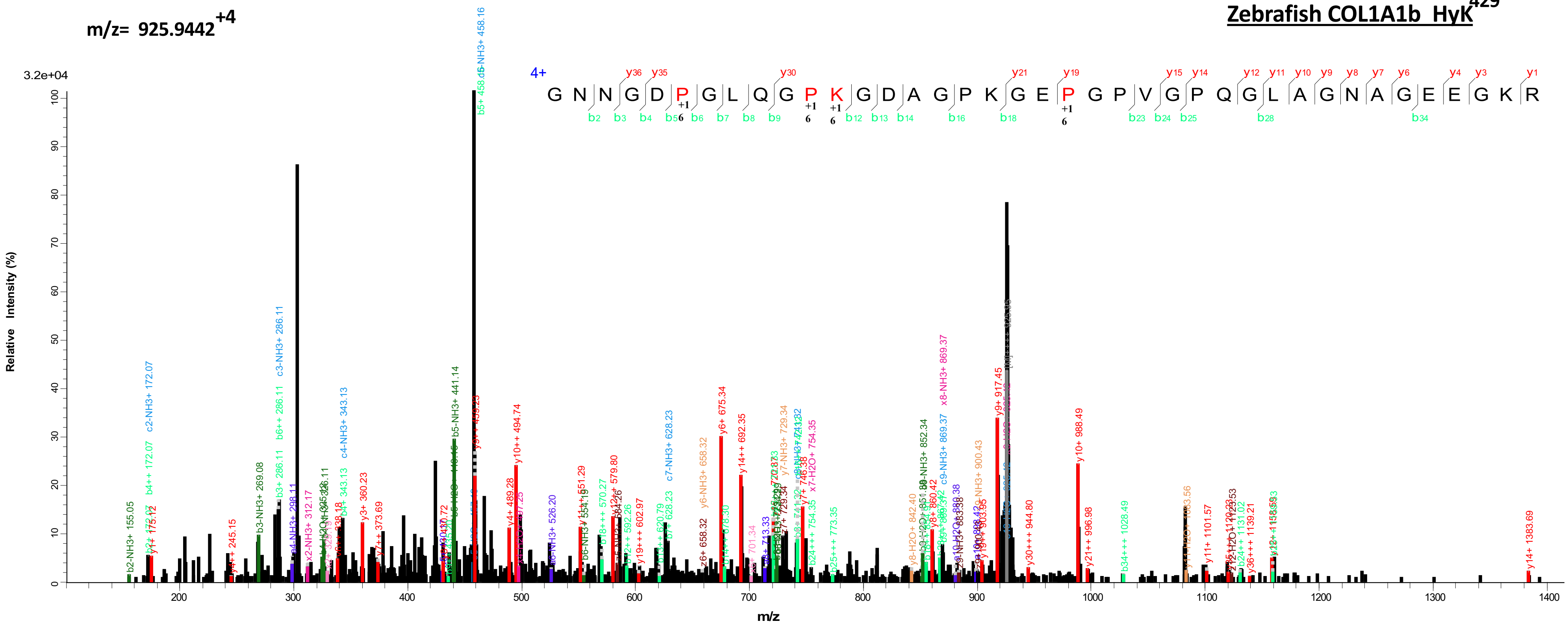

S2.43

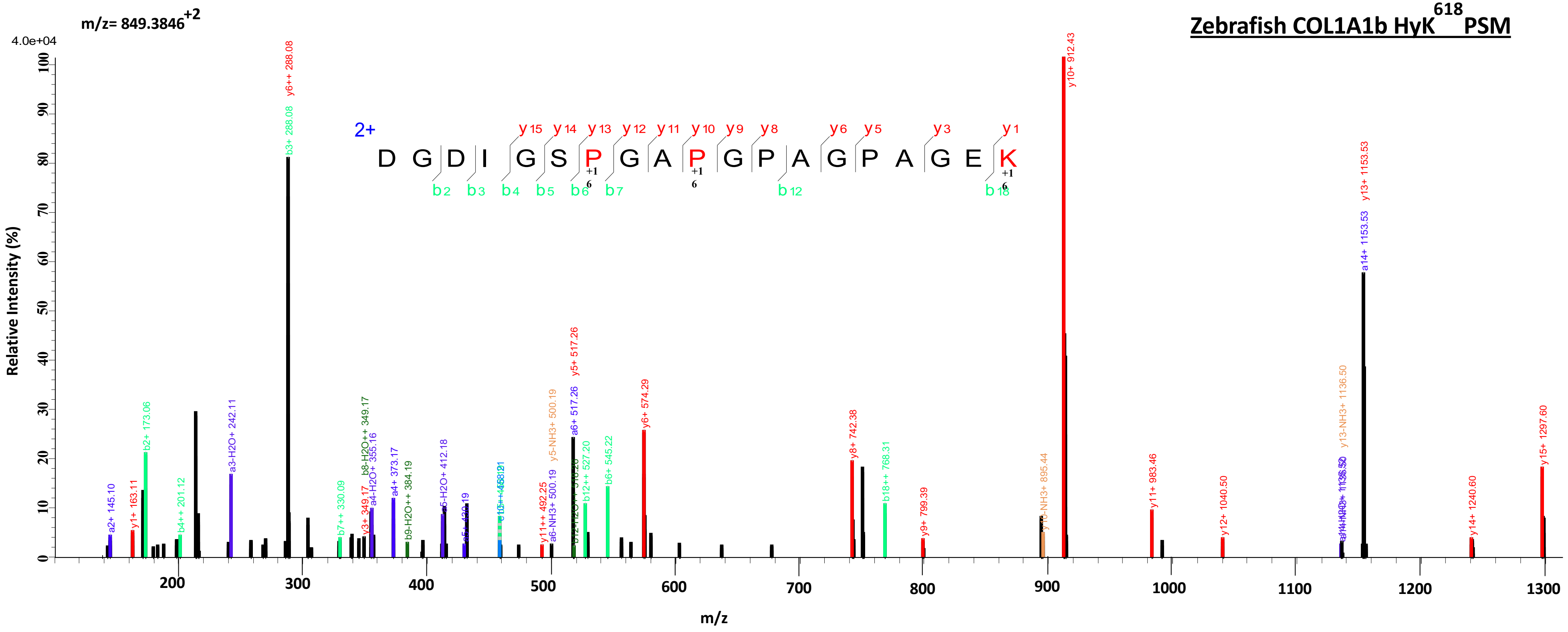
m/z= 849.3846+2
Zebrafish COL1A1b HyK618 PSM
2+
D G D I G S P G A P G P A G P A G E K
Relative Intensity (%)
m/z

S2.44

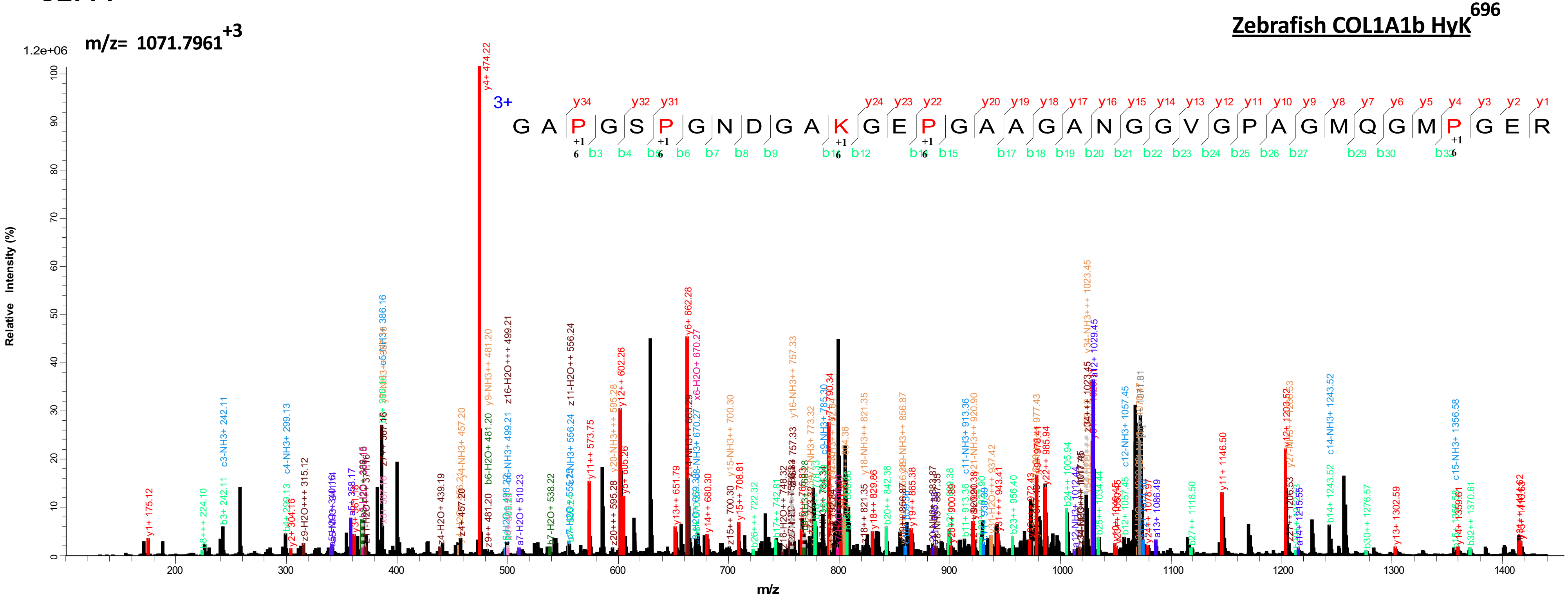

Zebrafish COL1A1b HyK696
m/z= 1071.7961+3
1.2e+06
Relative Intensity (%)
m/z
3+
G A P G S P G N D G A K G E P G A A G A N G G V G P A G M Q G M P G E R

S2.45
m/z= 1126.1471^{+3}

Zebrafish COL1A1b G-HyK^{696}

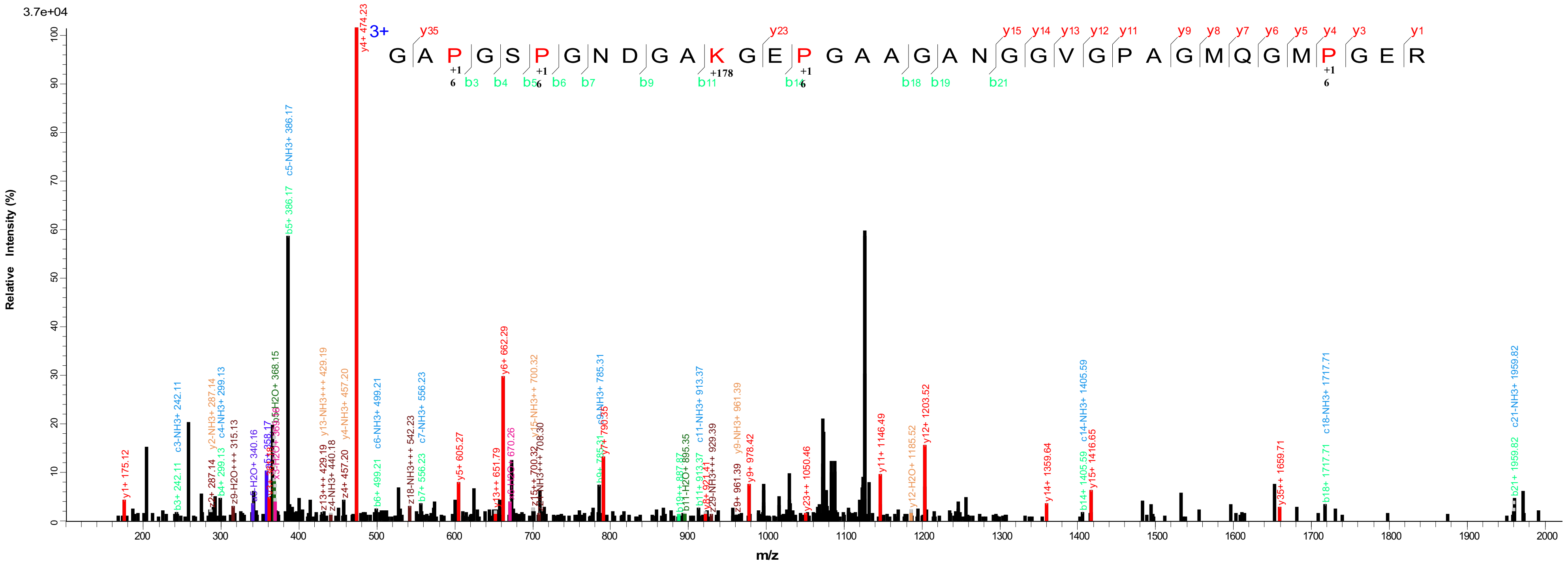

S2.46

m/z= 499.9886 +4

Zebrafish COL1A1b HyK738

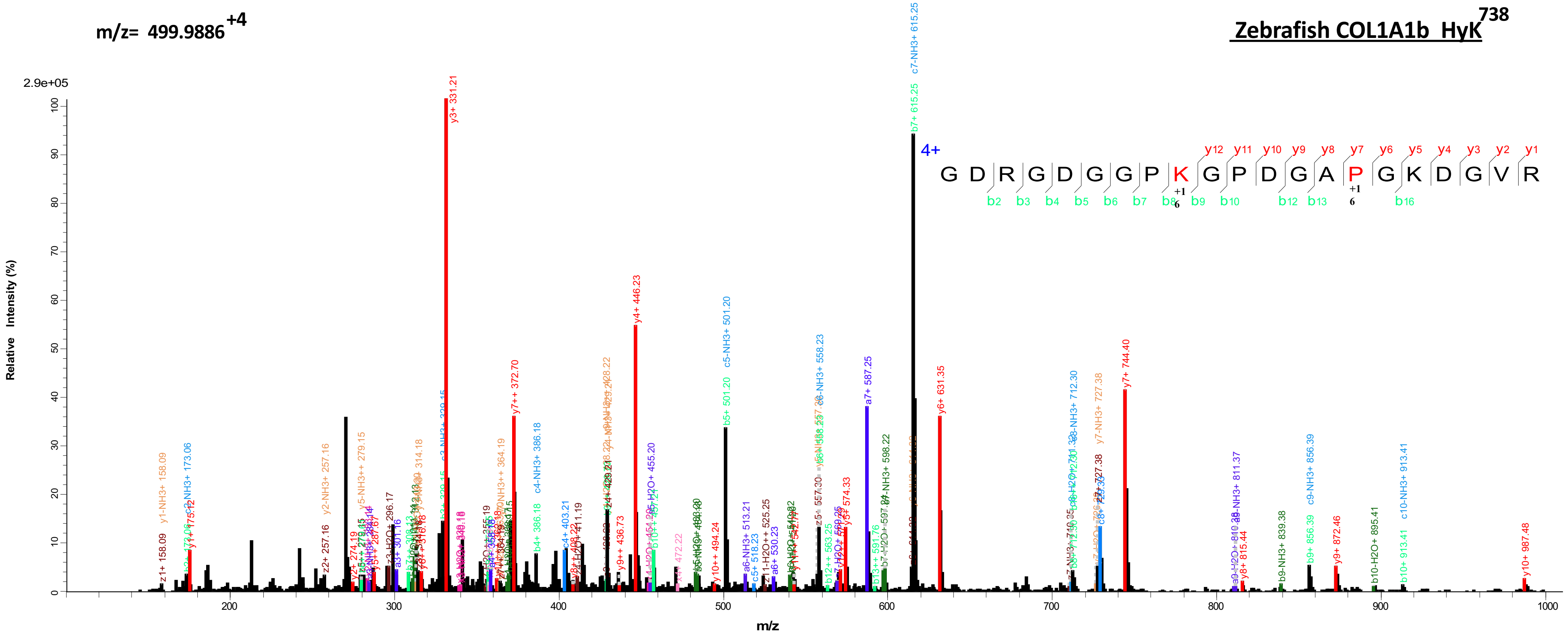

S2.47

m/z= 820.8835^{+2}

Zebrafish COL1A1b HyK^{768} PSM

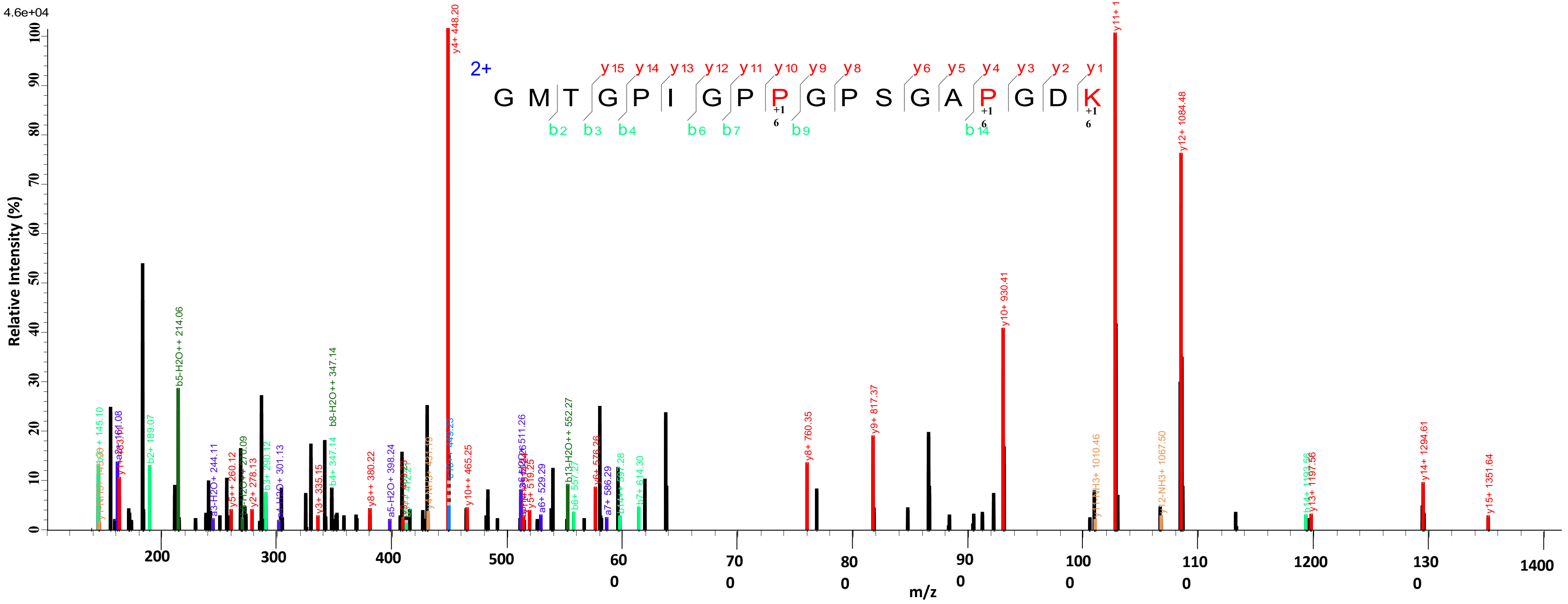

S2.48

m/z= 877.1677 $^{+4}$

Zebrafish COL1A1b G-HyK[822]

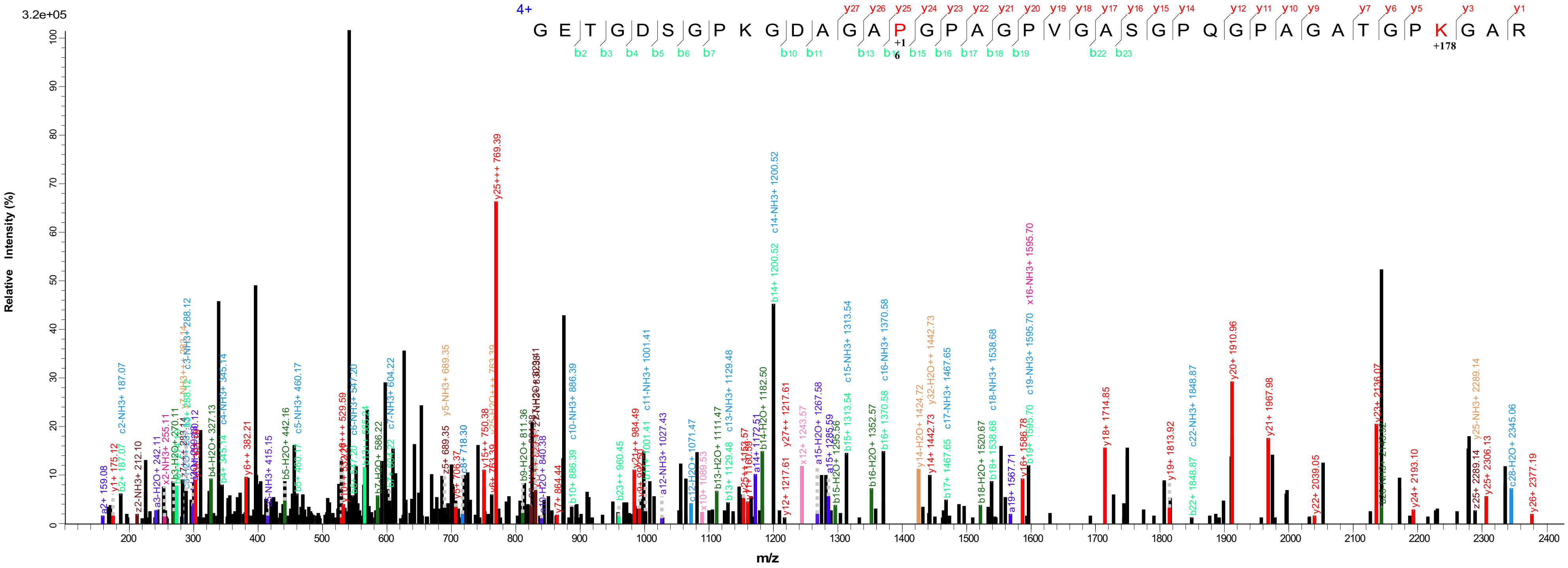

S2.49

m/z= 921.6785 +4

Zebrafish COL1A1b GG-HyK849

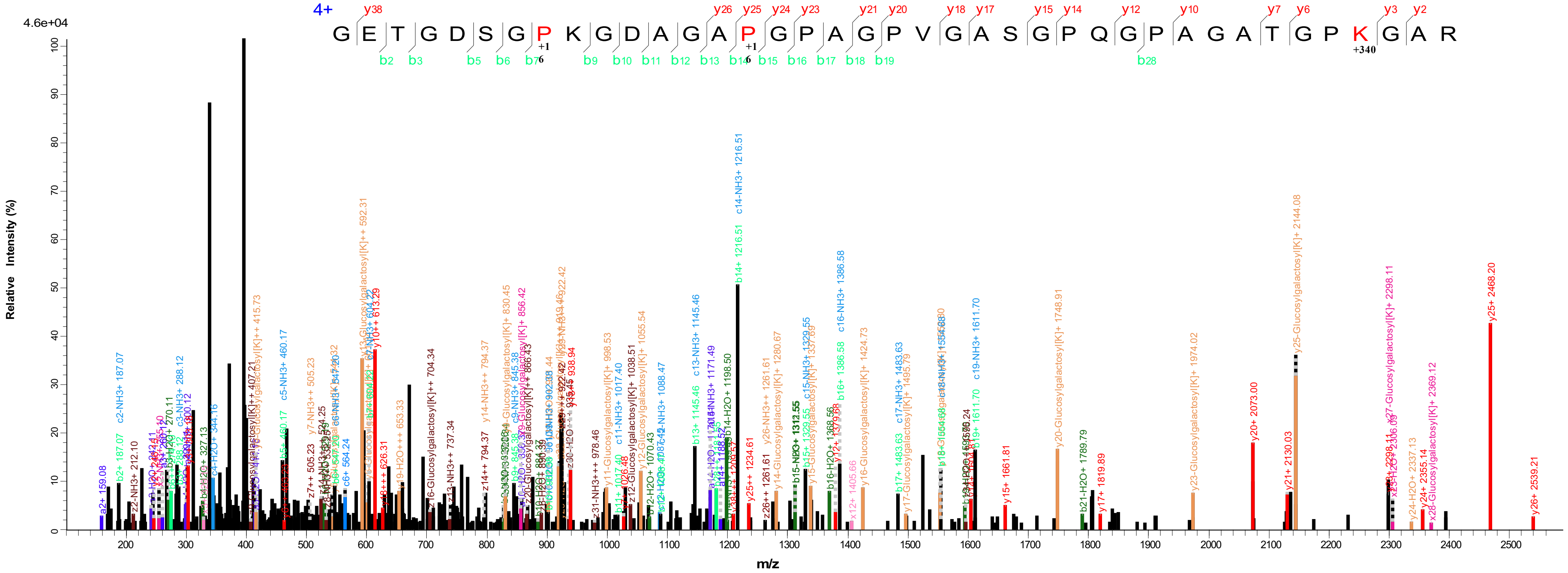

S2.50

m/z= 552.5163 $^{+4}$

Zebrafish COL1A1b HyK^{900}

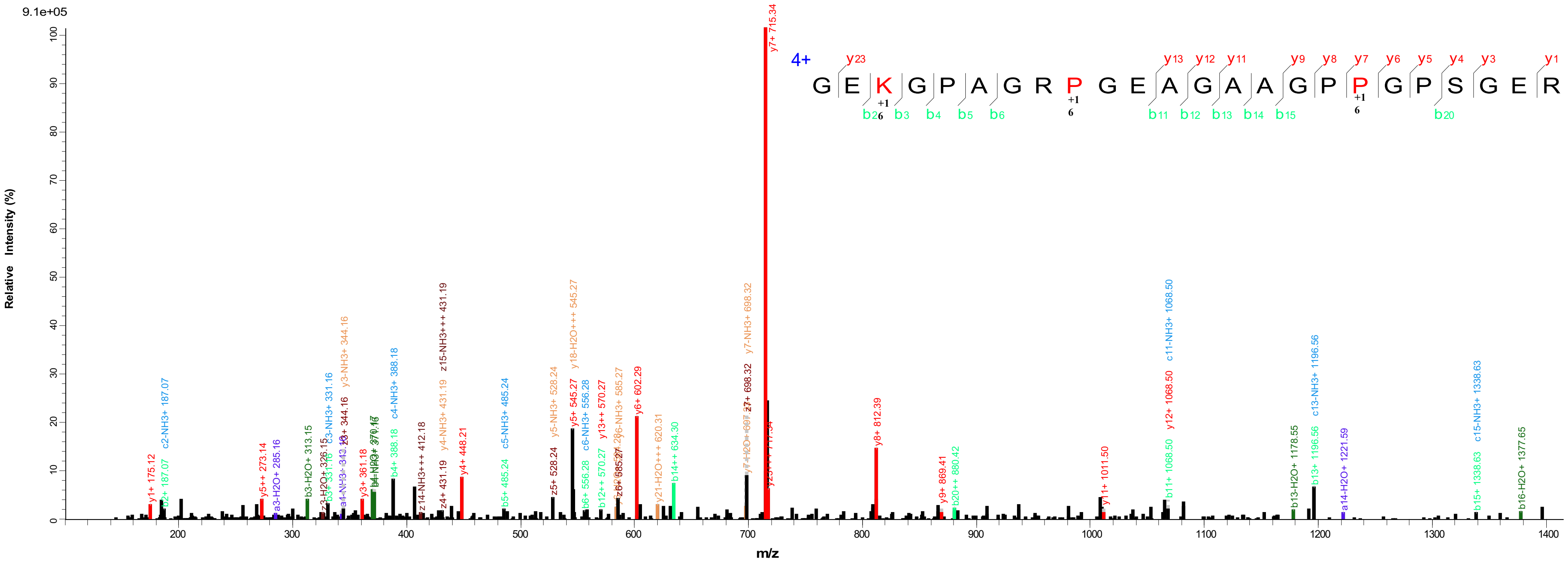

S2.51

m/z= 815.3836 $^{+4}$

Zebrafish COL1A1b HyK[1020] **3-HyP**[1031]

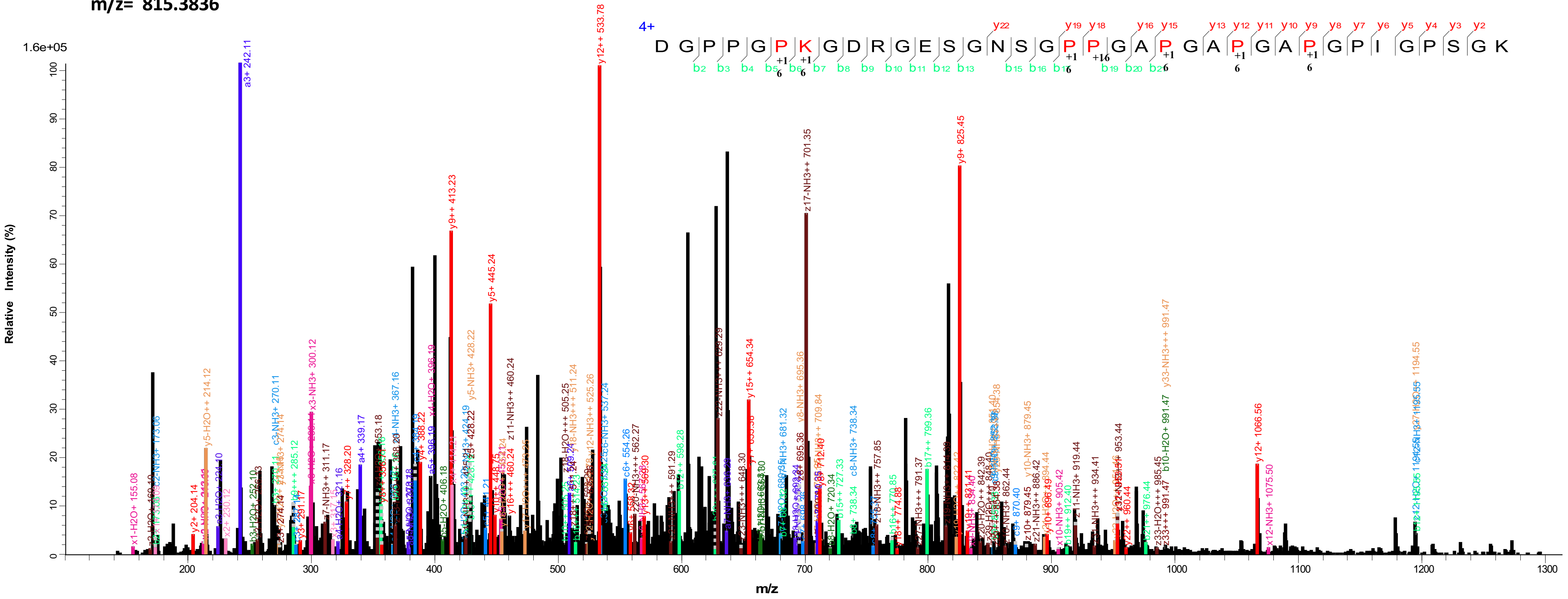

S2.52 m/z= 851.8932 +4

Zebrafish COL1A1b G-HyK 1020

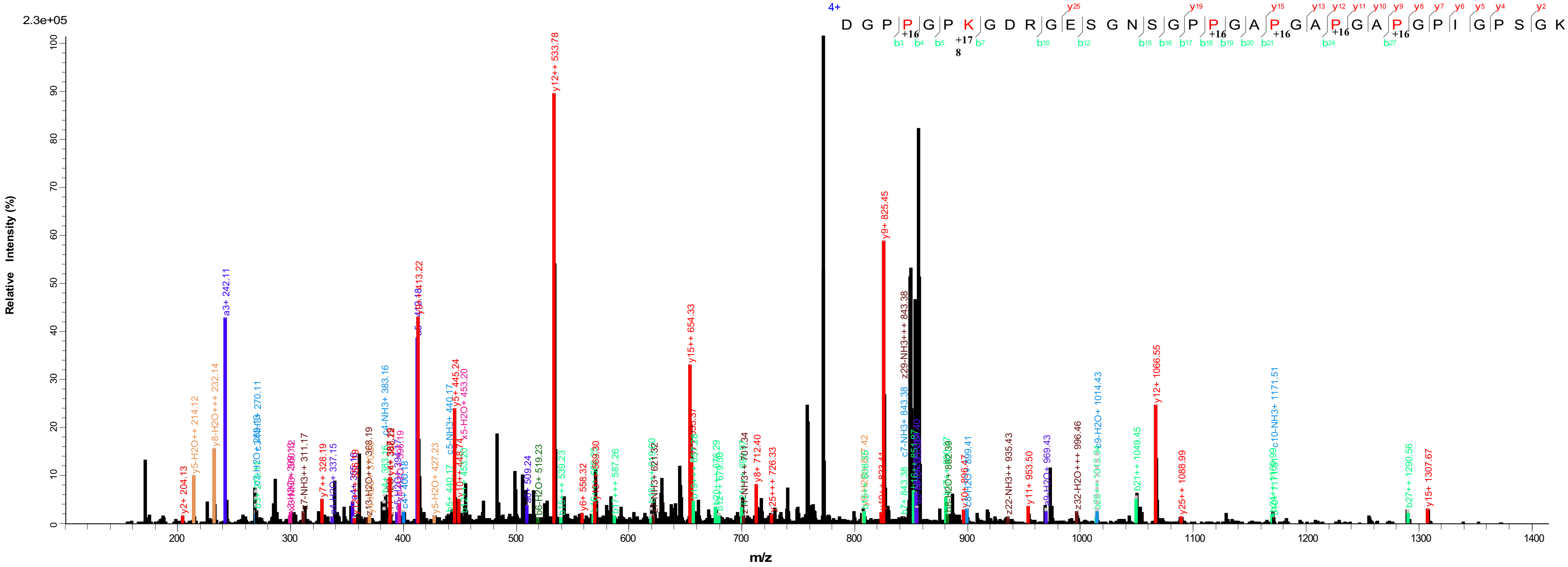

S2.53 m/z=639.5416 +4

Zebrafish COL1A2 3-HyP$^{36}_{1}$ HyK^{350}

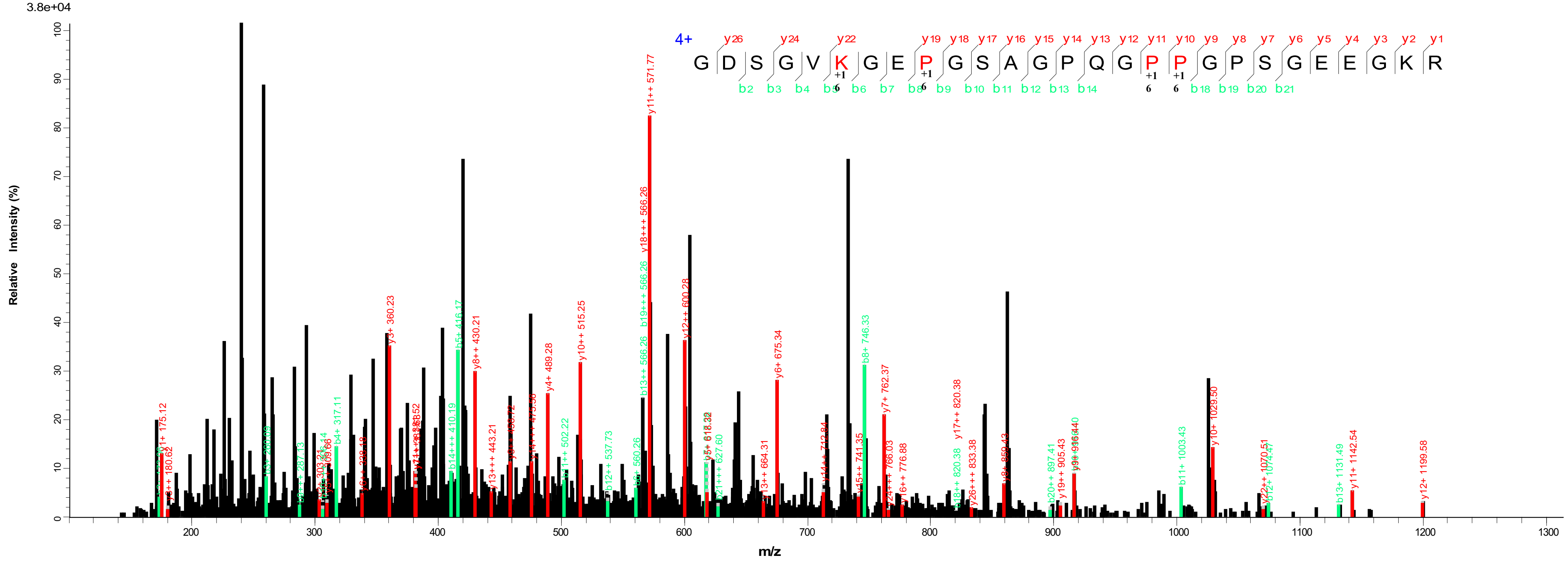

S2.54 m/z= 781.0231+3

Zebrafish COL1A2 3-HyP451

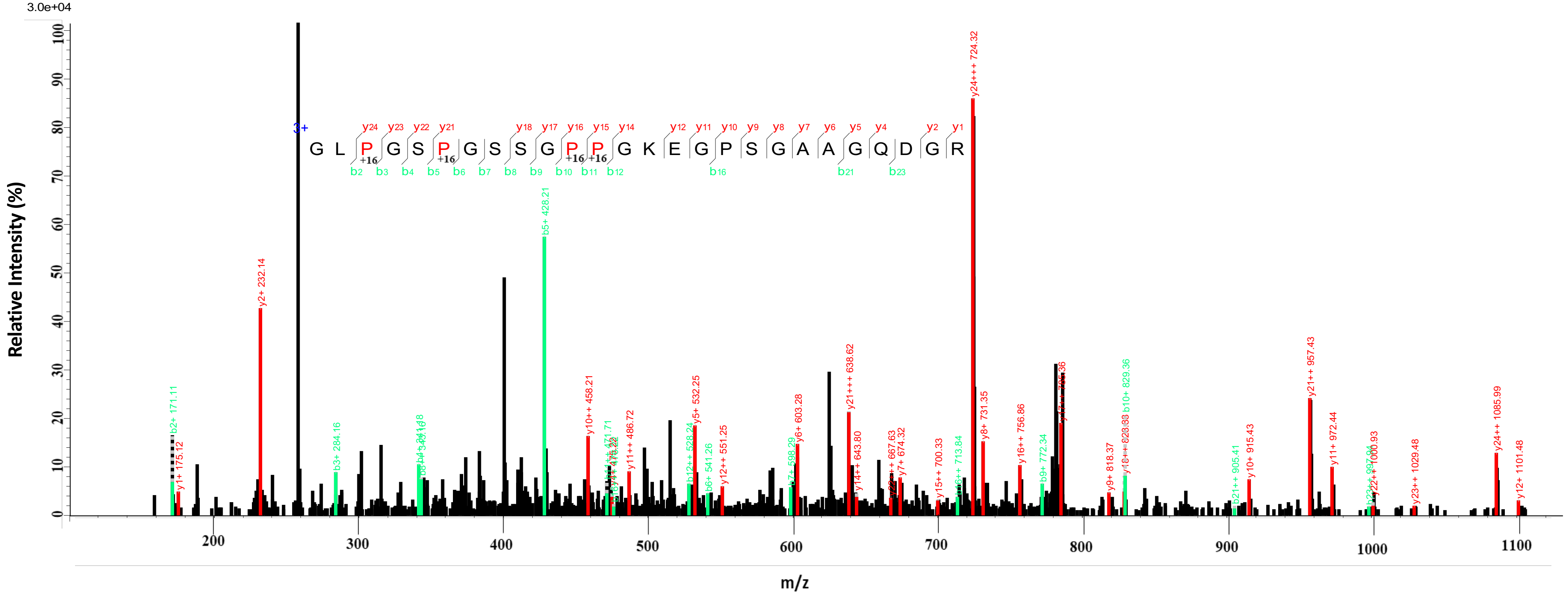

S2.55 m/z= 484.7433^{+2}

469

Zebrafish COL1A2 3-HyP

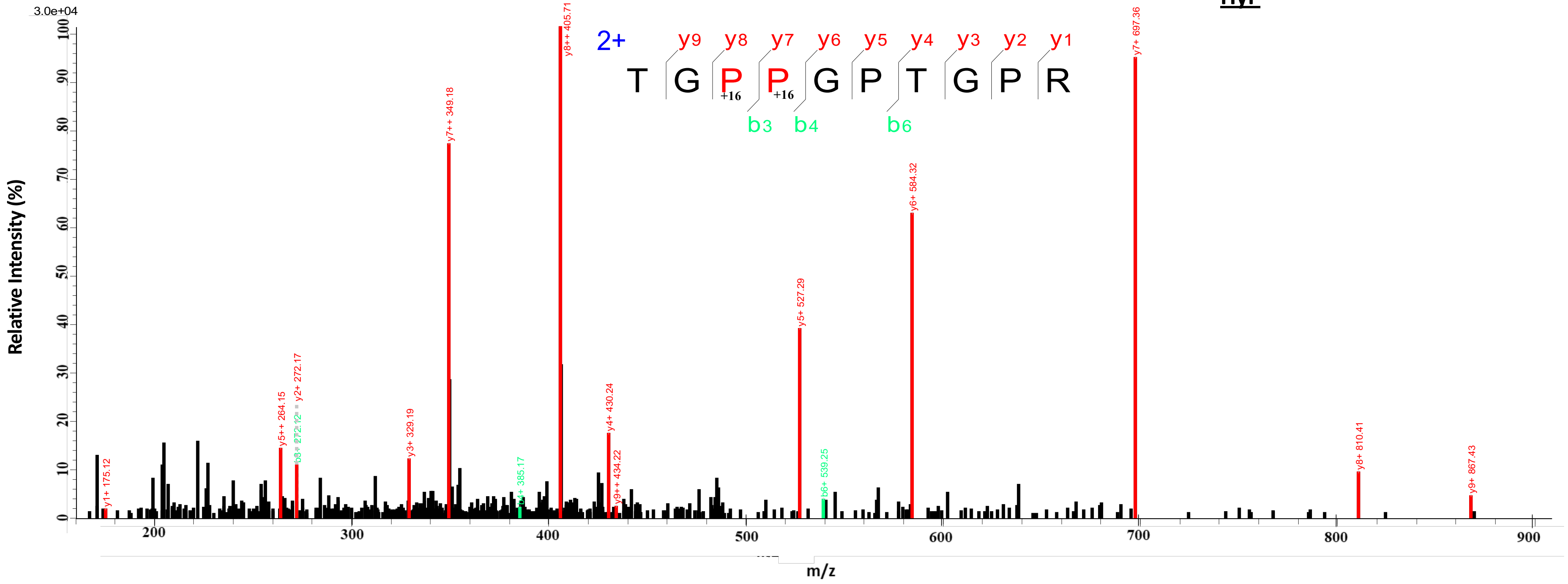

S2.56

m/z=733.8363 +4

Zebrafish COL1A2 3-HyP 673

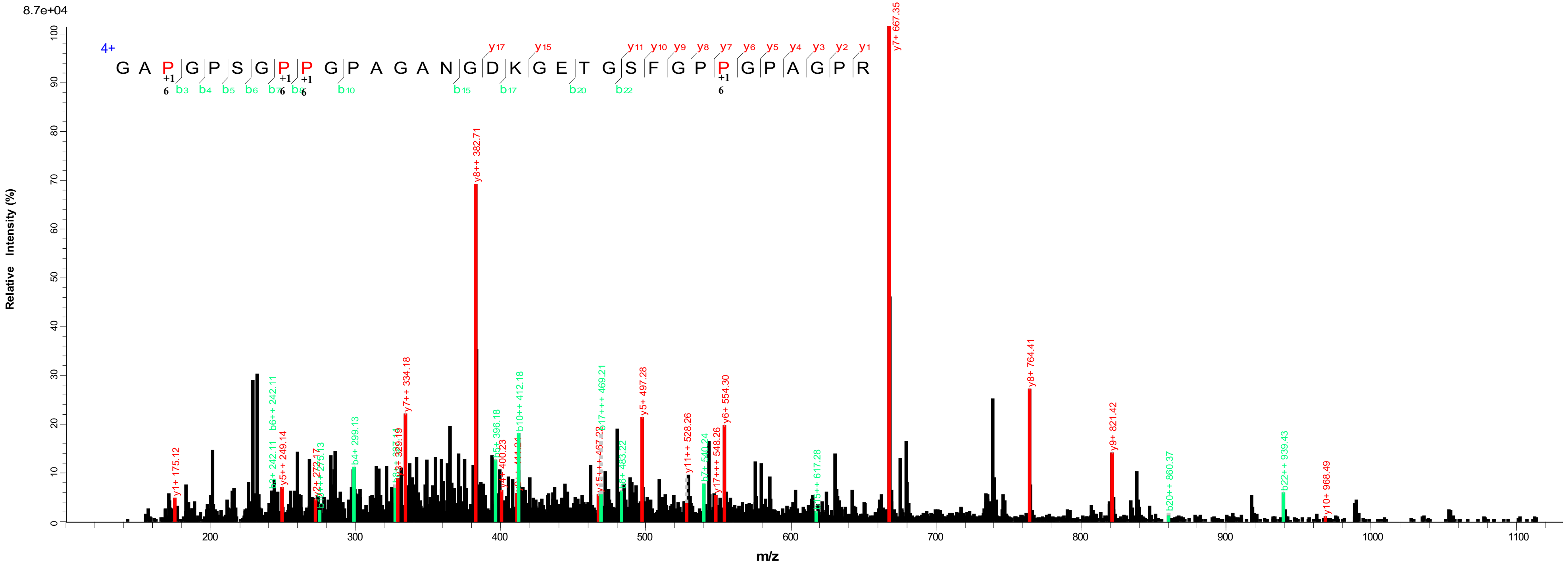

S2.57 m/z=1078.4793^{+2}

Zebrafish COL1A2 3-HyP^{718}

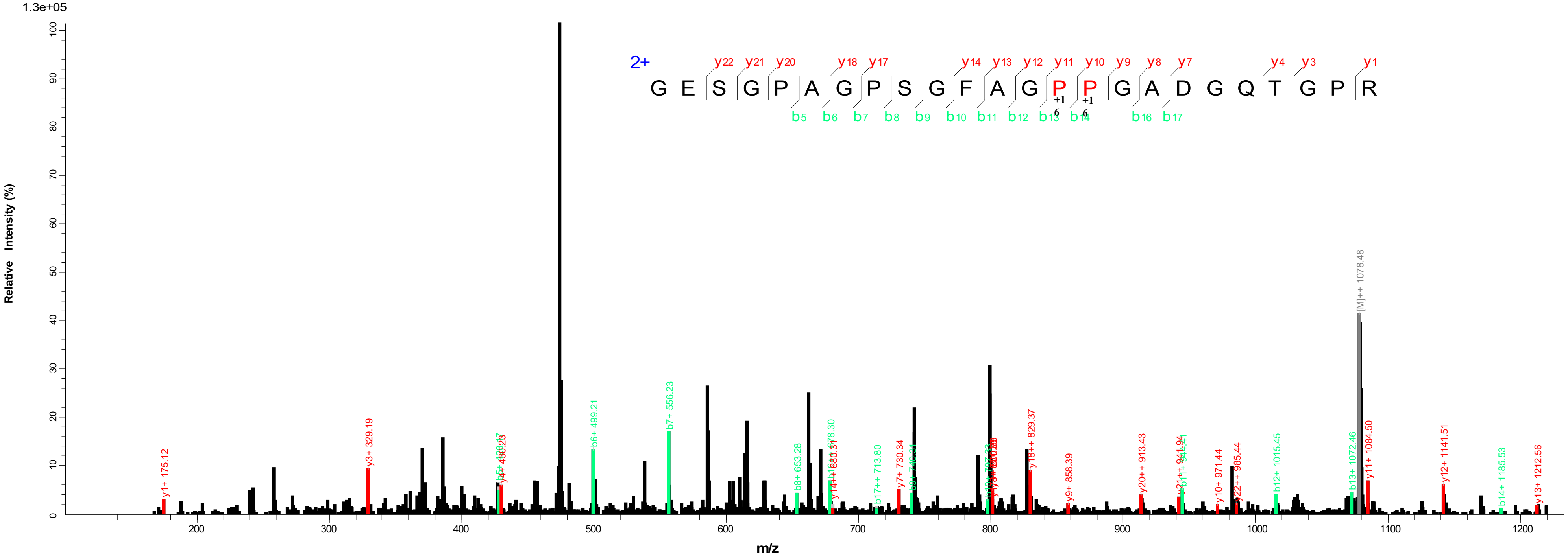

S2.58 m/z=901.4944^{+2}

Zebrafish COL1A2 3-HyP[787] PSM

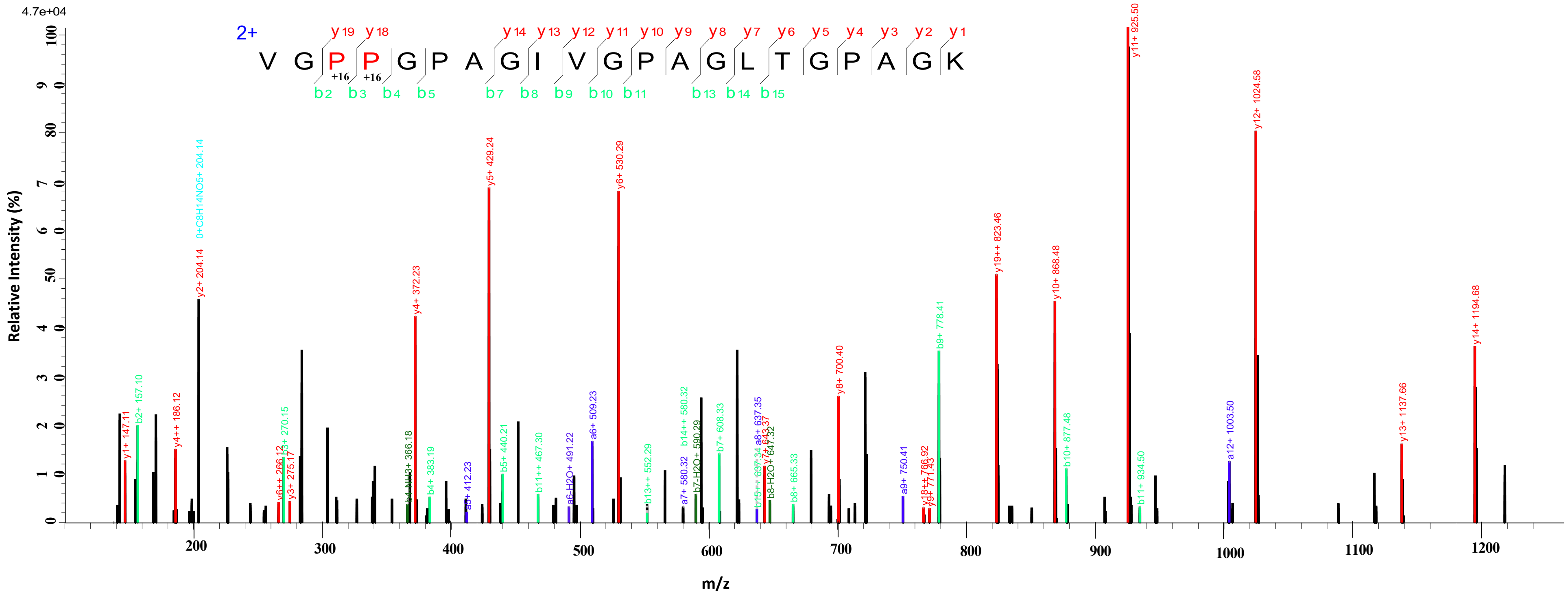

S2.59 m/z=741.3548^{+3}

Zebrafish COL1A2 3-HyP^{820} PSM

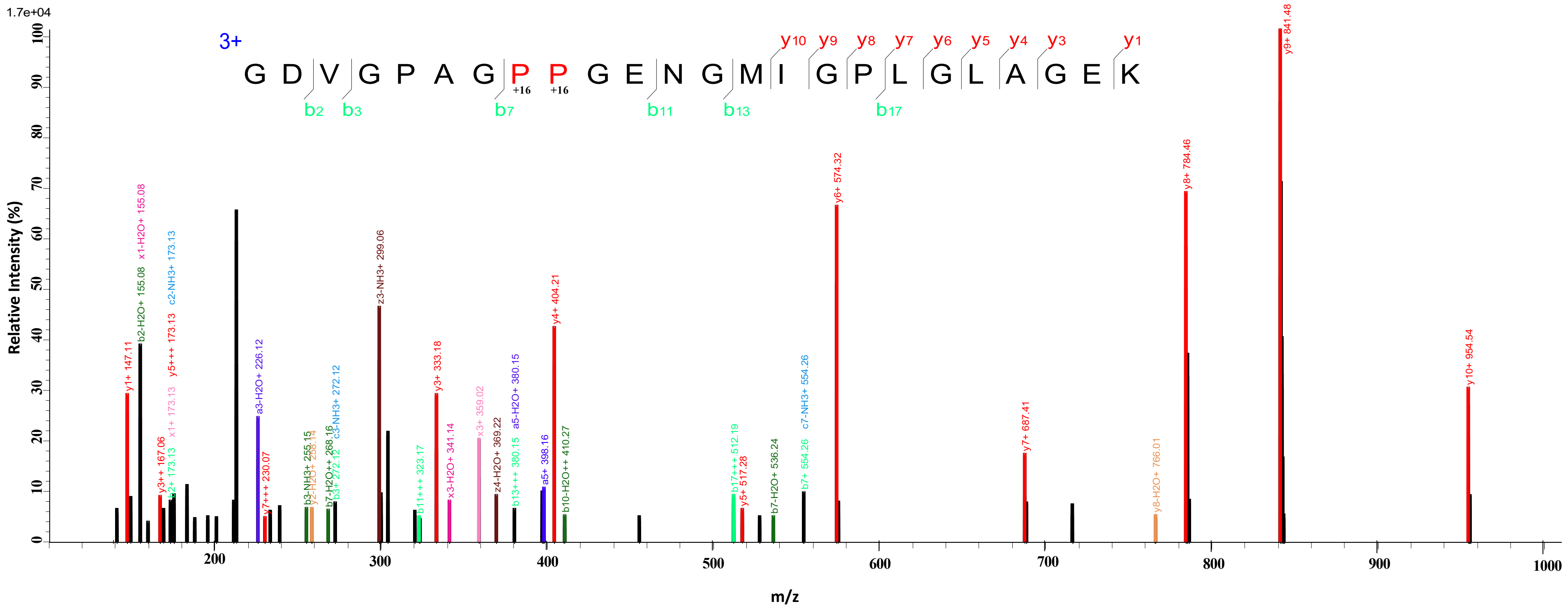

S2.60

m/z=741.3548 +3

Zebrafish COL1A2 3-HyP838 PSM

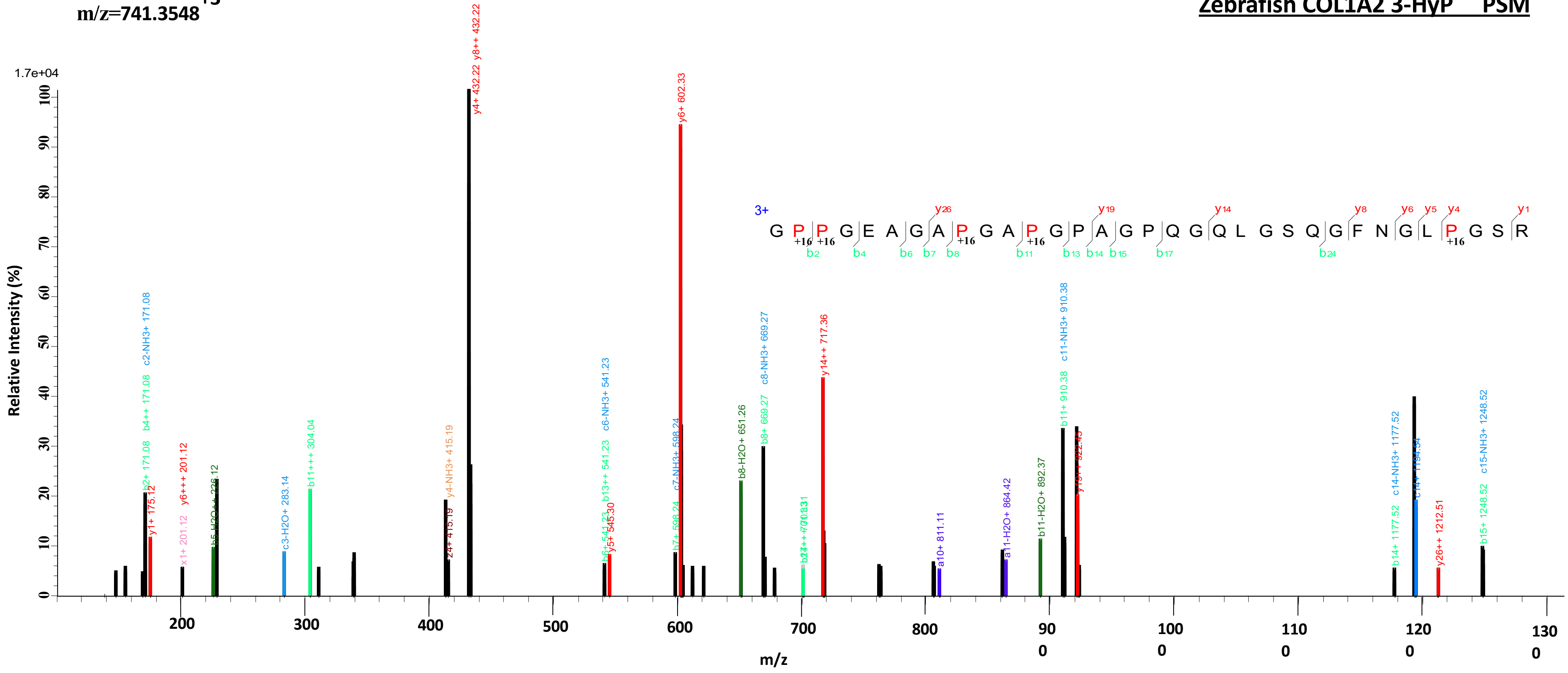

S2.61

m/z= 895.4399^{+2}

Zebrafish COL1A1b 3-HyP[872] PSM

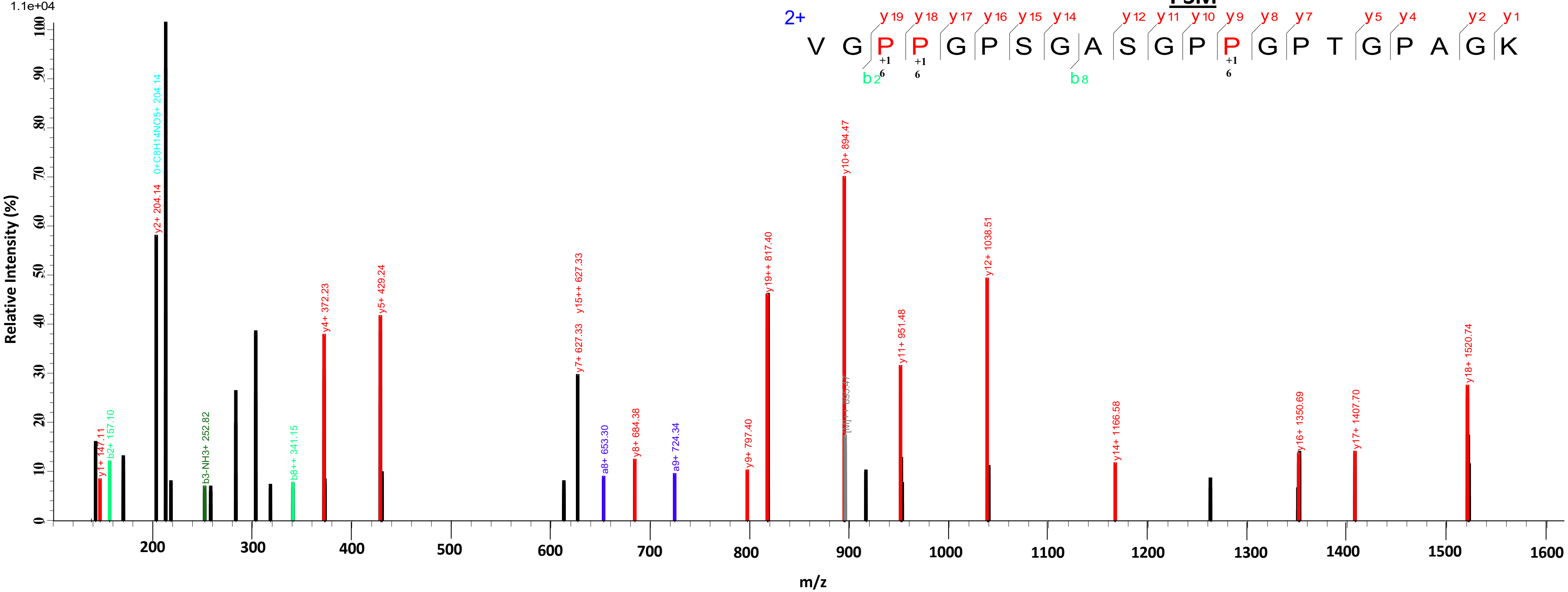

S2.62

m/z= 523.2359^{+3}

Zebrafish COL1A2 3-HyP^{1066}

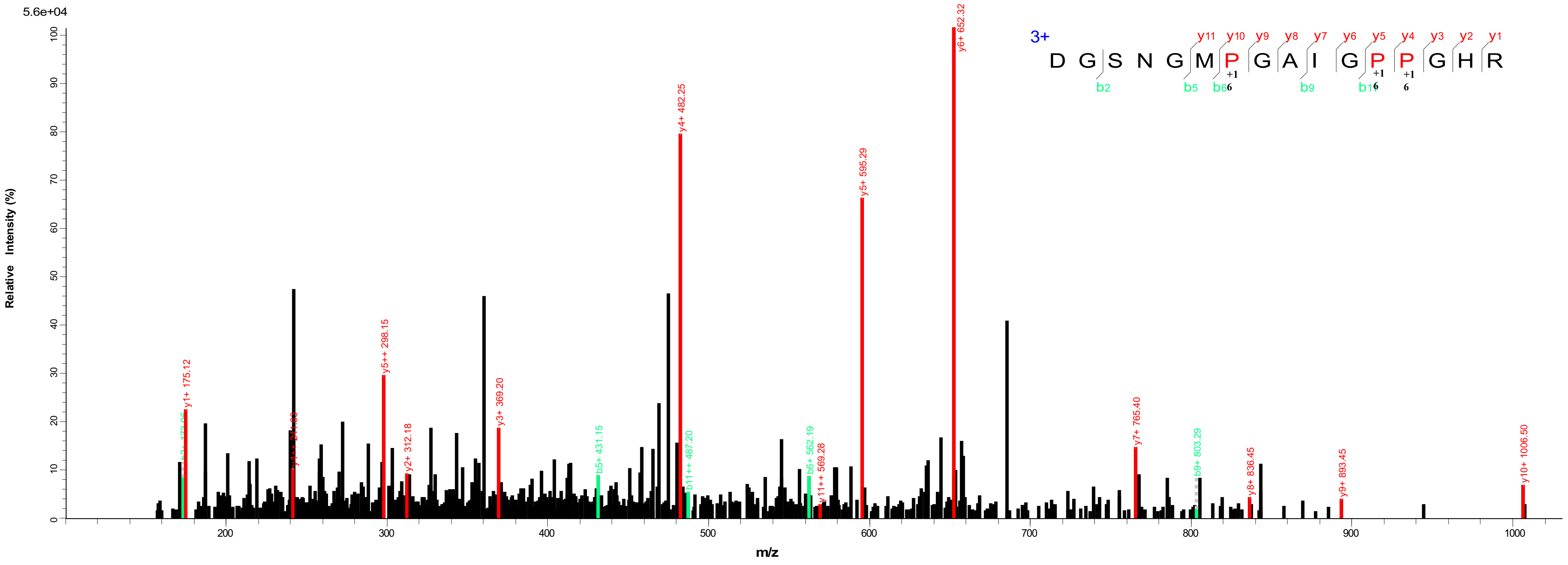

S2.63 m/z= 768.3572+3

Zebrafish COL1A2 HyK 179 & 188

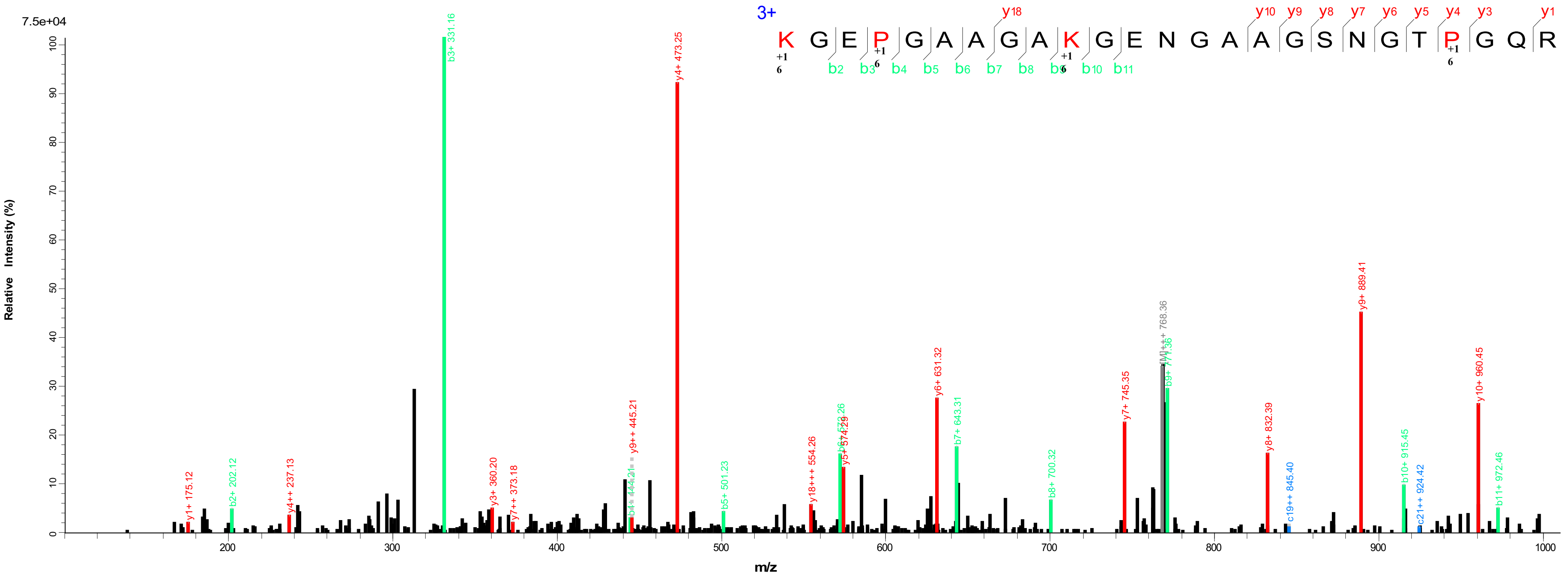

S2.64

m/z= 769.0211^{+3}

Zebrafish COL1A2 G-HyK^{188}

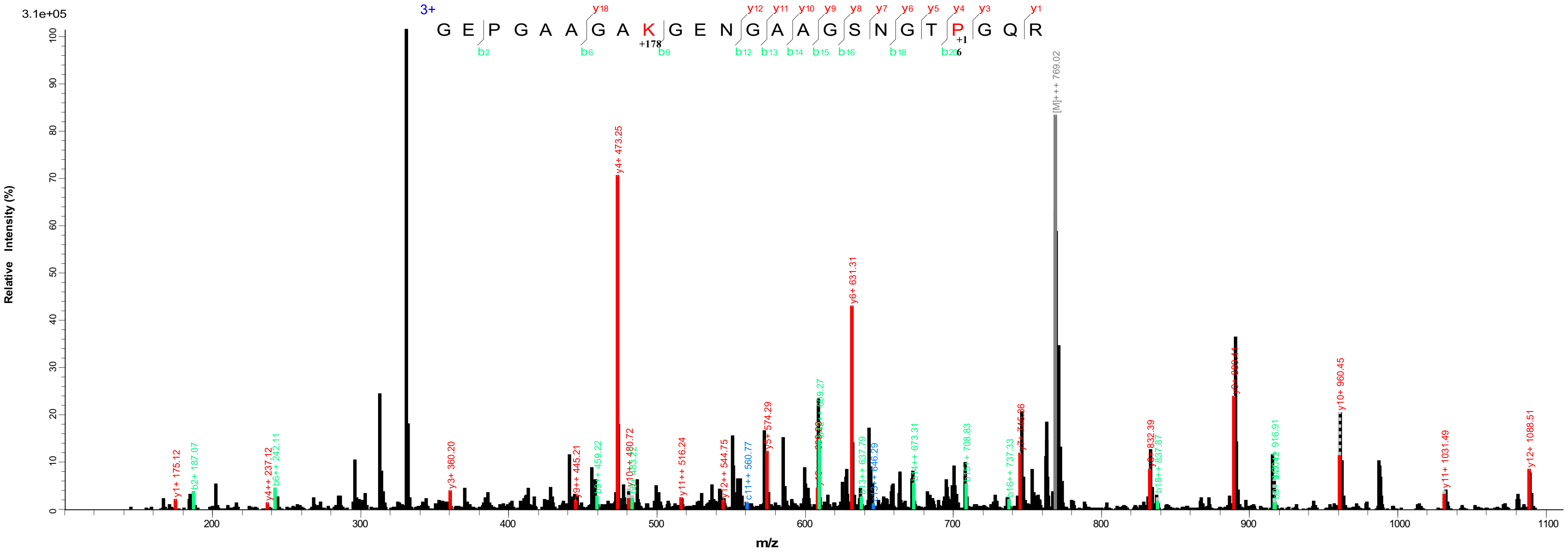

S2.65 m/z= 653.5395^{+4}

Zebrafish COL1A2 GG-HyK^{188}

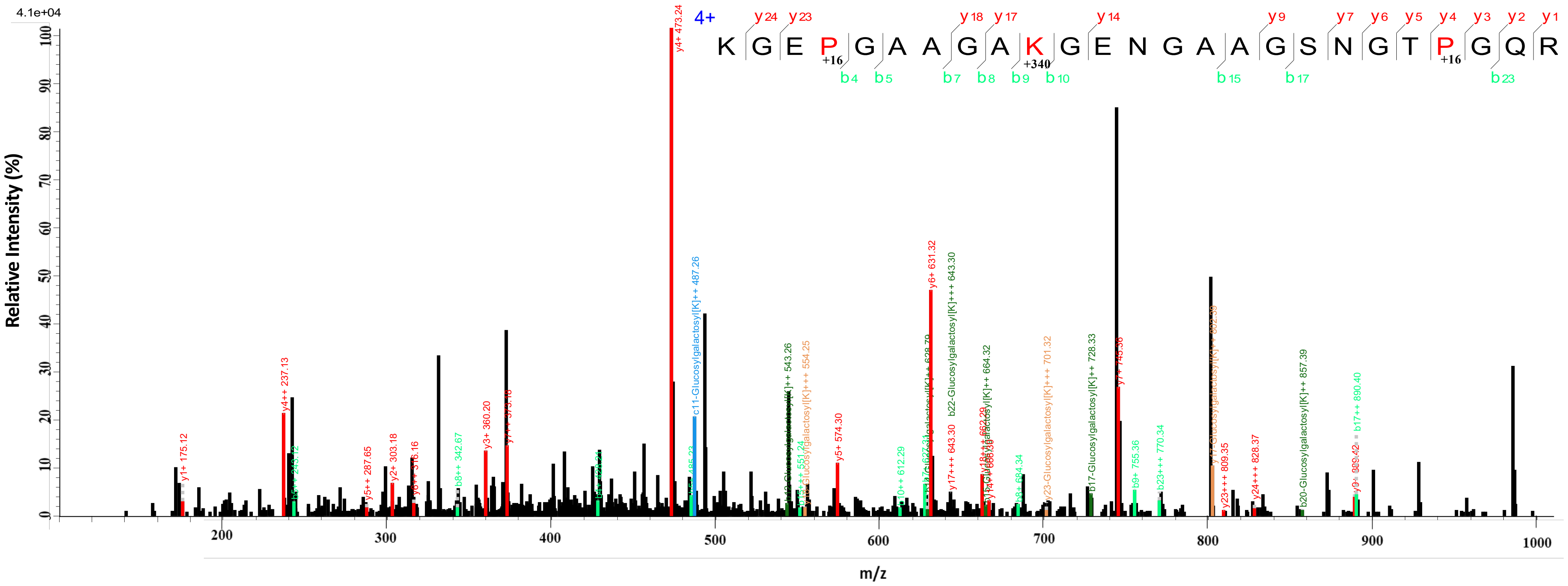

S2.66

m/z=864.0786^{+3}

Zebrafish COL1A2 HyK[254] PSM

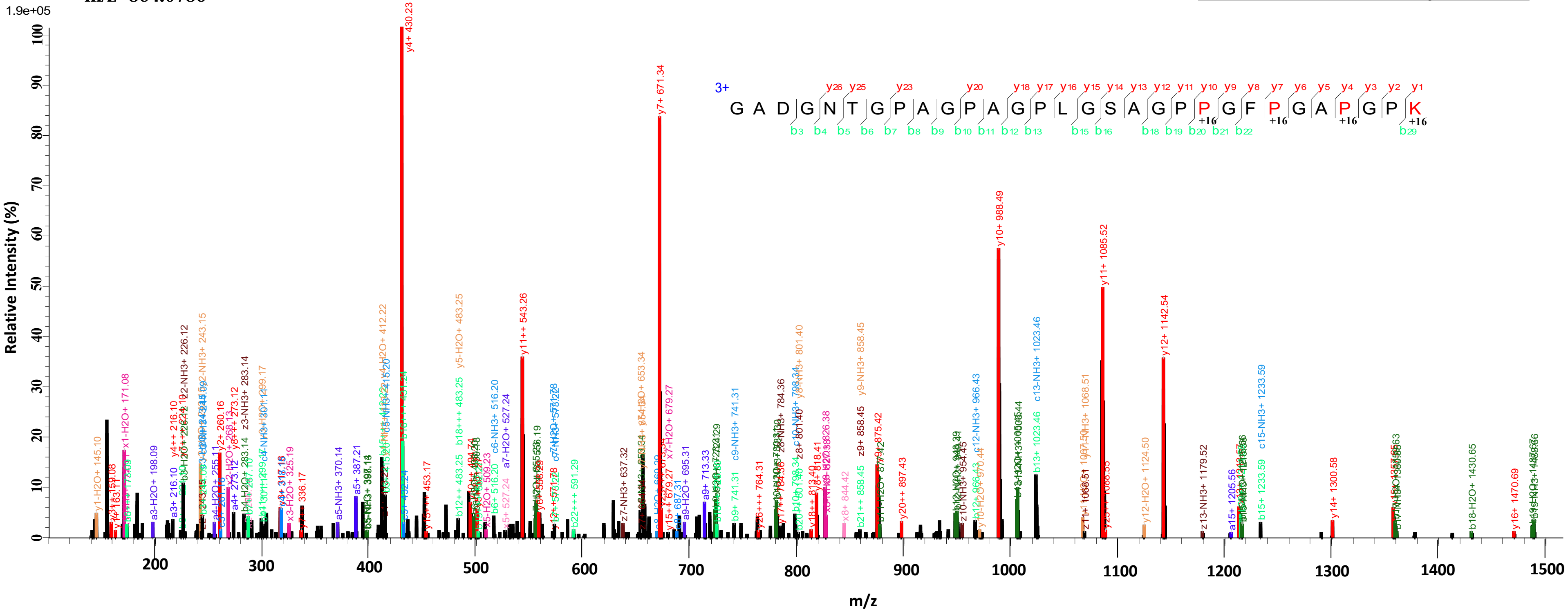

S2.67 m/z= 1056.7590+4

Zebrafish COL1A2 3-HyP244 HyK254

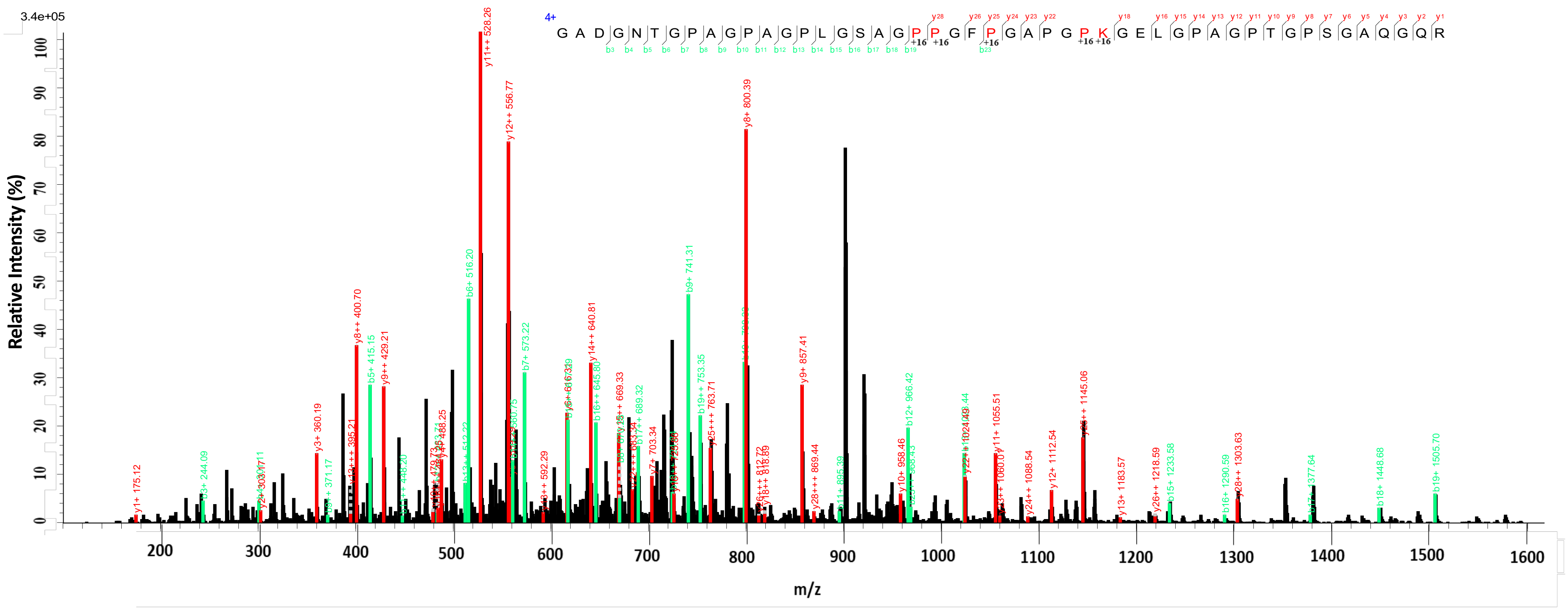

S2.68 m/z= 1093.2728 +4

Zebrafish COL1A2 G-HyK 254

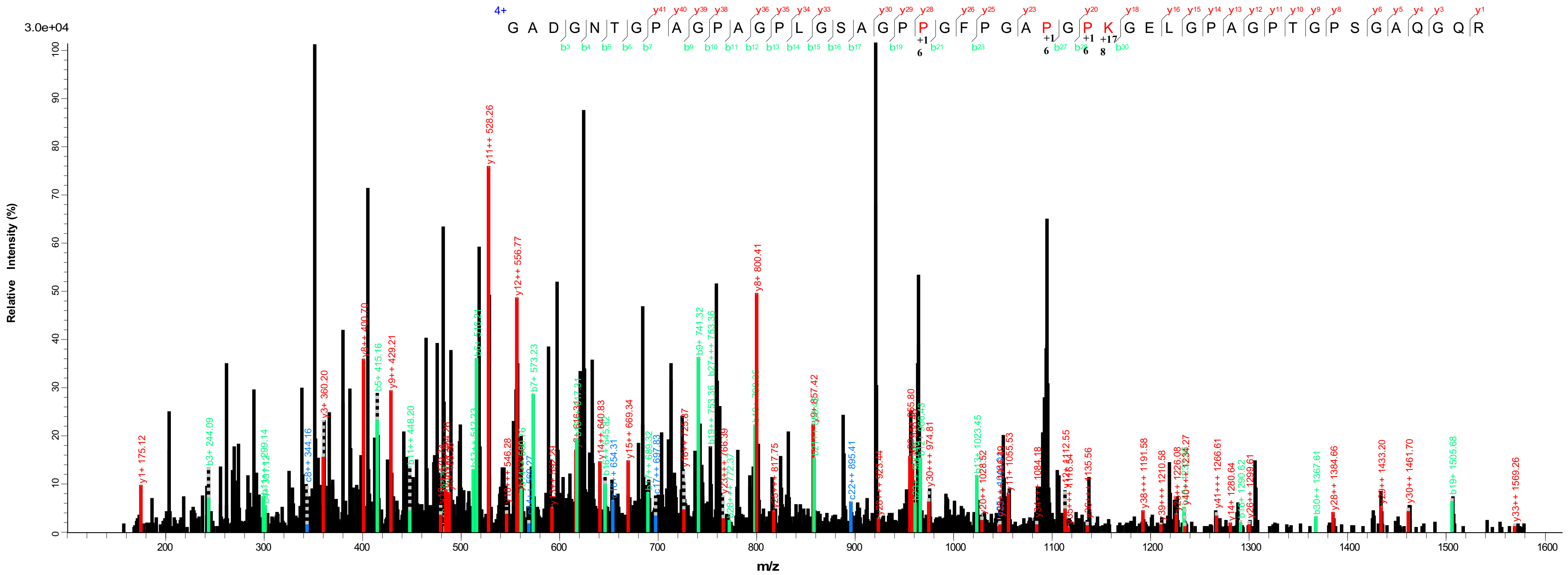

S2.69 m/z= 1133.7892 $^{+4}$

Zebrafish COL1A2 GG-HyK ^{254}

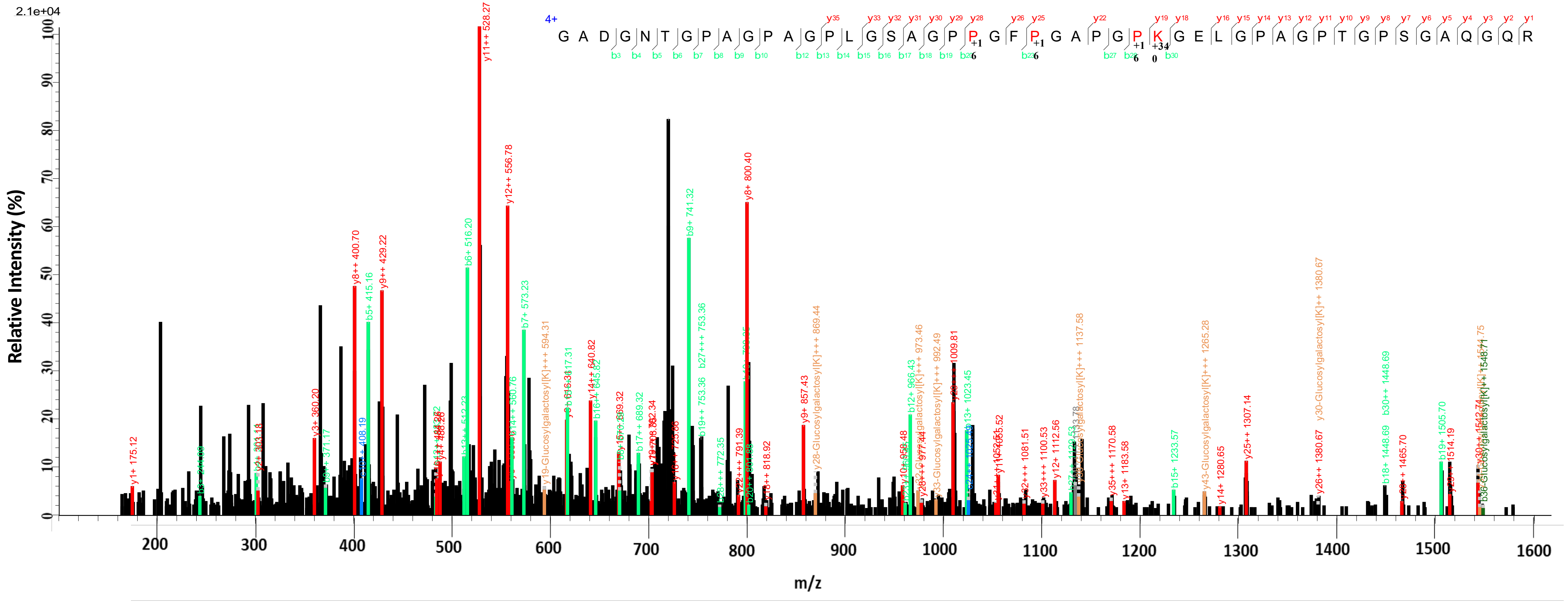

S2.70

m/z= 901.9349^{+4}

Zebrafish COL1A2 HyK $^{344 \& 350}$

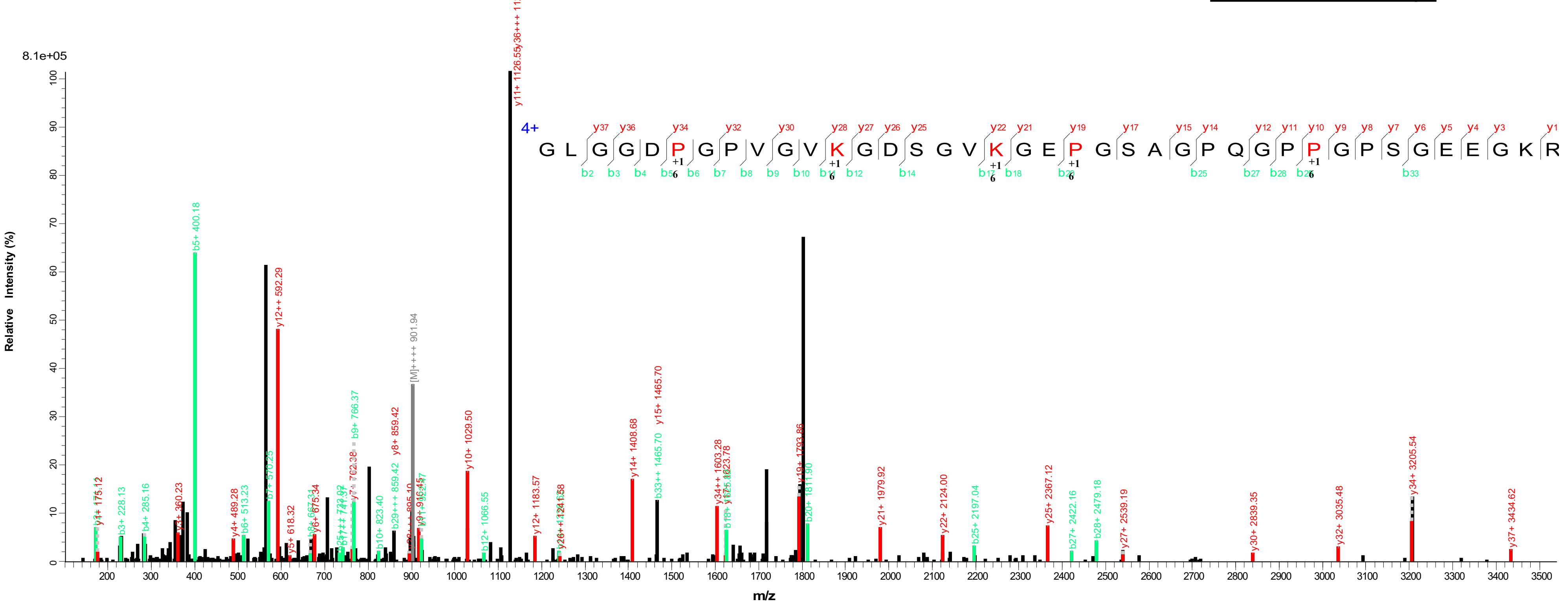

S2.71

m/z= 781.0248 +3

Zebrafish COL1A2 HyK 454

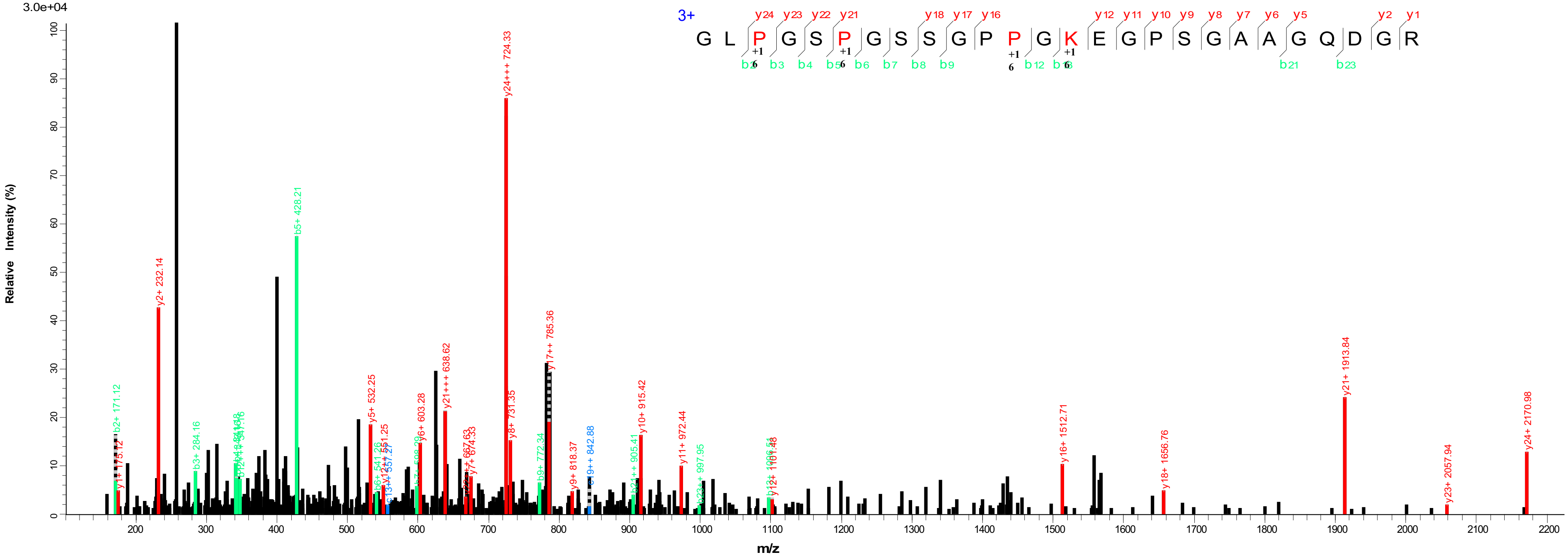

S2.72

m/z= 608.8016 +2

Zebrafish COL1A2 HyK[488] PSM

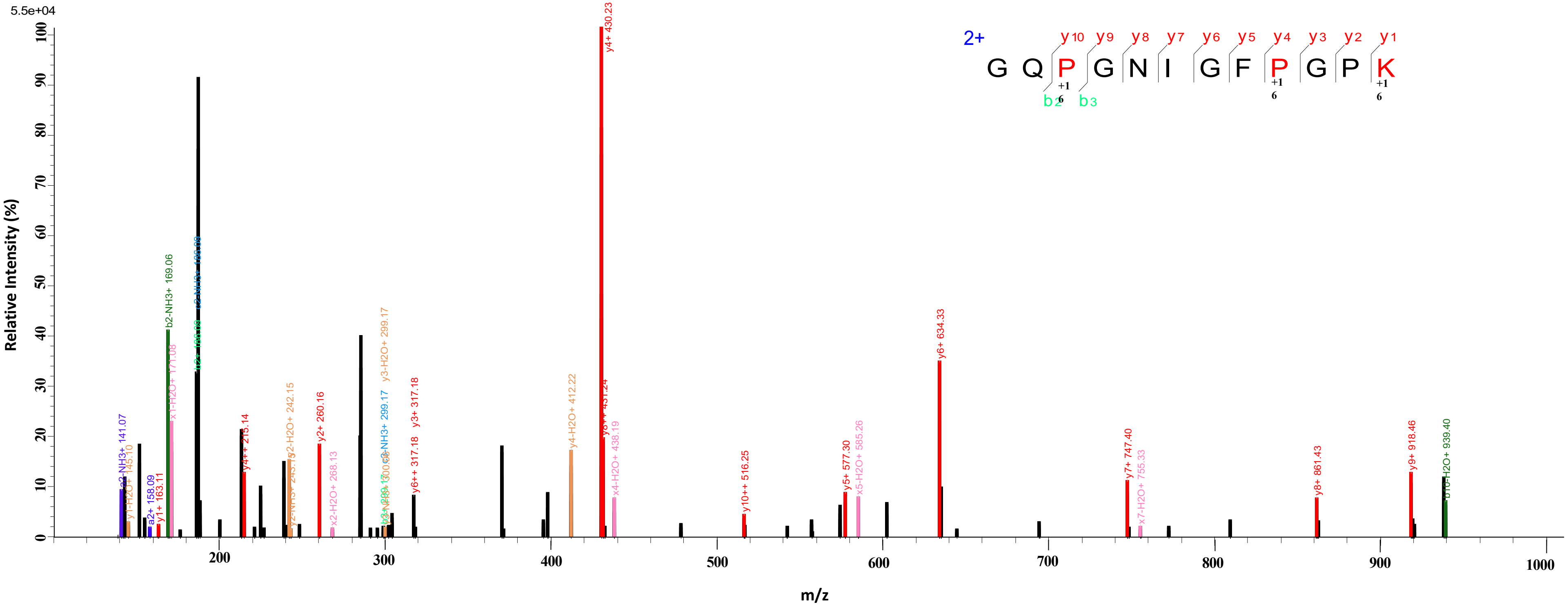

S2.73 m/z= 660.6644^{+3}

Zebrafish COL1A2 HyK^{500}

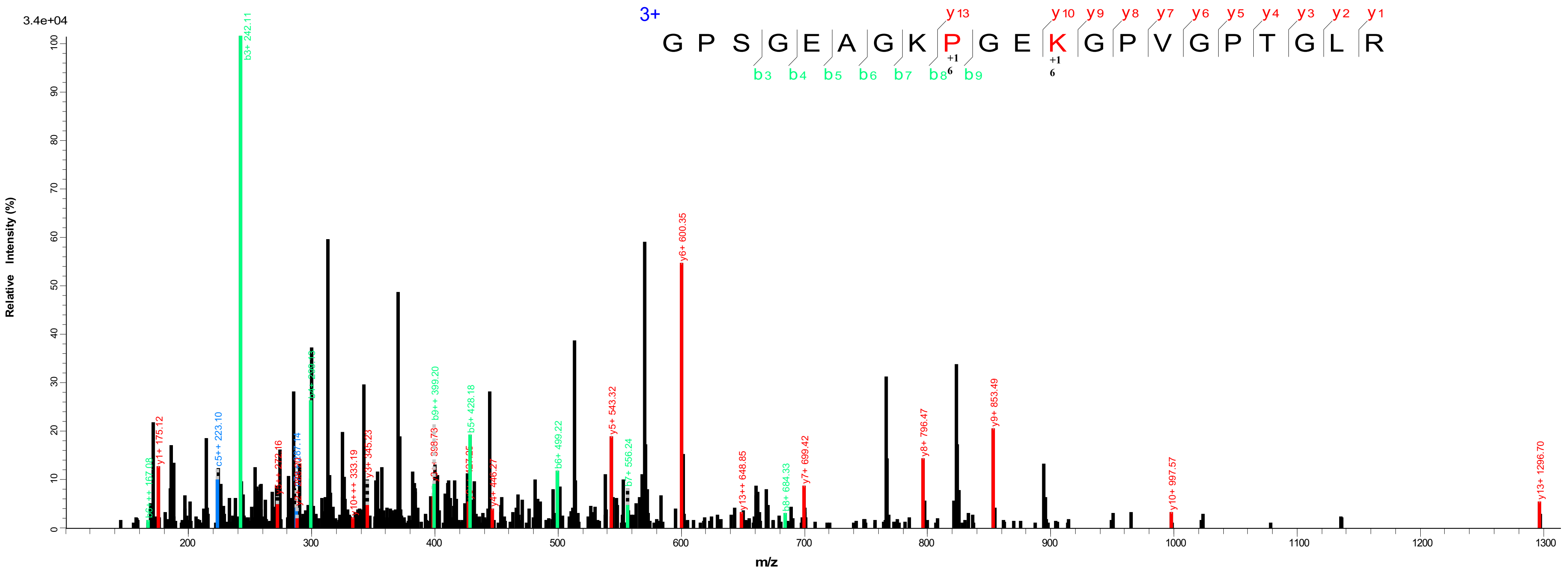

S2.74

m/z= 1060.9971^{+2}

Zebrafish COL1A2 HyK^{533} PSM

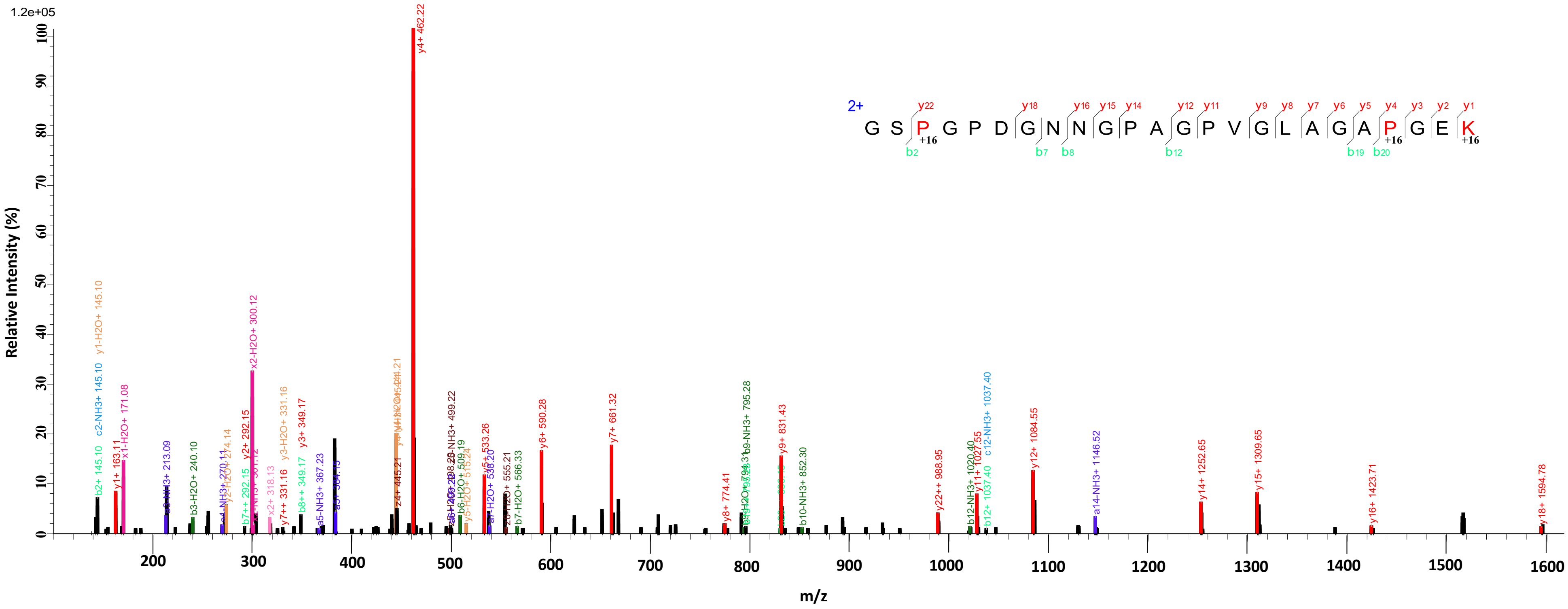

S2.75

m/z= 685.8494^{+2}

Zebrafish COL1A2 HyK^{578} PSM

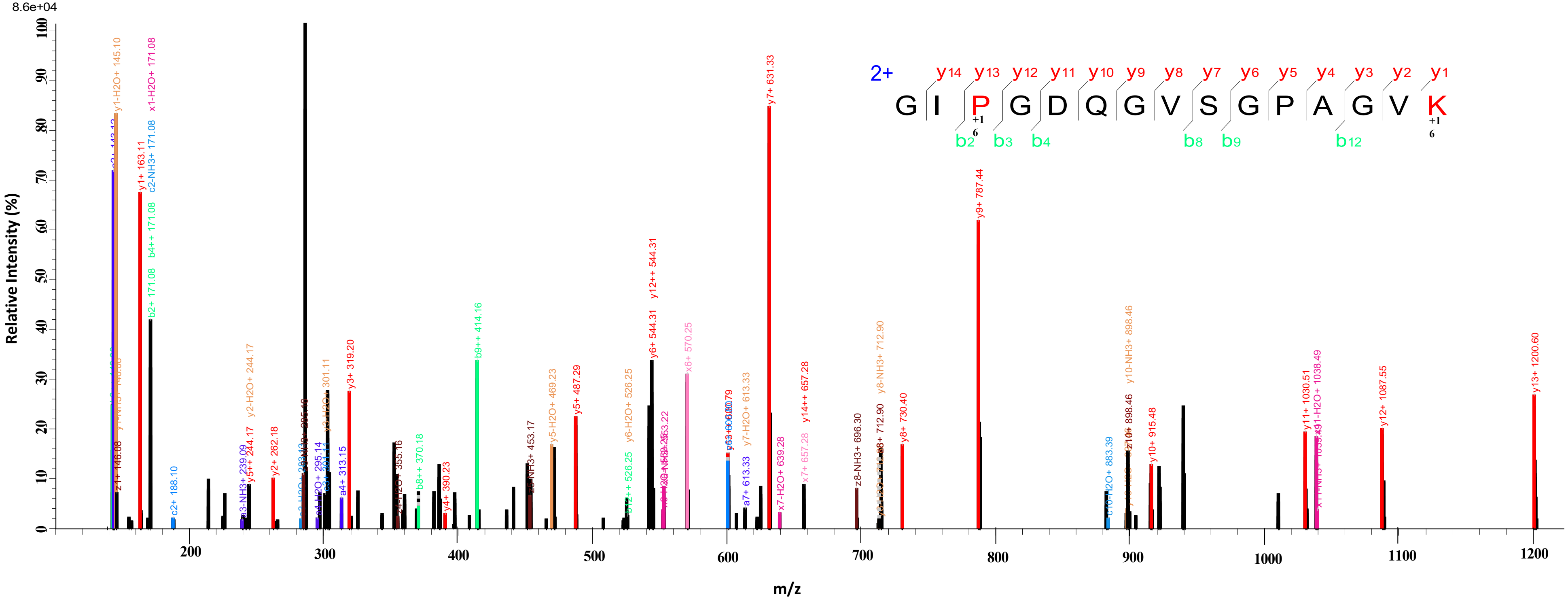

S2.76 m/z= 1018.9843 +2

Zebrafish COL1A2 GG-HyK[578]

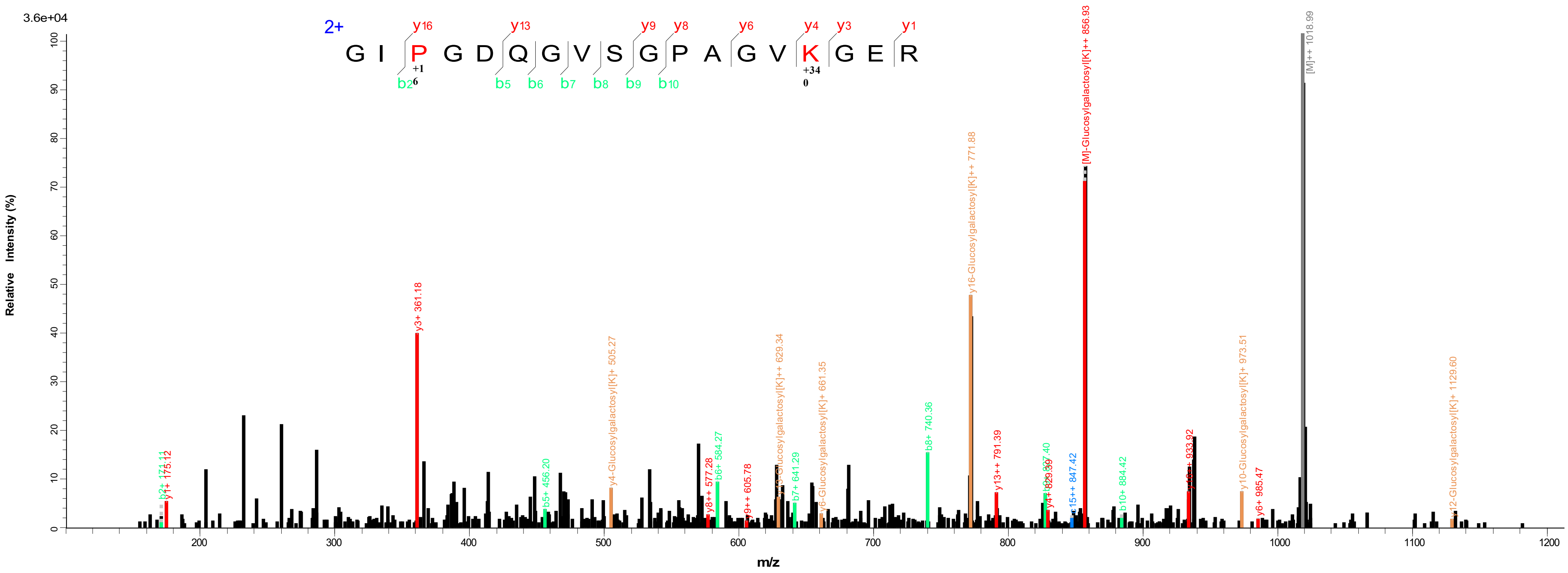

S2.77

m/z= 575.6131 $^{+3}$

Zebrafish COL1A2 HyK 644 & 647

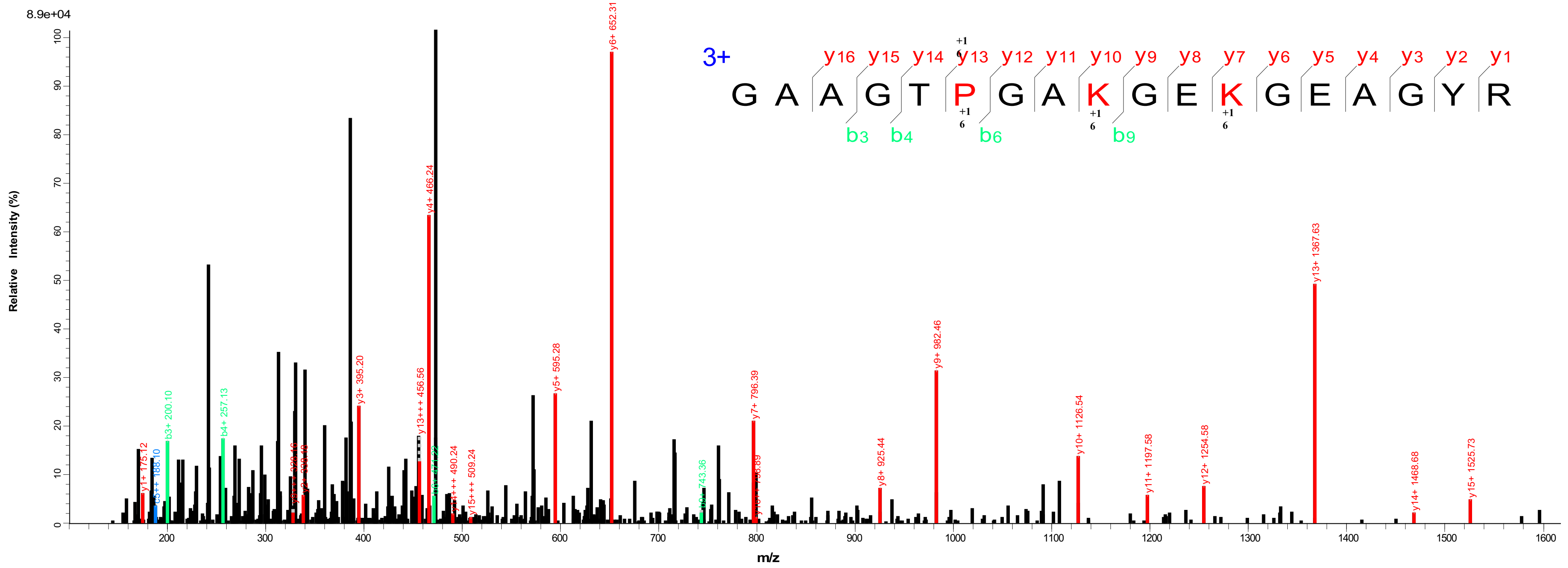

S2.78

m/z= 629.6290+3

Zebrafish COL1A2 G-HyK^{644}HyK^{647}

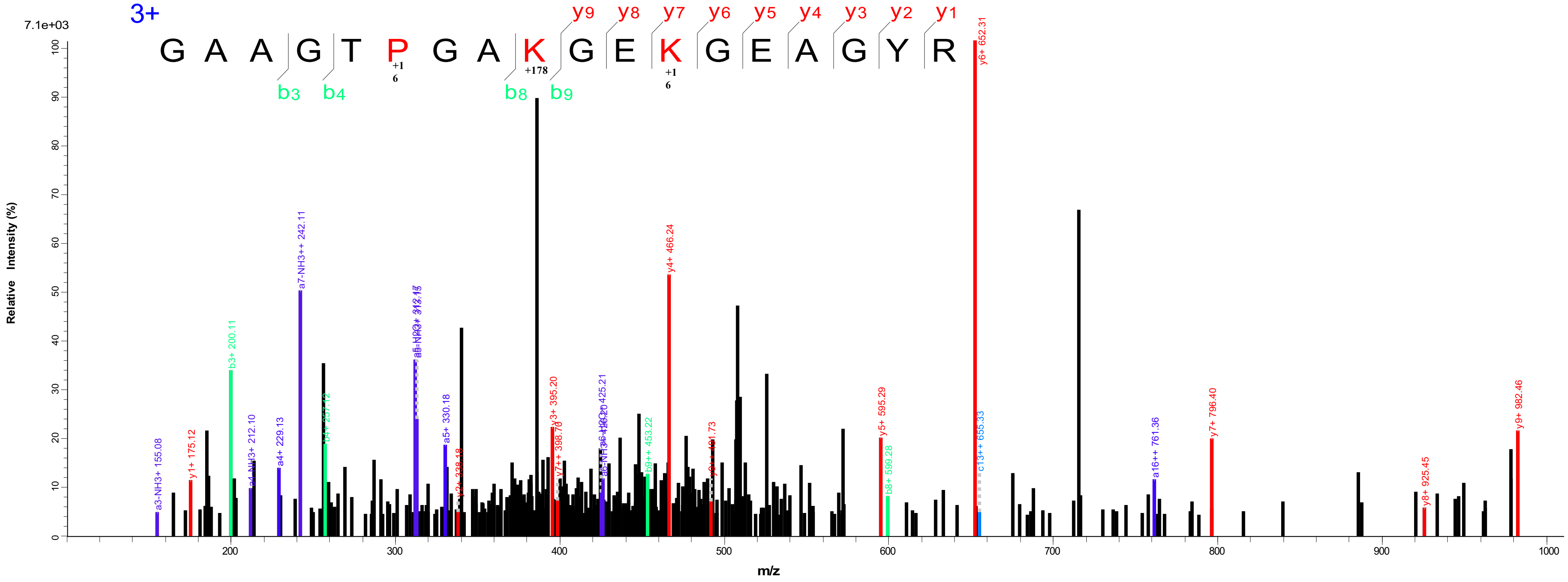

S2.79 m/z= 683.6472^{+3}

Zebrafish COL1A2 GG-HyK^{644}, HyK^{647}

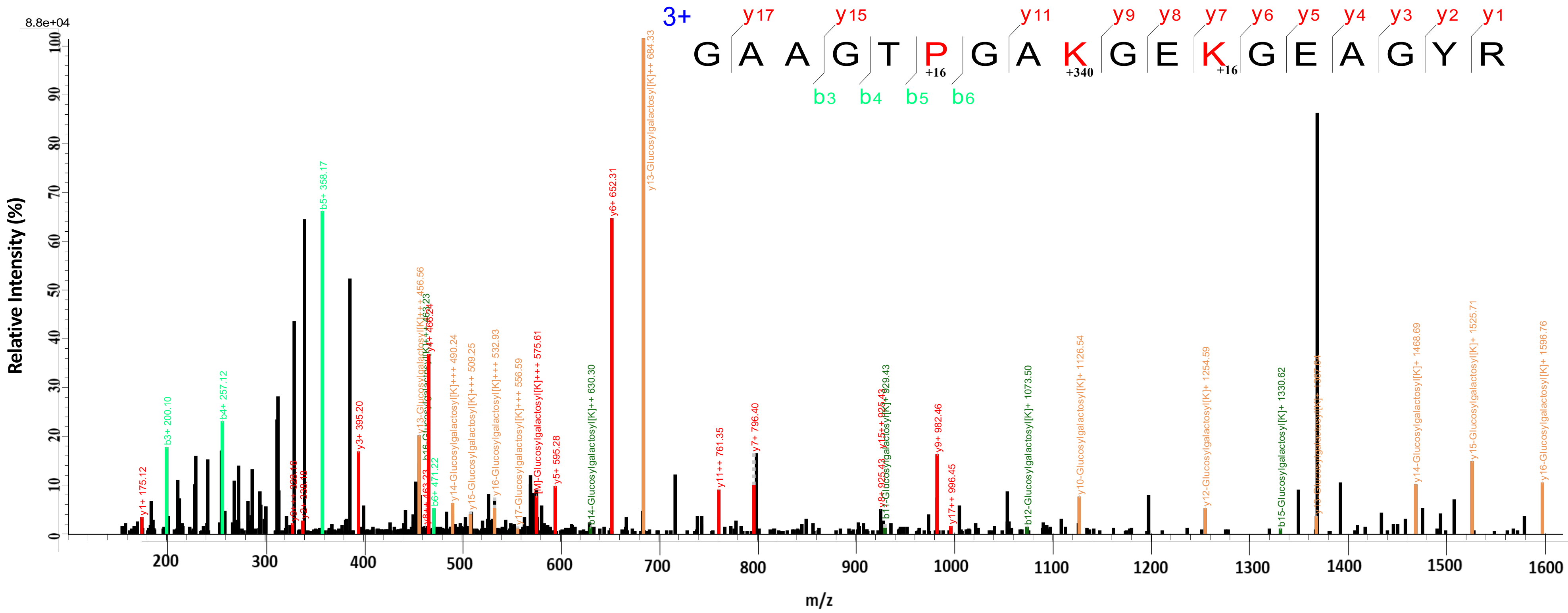

S2.80 m/z= 683.6477^{+3}

Zebrafish COL1A2 HyK^{644}GG-HyK^{647}

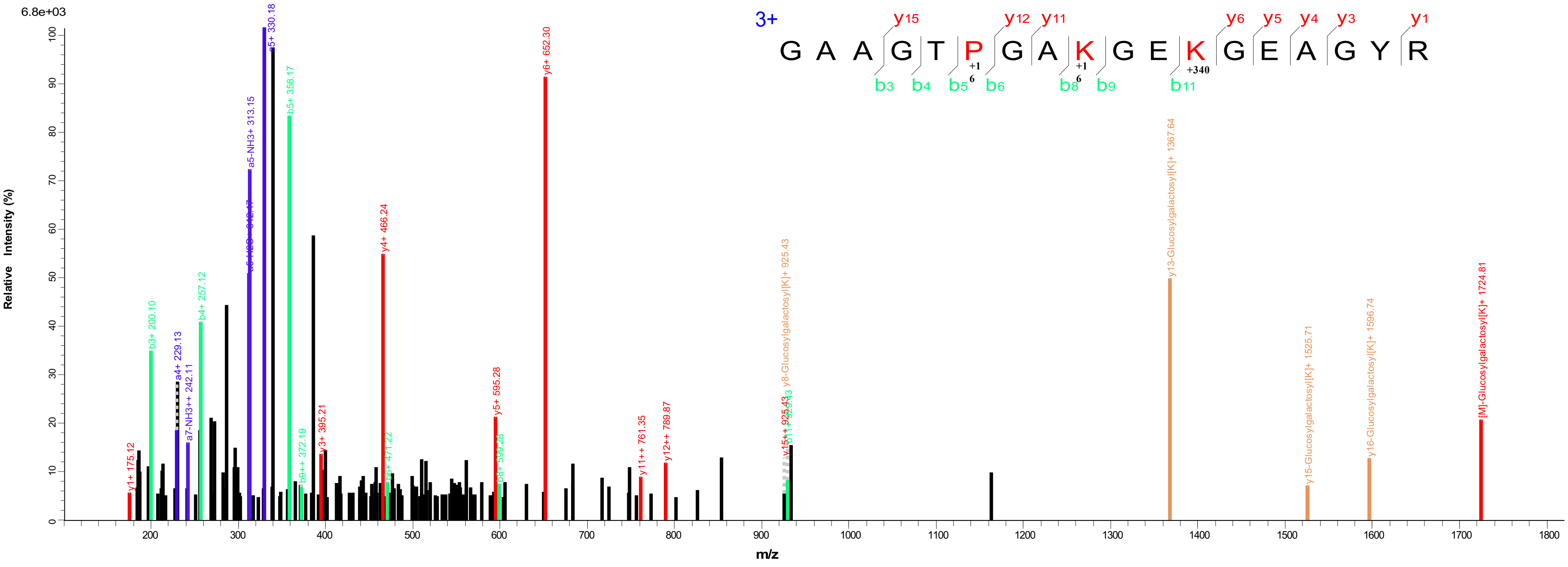

S2.81 m/z= 978.1477^{+3}

Zebrafish COL1A2 HyK^{683}

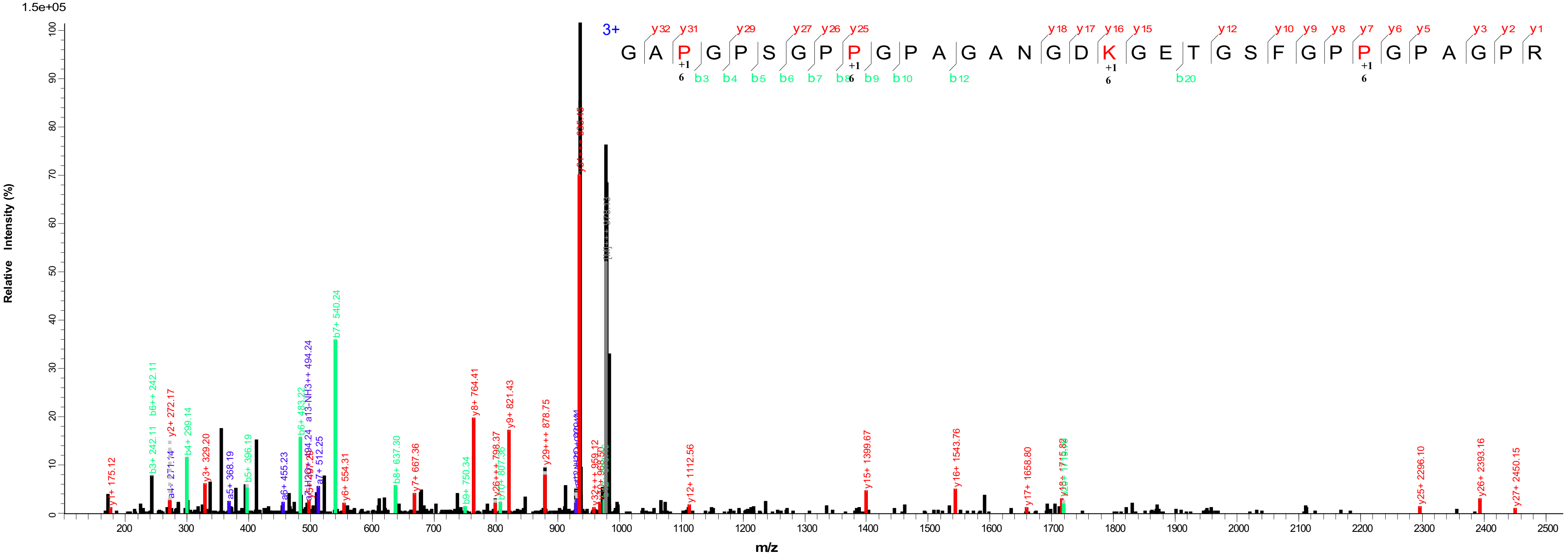

S2.82
m/z= 834.6666 $^{+4}$

Zebrafish COL1A2 HyK $^{731\ \&\ 737}$

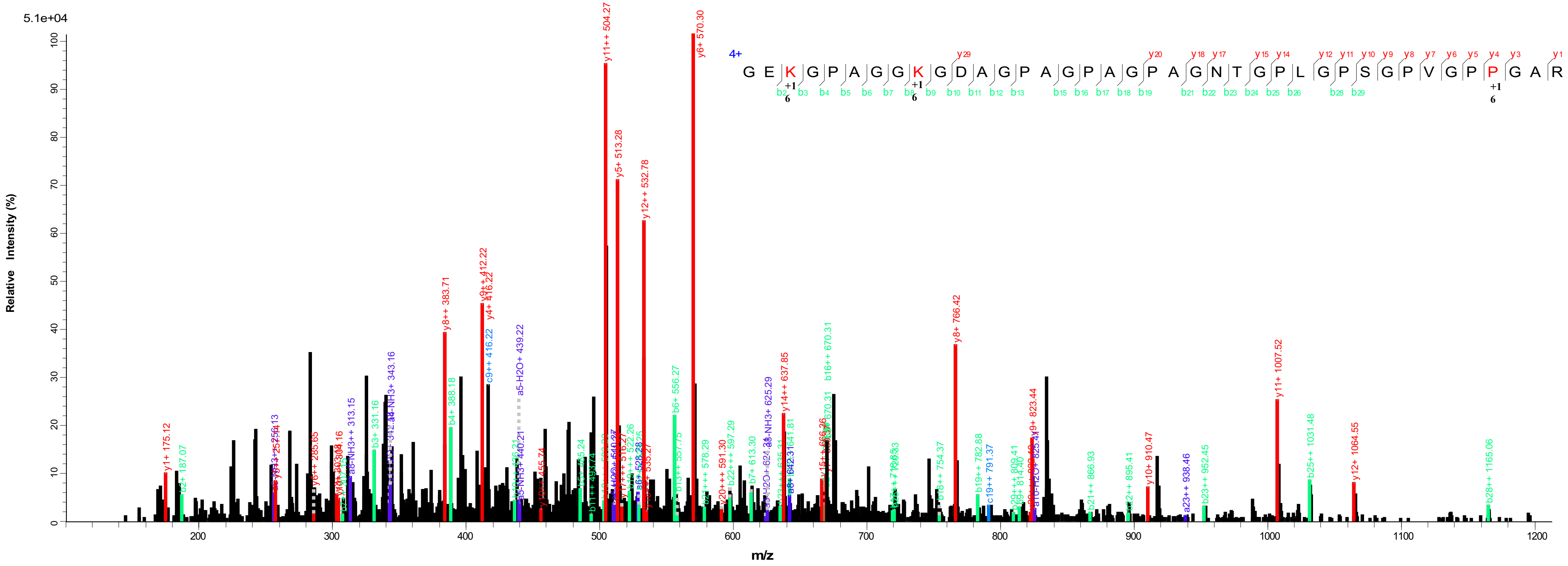

S2.83

m/z= 1111.5439 +2

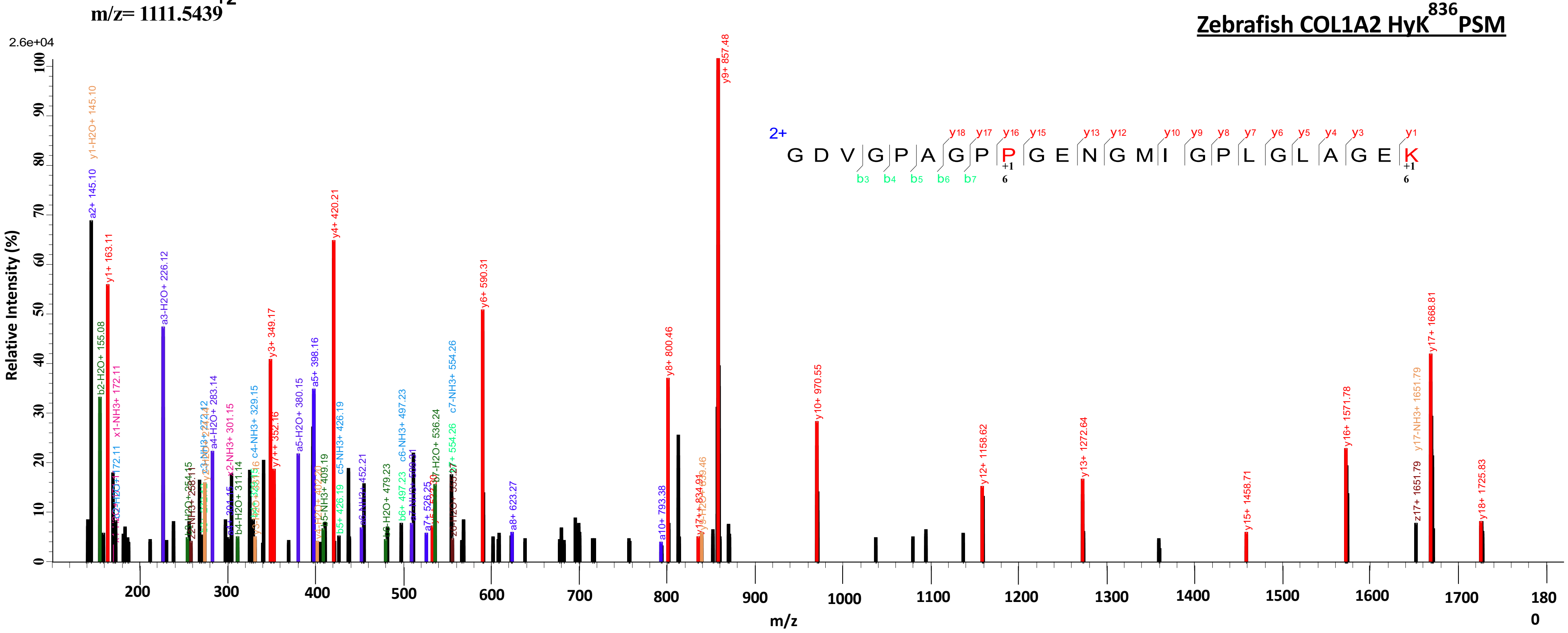

Supplementary Figure 3: Peptide spectral matches (PSMs) for 3-hydroprolines detected in collagen I alpha 1 chain present in the ECM of human and mice hearts (Data source- PXD0028908, PXD002488). For human data, PSMs with <20 ppm fragment mass tolerance were included in the analysis. For mice data, PSMs with <50 ppm fragment mass tolerance were included in the analysis. PSMs were identified by MyriMatch. The ’b’ and ‘y’ ions were annotated using pLABEL and IDPicker.

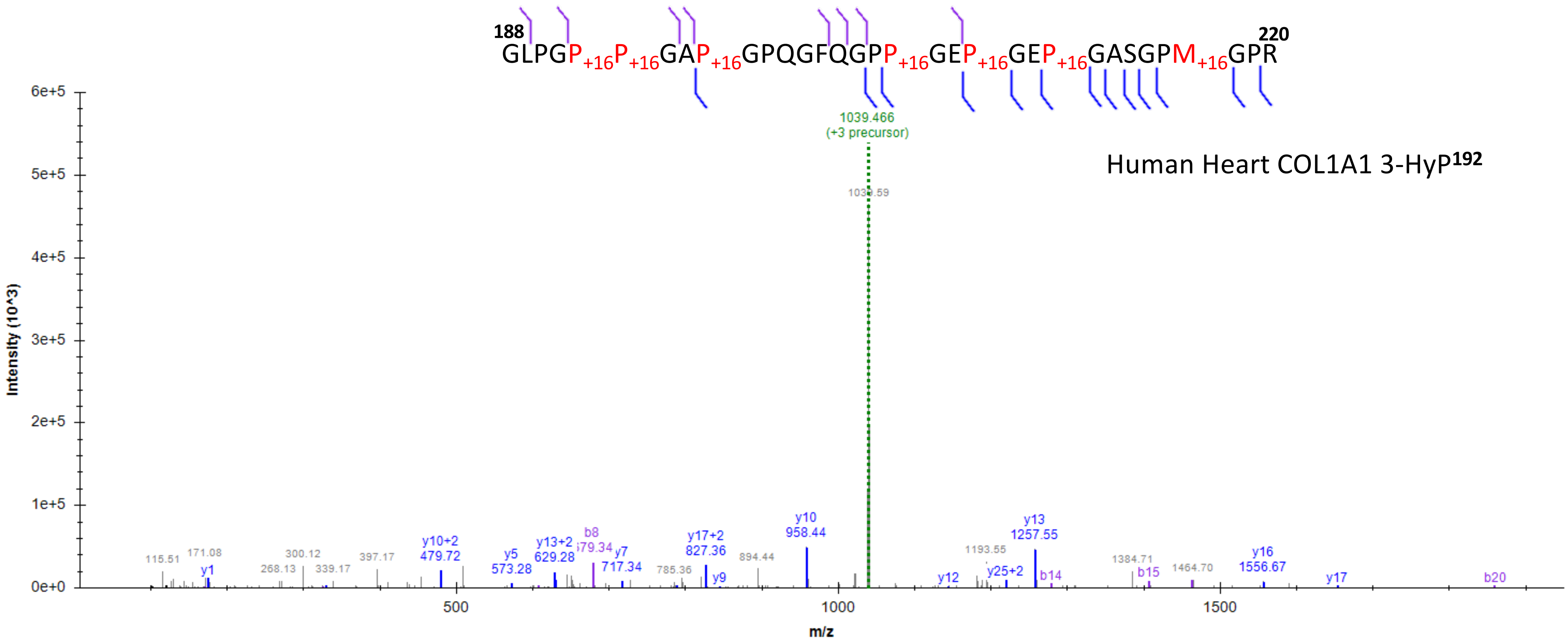
188
GLPGP+16P+16GAP+16GPQGFQGPP+16GEP+16GEP+16GASGPM+16GPR
220
Human Heart COL1A1 3-HyP192
1039.466
(+3 precursor)
Intensity (10^3)
m/z
6e+5
5e+5
4e+5
3e+5
2e+5
1e+5
0e+0
500
1000
1500
y1
y10+2 479.72
y5 573.28
y13+2 629.28
b8
y7 717.34
y17+2 827.36
y9
y10 958.44
y12
y25+2
y13 1257.55
b14
b15
y16 1556.67
y17
b20
115.51
171.08
268.13
300.12
339.17
397.17
785.36
894.44
1193.55
1384.71
1464.70

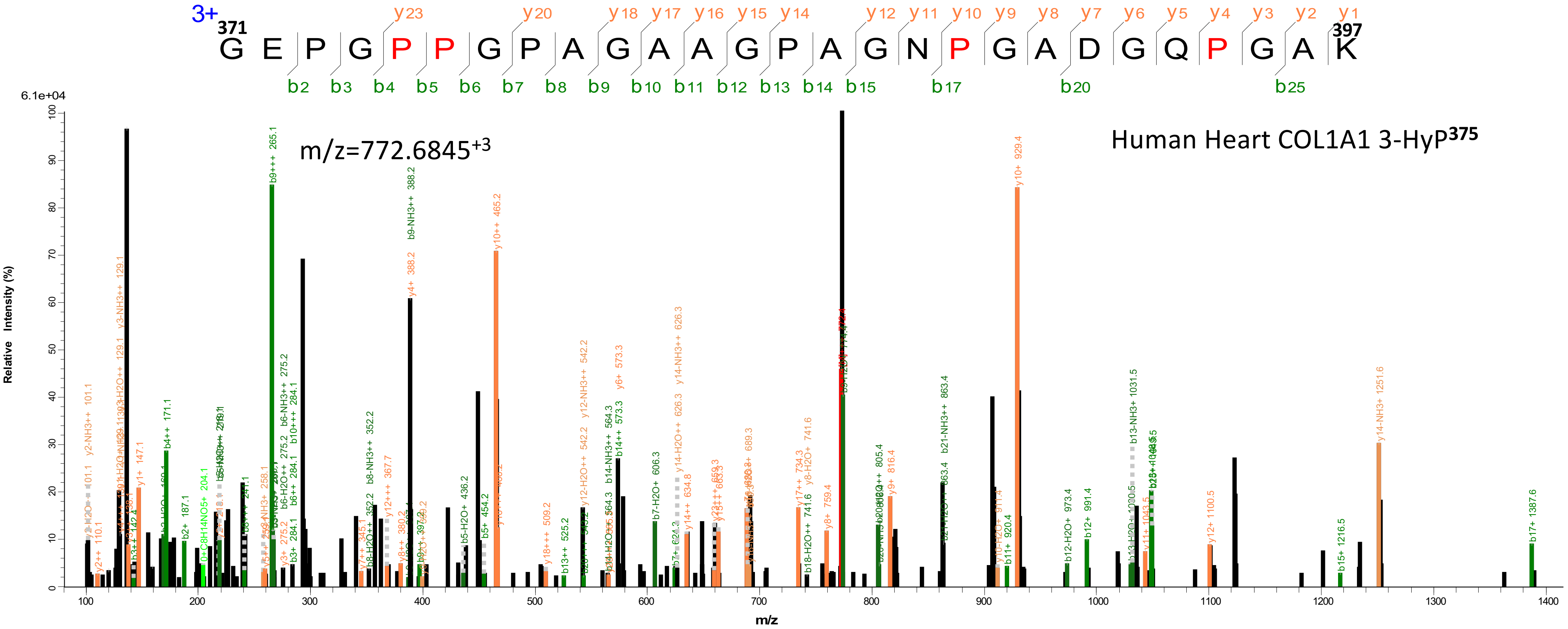

Human Heart COL1A1 3-HyP375
m/z=772.6845+3
3+
371
G E P G P P G P A G A A G P A G N P G A D G Q P G A K
397
y23 y20 y18 y17 y16 y15 y14 y12 y11 y10 y9 y8 y7 y6 y5 y4 y3 y2 y1
b2 b3 b4 b5 b6 b7 b8 b9 b10 b11 b12 b13 b14 b15 b17 b20 b25
6.1e+04
Relative Intensity (%)
m/z

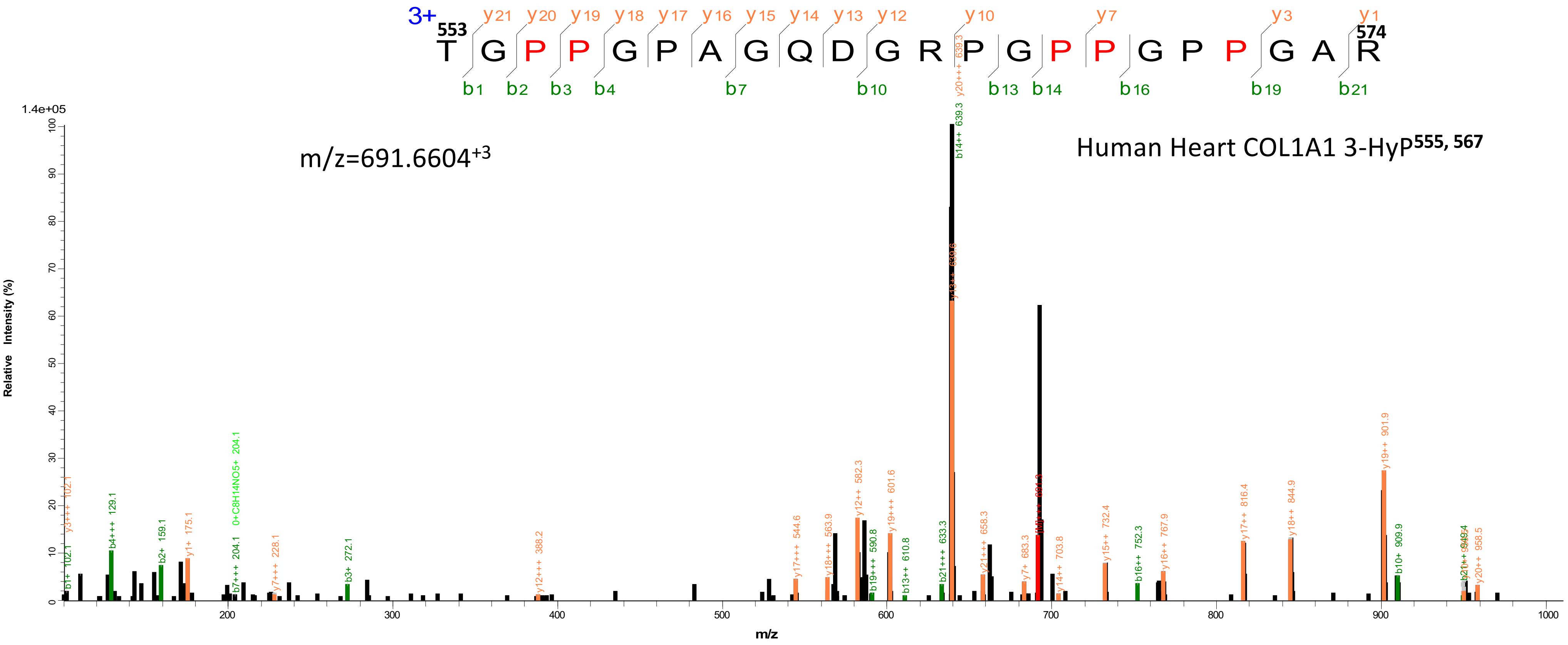

3+
553
T G P P G P A G Q D G R P G P P G P P G A R
574
y21 y20 y19 y18 y17 y16 y15 y14 y13 y12 y10 y7 y3 y1
b1 b2 b3 b4 b7 b10 b13 b14 b16 b19 b21
m/z=691.6604+3
Human Heart COL1A1 3-HyP555, 567
1.4e+05
Relative Intensity (%)
m/z

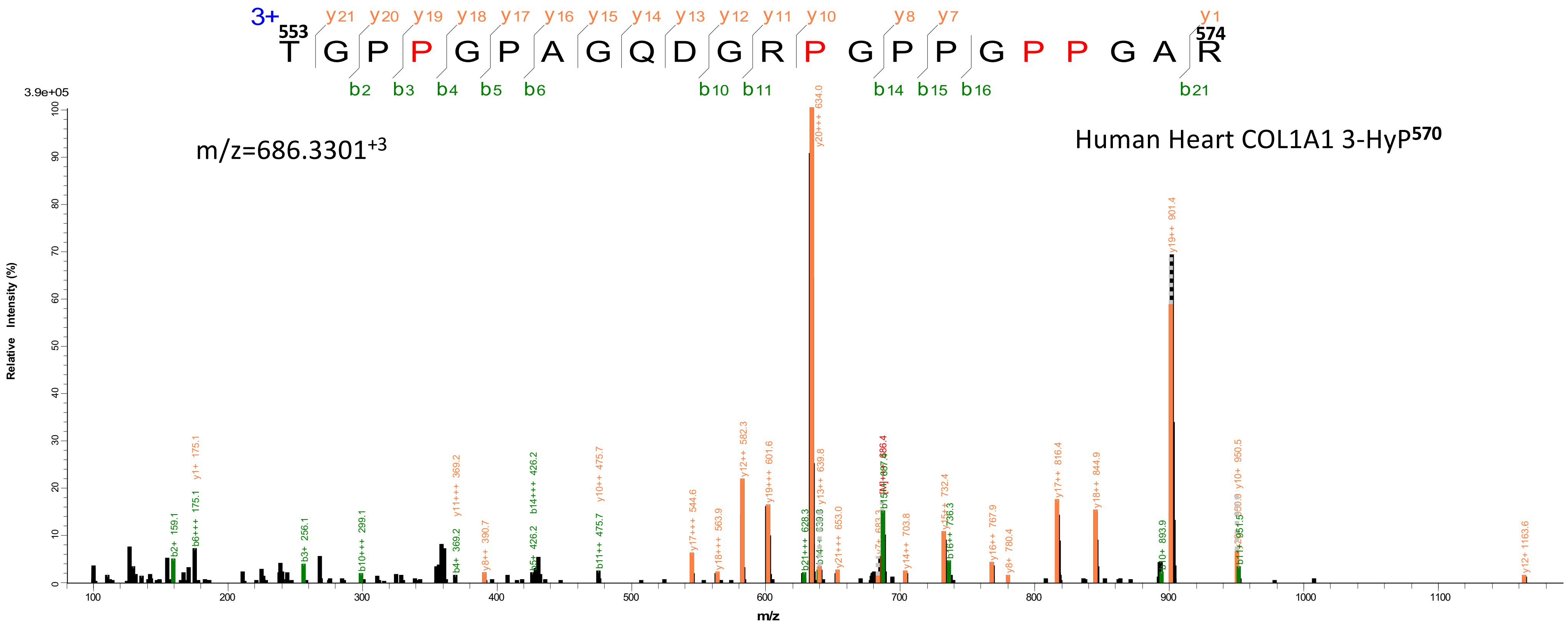
3+
553
574
T G P P G P A G Q D G R P G P P G P P G A R
y21 y20 y19 y18 y17 y16 y15 y14 y13 y12 y11 y10 y8 y7 y1
b2 b3 b4 b5 b6 b10 b11 b14 b15 b16 b21
3.9e+05
m/z=686.3301+3
Human Heart COL1A1 3-HyP570
Relative Intensity (%)
m/z
y20+++ 634.0
y19++ 901.4
y12++ 582.3
y19+++ 601.6
y17++ 816.4
y18++ 844.9
y10+ 950.5
y12+ 1163.6
y1+ 175.1
b6+++ 175.1
b2+ 159.1
b3+ 256.1
b10+++ 299.1
y11+++ 369.2
b4+ 369.2
y8++ 390.7
b14+++ 426.2
b5+ 426.2
y10++ 475.7
b11++ 475.7
y17+++ 544.6
y18+++ 563.9
b21+++ 628.3
y13++ 639.8
y21+++ 653.0
y14++ 703.8
y15++ 732.4
b16++ 736.3
y16++ 767.9
y8+ 780.4
b10+ 893.9
b11+ 951.5

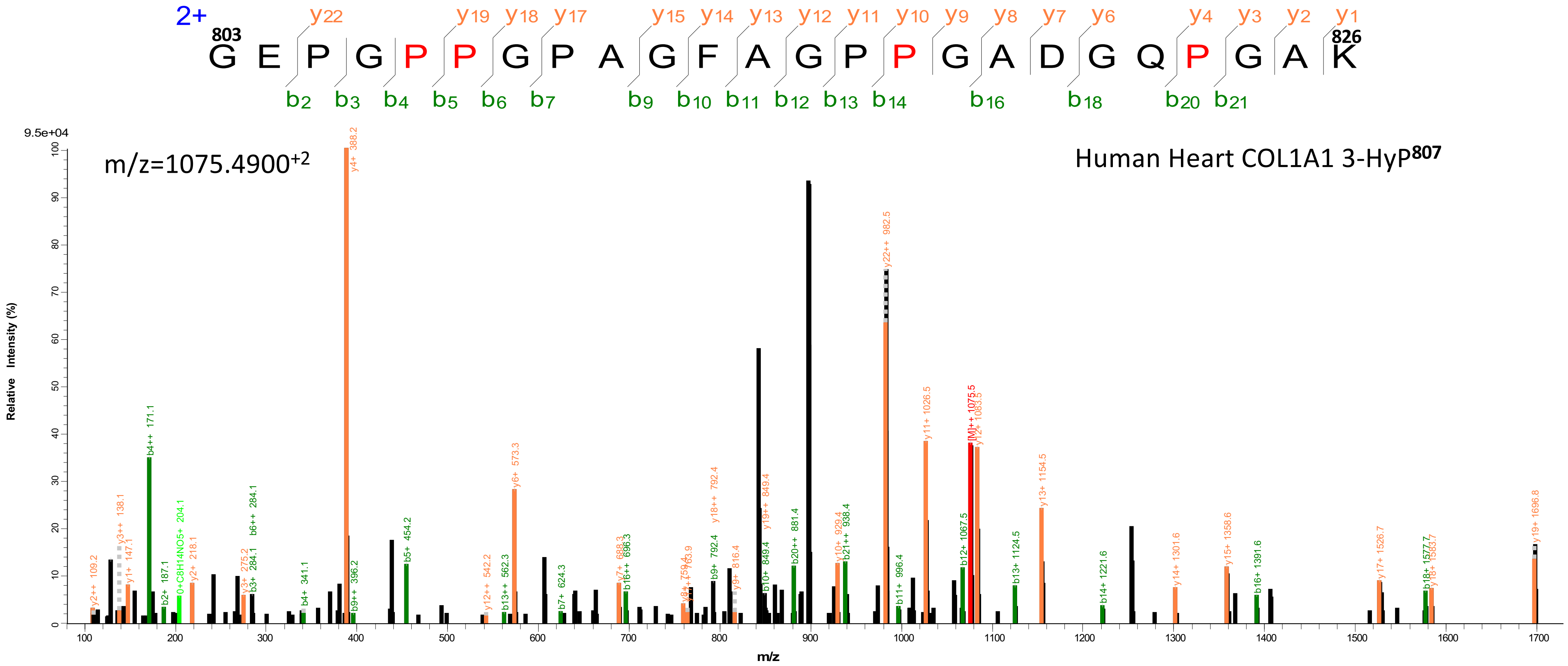
2+
803
G E P G P P G P A G F A G P P G A D G Q P G A K
826
y22 y19 y18 y17 y15 y14 y13 y12 y11 y10 y9 y8 y7 y6 y4 y3 y2 y1
b2 b3 b4 b5 b6 b7 b9 b10 b11 b12 b13 b14 b16 b18 b20 b21
m/z=1075.4900+2
Human Heart COL1A1 3-HyP807
9.5e+04
Relative Intensity (%)
m/z

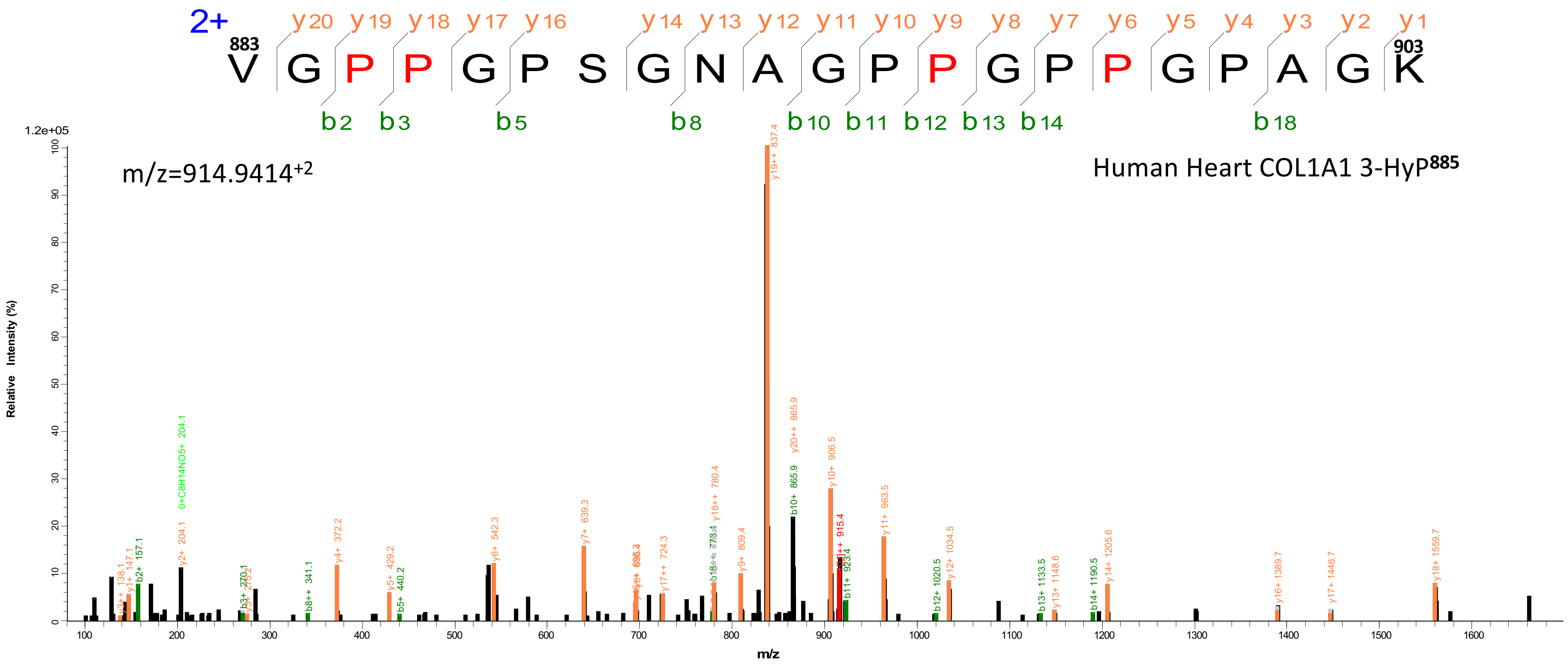
2+
883
V G P P G P S G N A G P P G P P G P A G K
903
y20 y19 y18 y17 y16 y14 y13 y12 y11 y10 y9 y8 y7 y6 y5 y4 y3 y2 y1
b2 b3 b5 b8 b10 b11 b12 b13 b14 b18
m/z=914.9414+2
Human Heart COL1A1 3-HyP885
1.2e+05
Relative Intensity (%)
m/z

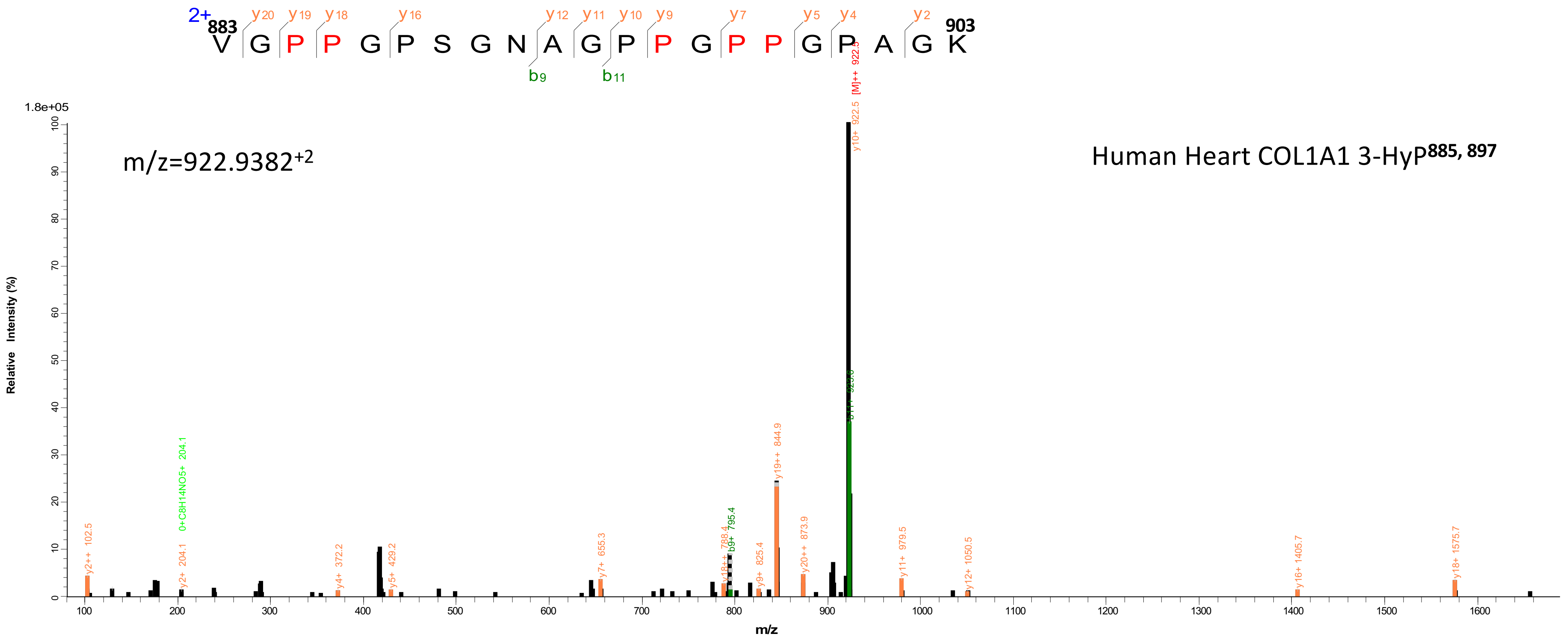
2+
883
V G P P G P S G N A G P P G P P G P A G K
903
y20 y19 y18 y16 y12 y11 y10 y9 y7 y5 y4 y2
b9 b11
1.8e+05
m/z=922.9382+2
Human Heart COL1A1 3-HyP885, 897
Relative Intensity (%)
m/z
y2++ 102.5
0+C8H14NO5+ 204.1
y2+ 204.1
y4+ 372.2
y5+ 429.2
y7+ 655.3
b9+ 795.4
y9+ 825.4
y19++ 844.9
y20++ 873.9
[M]++ 922.5
y10+ 922.5
y11+ 979.5
y12+ 1050.5
y16+ 1405.7
y18+ 1575.7

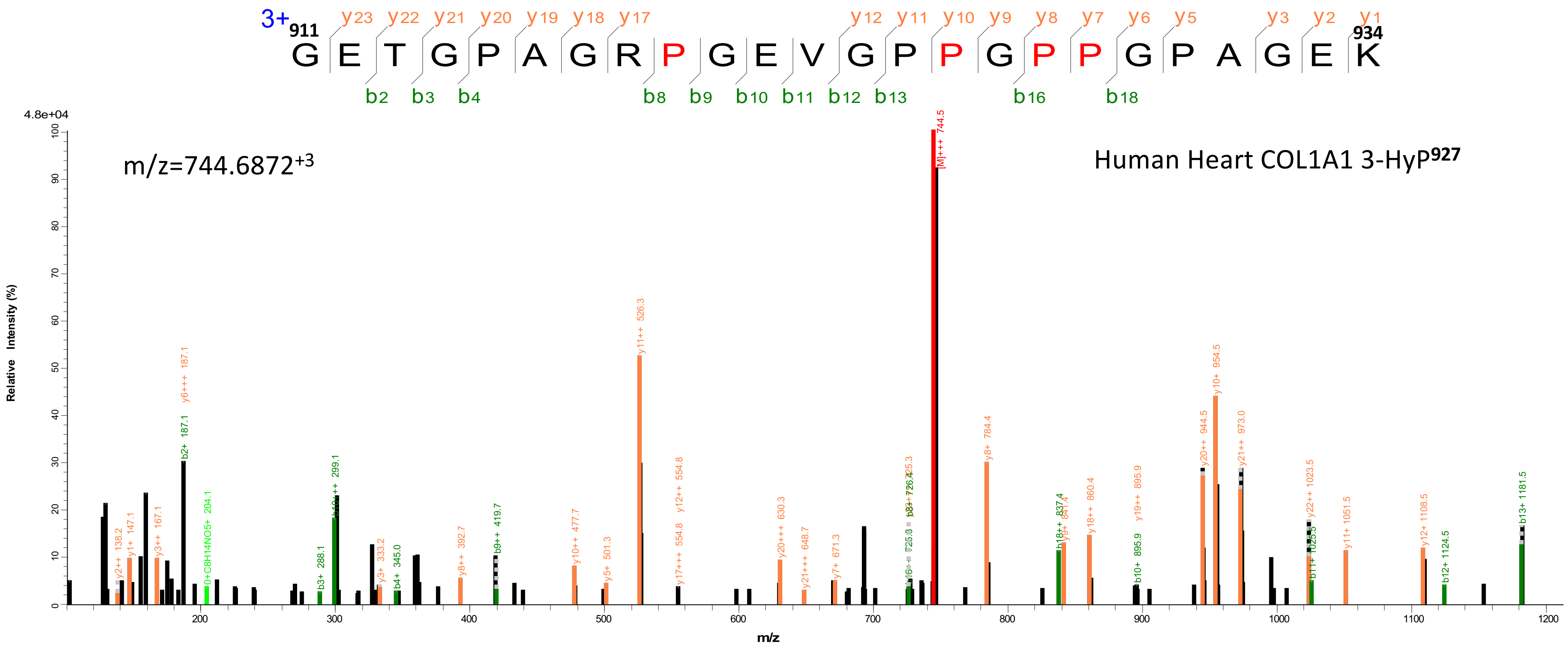
3+ 911 G E T G P A G R P G E V G P P G P P G P A G E K 934
y23 y22 y21 y20 y19 y18 y17 y12 y11 y10 y9 y8 y7 y6 y5 y3 y2 y1
b2 b3 b4 b8 b9 b10 b11 b12 b13 b16 b18
m/z=744.6872+3
Human Heart COL1A1 3-HyP927
4.8e+04
Relative Intensity (%)
m/z

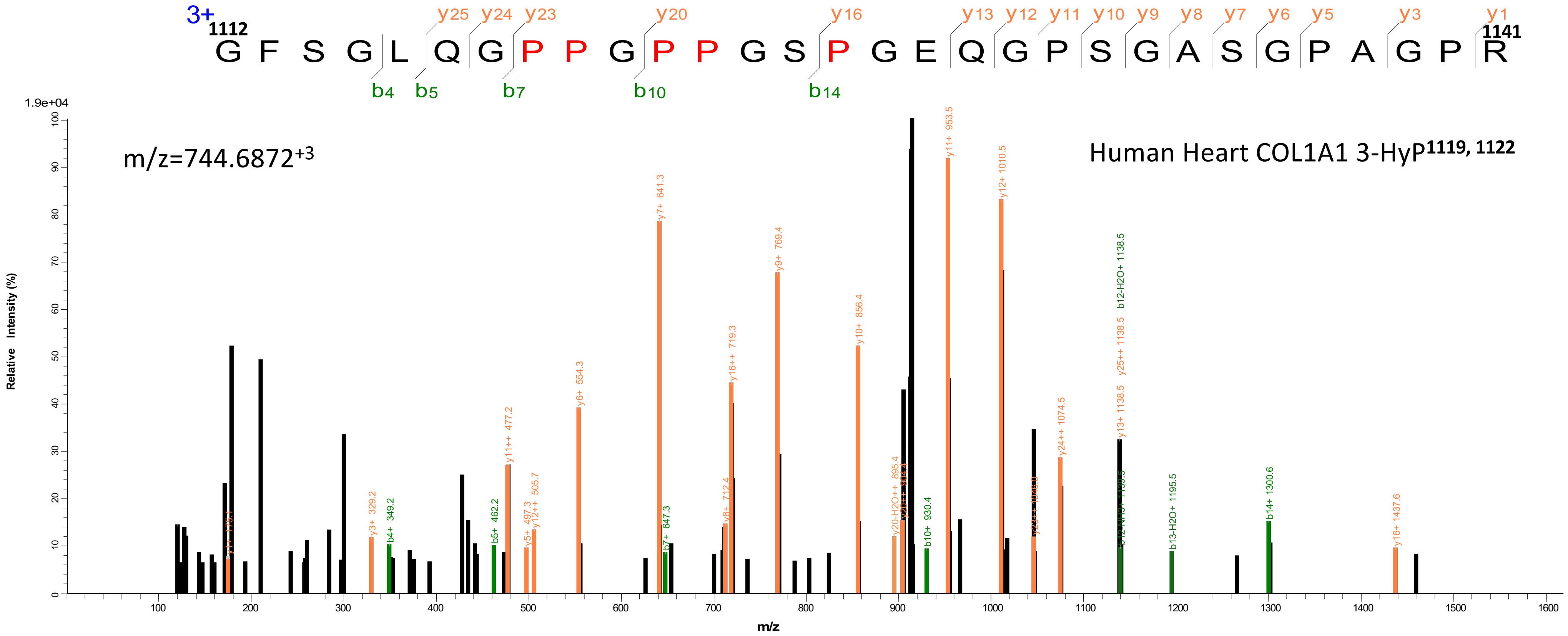

3+
1112
G F S G L Q G P P G P P G S P G E Q G P S G A S G P A G P R
1141
y25 y24 y23 y20 y16 y13 y12 y11 y10 y9 y8 y7 y6 y5 y3 y1
b4 b5 b7 b10 b14
m/z=744.6872+3
Human Heart COL1A1 3-HyP1119, 1122
1.9e+04
Relative Intensity (%)
m/z

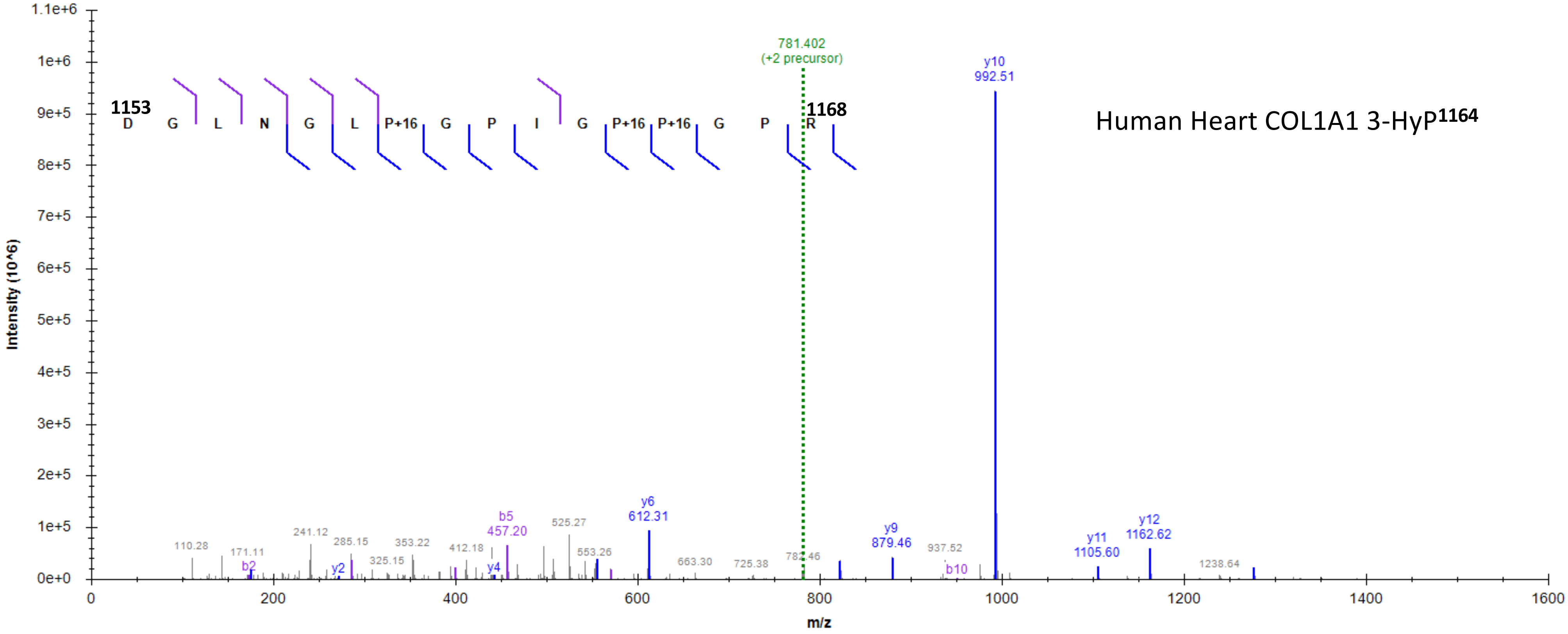

Human Heart COL1A1 3-HyP1164
1153
D G L N G L P+16 G P I G P+16 P+16 G P R
1168
781.402
(+2 precursor)
y10
992.51
y6
612.31
b5
457.20
y9
879.46
y11
1105.60
y12
1162.62
b2
y2
y4
b10
110.28
171.11
241.12
285.15
325.15
353.22
412.18
525.27
553.26
663.30
725.38
937.52
1238.64
Intensity (10^6)
m/z

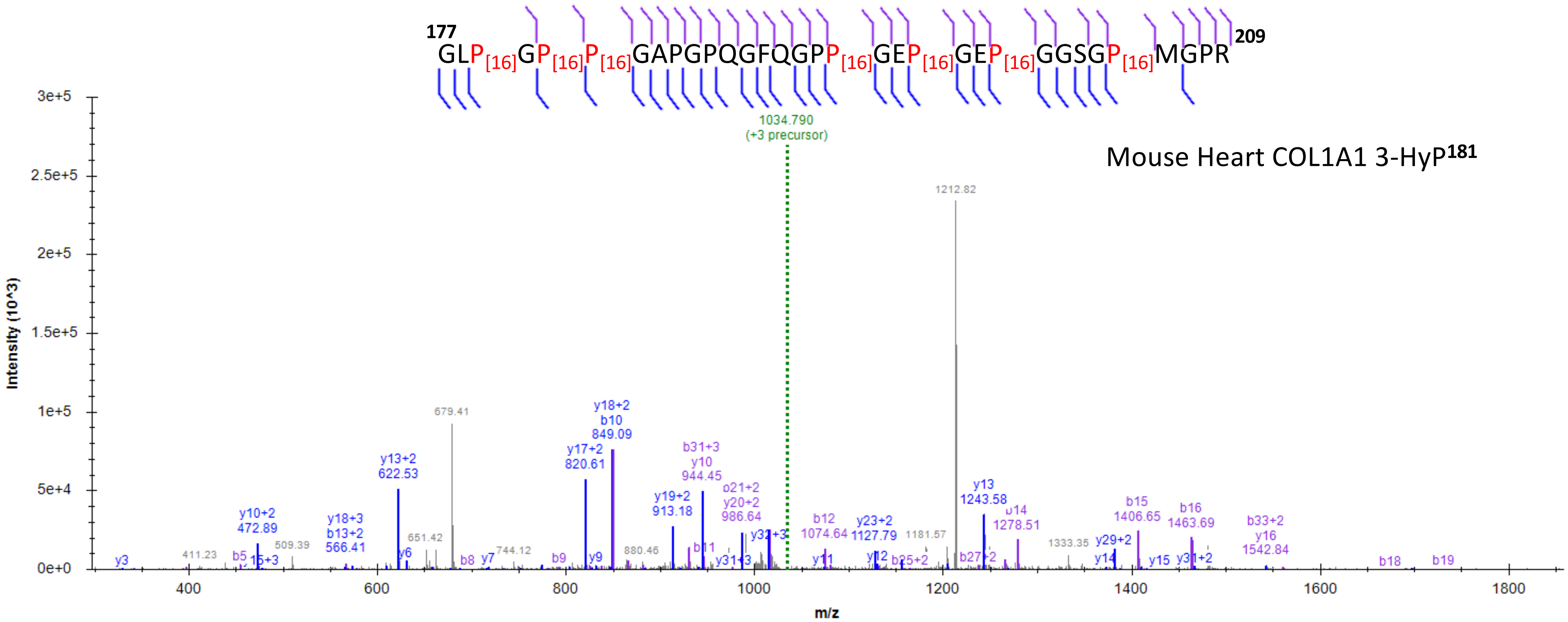

177
GLP[16]GP[16]P[16]GAPGPQGFQGPP[16]GEP[16]GEP[16]GGSGP[16]MGPR
209
Mouse Heart COL1A1 3-HyP181
1034.790
(+3 precursor)
1212.82
679.41
y18+2
b10
849.09
y17+2
820.61
b31+3
y10
944.45
y13+2
622.53
y19+2
913.18
b21+2
y20+2
986.64
y13
1243.58
b14
1278.51
b15
1406.65
b16
1463.69
b33+2
y16
1542.84
y10+2
472.89
y18+3
b13+2
566.41
b12
1074.64
y23+2
1127.79
1181.57
1333.35
y29+2
509.39
651.42
744.12
880.46
411.23
Intensity (10^3)
m/z
3e+5
2.5e+5
2e+5
1.5e+5
1e+5
5e+4
0e+0
400
600
800
1000
1200
1400
1600
1800

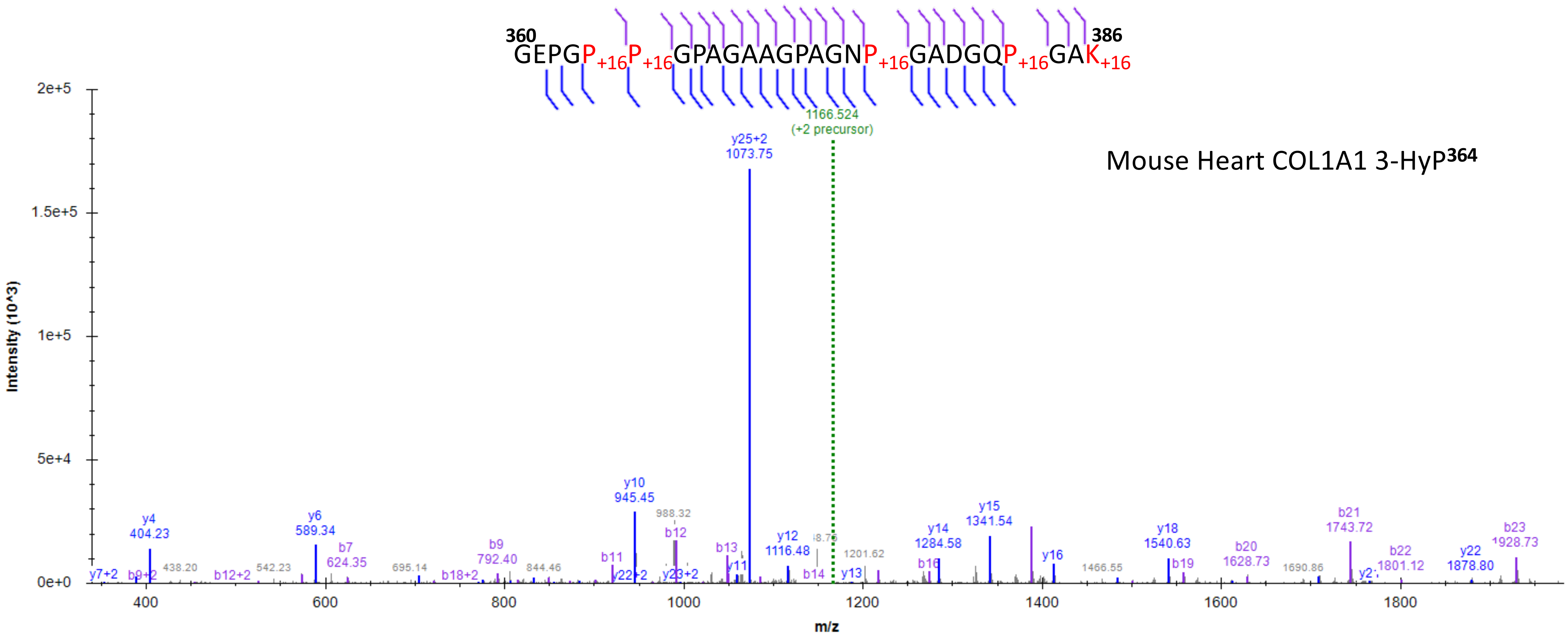

360
GEPGP+16P+16GPAGAAGPAGNP+16GADGQP+16GAK+16
386
Mouse Heart COL1A1 3-HyP364
1166.524
(+2 precursor)
y25+2
1073.75
y10
945.45
y4
404.23
y6
589.34
b7
624.35
b9
792.40
b11
b12
b13
y12
1116.48
y14
1284.58
y15
1341.54
y16
y18
1540.63
b19
b20
1628.73
b21
1743.72
b22
1801.12
y22
1878.80
b23
1928.73
Intensity (10^3)
m/z

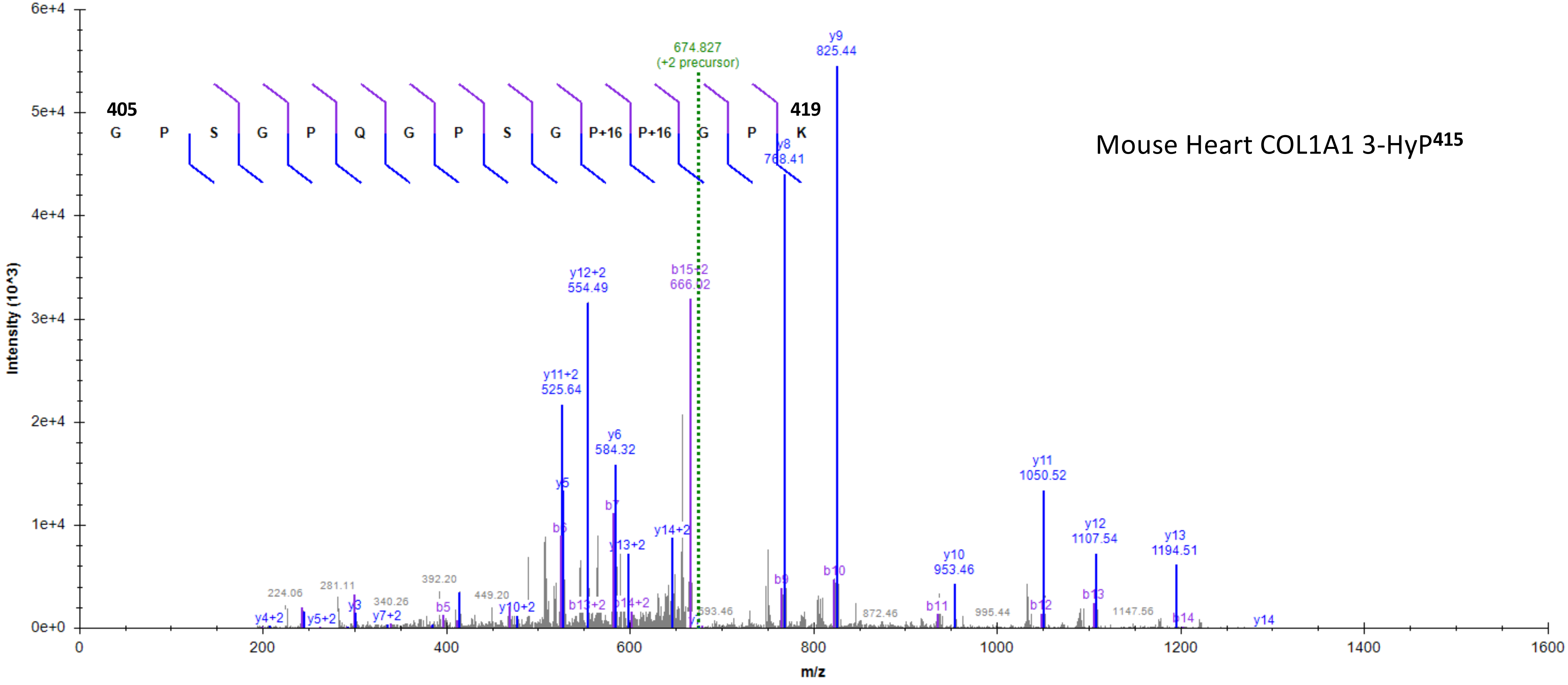
Mouse Heart COL1A1 3-HyP415
405
G P S G P Q G P S G P+16 P+16 G P K
419
674.827
(+2 precursor)
y9
825.44
y8
768.41
y12+2
554.49
b15+2
666.02
y11+2
525.64
y6
584.32
y5
b7
b6
y14+2
y13+2
y11
1050.52
y12
1107.54
y13
1194.51
y10
953.46
b10
b9
b11
b12
b13
b14
y14
224.06
281.11
340.26
392.20
449.20
593.46
872.46
995.44
1147.56
y4+2
y5+2
y3
y7+2
b5
y10+2
b13+2
b14+2
Intensity (10^3)
m/z
0e+0
1e+4
2e+4
3e+4
4e+4
5e+4
6e+4
0
200
400
600
800
1000
1200
1400
1600

Mouse Heart COL1A1 3-HyP472

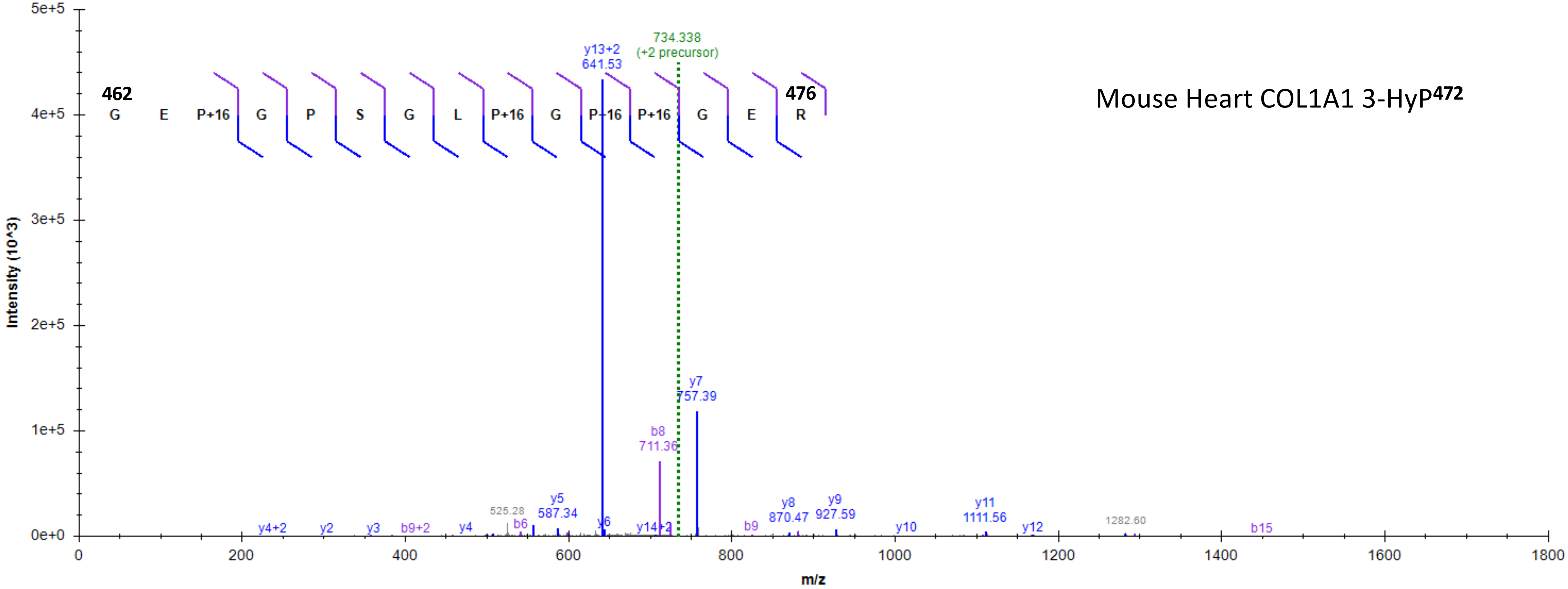

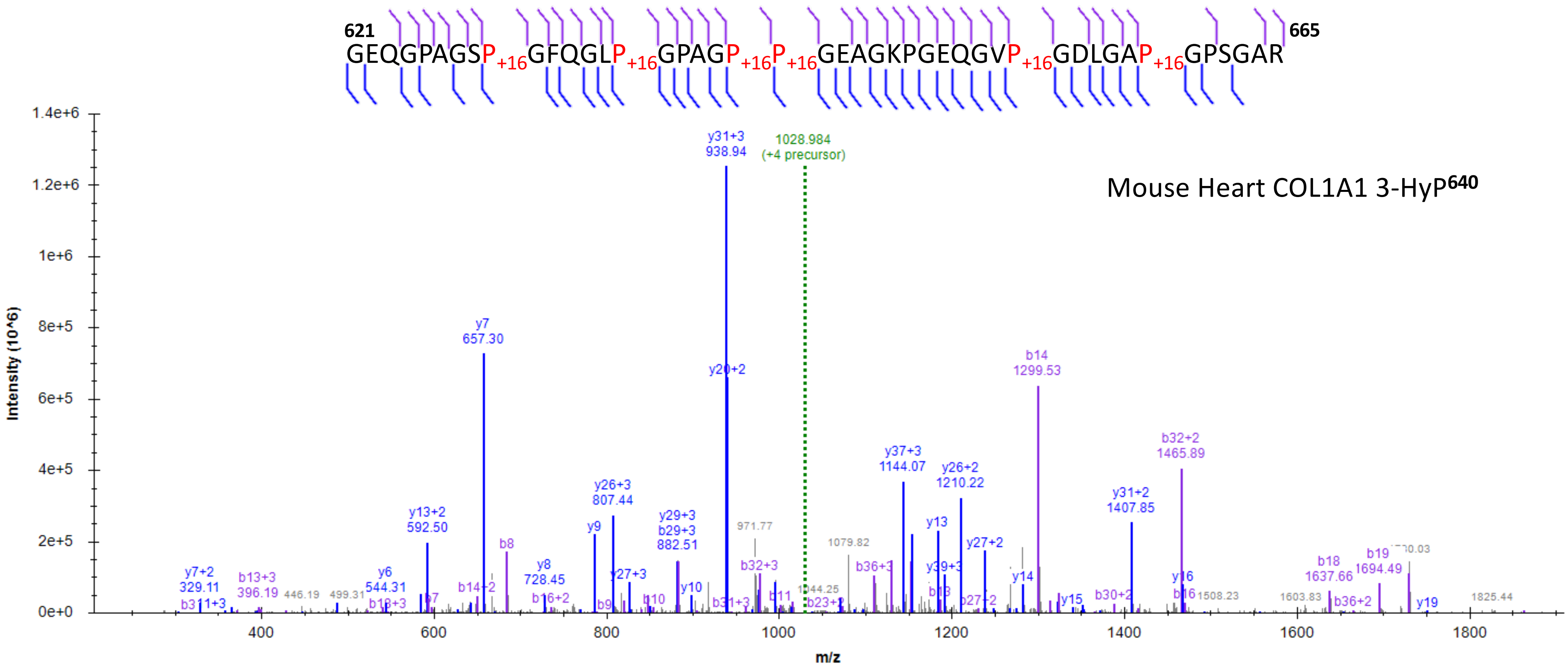
621
GEQGPAGSP+16GFQGLP+16GPAGP+16P+16GEAGKPGEQGVP+16GDLGAP+16GPSGAR
665
Mouse Heart COL1A1 3-HyP640
1.4e+6
1.2e+6
1e+6
8e+5
6e+5
4e+5
2e+5
0e+0
Intensity (10^6)
400
600
800
1000
1200
1400
1600
1800
m/z
y31+3 938.94
1028.984 (+4 precursor)
y7 657.30
y20+2
b14 1299.53
b32+2 1465.89
y37+3 1144.07
y26+2 1210.22
y26+3 807.44
y31+2 1407.85
y13+2 592.50
y29+3 b29+3 882.51
y9
y13
y27+2
b8
y8 728.45
971.77
1079.82
b32+3
b36+3
y39+3
y14
b18 1637.66
b19 1694.49
y16
y7+2 329.11
b13+3 396.19
y6 544.31
446.19
499.31
b14+2
y27+3
y10
1044.25
b23+2
b13
b27+2
y15
b30+2
b16
1508.23
1603.83
b36+2
y19
1825.44
b31+3
b18+3
b7
b16+2
b9
b10
b11

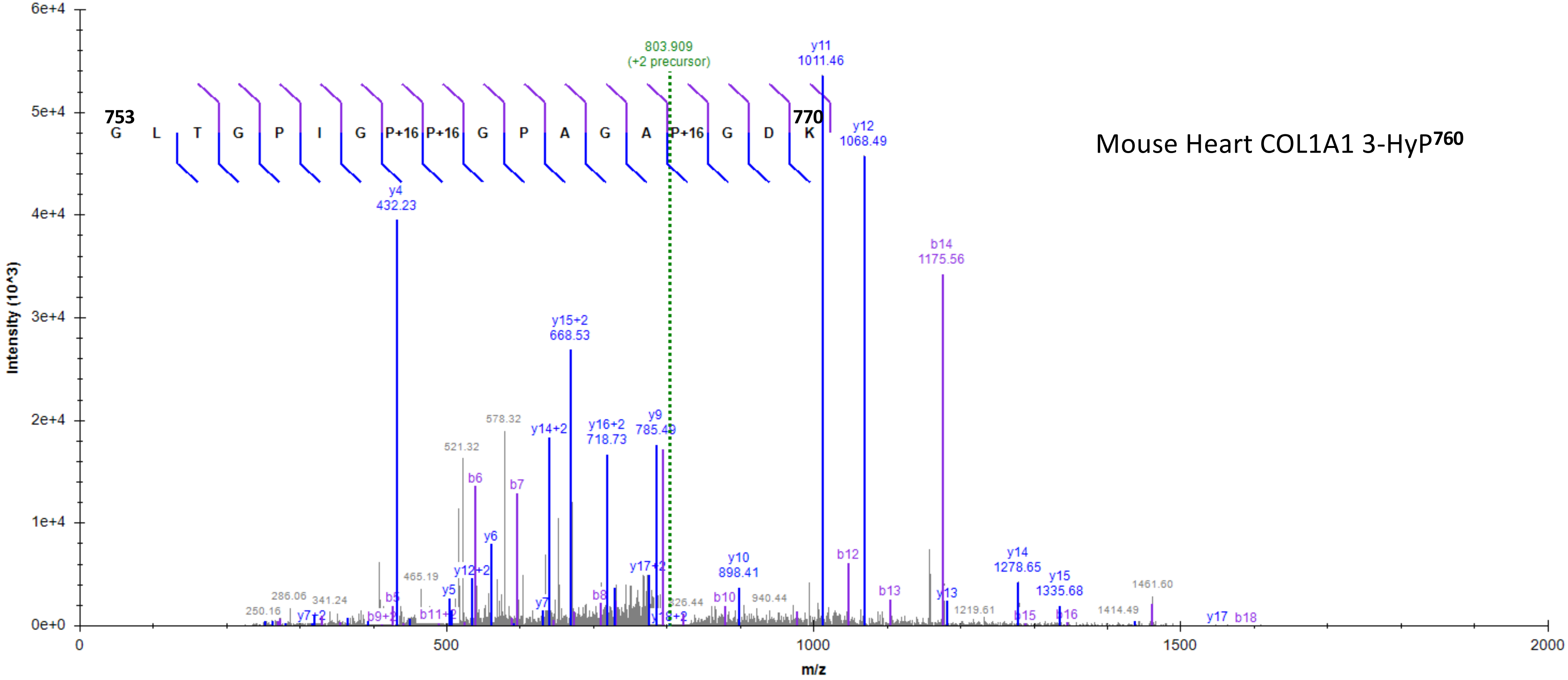
Mouse Heart COL1A1 3-HyP760
753
770
G L T G P I G P+16 P+16 G P A G A P+16 G D K
803.909
(+2 precursor)
y4
432.23
y11
1011.46
y12
1068.49
b14
1175.56
y15+2
668.53
y14+2
y16+2
718.73
y9
785.49
578.32
521.32
b6
b7
y6
y10
898.41
b12
y14
1278.65
y15
1335.68
1461.60
Intensity (10^3)
m/z

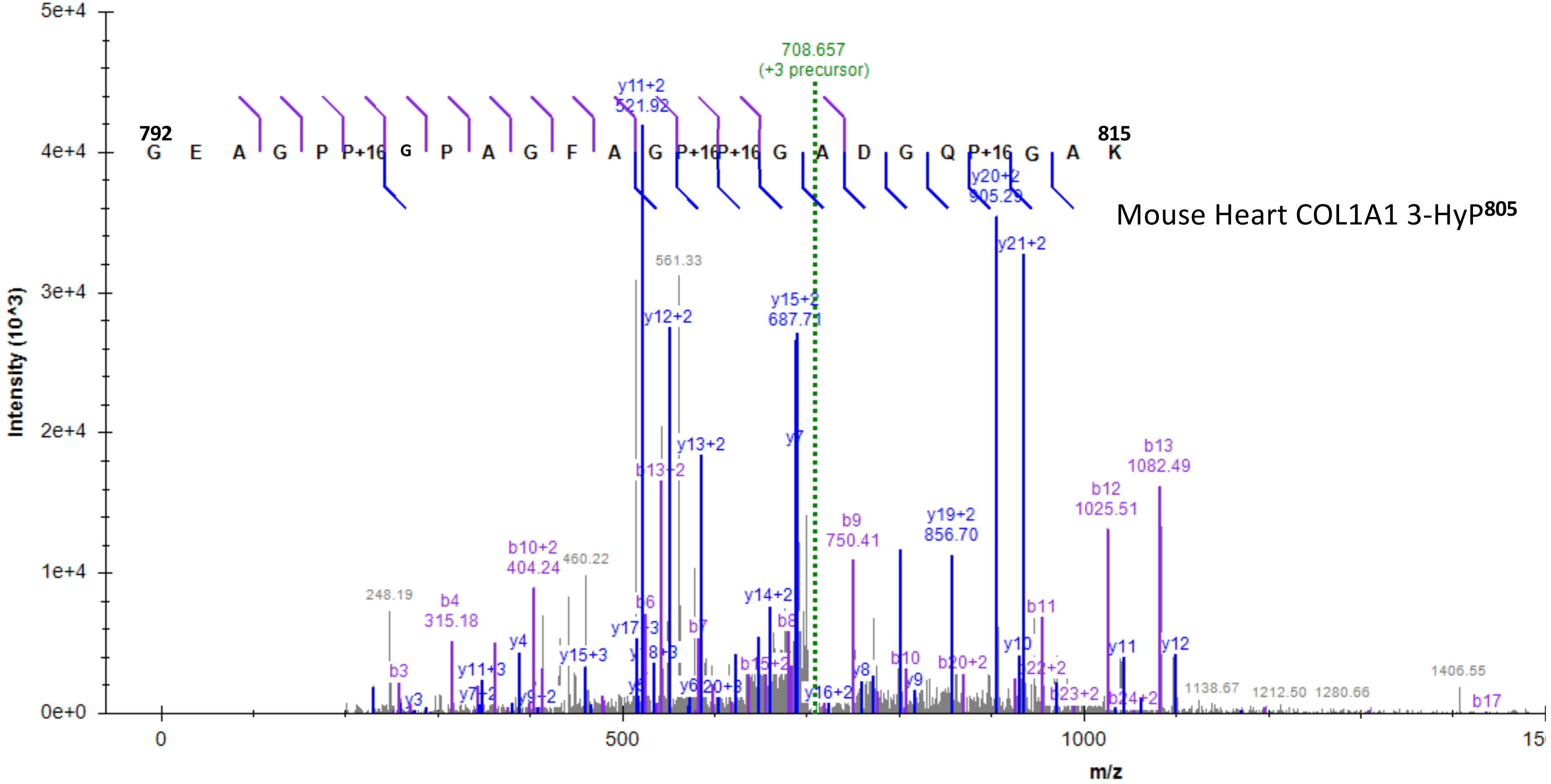
Mouse Heart COL1A1 3-HyP805
792
G E A G P P+16 G P A G F A G P+16 P+16 G A D G Q P+16 G A K
815
708.657
(+3 precursor)
Intensity (10^3)
m/z
5e+4
4e+4
3e+4
2e+4
1e+4
0e+0
0
500
1000
y11+2
521.92
y20+2
905.29
y21+2
561.33
y12+2
y15+2
687.71
y13+2
b13-2
b13
1082.49
b12
1025.51
b9
750.41
y19+2
856.70
b10+2
404.24
460.22
248.19
b4
315.18
y14+2
b11
y4
y15+3
y10
y11
y12
1138.67
1212.50
1280.66
1406.55
b17

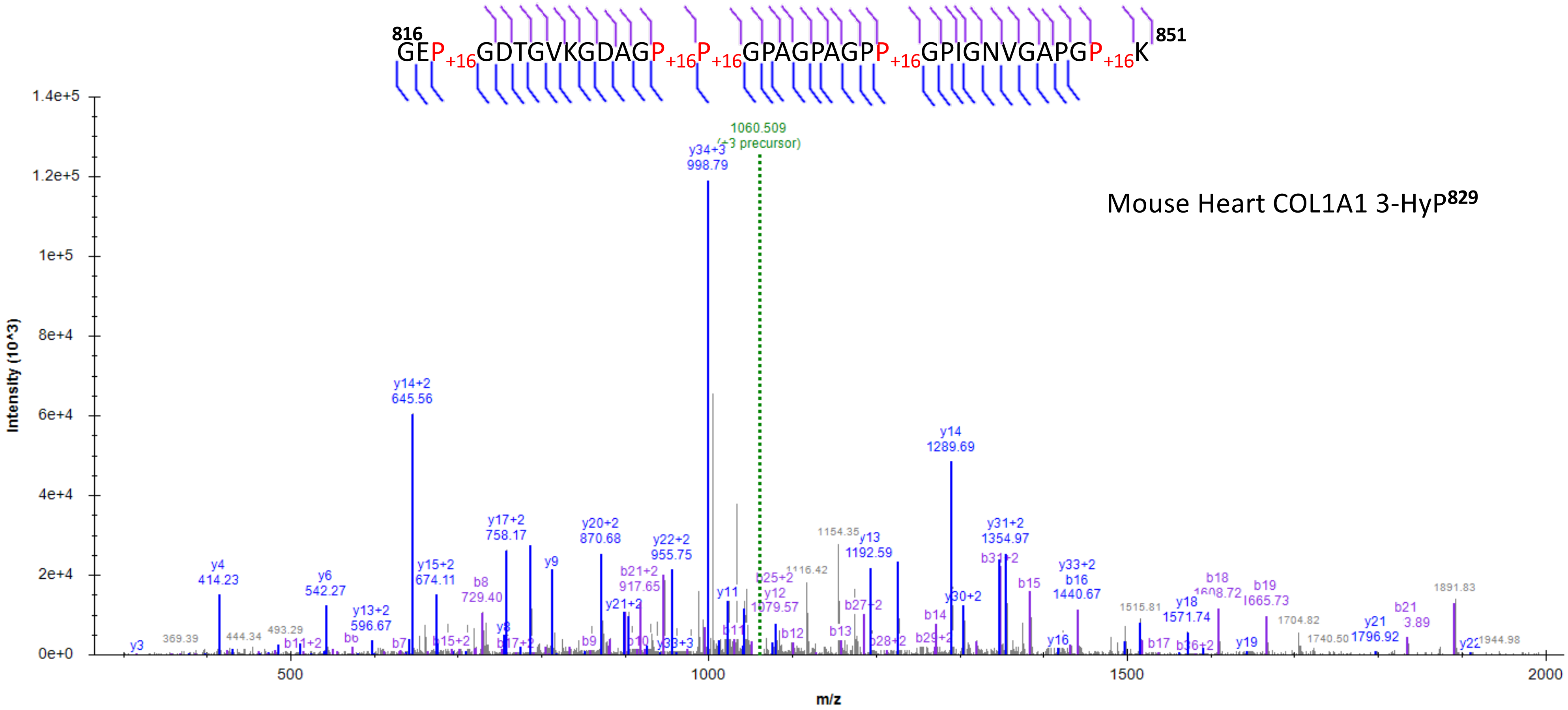
816 GEP+16GDTGVKGDAGP+16P+16GPAGPAGPP+16GPIGNVGAPGP+16K 851
Mouse Heart COL1A1 3-HyP829
1060.509 (+3 precursor)
Intensity (10^3)
m/z

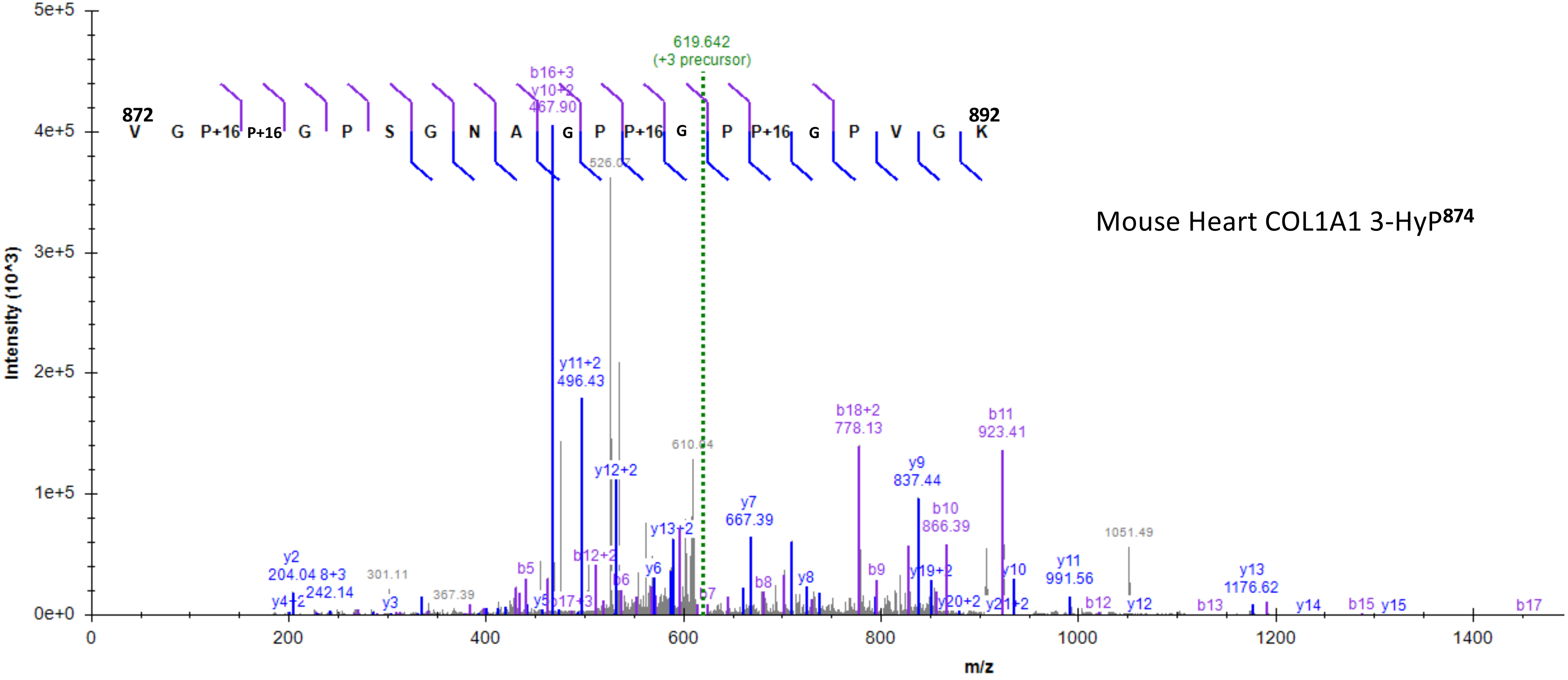
Mouse Heart COL1A1 3-HyP874
872
V G P+16 P+16 G P S G N A G P P+16 G P P+16 G P V G K
892
619.642
(+3 precursor)
Intensity (10^3)
m/z
y11+2
496.43
b18+2
778.13
b11
923.41
y9
837.44
b10
866.39
y7
667.39
y12+2
y13+2
y11
991.56
y13
1176.62
1051.49
610.64
526.07
y2
204.04
242.14
301.11
367.39

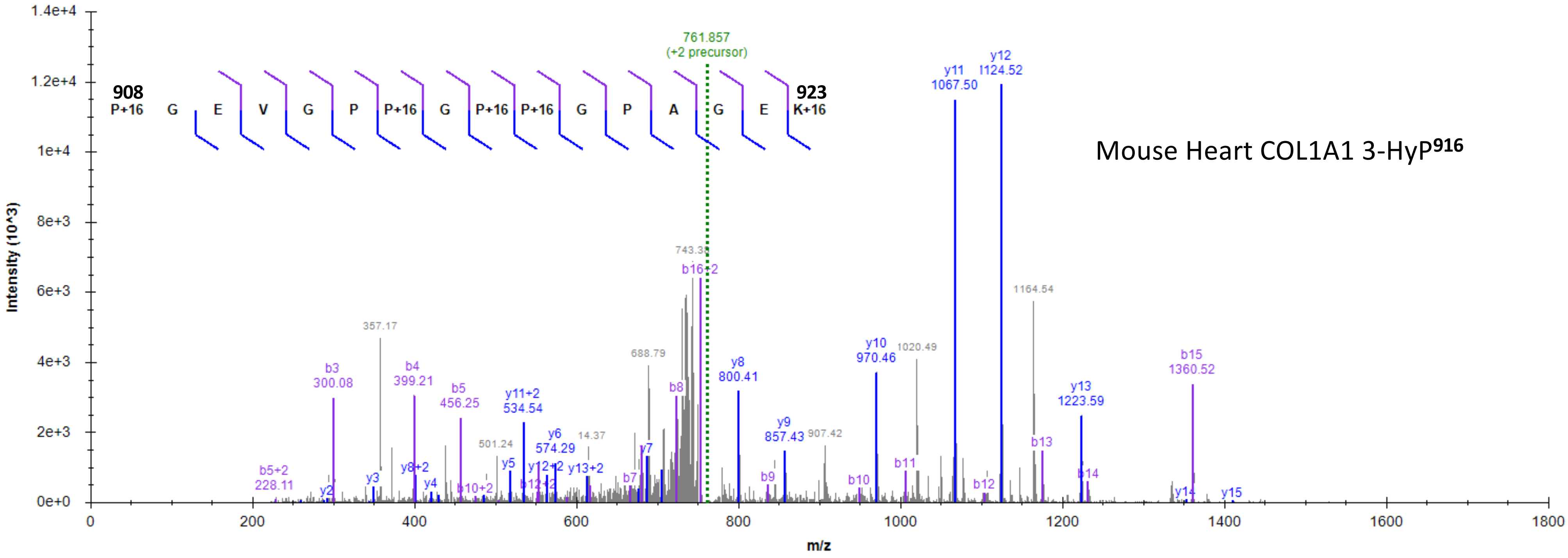

Mouse Heart COL1A1 3-HyP[916]

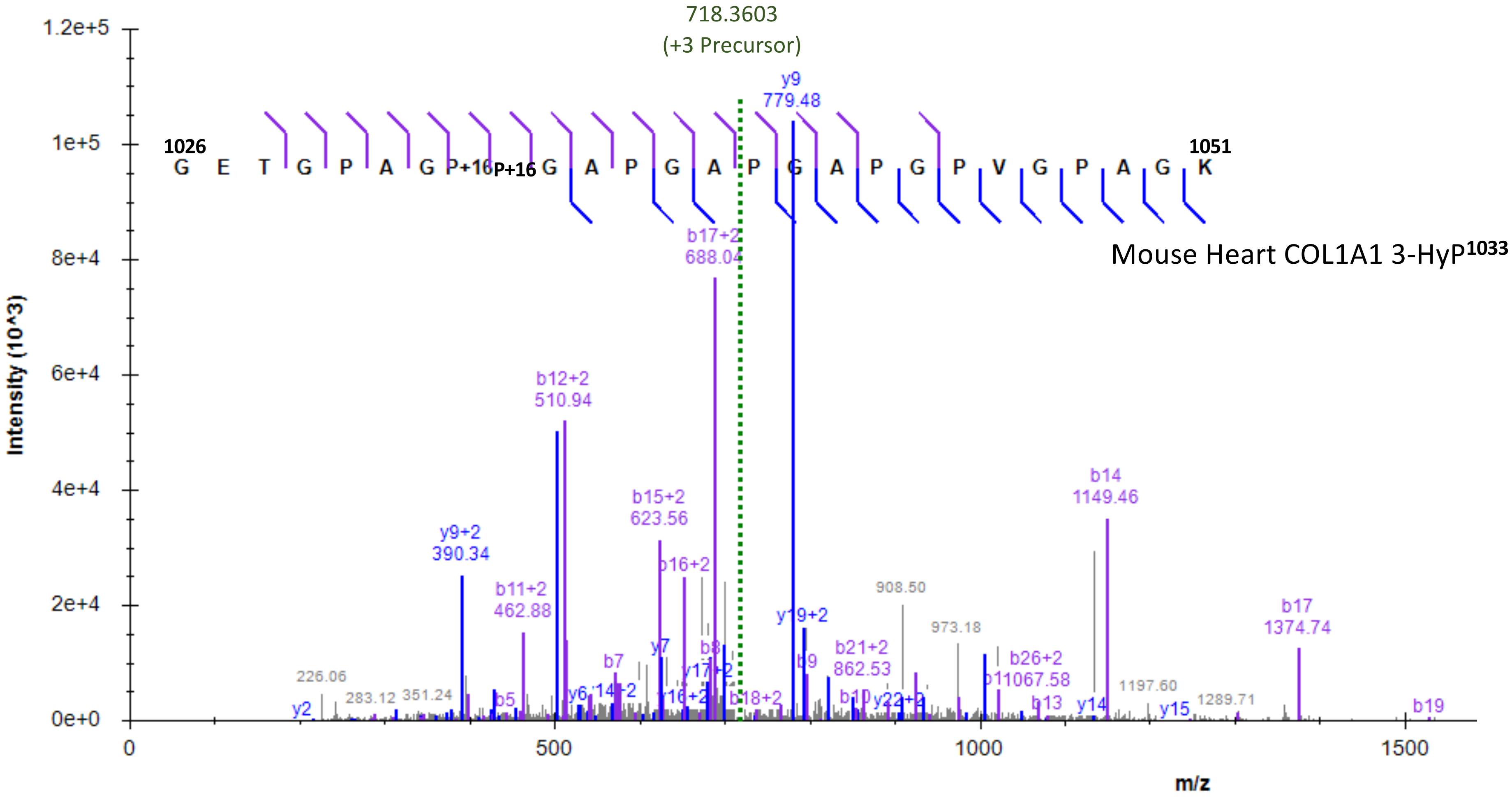
718.3603
(+3 Precursor)
Mouse Heart COL1A1 3-HyP1033
1026
G E T G P A G P+16 P+16 G A P G A P G A P G P V G P A G K
1051
y9
779.48
b17+2
688.04
b12+2
510.94
b15+2
623.56
y9+2
390.34
b11+2
462.88
b16+2
b14
1149.46
b17
1374.74
y19+2
b21+2
862.53
b26+2
b11067.58
908.50
973.18
226.06
283.12
351.24
1197.60
1289.71
y2
b5
y6
b7
y7
b8
b9
b10
b13
y14
y15
b19
b18+2
Intensity (10^3)
m/z
1.2e+5
1e+5
8e+4
6e+4
4e+4
2e+4
0e+0
0
500
1000
1500

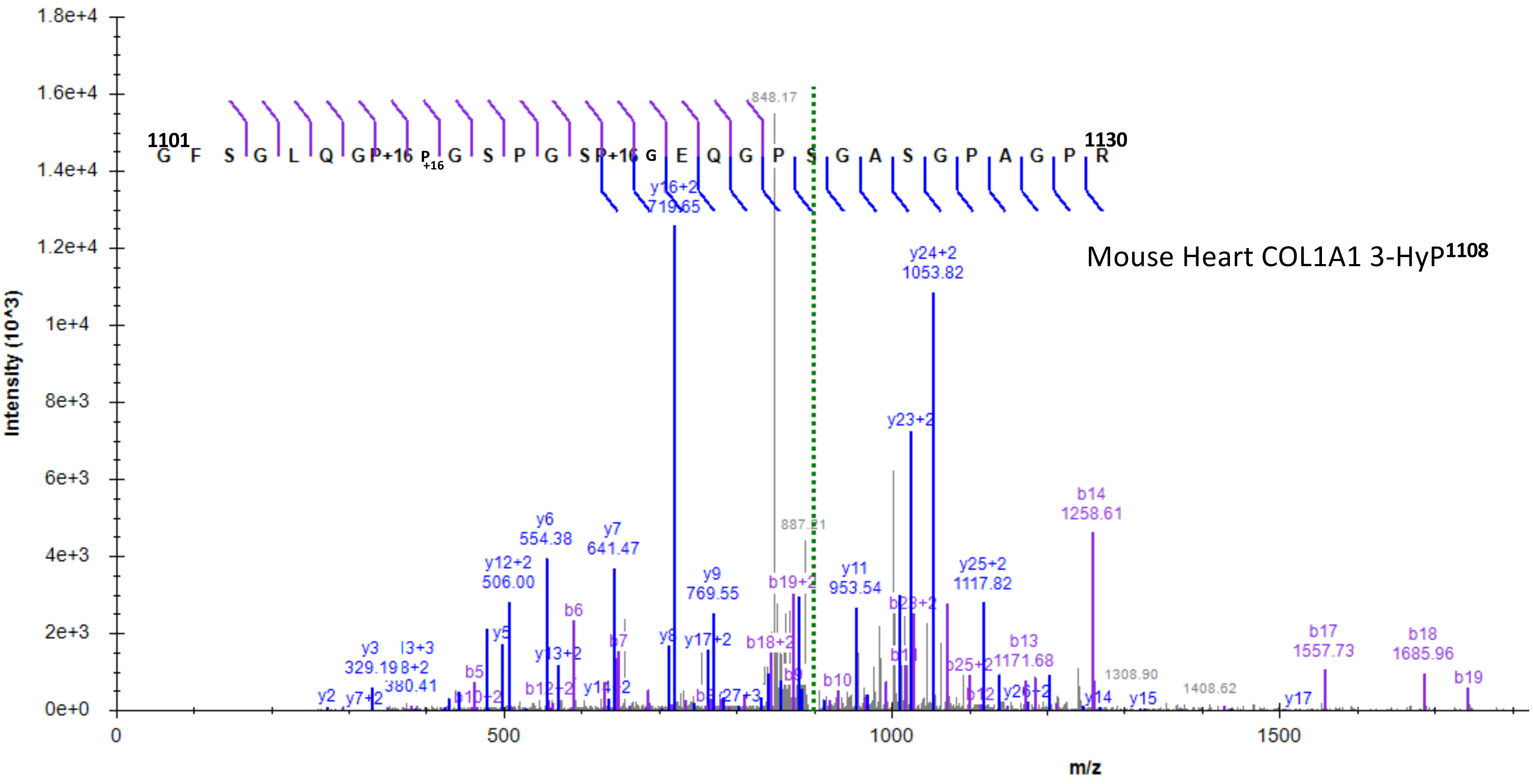
Mouse Heart COL1A1 3-HyP1108
1101
1130
G F S G L Q G P+16 P+16 G S P G S P+16 G E Q G P S G A S G P A G P R
Intensity (10^3)
m/z
y16+2
719.65
848.17
y24+2
1053.82
y23+2
b14
1258.61
y6
554.38
y7
641.47
887.21
y12+2
506.00
y9
769.55
b19+2
y11
953.54
y25+2
1117.82
b6
b13
1171.68
b17
1557.73
b18
1685.96
b19
y3
329.19
380.41
1308.90
1408.62
y2
y15
y17
b10

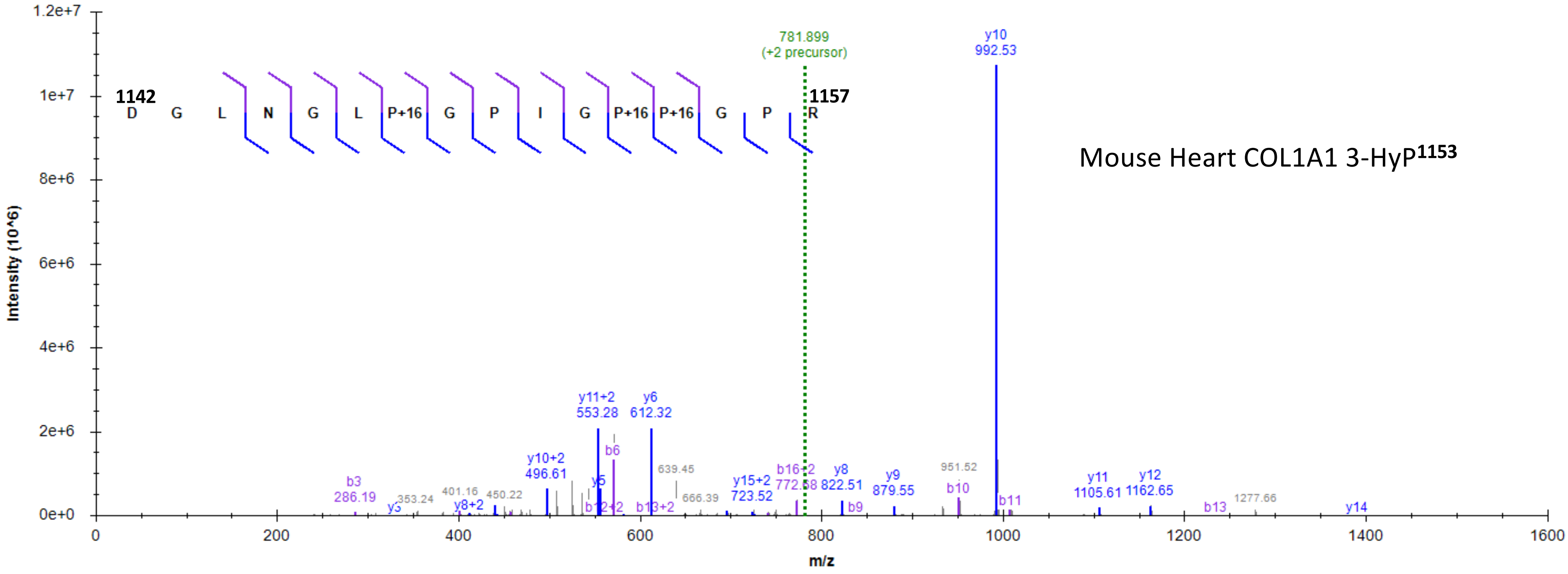
1142
D G L N G L P+16 G P I G P+16 P+16 G P R
1157
781.899
(+2 precursor)
y10
992.53
Mouse Heart COL1A1 3-HyP1153
Intensity (10^6)
m/z
y11+2
553.28
y6
612.32
y10+2
496.61
b6
639.45
b3
286.19
353.24
401.16
450.22
y8+2
y5
666.39
y15+2
723.52
b16+2
772.58
y8
822.51
y9
879.55
951.52
b10
b9
b11
y11
1105.61
y12
1162.65
b13
1277.66
y14

Supplementary Figure 4: Raw intensities of LH2 (reported by Ma et.al) corresponding to each time point (n=4) each were used in this plot to observed the changes during post-amputation. ANNOVA was used to test the significance of relative changes across time-points (*** <0.001).

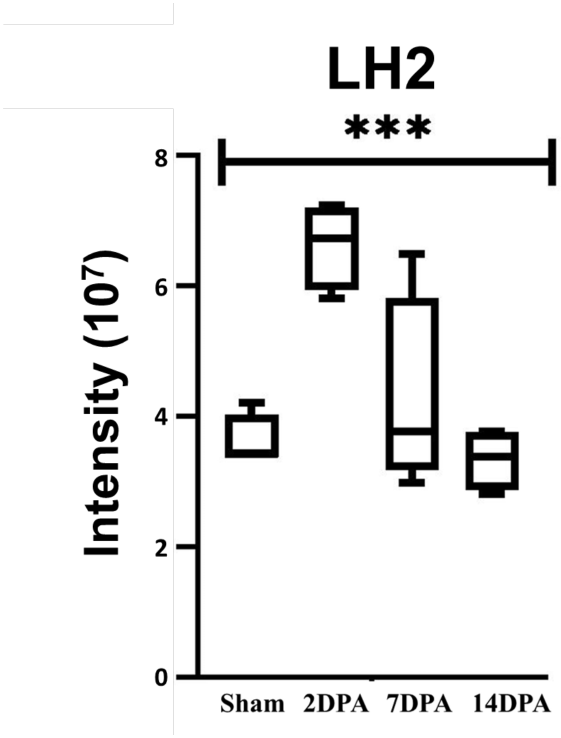

Supplement: Supplementary file 1 [file DataSheet1.PDF]
